# Zero-shot design of drug-binding proteins via neural iterative selection−expansion

**DOI:** 10.1038/s41586-026-10670-w

**Published:** 2026-06-24

**Authors:** Benjamin Fry, Kaia Slaw, Nicholas F. Polizzi

**Affiliations:** 1 Harvard Graduate Program in Biophysics, Harvard University, Boston, MA USA; 2 Department of Cancer Biology, Dana-Farber Cancer Institute, Boston, MA USA; 3 Department of Biological Chemistry and Molecular Pharmacology, Harvard Medical School, Boston, MA USA

**Keywords:** Protein design, Computational biophysics

## Abstract

The design of proteins that bind to small molecules has been challenging because it requires simultaneous optimization of the protein sequence, protein structure and ligand conformation^1–7^. Current deep-learning algorithms have struggled to navigate this landscape, precluding the zero-shot design of binders. Here we show that by combining two neural networks in an iterative design algorithm, small-molecule binding proteins can be created from scratch with high accuracy. We trained a graph neural network—ligand-aware sequence engineering message-passing neural network (LASErMPNN)—to design compatible protein sequences for an input protein backbone and docked ligand. We paired  LASErMPNN with a structure predictor that models a three-dimensional protein–ligand complex for an input protein sequence and ligand identity. The closed-loop iteration of these reciprocal networks optimized sequence–structure–ligand compatibility, and outperformed a comparable design loop using a physics-based energy function. We used our strategy, termed neural iterative selection–expansion (NISE), to design proteins that, using different folds, specifically bind to two chemically distinct small-molecule drugs, exatecan and apixaban, with success rates of 100% and 83%, respectively. The tightest NISE binders had nanomolar-to-picomolar affinities, surpassing those of the next-leading method by 70-fold for exatecan and nearly 10,000-fold for apixaban. LASErMPNN then suggested two amino-acid substitutions that improved the affinity of the tightest exatecan binder by 100-fold without any experimental input. The optimized binder protected the labile lactone ring of exatecan from hydrolysis for days. Our work describes a general recipe for using neural networks to automate the design of small-molecule binding proteins for applications in drug delivery, sensing and catalysis.

## Abstract (web summary)

By pairing two neural networks in an iterative optimization algorithm, small-molecule binding proteins can be designed from scratch with high accuracy, affinity and success rates, showing promise for applications in drug delivery and sequestration.

## Main

The de novo design of small-molecule binding proteins remains a considerable challenge^1,2,5,6,8–14^, despite rapid progress elsewhere in the field^15–26^. Notable successes^1–4,6,13,27–29^ have relied mainly on high-throughput experimental selection. The few cases^3,4^ with high computational hit rates (33%) approximated the functional groups of ligands as parts of amino acids. To generalize binder design, neural networks can in principle learn directly from training data to predict protein sequences and protein–ligand co-structures.

**Fig. 1:**
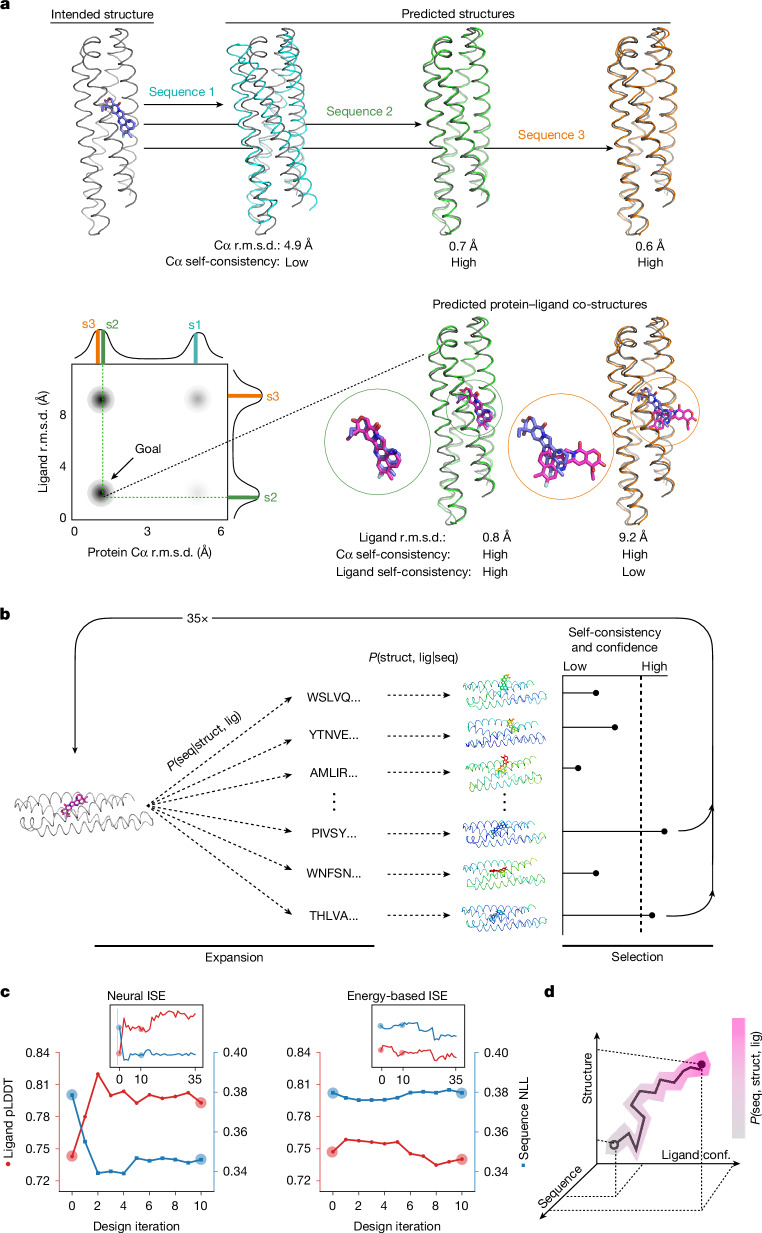
A self-consistency optimization algorithm for the design of small-molecule binding proteins. **a**, The principle of self-consistency shown for a four-helix bundle and a docked small molecule (shown as a stick model in blue). Top, two self-consistent designs with respect to backbone, computed with, for example, AF2. Bottom, one of these self-consistent backbone designs is self-consistent with respect to the ligand (predicted docked ligand in magenta), computed using, for instance, RFAA. The schematic heat map shows a typical outcome for a NISE trajectory with the goal to populate the bottom left corner. Marginal distributions are shown using coloured bars for protein sequences 1–3 (s1–s3). **b**, Schematic of the NISE protocol. NISE starts with an initial protein structure (backbone coordinates only) and a docked ligand location (magenta). The NISE process iteratively applies neural-network-based sequence design (expansion) and co-structure prediction. lig, ligand; *P*, probability; seq, sequence; struct, structure. High-confidence (high ligand pLDDT) self-consistent designs are used as new inputs (selection) for sequence design. The depicted proteins and ligands are coloured by model confidence (low to high, red– yellow–green–cyan–blue). **c**, Comparison of neural and energy-based iterative selection–expansion (ISE) protocols for generating modified protein–ligand coordinates. In both cases, LASErMPNN was used to design the sequences. For energy-based ISE, the co-structure predictor was replaced with Rosetta energy minimization and designs were selected on the basis of low ligand energy. After 35 rounds of ISE, the structures of all designed sequences were predicted using RFAA. Ligand pLDDT (red, third quartile) and sequence negative log-likelihood (NLL; blue, first quartile) were plotted against design iteration. NISE (but not energy-based ISE) simultaneously optimized both ligand confidence (higher pLDDT) and protein sequence quality (lower NLL). Data are from a NISE trajectory using the input structure that produced EPIC (Fig. 3) and with exatecan used as the ligand. Quartiles were produced from *n* = 1,500 designs per iteration (*n* = 500 for the first round). **d**, Simultaneous optimization of designs along two reciprocal conditional-probability distributions, *P*(seq|struct, lig) and *P*(struct, lig|seq), in **c** suggests that NISE is optimizing within the joint probability distribution, *P*(seq, struct, lig). conf., conformation.

Self-consistency describes the agreement between the intended structure of a designed sequence and its predicted structure. Maximizing self-consistency has been a guiding principle for the design of new topologies^30^ and of binders to proteins and peptides^15,16,23,31^. However, this principle has not been extended to small-molecule binder design because of the complexities of encoding non-amino-acid chemistry. Models such as RoseTTAFold-All Atom (RFAA)^8^, Boltz-1/2 (refs. ^32,33^) and AlphaFold3 (AF3) (ref. ^34^) can predict a protein–ligand co-structure from a protein sequence and a ligand simplified molecular-input line-entry system (SMILES) string. Using such models, a self-consistent design has not only a predicted structure that closely resembles the intended backbone, but also a ligand that is predicted to bind in the intended site (Fig. 1a). This additional dimension should enable a more nuanced assessment of design quality. We reasoned that maximizing sequence–structure–ligand self-consistency would lead to the design of high-affinity small-molecule binding proteins with good success rates.

To design binders, we implemented a self-consistency optimization algorithm, NISE, which explicitly considers small-molecule ligands. We applied NISE to two small-molecule drugs, exatecan and apixaban, and achieved highest affinity binders with dissociation constants (*K*_d_) of 120 nM and 80 pM, respectively. These *K*_d_ values surpass other methods^4,6^ considerably.

## NISE sampling algorithm

The de novo design of small-molecule binding proteins typically begins by docking a ligand into a precomputed protein scaffold, and then designing a sequence for the resulting pose^1,4^. Because the initial pose is rarely optimal, the designed sequence is also unlikely to be optimal. Therefore, a means of jointly refining the sequence, backbone and ligand conformation in a coupled manner is needed. A few methods, such as COMBS^3,4^, RIFdock^2,6^ and even brute-force rigid-body docking (Supplementary Information), can position ligands into a blank backbone for subsequent sequence design, with optional constraints on ligand orientation and burial. NISE then performs the joint refinement.

To maximize tripartite self-consistency for a given input structure (backbone and docked ligand), we iteratively designed sequences and predicted co-structures (Fig. 1b). For each sequence, a protein–ligand co-structure was computed and compared to the input structure from the previous round. Only designs with high self-consistency (low root mean square deviation (r.m.s.d.)) with respect to both backbone and ligand coordinates were kept. We then selected a few co-structures with the most confidently predicted ligands as new inputs (both backbone and ligand atoms) for the next round of sequence design. Throughout the NISE process, we avoid using energy functions, relying instead on neural networks and model confidence to guide optimization. To avoid getting trapped in local minima, we encouraged exploration by sampling many sequences for a given backbone–ligand pair in each round, drawing from the probability distribution of the LASErMPNN neural network (see below) at a high softmax temperature. The structures, sequences and ligand conformers become progressively more compatible after each round (Fig. 1c, left).

This approach is similar to iterative coordinate ascent^35^, in which alternating argmax sampling from two conditional distributions, *P*(*a*|*b*) and *P*(*b*|*a*), locally climbs to a high probability mode in the joint distribution, *P*(*a*,* b*). NISE samples broadly from *P*(sequence|structure, ligand conformation) and takes a confidence-based argmax over *P*(structure, ligand conformation|sequence). Together, this means that NISE climbs towards a high probability mode in the joint distribution representative of the underlying training data, protein–ligand co-structures in the Protein Data Bank (PDB; Fig. 1d).

## LASErMPNN neural network

**Fig. 2:**
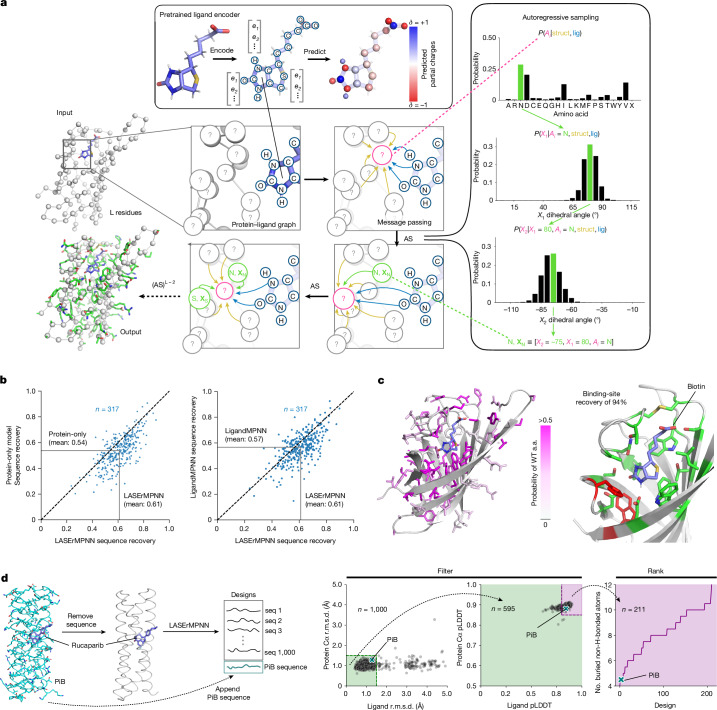
LASErMPNN designs protein sequences conditioned on protein–ligand co-structures. **a**, LASErMPNN is an encoder–decoder network. It uses protein–ligand co-structures as input (protein backbone atoms and ligand atoms) and is trained to autoregressively predict (box labelled autoregressive sampling) the amino-acid identity and side-chain dihedral angles of each protein residue. LASErMPNN forms a heterograph from the protein–ligand co-structures, encoding the protein and ligand nodes separately. The ligand node embeddings (*e*_1_, *e*_2_, etc) are taken from a pretrained ligand encoder (box labelled pretrained ligand encoder) and locked during training. The ligand encoder is trained to predict the atomic properties of ligands (such as the partial atomic charge) conditioned on atomic coordinates and elements. Autoregressive sampling (AS) is performed in turn for each L residue of the protein. Each new residue prediction is conditioned on previously decoded residue identities and their associated dihedral angles (green). LASErMPNN outputs a three-dimensional structure of the sampled side-chain coordinates built from predicted residue identities (*A*_*i*_) and side-chain dihedral angles (*X*_1_, *X*_2_, *X*_3_ and *X*_4_). **b**, LASErMPNN performance on a held-out test set of proteins compared with a similarly trained protein-only model (left, all ligand information removed) and a retrained LigandMPNN model (right). These plots show the prediction accuracy across binding-site residues when the model is tasked with predicting the entire protein sequence of each test protein (argmax sampling). **c**, Left, LASErMPNN scores the wild-type (WT) sequence of monomeric streptavidin favourably, with high-probability amino acids (a.a.; shown in magenta) located in the biotin binding site and protein core. Right, the LASErMPNN design with highest binding-site sequence recovery (out of 10,000 sequences). Accurately predicted residues are depicted in green and two designed mutations in red. This design was in the top 25 when ranked by buried, non-hydrogen-bonded polar atoms. **d**, LASErMPNN designs using the PiB structure (PDB: 8TN6_A) as the input. RFAA-predicted co-structures for 1,000 designs were filtered and ranked by the number of buried, polar non-hydrogen-bonded atoms. The original high-affinity PiB sequence was ranked fourth.

We set out to train a neural network to learn the probability distribution of protein sequences conditioned on the three-dimensional structures of protein–ligand complexes. LASErMPNN is a heterograph neural network trained on protein–ligand co-crystal structures from the PDB. It predicts protein sequences and side-chain dihedral angles from protein backbone coordinates and ligand atomic coordinates (Fig. 2a and Supplementary Fig. 1). During inference, LASErMPNN autoregressively decodes each residue from an input protein–ligand complex using a randomly chosen decoding order. We trained the model until the accuracies of the training and validation sets overlapped. We then tuned the hyperparameters of the model to maximize foldability of the designed sequences without markedly losing sequence recovery (Supplementary Fig. 2). The model accurately recovered native protein sequences in a held-out test set, outperformed a similarly trained ligand-free version of itself, and was comparable to (even slightly outperformed) a retrained version of a similar model, LigandMPNN^36^ (Fig. 2b).

**Extended Data Fig. 1:**
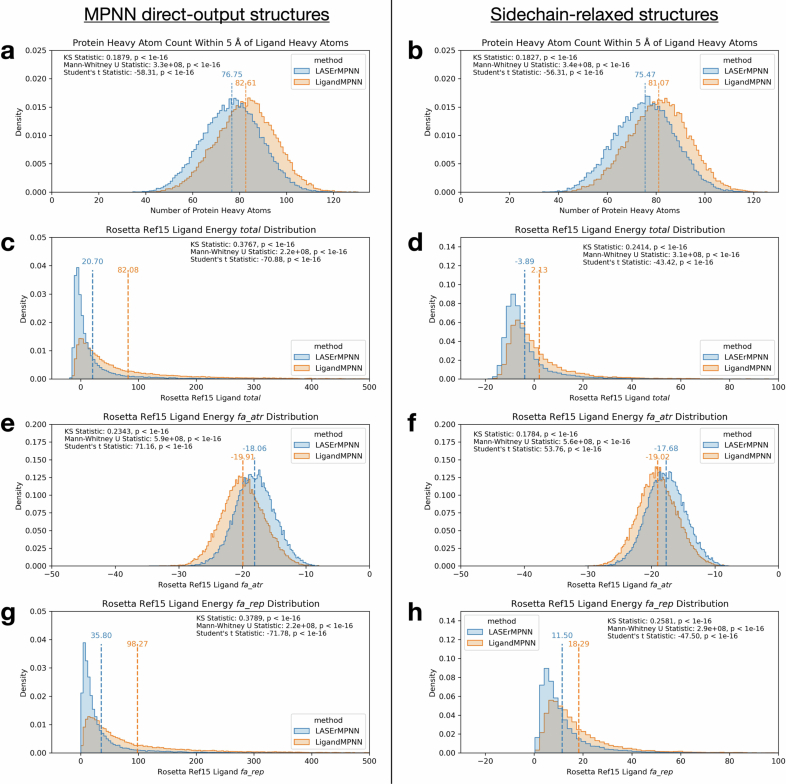
LigandMPNN generates binding-site sequences that are more overpacked than those from LASErMPNN. We assessed direct outputs and Rosetta-relaxed (sidechains only) outputs from both LASErMPNN and LigandMPNN on a diverse set of apixaban–NTF2 docked poses generated by CARPdock (30,000 generated sequences total across 10,000 poses). **a**, LigandMPNN packer places more protein heavy atoms within 5.0 Å of the ligand than LASErMPNN. **b**, After Rosetta fixed backbone, fixed ligand Relax protocol, LigandMPNN places more atoms within 5.0 Å of ligand heavy atoms. **c**,**d**, LASErMPNN sequences have lower (more favorable) total ligand energies than LigandMPNN sequences before and after a sidechain-only Rosetta Relax protocol (fixed ligand, fixed backbone). While LigandMPNN sequences have lower ligand attractive van der Waals energies (fa_atr) than LASErMPNN sequences before and after a sidechain-only Rosetta Relax protocol (**e**,**f**), LigandMPNN sequences have substantially higher ligand repulsive van der Waals energies (fa_rep) than LASErMPNN sequences both before and after a sidechain-only Rosetta Relax protocol (**g**,**h**). This makes the packed ligands from LigandMPNN higher energy on average (i.e. more energetically unfavorable) than those from LASErMPNN (**c**,**d**). For these computations, hydrogens were stripped from all protein and ligand structures. Both LASErMPNN and LigandMPNN models output packed sidechain coordinates directly. Direct MPNN outputs and Rosetta-Relaxed outputs were scored by Rosetta score_jd2 and Relax protocols, respectively. Differences in both means and empirical distributions are statistically significant for all panels (Student’s t-test, Mann-Whitney U-test, and Kolmogorov-Smirnov test; p < 1e-16; all statistical tests two-sided).

LASErMPNN differs from LigandMPNN in several key ways. LASErMPNN contains a distinct, pretrainable ligand encoder; performs simultaneous decoding of side-chain dihedral angles and amino-acid identity; and includes ligand nodes in each round of encoding and decoding. To enable accurate decoding of side-chain dihedrals in the presence of backbone noise, we first idealized and then noised entire backbone ‘frames’. This was necessary to reduce the memorization of crystal-structure artefacts, and contrasts with the approach of LigandMPNN, which applies noise to backbone atoms independently (Supplementary Information). Ablation studies showed that the performance of LASErMPNN depends on the simultaneous prediction of side-chain dihedral angles during sequence decoding and on pretraining a ligand encoder module using a large set of synthetic ligands^37,38^. The training task of the ligand encoder was to predict atom-level properties derived from quantum chemical computations, such as partial charge (Extended Data Table 1). A useful by-product of ligand-encoder pretraining is that predicted partial charges provide a diagnostic read-out of the model’s understanding of new ligands, a capability that LigandMPNN does not have. In a head-to-head comparison between LASErMPNN and LigandMPNN, we observed a general tendency for LigandMPNN to produce designs that were more overpacked, as measured by an increased density of protein heavy atoms (within 5 Å of the ligand) and a higher van der Waals repulsion energy of both the ligand and overall structure (Kolmogorov–Smirnov test, *P* < 1 × 10^−16^; Extended Data Fig. 1).

**Extended Data Fig. 2:**
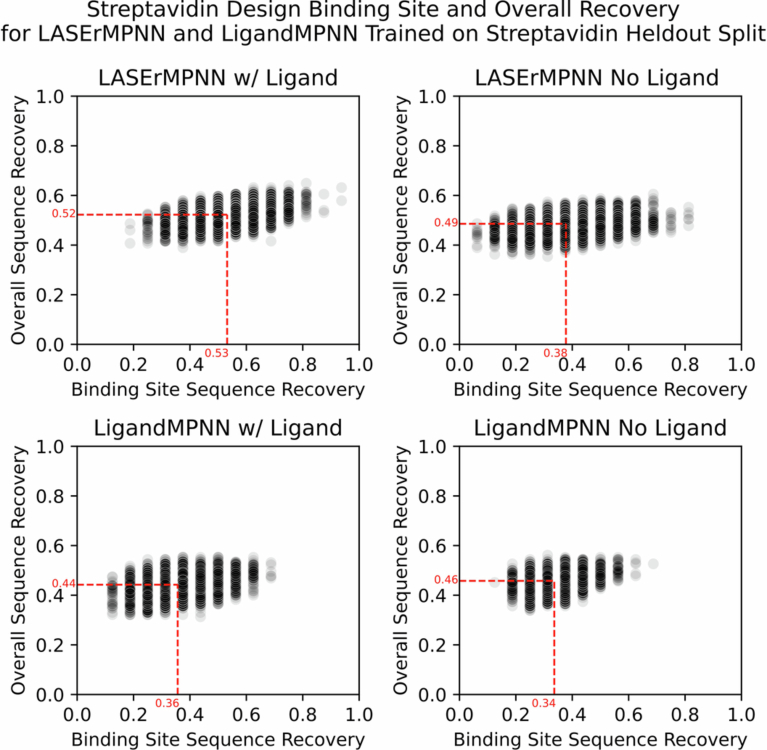
A LASErMPNN model trained on the streptavidin-holdout dataset outperforms a reimplementation of the LigandMPNN model trained on the same dataset. We used LASErMPNN and a retrained LigandMPNN to design sequences for monomeric streptavidin (PDB 4jnj), with and without the bound biotin. The mean of overall sequence recovery and binding-site sequence recovery are plotted as red dashed lines. Each plot shows 10,000 sequences sampled by each model at overall temperature 0.1 and binding-site temperature 1.0 with (left) and without (right) the biotin ligand. Specifically, we designed 2,500 sequences for each of 4 biological assemblies (monomers) in the PDB 4jnj.

As a strict in silico test case, we rigorously held out the streptavidin fold (on the basis of sequence, structure and evolutionary similarity) from the training set. The streptavidin–biotin complex is one of the strongest known non-covalent complexes in nature^39^, and recovery of this sequence would be a convincing indication that LASErMPNN is performing as intended. LASErMPNN scored the native sequence of monomeric streptavidin favourably^40^, displaying high probabilities for native core and binding-site residues (Fig. 2c). Designed sequences for monomeric streptavidin with bound biotin showed high sequence recovery of binding-site residues (53% and 38% on average, with and without biotin, respectively, Extended Data Fig. 2). Although RFAA struggled to predict self-consistent structures for this β-barrel topology, many of the designed sequences were predicted by Boltz-1 to fold without using multiple sequence alignments (65% success rate for Boltz-1 versus 0.4% for RFAA; protein Cα and ligand heavy-atom r.m.s.d. < 2.5 Å, Supplementary Fig. 3). These results contrast with a retrained model of LigandMPNN, which had an average binding-site sequence recovery of 36% and 34% with and without biotin, respectively, and a 13% success rate for self-consistent structures by Boltz-1 (Extended Data Fig. 2 and Supplementary Fig. 4). We ranked LASErMPNN-designed self-consistent structures using a formula that penalizes buried, non-hydrogen-bonded, polar atoms. This showed an inverse correlation with binding-site sequence recovery (Supplementary Fig. 5). The top-25 streptavidin designs showed up to 94% binding-site sequence recovery (out of 10,000 total designs; Fig. 2c). The few residues that were in disagreement were biochemically plausible and represented in a multiple sequence alignment (Supplementary Information). Thus, LASErMPNN can rapidly sample binding-site sequences that have taken millions of years to evolve.

We tested the same filtering and ranking approach on a previously de novo designed drug-binding protein^3^, PiB, a four-helix bundle that tightly binds to the drug rucaparib (Fig. 2d and Supplementary Fig. 6). The structure of PiB was held out from the training sets of both LASErMPNN and RFAA, and therefore represents a benchmark closely aligned with our design objective. We used LASErMPNN to design 1,000 sequences for the PiB backbone bound to rucaparib. Most designed sequences had self-consistent co-structures predicted by RFAA. We filtered RFAA-folded designs by self-consistency (protein Cα and ligand r.m.s.d.) and ligand confidence (predicted local distance difference test, pLDDT), then ranked the remaining designs using the same formula that penalizes buried, non-hydrogen-bonded, polar atoms. The top-ranked design had a binding-site sequence recovery of 74% (differing mainly at surface residues) and a recovery of 80% for all core residues. This provides confidence that the design method and ranking scheme can produce and prioritise high-quality de novo binders. When assessed using the same metrics, the PiB sequence was ranked near the top of the designed sequences (fourth out of 1,001).

## Exatecan design objective

**Fig. 3:**
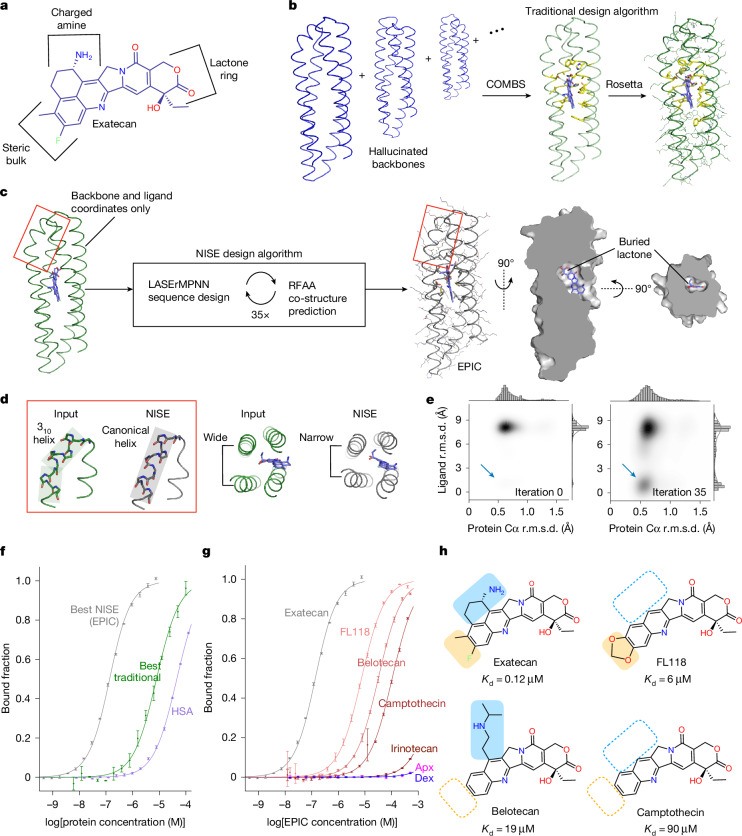
Design of an exatecan-binding protein using NISE. **a**, Chemical structure of exatecan. **b**, Traditional design algorithm using COMBS and Rosetta to design an exatecan-binding protein. Starting from an ensemble of computationally generated four-helix bundles (coloured by AF2 Cα pLDDT; blue > 90), COMBS uses van der Mers (yellow) to dock exatecan (blue) into the bundle. A flexible-backbone design protocol using Rosetta then designs the remainder of the sequence. **c**, The NISE design algorithm uses backbone and ligand coordinates as the input. The sequence of the traditional design in **b** was stripped and its structure was used as input for NISE. Four designs were chosen from the NISE output. The design model for EPIC, the highest-affinity protein, is shown in grey. **d**, Structural refinement of the design model using NISE (grey); the input structure (green) is shown for comparison. A 3_10_ helix (green) near the ligand (red box in **c**) is converted to a canonical helix (grey) and a wide helix–helix interface (green) is narrowed. Exatecan (blue) is drawn deeper into the bundle. **e**, Density plots of protein and ligand r.m.s.d. values for two iterations of the NISE protocol using the input structure in **c**. Note the increase in protein–ligand self-consistency in iteration 35 compared with iteration 0 (blue arrow). Marginal distributions are shown (*n* = 4,000). **f**, Fluorescence anisotropy of exatecan as a function of titrating different proteins (converted to the fraction of bound exatecan (bound fraction)): the highest-affinity NISE design (EPIC, *K*_d_ = 0.12 ± 0.03 µM), the highest-affinity traditional design (*K*_d_ = 8 ± 0.7 µM) and HSA (*K*_d_ = 43 ± 3 µM). [Exatecan] = 50 nM; fits to a 1:1 binding model are shown as solid lines. **g**, Fluorescence anisotropy of several fluorescent ligands binding to EPIC. [Ligand] = 50 nM; fits to a 1:1 binding model are shown as solid lines. Irinotecan showed negligible binding at the highest protein concentrations, precluding an accurate fit. Dexamethasone (dex) and apixaban (apx) were conjugated to fluorescein isothiocyanate (FITC) and showed no appreciable binding. **f**,**g**, Data are mean ± s.d. of three technical replicates.** h**, Structure–activity relationship for the series of camptothecin-class molecules shown in **g**. The change in binding free energy (ΔΔ*G*) was computed relative to the affinity of the exatecan–EPIC complex. FL118, ΔΔ*G* = 2.3 kcal mol^−1^; belotecan, ΔΔ*G* = 3.0 kcal mol^−1^; and camptothecin, ΔΔ*G* = 3.9 kcal mol^−^^1^.* K*_d_ values represent the mean of 1,000 fits performed by bootstrapping of optimal-fit residuals; bounds are 95% confidence intervals derived from the bootstrapped fits.

**Extended Data Fig. 3:**
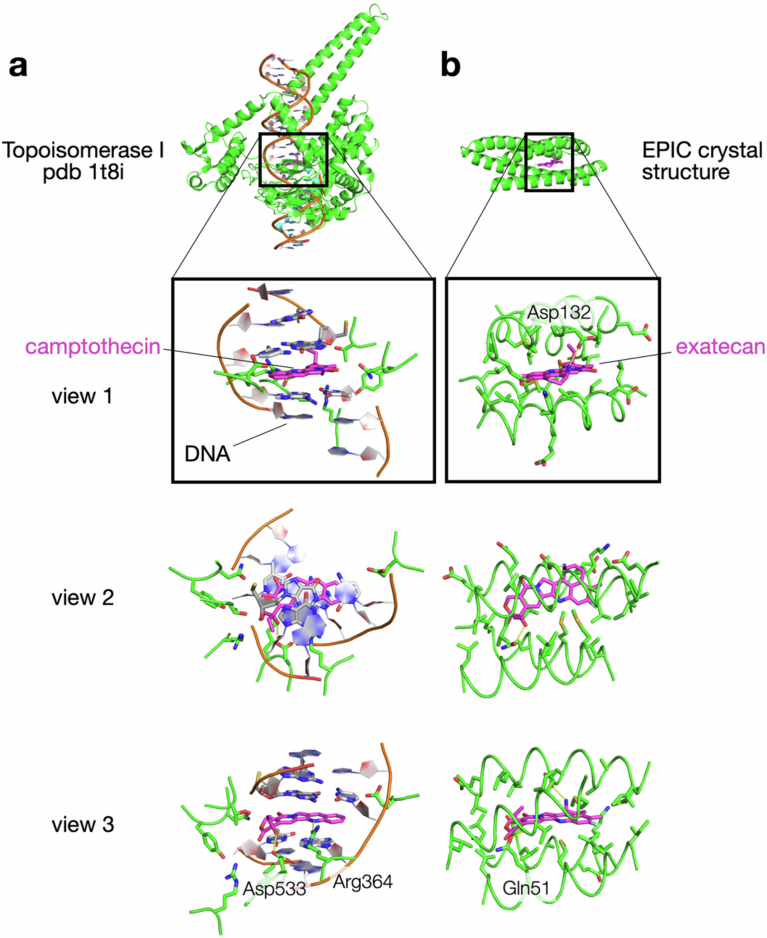
Binding sites of camptothecin in Topoisomerase I and exatecan in EPIC are dissimilar. The crystal structures of Topoisomerase I (left, **a**) and EPIC (right, **b**) were aligned by ring atoms in camptothecin and exatecan, respectively. Each row shows the same view of the ligands (magenta) in the two proteins. Residues with heavy atoms within 5 Å of ligand heavy atoms are shown as stick representation. Residues that participate in H-bonding interactions with the ligand are labeled. The binding site of camptothecin in Topoisomerase I is highly dominated by DNA, in which the ligand is intercalated via π-stacking interactions. In contrast, the binding site of exatecan in EPIC is exclusively comprised of protein interactions, where aliphatic sidechains and Gly Cα atoms pack against exatecan.

Exatecan is a member of the camptothecin class of clinically approved small-molecule anticancer therapeutics. It binds to and inhibits^41^ the DNA–protein complex of topoisomerase I (Fig. 3a). It is one of the most soluble and potent members of the camptothecin class (cellular half-maximum inhibitory concentration (IC_50_) of around 1 nM) and is therefore a popular payload in antibody–drug conjugates^42,43^. Like other camptothecins, exatecan has a characteristic lactone ring that is prone to hydrolysis at physiological pH. In plasma, the free drug is hydrolysed^41^ with a half-life of around 2 h (Figs. 3a and 5a). The hydrolysed, ring-open, carboxylate form of the drug is considerably less bioactive, owing in part to its lower cell permeability and because this form is bound predominantly by human serum albumin^41,43^ (HSA). We sought to design a small protein that encapsulates the labile lactone ring of exatecan, thus protecting it from hydrolysis (Figs. 3b,c and 5c). A designed binder could be used as a delivery vehicle for the bioactive form of the drug or as a sponge for free drug molecules that were liberated from antibody–drug conjugates prematurely. There are no structures of exatecan in the PDB or the Cambridge Structural Database, and few structures of related compounds (Extended Data Fig. 3a), making this a challenging target for binder design with neural networks.

## Design of exatecan binders using NISE

We performed family-wide hallucination of single-chain four-helix bundles to create initial scaffolds for docking exatecan (Supplementary Information). Helical bundles contain a central cavity that can accommodate a variety of small molecules, and their small size, high designability^44^ and thermostability make them attractive scaffolds for de novo binder design^3,4,28,45^. In brief, we created four-helix coiled-coil domains using parametric equations^4,44^, generated loops using RFdiffusion^15^ (with loop lengths determined by structural bioinformatics^46^), designed sequences for each bundle using ProteinMPNN^47^, and predicted bundle structures with AlphaFold2 (AF2)^48^. The resulting focused library consisted of 40 AF2-predicted four-helix bundles with very high confidence (Cα pLDDT). Each structure had at least eight different sequences that passed confidence thresholds, making each sufficiently designable as a scaffold for small-molecule binders.

We generated two exatecan conformers using an X-ray crystal structure of a camptothecin derivative from the Cambridge Structural Database (CSD) as a starting point. Newly built coordinates were optimized with a molecular mechanics forcefield (Supplementary Fig. 7 and Supplementary Information). These conformers were docked into each of our 40 backbone templates using a previously described method, COMBS^3,4^. Docked ligands were filtered by burial in the protein.

The docked ligands underwent a traditional design process, first using COMBS and van der Mers^3,4^ to design the binding site, and then using Rosetta^49^ to compute the remainder of the sequence with a flexible backbone algorithm (Fig. 3b). Final designs passed metrics for ordering, such as self-consistency by co-structure prediction with RFAA (low r.m.s.d. for overall backbone, binding site and ligand). We ordered synthetic DNA for 16 designs, representing 8 distinct ligand positions and backbones (that is, poses), for a direct comparison to NISE.

For the initial input to NISE, we chose a model from the 16 COMBS–Rosetta designs. The designs were ranked by self-consistency using a linear combination of ligand heavy-atom r.m.s.d., binding-site residue Cα r.m.s.d. and overall backbone Cα r.m.s.d. values (Supplementary Information methods). The sequence of the top-ranked design was discarded and only the backbone and ligand coordinates were used as input to NISE. No experimental characterization of the designs was performed before this choice.

We implemented the NISE protocol with RFAA, optimizing designs by selecting the top three self-consistent structures by ligand confidence (pLDDT) from each round. These were then expanded in the next round by sampling 1,000 sequences for each structure (designing the entire sequence with LASErMPNN each time). Using NISE, we noticed a striking asymmetry: most designed sequences were predicted by RFAA to be self-consistent with respect to Cα coordinates, but far fewer were predicted to bind to the ligand in the correct orientation, although most ligands had an accurately predicted centre of mass (Supplementary Fig. 8). More ligands were predicted to bind in the correct orientation in later design cycles, but these remained the minority compared with alternative binding modes that tended to expose most of the polar atoms of exatecan to solvent (Fig. 3e). These results strongly indicate that self-consistency with respect to the ligand is a much more stringent computational screen for binding than self-consistency with respect to the backbone alone, although backbone-only self-consistency has been used with some success recently^13^.

Although we only explicitly selected for self-consistency and ligand confidence, we observed that NISE also optimized designed sequences according to LASErMPNN confidence (that is, the negative log-likelihood (NLL) decreases; Fig. 1c, left). Thus, by iteratively optimizing reciprocal conditionals, NISE climbs the nearest mode in the joint distribution of *P*(sequence, structure, ligand conformation) (Fig. 1d). This contrasts with an alternative iterative optimization scheme using Rosetta energy minimization to sample new backbone and ligand coordinates, in which the top structures were selected by ligand energy (Fig. 1c, right, and Supplementary Figs. 9 and 10). Iteratively selecting Rosetta-minimized structures by ligand energy and expanding them using sequence design with LASErMPNN did not reduce the NLL of designed sequences nor increase the pLDDT of ligands in predicted co-structures. This implies that these structures are not sufficiently altered to more strongly encode optimal sequences. These results show that both neural networks in NISE are important for optimization.

After pooling and filtering the designs by self-consistency (Supplementary Figs. 11 and 12), we selected four NISE designs (40% mean pairwise sequence identity) with the least number of buried, non-hydrogen-bonded, polar atoms. The NISE algorithm altered exatecan binders from the original input in several ways (Fig. 3d). First, as expected, the sequences were overwhelmingly changed relative to the input design from COMBS and Rosetta, including most binding-site residues (Supplementary Fig. 13 and Supplementary Table 1). Second, a 3_10_ helix segment near the binding site was remodelled into a more ideal coiled coil domain. Third, the gap in a wide helix–helix interface was narrowed to enable better interhelical side-chain packing and increased supercoiling of the bundle. This global structural rearrangement, which reduced the superhelical radius from 7.5 Å to around 7.2 Å, enabled the inclusion of more contacts with the ligand. Fourth, the ligand was translated and rotated by several Å into the bundle interior, increasing its buried hydrophobic surface area. The intended binding mode was maintained, with the labile lactone ring buried in the core of the protein, although some ligand torsional angles became distorted by RFAA. The four designs maintained the same binding mode, yet sampled various degrees of ligand burial, binding-pocket polarity and protein supercoiling, differing from each other and from the input backbone by an average Cα r.m.s.d. of 1.2 Å and 1.3 Å, respectively. The most marked changes in the sequence and structure occurred at early iterations of NISE; subtle refinements in later rounds were important for minimizing the number of buried, non-hydrogen-bonded polar atoms (Supplementary Table 2).

## Characterization of exatecan binders

We ordered synthetic DNA sequences of each of the NISE-designed proteins and expressed the proteins in *Escherichia coli*. Each design was expressed at good yield and was monomeric as assessed by size-exclusion chromatography (SEC; Supplementary Fig. 14). Exatecan is intrinsically fluorescent, so its binding can be tracked in fluorescence polarization experiments using an unlabeled ligand. Fluorescence polarization experiments showed that all four designs bound to exatecan (Fig. 3f and Supplementary Fig. 15), with *K*_d_ values ranging from 0.12 µM to 17 µM (three designs had a *K*_d_ < 10 µM). In comparison, HSA, a non-specific binder of exatecan, had a *K*_d_ of 43 µM. The highest-affinity design, which we named exatecan–protein interaction construct (EPIC), bound to exatecan approximately 360-fold more tightly than HSA. Structurally, the binding site of EPIC contained the fewest polar residues of the four NISE designs, although they all shared a buried Gln51 residue (near the lactone ring) and Asp132, which engaged with the amine group of exatecan. EPIC also buried more of the ligand apolar surface area than did the weakest NISE binder.

We also expressed 16 COMBS-designed proteins. Only three were found to bind to exatecan, with *K*_d_ values of 8 µM, 12 µM and 44 µM (Fig. 3f, Supplementary Fig. 15 and Supplementary Table 1). The design used as the input pose for NISE caused an increase in fluorescence polarization when mixed with exatecan. However, we could not fit the data to a single-site model and protein aggregation was detected by SEC. The three monomeric binders were the top-ranked designs by both self-consistency (ligand r.m.s.d. and backbone Cα r.m.s.d.) and ligand pLDDT (Supplementary Fig. 16). Two additional COMBS–Rosetta designs for the same input pose also bound to exatecan but with weaker affinities (*K*_d_ values of 15 µM and 43 µM; Supplementary Fig. 15), suggesting that the improvements by NISE stem from joint sequence–structure–ligand optimization rather than from pose selection alone.

The substantially higher affinities of NISE designs compared with those of COMBS cannot be attributed to the use of RFAA as a filter for quality, since designs from both methods incorporated self-consistent co-structures using RFAA. Instead, we attribute the tighter binding achieved by NISE to its ability to resculpt the backbone and binding site to make the overall sequence more compatible with both folding and binding (Fig. 3d). Indeed, the binding site of EPIC was almost completely changed compared with the input COMBS model, which contained several residues that formed hydrogenbonds with polar atoms of the ligand (Supplementary Fig. 13). The binding site of EPIC takes a more subtle balance between shape-complementary apolar packing and engagement with ligand polar groups, and only one of the original hydrogen-bonding residues (Asp132) was preserved.

EPIC is highly thermostable (Supplementary Fig. 17) and specific to the camptothecin class of drugs; its affinity is proportional to the steric and chemical similarity of exatecan (Fig. 3g,h and Supplementary Fig. 15). EPIC bound to exatecan at least 50-fold more tightly than did any other ligand tested. Fluorescence polarization experiments showed progressively weaker binding to FL118, belotecan and camptothecin (*K*_d_ values of 6 µM, 19 µM and 90 µM, respectively), with a clear structure–activity relationship for the fluorophenyl ring substituents and amine group of exatecan. Whereas elimination of either functional group was detrimental to binding, desolvation and packing of the fluoro and methyl groups (change in binding free energy, ΔΔ*G* = 3.0 kcal mol^−1^) seemed to drive affinity more than did the interaction with the exposed amine (ΔΔ*G* = 2.3 kcal mol^−1^). EPIC showed no appreciable binding to irinotecan, which contains a bulky prodrug substituent that is sterically incompatible with the designed binding pocket. As expected for a well-sculpted pocket, EPIC does not bind to drugs of other classes, such as anticoagulants (apixaban) and steroids (dexamethasone). Although we did not explicitly bias against off-target ligands during design (that is, negative design), assessment of the EPIC lineage in the NISE trajectory showed a consistently low ligand pLDDT when co-folded with the off-target ligand, camptothecin. By contrast, the ligand pLDDT of exatecan progressively increased in agreement with the experimental results, which showed that EPIC binds to exatecan most tightly (Supplementary Table 2).

## Neural proofreading of the EPIC sequence

**Fig. 4:**
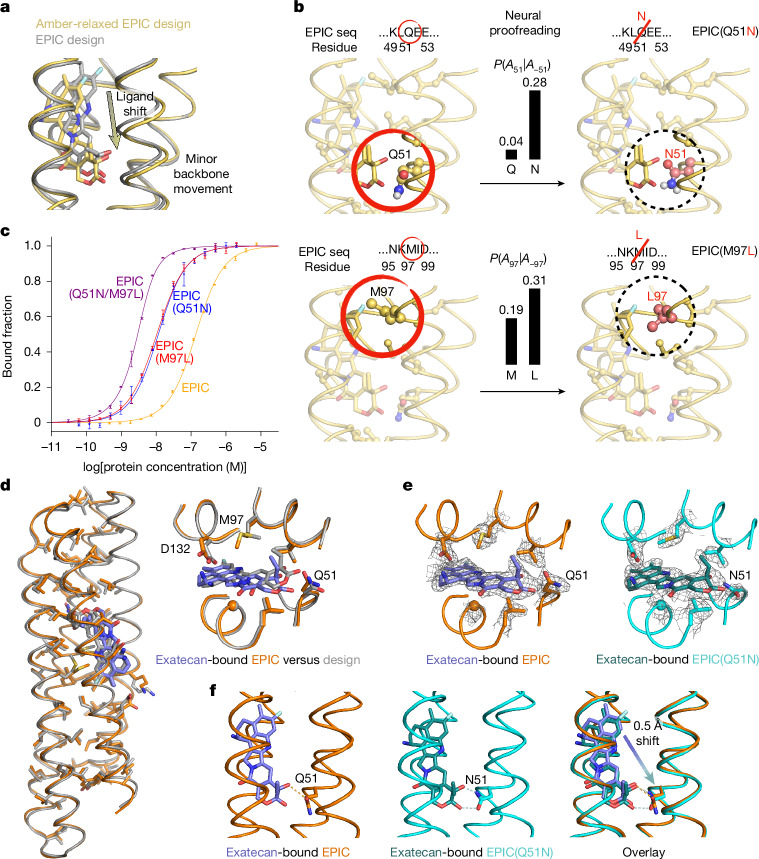
Neural proofreading by LASErMPNN increases the affinity of EPIC for exatecan. **a**, The computational model of EPIC (grey, RFAA prediction) was superimposed onto the same model after coordinate relaxation using the Amber molecular mechanics energy function (yellow). After energy minimization, exatecan moved further into the binding pocket and the backbone around the ligand moved slightly. These new backbone and ligand coordinates were input into LASErMPNN for neural proofreading. **b**, Neural proofreading of EPIC using LASErMPNN to compute the probability of an amino acid (*A*_*i*_) given the entire context of the remainder of the designed sequence (*A*_*−*__*i*_). Examples are shown for neural proofreading of residues 51 (top) and 97 (bottom). **c**, The single amino-acid substitutions have binding affinities that are more than ten-fold higher than that of EPIC, as measured by fluorescence anisotropy of exatecan (converted to the fraction of bound exatecan). Fits are shown as solid lines and [exatecan] = 50 nM. EPIC(Q51N), *K*_d _= 8.0 ± 1.6 nM; EPIC(M97L), *K*_d _= 7.4 ± 0.7 nM; and EPIC(Q51N/M97L), *K*_d _ = 1.2 ± 0.2 nM. Data are mean and s.d. of three technical replicates.* K*_d_ values are mean and s.d. of optimal fits for three biological replicates. The quadratic form of a 1:1 binding model was used for fitting. Global fits are provided in the Supplementary Information. **d**–**f**, The X-ray crystal structure of EPIC is in close agreement with the design model. **d**, Overlay of the EPIC design model (grey) with the X-ray crystal structure of the exatecan–EPIC complex (EPIC in orange and exatecan in blue). The side chains of the core residues are shown. The enlarged binding site shows Asp132, Met97 and Gln51. **e**, The composite omit map for the structure of EPIC with a resolution of 2.0 Å (orange, left; contoured at 1*σ*) shows clear density (grey mesh) for the ligand (blue) and surrounding side chains. Similarly, the composite omit map for the structure of EPIC(Q51N) with a resolution of 2.2 Å (cyan, right; contoured at 1*σ*) shows clear density (grey mesh) for the ligand (green) and side chains, including Asn51. **f**, Side view of EPIC (orange) and EPIC(Q51N) (cyan) highlighting hydrogen-bonding interactions (dashed lines) between exatecan and Gln51 or Asn51. Exatecan moves slightly deeper into the pocket of EPIC(Q51N) to interact with Asn51.

We wanted to determine whether the affinity of EPIC for exatecan could be improved through computation alone. Some strategies use an additional round of backbone diversification starting from the computational structure, followed by ligand docking and several rounds of sequence design to create and test thousands of new sequences^1,6^. Instead, we reasoned that LASErMPNN could refine the EPIC sequence in a more focused approach, noting that even a single amino-acid change could contribute considerably to the binding affinity of the designed protein. We therefore used LASErMPNN to ‘proofread’ the EPIC sequence by suggesting single amino-acid substitutions in the binding site that decrease its NLL (Fig. 4a–c).

**Fig. 5:**
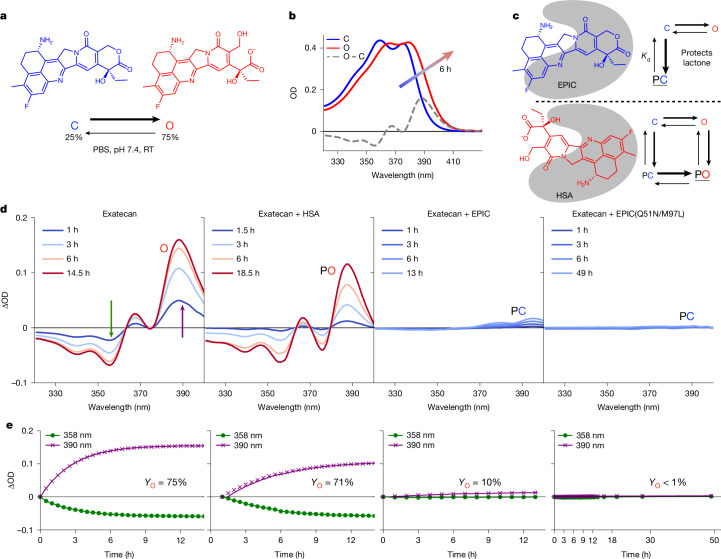
EPIC protects exatecan from hydrolysis. **a**, Equilibrium populations of closed (C) lactone (blue) and open (O) carboxylate (red) forms of exatecan at room temperature (RT) in PBS at pH 7.4. **b**, C versus O states can be monitored spectroscopically by absorbance. The O state (red) has a characteristic red-shifted absorbance and a narrowing of the two prominent peaks. The absorbance difference (ΔOD) for the O–C spectrum is shown as a grey dashed line. The conversion from C to O forms is nearly complete within 6 h (3 half-lives) at room temperature and pH 7.4. **c**, Thermodynamic schemes for EPIC (top) and HSA (bottom). After binding, the lactone ring of exatecan becomes buried in EPIC, protecting it from hydrolysis. By contrast, exatecan binding to HSA does not protect the lactone ring from hydrolysis. P, protein. The dominant equilibrium species is underlined. **d**, Time-resolved absorbance difference spectra for exatecan alone (20 µM) and exatecan with HSA (500 µM), EPIC (20 µM) or EPIC(Q51N/M97L) (20 µM). The concentration of exatecan was 20 µM in all experiments. Experiments were performed at room temperature (295 K) in PBS (pH 7.4) and were initiated using 100% ring-closed exatecan from a DMSO stock. The key for each graph denotes the time delay (*t*; in h). Difference spectra were computed by subtracting the absorbance spectrum at t = 0 h (t = 1 h for HSA owing to baseline shifts at early time points) from absorbance spectra recorded at the stated time delays. Spectra are labeled using the predominant species at the longest time delay. Purple and green arrows are positioned at the corresponding wavelengths shown in **e**, illustrating the direction of spectral evolution. **e**, Kinetic traces at 358 nm and 390 nm for the absorbance difference plots in **d**. For clarity, more data points are shown in the kinetic traces than for the spectra. No significant spectral evolution was observed for EPIC(Q51N/M97L) up to 50 h. The equilibrium yield for O state (*Y*_O_) was computed from a kinetic fit of a two-state mechanism. For HSA, the O state was considered as O + PO (HSA bound to ring-open exatecan). Representative single datasets in **b**, **d** and **e** are shown; results are robust across at least two biological replicates.

There are a few reasons to expect that the same model that designed the EPIC sequence can also offer meaningful suggestions for alterations. During NISE, LASErMPNN designs sequences autoregressively at a high temperature, which means that only the last residue is chosen in the full sequence context. Revisiting each binding-site residue at a lower temperature, with all the other residues fixed, can shift the conditional distribution. We also used RFAA-predicted (and Amber-relaxed)^50^ coordinates of the EPIC sequence as input, providing a subtly different structure that can influence model probabilities (Fig. 5a and Supplementary Fig. 18).

Using an Amber-relaxed, RFAA-predicted model, neural proofreading by LASErMPNN suggested several possible alterations to the EPIC binding site (Fig. 4a,b and Supplementary Fig. 18). We individually characterized the single amino-acid substitutions, EPIC(Q51N) and EPIC(M97L), each of which improved the NLL of EPIC. These alterations were prioritized because the residues were in direct contact with the ligand and represented both hydrophobic packing and polar hydrogen-bonded interactions. Fluorescence polarization experiments showed that each alteration increased the affinity of EPIC for exatecan by more than ten-fold (*K*_d_ values of 8.0 nM and 7.4  nM; ΔΔ*G *≅ −1.6 kcal mol^−1^; Fig. 4c and Supplementary Fig. 19). We combined these sterically compatible alterations in a double-mutant protein, EPIC(Q51N/M97L), and found that the substitutions are energetically additive, resulting in a 100-fold higher affinity than EPIC for exatecan (*K*_d_ = 1.2 nM; ΔΔ*G* = −2.7 kcal mol^−1^). Another mutant, EPIC(G104M), was predicted to decrease affinity (increase NLL for the ligand-bound structure) while stabilizing the unbound state (decrease NLL for the ligand-free structure). As predicted, this mutant showed markedly weakened binding to exatecan (*K*_d_ = 57 µM; Supplementary Fig. 20). SEC experiments showed that each mutant protein remained monomeric (Supplementary Fig. 21). These results demonstrate the predictive power of proofreading for in silico affinity maturation without the use of an experimentally determined structure.

Interestingly, using models such as Boltz-1/2 and AF3, we observed a correlation between measured binding affinity and ligand pLDDT for EPIC and its proofread variants, further motivating the optimization of ligand pLDDT by NISE (Extended Data Figs. 6 and 7).

## Crystal structures of EPIC and EPIC(Q51N)

**Extended Data Fig. 4:**
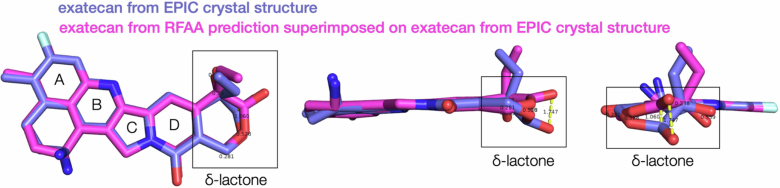
Differences in the predicted and crystallographic conformers of exatecan. Exatecan from the crystal structure (blue) of EPIC was superimposed onto exatecan from the RFAA-predicted structure (magenta) of EPIC via the rigidly linked core of the ligand (A, B, C, and D rings). After superposition, the heavy atoms of the lactone ring had an RMSD of 0.9 Å, with the largest displacement of 1.7 Å occurring between the carbonyl oxygens (yellow dash). The amine of exatecan also showed some discrepancy between the RFAA prediction and the crystal structure.

**Extended Data Fig. 5:**
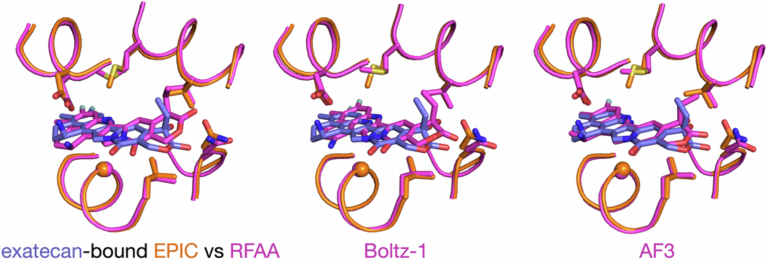
Performance of co-structure predictors on EPIC structure. Comparison of X-ray structure of EPIC (orange protein, blue exatecan) with predicted co-structures (magenta) from RFAA, Boltz-1, and AF3. While all models accurately predict the protein backbone and sidechain conformations, AF3 predicts the co-structure most accurately of the three.

**Extended Data Fig. 6:**
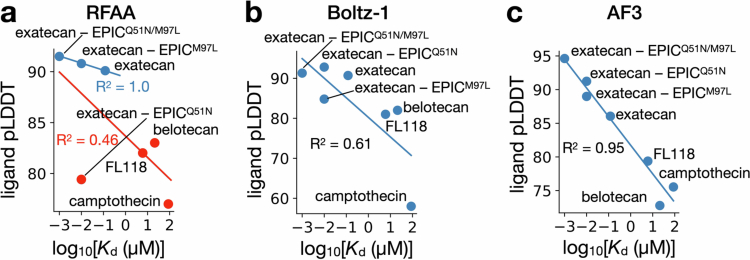
Correlation between predicted ligand confidence and measured binding affinity. Protein–ligand co-structure prediction for various camptothecin-class compounds with EPIC and its mutants, using **a**, RFAA, **b**, Boltz-1, and **c**, AF3. RFAA was run with 100 recycles. Points labeled with a ligand name and without a protein name are co-folded with EPIC protein sequence. For RFAA, points in red were predicted to bind in a different binding mode than exatecan in the crystal structures of EPIC and EPIC^Q51N^. For Boltz-1 and AF3, all ligands were predicted to bind in the same orientation as exatecan (consistent with crystal structures of exatecan-bound EPIC and EPIC^Q51N^). If only including the three points predicted to bind in the observed crystallographic binding mode (blue points), the correlation between affinity and ligand pLDDT is strong (R^2^ = 1) but statistically underpowered. The correlation drops dramatically if including the red points (R^2^ = 0.46). Boltz-1 shows a stronger correlation and AF3 shows the strongest correlation with affinity. Data for non-binders (not shown): EPIC and dexamethasone, ligand pLDDT (70.4, 35.9, 72.7 for RFAA, Boltz-1, and AF3, respectively); EPIC and apixaban, ligand pLDDT (83.4, 52.5, 74.8 for RFAA, Boltz-1, and AF3, respectively).

The structure of EPIC was determined at a resolution of 2.0 Å and showed strong density for bound exatecan (Fig. 4d,e, Extended Data Table 2 and Supplementary Fig. 22). The binding mode of the ligand was as predicted, with expected deviation near the δ-lactone owing to RFAA having struggled to accurately model the correct bond angles for the ligand in this region (Fig. 4d and Extended Data Fig. 4; the aromatic-ring superposition of the originally modelled conformer onto that from RFAA shows a 0.9 Å heavy-atom r.m.s.d. at the δ-lactone). Boltz-1 and AF3 modelled the observed ligand conformer more accurately (Extended Data Fig. 5 and Supplementary Information). Asp132 accepts the intended hydrogen bond from the ligand amine, whereas Gln51 receives a hydrogen bond from the hydroxyl group of exatecan. However, the large side chain of Gln51 is too sterically constrained to donate a hydrogen bond to the carbonyl group of the lactone ring. The backbone agreed closely with the input backbone to LASErMPNN that was used directly upstream in the NISE design cycle (Cα r.m.s.d. 0.8 Å). Rotamers of both core and binding-site residues were accurately predicted by LASErMPNN (Fig. 4d). After superposition on binding-site residues (Cα within 8 Å of ligand heavy atoms, r.m.s.d. 0.4 Å), the ligand coordinates differed from those in the input structure by an average atomic displacement of less than 1 Å and a rotation of 14° around a central axis (Fig. 4d). Thus, the designed structure of EPIC was largely achieved. The structure of EPIC shares no similarity with that of topoisomerase I, including at the drug-binding site, showing that the NISE design algorithm can generate highly original de novo binders (Extended Data Fig. 3).

The structure of exatecan-bound EPIC(Q51N) (2.2 Å resolution) provided insight into the higher affinity of this complex compared with that of EPIC (Fig. 4e,f). The shorter side chain of Asn51 in EPIC(Q51N) draws exatecan slightly further into the pocket (by approximately 0.5 Å), and its carboxamide group engages in a bidentate hydrogen-bonding interaction with the hydroxyl group and lactone carbonyl of exatecan (Fig. 4f). As a result, the ligand is buried more deeply and further desolvated .

## EPIC protects exatecan from hydrolysis

The lactone ring of exatecan hydrolyses rapidly under physiological conditions (Fig. 5a,b), decreasing to a 15% steady-state level in human plasma^41^ within 10 h. The high concentration of HSA in plasma drives HSA binding to exatecan but does not prevent hydrolysis (Fig. 5c–e), consistent with a camptothecin-bound structure (PDB: 4L9K) in which the ring-open form is stabilized by nearby Arg residues (Supplementary Fig. 23). EPIC behaves oppositely: by sequestering the labile lactone ring from the solvent, EPIC and its mutants markedly repartition the equilibrium towards the intact bioactive form of the drug (Fig. 5d,e). Time-resolved absorption experiments using EPIC(Q51N/M97L) showed that more than 99% of exatecan stayed in the ring-closed form in PBS (pH 7.4) at room temperature for at least 50 h; there was no major hydrolysis product detected during this time. EPIC(Q51N/M97L) also protected exatecan in the presence of physiological concentrations (500 µM) of HSA (Supplementary Figs. 24 and 25). Thus, EPIC and its variants maintain a reservoir of the bioactive form of the drug, which has potential applications in drug delivery and targeting.

## Design of apixaban binders using NISE

**Fig. 6:**
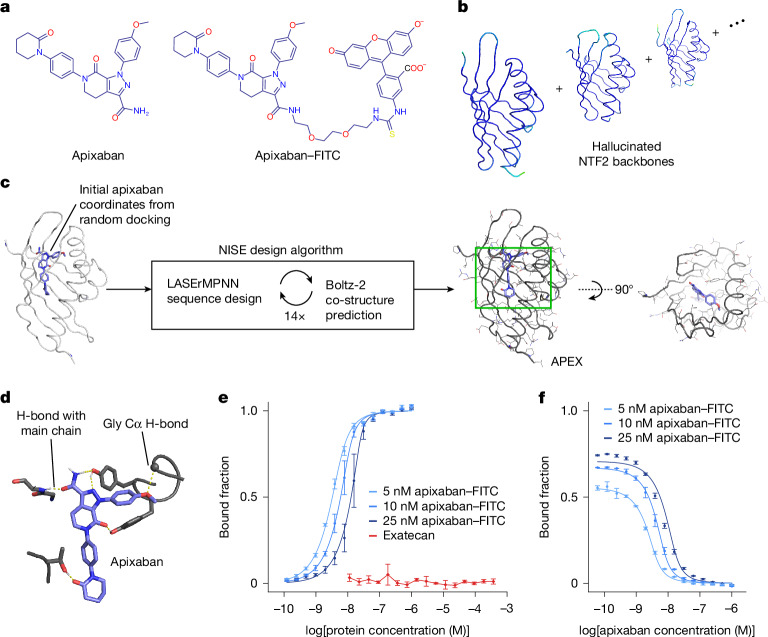
Design of high-affinity apixaban-binding proteins using NISE and NTF2 folds. **a**, Chemical structure of apixaban and a FITC-appended derivative. **b**, An ensemble of 50 computationally generated NTF2 folds was used (coloured by Boltz-2 Cα pLDDT; blue > 95). **c**, Apixaban (blue) was placed in these NTF2 folds using rigid-body docking (Supplementary Information). NISE trajectories (14–28 iterations each) were then run for a few docked poses using Boltz-2 instead of RFAA. Six diverse NISE designs were chosen for experimental testing. The area indicated by the green box is shown in **d**. The design model is shown (in grey) for the highest-affinity protein, APEX. **d**, Close-up of the Boltz-2 predicted structure of APEX. APEX forms several hydrogen bonds with apixaban using both main-chain and side-chain interactions. A Cα hydrogen bond is also formed through a Gly residue. **e**, Fluorescence anisotropy experiments show tight, specific binding of APEX to apixaban–FITC (converted to the fraction of bound apixaban–FITC). **f**, Fluorescence anisotropy competition experiments using a constant amount of apixaban–FITC and APEX protein, incubated with varying amounts of apixaban. Data for three concentrations were fitted globally to determine the *K*_d_ of APEX with apixaban–FITC (**e**; *K*_d_ = 990 pM, 95% confidence interval 720–1,280 pM) and apixaban (**f**; *K*_d_ = 80 pM, 95% confidence interval 54–122 pM). Fits are shown as solid lines. In **e**, APEX shows no appreciable binding to exatecan (red; [exatecan] = 50 nM; the connecting line is not a fit and anisotropy values were converted to the bound fraction on the basis of maximum and minimum values from apixaban–FITC). Data in **e** and **f** are mean ± s.d. of three technical replicates. *K*_d_ values are the mean of 1,000 fits performed by bootstrapping of the optimal-fit residuals, and bounds are 95% confidence intervals derived from bootstrapped fits.

To test the robustness of the NISE algorithm, we applied it to a different protein fold and small-molecule target. As scaffolds, we chose the mixed α/β NTF2 fold. Like helical bundles, NTF2 backbones have previously been used in small-molecule binder design^6,27,51^ and contain a variable-sized pocket. As the target, we picked the factor Xa inhibitor, apixaban, a clinically approved anticoagulant^52,53^ (Fig. 6a). There remains a clinical need for an inexpensive antidote to apixaban that avoids the procoagulant effects observed with the recently withdrawn antidote, andexanet alfa^53^, a catalytically inactive variant of factor Xa.

Although apixaban was a previous target for the COMBS design algorithm, which used four-helix bundles^4^ (with a lowest *K*_d_ of 600 nM, two binders out of six tested designs), the fold and target were chosen, in part, because NTF2 scaffolds have been used more recently with LigandMPNN and Rosetta to design a binder to apixaban^6^. In that study, three cycles of LigandMPNN-based design and Rosetta minimization on thousands of apixaban-docked NTF2 backbones yielded four binders out of 9,024 tested designs (best *K*_d_ = 680 nM). The computational algorithm differs notably from NISE in that designs were not iteratively optimized to improve neural-network-based confidence metrics: no selection–expansion of the best candidates was performed during the design trajectory.

We set out to design an apixaban binder using NISE and a small, Boltz-2-predicted subset of 50 of the same NTF2 backbones^6,51^ used previously for this target (Fig. 6b). Using the same backbones and small-molecule target enabled a head-to-head comparison of the methods. Our design process used LASErMPNN for sequence design and replaced RFAA with Boltz-2 for co-structure prediction. We found that using Boltz reduced the number of sequences needed per round of NISE to pass self-consistency thresholds while maximizing design metrics such as ligand pLDDT (Supplementary Figs. 26 and 27).

Instead of using COMBS, we docked two crystallographic conformers of apixaban (from PDB: 6W70; Supplementary Fig. 28) into the NTF2 backbones using random rotations and translations (Supplementary Information). We filtered candidate ligand positions by depth of burial in the protein and steric clashing with the protein backbone. We clustered the remaining ligands by their docked orientations, then used LASErMPNN to design three sequences each and Boltz-2 to predict co-structures. We filtered these designs by self-consistency (protein Cα and ligand heavy-atom r.m.s.d. < 2.0 Å).

For the remaining co-structures, we performed an additional round of sequence design (five sequences each) and co-structure prediction, then ranked the remaining poses using a linear combination of ligand pLDDT and binary affinity probability (P(bind)) in Boltz-2. Unlike ligand pLDDT, P(bind) was explicitly trained to differentiate between binders and non-binders. Because this closely aligns with our design task, and because P(bind) is not trivially correlated with ligand pLDDT (Extended Data Fig. 8), we linearly combined P(bind) with ligand pLDDT to create a composite score for ranking designs. We found that additional selection by P(bind) further biased designs with a high ligand pLDDT towards a higher shape complementarity (Supplementary Fig. 29).

For a few top-ranked poses, we ran NISE trajectories to optimize the score (Fig. 6c), resulting in changes to the protein structure, sequence and ligand conformation (Supplementary Fig. 30). After pooling, filtering and ranking designs from the NISE trajectories (Supplementary Figs. 31 and 32 and Supplementary Information), we ultimately chose six protein sequences for experimental analyses (four distinct poses, 29% mean pairwise sequence identity).

We expressed the chosen proteins in *E. coli* and purified them. All proteins were monomeric as assessed by SEC (Supplementary Fig. 33), and five of the six designs bound tightly to apixaban in competitive binding experiments (*K*_d_ < 50 nM, Fig. 6e,f and Extended Data Fig. 9). The highest affinity binder, apixaban-binding protein exemplar (APEX; Fig. 6c,d), bound the drug extremely tightly (*K*_d_ = 80 pM; 95% confidence interval of 54–122 pM; Fig. 6e,f and Supplementary Fig. 34), rivaling the native target of apixaban^52^, factor Xa (inhibition constant (*K*_i_) = 80–700 pM), at one-third the size (13 kDa compared with 43 kDa). Binding of apixaban was specific: APEX showed no detectable signs of binding exatecan (Fig. 6e).

**Extended Data Fig. 7:**
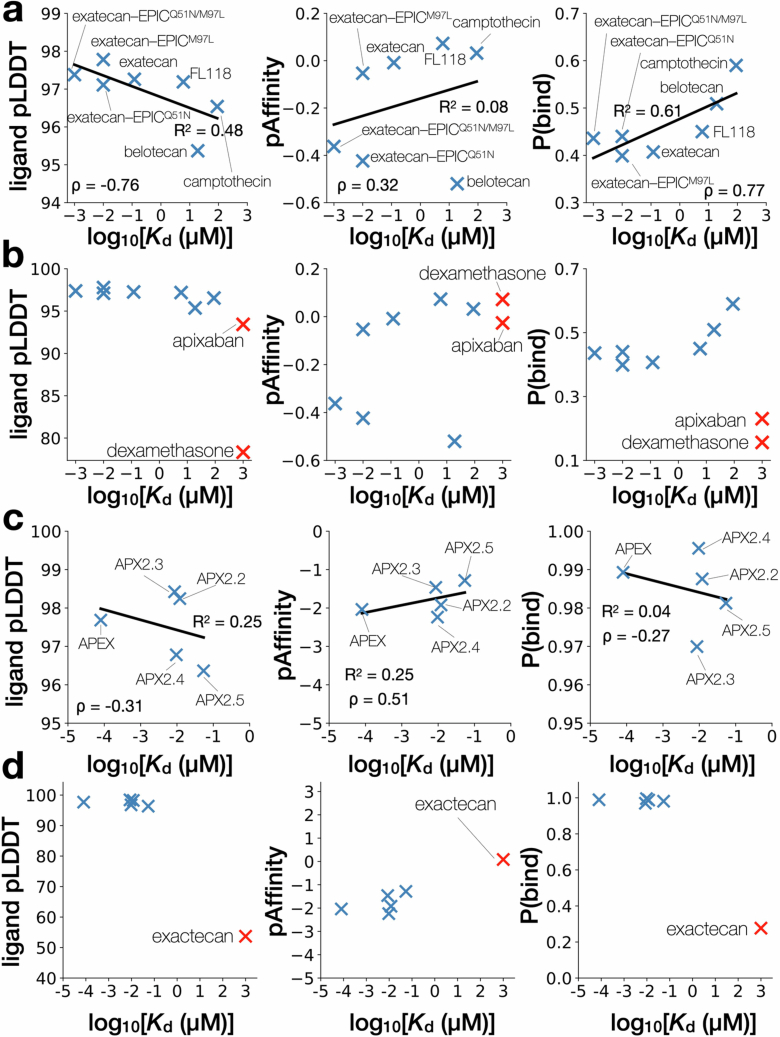
Correlation between predicted metrics from Boltz-2 and binding affinity of NISE-designed proteins. **a**, Protein–ligand co-structure prediction by Boltz-2 for various camptothecin-class compounds with EPIC and its mutants. Points labeled with a ligand name and without a protein name are co-folded with EPIC protein sequence. pAffinity is Boltz-2’s affinity_pred_value, which is the predicted log_10_[IC_50_(µM)], and P(bind) is Boltz-2’s affinity_probability_binary. Ligand pLDDT shows a weak positive correlation with experimentally determined binding affinity, pAffnity shows an even weaker correlation, and P(bind) shows an inverse correlation between binding affinity. ρ is the spearman rank correlation, R^2^ is correlation from least squares fitting (black line). **b**, Same data as in **a** but with additional results from co-structure predictions with apixaban and dexamethasone (red). Note that apixaban-peg-FITC and dexamethasone-peg-FITC did not show any experimentally detectable binding, so marked affinities of 1000 µM here are only for illustrative purposes. **c**, **d**, Same analysis as in **a**, **b** but for the 6 NISE-designed apixaban binders. These results show that P(bind) is a suitable classifier for predicting binding *vs* non-binding (its training domain) but less so for predicting relative affinity between similar ligands.

Analysis of the five verified binders showed a weak correlation between the ligand pLDDT from Boltz-2 and the measured binding affinity, whereas P(bind) discerned between on-target and off-target ligands but did not correlate with relative affinity (Extended Data Fig. 7c,d). We therefore asked whether these metrics capture binding specificity across the APEX design lineage. An assessment of the NISE trajectory leading to APEX showed a consistently low ligand pLDDT and P(bind) when co-folded with the off-target ligand, exatecan. It also showed an increasingly high ligand pLDDT and P(bind) when co-folded with the intended target, apixaban, in agreement with experimental results (Supplementary Table 3). The binding pocket of APEX is predicted to form a mixture of both main-chain and side-chain hydrogen-bonded interactions with apixaban (Fig. 6d). This is distinct from both factor Xa and a previously designed apixaban-binding helical bundle^4^ (Supplementary Fig. 35), highlighting the novelty of the binding solution found using our approach.

## Discussion

Tight coupling between two generative neural networks unlocks the zero-shot design of small-molecule binders, with one network used to sample broadly in sequence space and the other to model coupled changes in protein and ligand coordinates. As a result, NISE can accomplish design tasks that other methods struggled with. First, we used NISE to design helical bundles that bind to exatecan. All four experimentally tested designs bound the drug, contrasting with the state-of-the-art method, COMBS; the tightest COMBS binder was nearly 70-fold weaker. The tightest binder designed by NISE, EPIC, was further improved through neural proofreading and shown to protect the labile drug from hydrolysis for several days. To test NISE on an equal footing with an alternative algorithm using LigandMPNN and Rosetta^1,6^, we next tackled an identical design challenge: creating binders to apixaban using NTF2 backbones. Here, NISE was implemented with LASErMPNN and Boltz-2, and achieved a success rate (83%) that was more than three orders of magnitude higher than the alternative approach. This result is striking because we used the same previously published backbones as starting points for design. Moreover, tight binding was achieved by NISE using vastly different sequences and binding poses (Extended Data Fig. 9 and Supplementary Table 4). Notably, specialized docking methods were not needed: brute-force rigid-body docking was sufficient to seed NISE. The success rate alone does not fully capture the scale of the advance, as the highest affinity binder from NISE, APEX (*K*_d_ = 80 pM), bound to apixaban nearly 10,000-fold more tightly than did the best binder from LigandMPNN^6^ or COMBS^4^, rivalling even the native drug target^52^, factor Xa.

NISE differs from previous protocols in a few important ways. First, it allocates most of its compute to extensively sample a few designs. We first perform a broad search to nominate initial self-consistent designs, then NISE executes a deep search to optimize them. Second, NISE uses only neural networks for closed-loop optimization. We investigated the effects of replacing the co-structure predictor with a traditional approach to modelling structure using Rosetta (Fig. 1c, right). In this case, the designs failed to optimize, suggesting that current empirical energy functions cannot fully capture the nuances of productive protein–ligand interactions. Geometrically, the gradient of the Rosetta energy lies partly orthogonal to that of *P*(structure, sequence, ligand conformation), so following it does not efficiently climb the joint distribution. Further iterations of any Rosetta-based loop would likely not overcome this limitation (Fig. 1c and Supplementary Figs. 9 and 10). By contrast, NISE samples directly from the learned distributions of two reciprocal neural networks, iteratively climbing to a higher probability mode in the joint probability manifold, making the designs look more similar to the training data (structures in the PDB).

NISE is agnostic to the specific networks used; as these models improve, so will NISE. Indeed, a RFAA-based assessment would have led us to discard our apixaban binders (Supplementary Fig. 36); the use of Boltz-2 was important to select binders for experimental testing. LigandMPNN could be substituted for LASErMPNN to produce similar aggregate metrics (Supplementary Figs. 37 and 38), although we observed that LigandMPNN tends to overpack designs (Extended Data Fig. 1 and Supplementary Figs. 39 and 40). Differences in packing might stem from differences in the sampling algorithm: whereas LigandMPNN predicts rotamers only after the entire sequence is designed, LASErMPNN jointly determines the sequence and rotamers.

**Extended Data Fig. 8:**
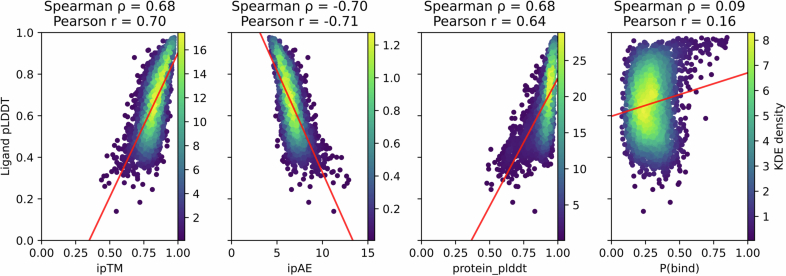
Ligand pLDDT is correlated with other confidence metrics but not Boltz-2’s P(bind). Ligand pLDDT is highly correlated with ipTM, ipAE, and protein Cα pLDDT (protein_plddt). It is weakly correlated with affinity_probability_binary, which we call P(bind), the predicted probability of binding generated by the ligand affinity prediction module. 3,000 datapoints are shown in each plot from a random subsample of 30,000 LASErMPNN sequences generated from 10,000 CARPdock poses of apixaban docked into our NTF2 scaffold library. We re-predicted co-structures with Boltz-2’s affinity module to extract P(bind).

**Extended Data Fig. 9:**
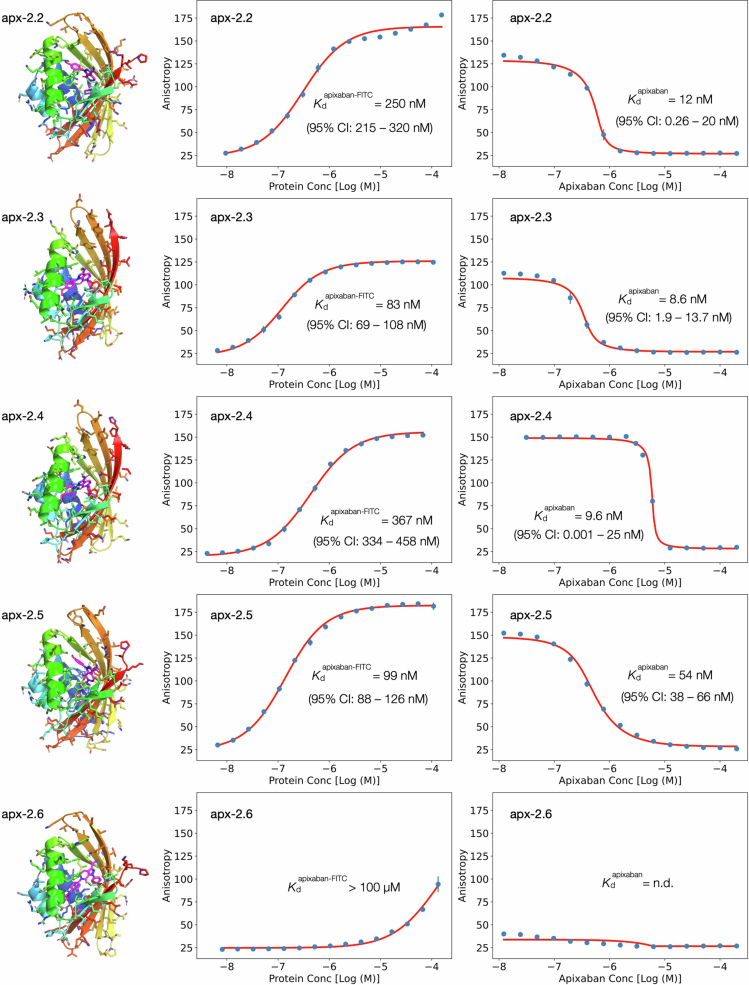
NISE designs bind apixaban with high affinity. **Left**, Protein–ligand co-structures predicted by Boltz-2 are shown for each NISE design. **Middle**, Binding curves monitoring fluorescence polarization of apixaban-FITC (50 nM) as a function of protein concentration. **Right**, Competition binding experiments monitoring fluorescence polarization of apixaban-FITC (50 nM) for constant protein concentration and varying apixaban concentration. Data (blue circles) is fit globally (red lines) for each row (i.e. each design) separately. The dissociation constants for apixaban-FITC and apixaban are fit simultaneously to a 1-to-1 binding model, sharing maximum anisotropy values. Minimum anisotropy was allowed to float locally but values did not differ significantly. Confidence intervals (95%) of the fit dissociation constants are shown in parentheses, derived from bootstrapped distributions of the fits for 500 trials. Blue points are the mean of three replicates with error bars representing standard deviation.

We focused on optimizing ligand pLDDT during NISE trajectories. This parameter is highly correlated with other metrics, such as protein Cα pLDDT and interfacial predicted aligned error (iPAE), which are commonly used for protein-binder design^15,23^ (Extended Data Fig. 8 and Supplementary Fig. 41). Adding P(bind) from Boltz-2 increased shape complementarity moderately. Maximizing model confidence and agreement was necessary but perhaps not sufficient by themselves. We found that performing NISE iterations beyond the plateau in ligand pLDDT or score was important for downstream filtering by additional biophysical metrics. Indeed, both EPIC and APEX came from later iterations. Using LASErMPNN and Boltz-2, a typical NISE trajectory of 14 iterations only takes around 5 h on four A6000 graphics processing units (with the majority of compute dedicated to co-structure prediction).

We found that positive design—focusing on moulding the binding pocket only to the target ligand—was sufficient to install a high degree of binding specificity. We showed that both EPIC and APEX were specific: they had the highest affinity for their target ligands and did not bind to dissimilar off-target ligands (Figs. 3g and 6e). For similar on- and off-target molecules, an even wider gap in affinities could be produced explicitly using NISE by selecting representatives from each cycle that maximize both on-target ligand pLDDT (positive design) and the gap in ligand pLDDT (or predicted affinity) of on- and off-target ligands (negative design).

In this work, we initialised designs from two precomputed sets of designable backbone scaffolds, helical bundles and NTF2 folds. These scaffolds have pockets that can fit a variety of ligands for future design targets. As generative backbone models improve (such as RFdiffusion-All Atom^8^, BoltzDesign1 (ref. ^54^) and BoltzGen^55^), we could also use bespoke backbones conditioned on target ligands as inputs to NISE. Currently, we find that using a small, precomputed set of highly designable scaffolds is a computationally efficient approach to achieving high-quality poses. Using structures that can be encoded by many sequences—such as helical bundles and NTF2 folds—enables the efficient sampling of sequences that support both folding and binding. Success with four-helix bundles and mixed α/β NTF2 folds shows that NISE is a general algorithm that does not rely on any specific protein fold, and we expect NISE to work with any fold that can be modelled accurately by co-structure predictors.

The design of small-molecule binding proteins is now approaching the hit rates of the design of PCR primers, yet the rules of protein–ligand molecular recognition are vastly more complex than Watson–Crick base pairing. We have shown that modern deep neural networks, coupled with appropriate training data, can capture and distil this complexity, which can ultimately be extracted using design algorithms such as NISE. We anticipate that these capabilities will catalyse the use of bespoke proteins as rapidly generated reagents to manipulate biology at the single-molecule level.

## Methods

See Supplementary Information.

### Reporting summary

Further information on research design is available in the Nature Portfolio Reporting Summary linked to this article.

## Online content

Any methods, additional references, Nature Portfolio reporting summaries, source data, extended data, supplementary information, acknowledgements, peer review information; details of author contributions and competing interests; and statements of data and code availability are available at 10.1038/s41586-026-10670-w.

## Supplementary information

Supplementary Information Supplementary Notes, Supplementary Figs. 1–50, Supplementary Tables 1–9 and Supplementary References.

Reporting Summary

Peer Review File

## Figures and Tables

**Extended Data Table 1:**
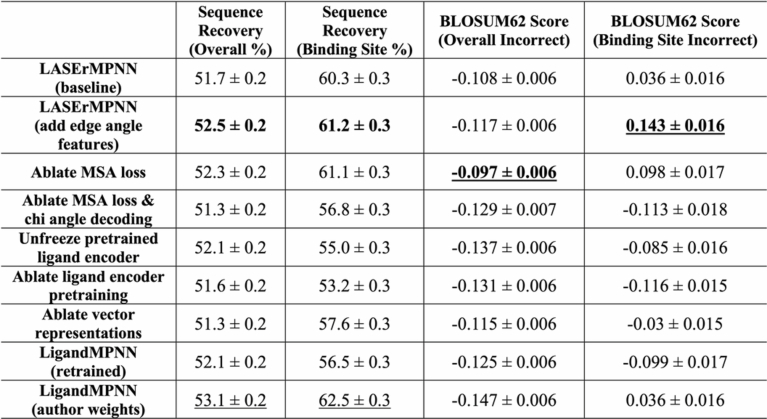
LASErMPNN Ablation Studies*

*The best values achieved over our reimplementation of the LigandMPNN test set are highlighted in bold, and the best values across all models are underlined. Note that the author weights released with the published version of LigandMPNN might have been trained on the entire PDB, potentially leaking its training data into this test set. (The paper and GitHub repository are unclear on this.). So, we retrained the LigandMPNN model on the same dataset as LASErMPNN for a fair comparison between model architectures. All values are means and standard deviations computed by bootstrapping (N = 100,000) the value of the mean sequence recovery or BLOSUM62 score uniformly over the 5 sequences generated for each of the 317 members of the test set. Data for each ablation was generated by a single model. Variance due to randomness in training initializations and optimization is not quantified (due to compute expense).

**Extended Data Table 2:**
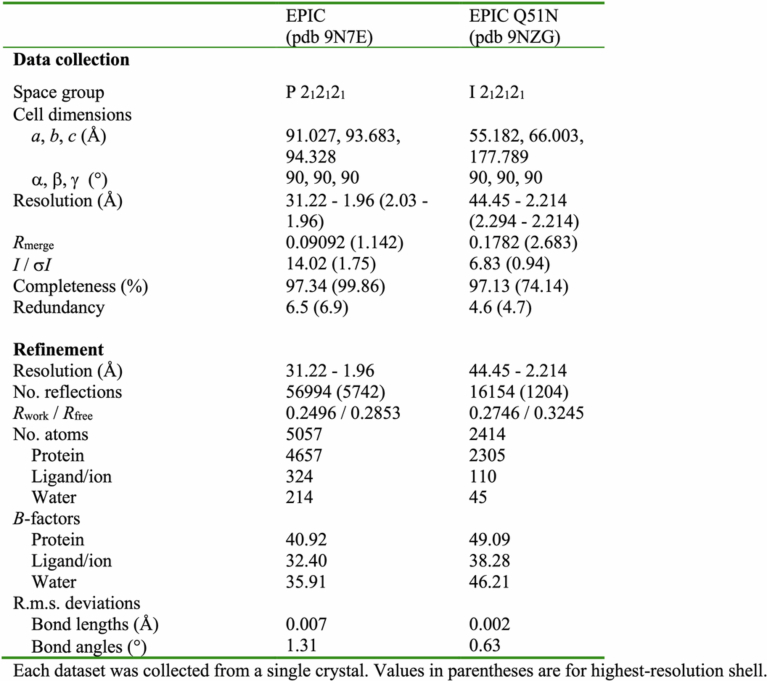
Data collection and refinement statistics (molecular replacement)

## Data Availability

Coordinates and data files for the X-ray crystal structures of exatecan-bound EPIC and exatecan-bound EPIC(Q51N) have been deposited in the PDB with accession codes 9NZE (EPIC) and 9NZG (EPIC(Q51N)). Other referenced PDB IDs include 6W70, 4JNJ, 4L9K and 8TN6. The X-ray crystal structure of the camptothecin derivative used to build exatecan conformers was retrieved from Cambridge Structure Database (CSD) deposition number 1504071. All data used to train LASErMPNN are publicly available from the PDB or the SPICE v.2.0.1 dataset^56^. The processed datasets used to train the LASErMPNN model are available at Zenodo^57^.

## Code availability

All model inference code, training code, processed datasets and dataset split information can be found at GitHub (https://github.com/polizzilab/LASErMPNN). A Google Colab notebook implementing LASErMPNN sequence design and proofreading can be found at https://colab.research.google.com/github/polizzilab/LASErMPNN/blob/main/run_lasermpnn.ipynb. An implementation of the NISE protocol using Boltz-1/2 can be found at GitHub (https://github.com/polizzilab/NISE). A minimal implementation of NISE using LASErMPNN and Boltz-2 can be found at https://colab.research.google.com/github/polizzilab/NISE/blob/main/NISE_LASErMPNN.ipynb. An implementation of the rigid ligand–protein docking protocol used to generate NISE inputs for apixaban binders can be found at GitHub (https://github.com/benf549/CARPdock). Analysis code and PDB files of designs can be found at Zenodo^58^.
