# Supplementary material for: Zero-shot design of drug-binding proteins via neural iterative selection−expansion
Source: Nature. 2026 Jun 24;656(8126):237–49. doi: 10.1038/s41586-026-10670-w (PMC13441969; doi:10.1038/s41586-026-10670-w)
Supplement: Supplementary file 1 [file 41586_2026_10670_MOESM1_ESM.pdf]

nature portfolio

https://doi.org/10.1038/s41586-026-10670-w

Supplementary information

Zero-shot design of drug-binding proteins via neural iterative selection–expansion

In the format provided by the authors and unedited

Supplementary Information

Zero-shot design of drug-binding proteins via neural iterative selection−expansion

Benjamin Fry[1,2,3,4], Kaia Slaw[2,3,4], Nicholas F. Polizzi[2,3]*

[1]Harvard Graduate Program in Biophysics, Harvard University, Boston, MA, 02215, USA.
[2]Department of Cancer Biology, Dana-Farber Cancer Institute, Boston, MA 02215, USA.
[3]Department of Biological Chemistry and Molecular Pharmacology, Harvard Medical School, Boston, MA 02215, USA.
[4]These authors contributed equally.

Corresponding author email: NicholasF_Polizzi@DFCI.harvard.edu

Table of Contents

Supplementary Text **5**
LASErMPNN Overview 5
Heterograph Construction 5
Ligand Encoder Pretraining 5
LASErMPNN Inputs and Outputs 6
LASErMPNN HeteroGATv2 Message Passing 6
LASErMPNN Loss Terms 7
Training & Inference Differences 8
Protein Residue Frame Noising 8
Training LASErMPNN 9
van Der Mer (vdM)-Inspired Pseudoligand Sampling 9
Protein Data Bank Dataset Curation 9
LigandMPNN Dataset Split Reconstruction 10
Streptavidin Hold-out Dataset Split Construction 10
NISE Protocol Implementation for Exatecan Binders 11
Design Filtering and Ranking 11
Benchmarking LASErMPNN with Streptavidin–Biotin complex 12
Testing ranking protocol of LASErMPNN designs using rucaparib-PiB complex 13
Details of ablation studies 13
Neural proofreading protocol 14
Training noise levels and foldability optimization 15
AlphaFold3 Self-Consistency Test Set Evaluation 15
Computation of Buried, Non-H-bonded Atoms 16
Rotamer Analysis 16
Analysis of NISE Design Trajectory 16
Rosetta Iterative Selection–Expansion Trajectory 16
Correlation of Ligand pLDDT and Binding Affinity using AF3, Boltz-1, and RFAA 17
Creation of Exatecan Conformers 17
Small Molecules 18
Family-wide Hallucination of Four-helix Bundles 18
Fluorescence Polarization Experiments to Determine Binding Affinities 19
Fits to Fluorescence Anisotropy Data 19
Cloning, Protein Expression, and Protein Purification 20
Size-exclusion Chromatography 21
Circular Dichroism to Determine Secondary Structure and Stability 21
Hydrolysis Experiments 21
Binding to HSA is Compatible with Hydrolysis 21
Analysis of Exatecan Hydrolysis Data 22
Design of Exatecan Binders via COMBS and Rosetta 22
Initial COMBS Model for NISE design 23
Comparison of Input COMBS Model vs EPIC Design 23
Selection of Additional Pair of COMBS Designs for Experimental Characterization 24
Ranking of COMBS Designs by Metrics from Protein–ligand Co-structure Prediction 24
Ranking of Designable Poses by Metrics from Protein–ligand Co-structure prediction 24
X-ray Crystal Structures 24
Comparison of X-ray Crystal Structure and Results from Co-structure Predictors 25
Creation of NTF2 Scaffold Library 25
Design of Apixaban-Binding Proteins 26
Implementation of Rigid Protein-Ligand Docking Protocol (CARPdock) 28
Correlation of Ligand pLDDT with other Confidence Metrics from Boltz-2 29
LigandMPNN Evaluation on Streptavidin Holdout Split 29
Comparing LASErMPNN and LigandMPNN within NISE 30
Re-screening CARPdock Poses with LigandMPNN 30
Comparing LASErMPNN and LigandMPNN Designs using Rosetta Energy 31
Interpretation of NISE Design Success 32
EPIC and APEX NISE Trajectory Lineage Analysis 34
Ablations of Filtering Metrics 35
Prediction of Apixaban-Binding Proteins with RFAA 35
Statistical Bootstrapping NISE Confidence Metrics for Uncertainty Quantification 35

Evaluating H-Bond Networks for Calculation of Buried, Non-H-bonded Polar Atoms 35
NTF2 ligand-binding experiments 35
Fluorescence polarization competition experiments for NTF2 designs 36
Size-Exclusion Chromatography of NTF2 designs 36
Runtime Quantification 36
Code availability 37
NISE pseudocode 37
Energy minimization code for Rosetta iterative selection-expansion trajectory 37
Rosetta code for fixed backbone sidechain minimization (for BUNs calculation) 38
Rosetta apo structure repacking code 39
Rosetta ligand params file generation code 39
AMBER energy minimization code 39
MMseqs2 sequence-clustering code 40

Supplementary Figures 42
Supplementary Fig. 1. Block diagram of information flow through the LASErMPNN architecture 42
Supplementary Fig. 2. Using AF2 foldability to identify optimal amount of training noise 43
Supplementary Fig. 3. Distributions of various RMSD metrics for LASErMPNN-designed sequences of streptavidin-biotin 44
Supplementary Fig. 4. Co-structure predictions of sequences designed by LASErMPNN more closely resemble the monomeric streptavidin structure than those from LigandMPNN 45
Supplementary Fig. 5. Binding-site sequence recovery is inversely correlated with the number of buried, non-H-bonded, polar atoms 46
Supplementary Fig. 6. Filtering and ranking protocol of LASErMPNN-designed sequences ranks PiB favorably 47
Supplementary Fig. 7. Building conformers of exatecan 48
Supplementary Fig. 8. NISE improves the self-consistency of the predicted ligand and the negative log likelihood (NLL) of designed sequences 49
Supplementary Fig. 9. Energy-based selection–expansion is unable to optimize sequence NLL or ligand pLDDT 50
Supplementary Fig. 10. An energy-based iterative selection–expansion protocol using Rosetta does not optimize neural network confidence 51
Supplementary Fig. 11. NISE filtering and ranking protocol for selecting exatecan-binding proteins for experimental characterization 52
Supplementary Fig. 12. NISE filtering and ranking protocol for selection of exatecan binders 53
Supplementary Fig. 13. Comparison of traditional design model (COMBS and Rosetta) with output from NISE (EPIC model) 54
Supplementary Fig. 14. Size exclusion chromatograms of various exatecan-binder designs 55
Supplementary Fig. 15. Fluorescence anisotropy data and fits to a single-site binding model 56
Supplementary Fig. 16. Ligand confidence and self-consistency are correlated with experimental outcomes of binding 57
Supplementary Fig. 17. Determination of secondary structure content and thermal stability of EPIC by circular dichroism (CD) spectroscopy 58
Supplementary Fig. 18. Heatmap of probabilities after proofreading of binding-site residues 58
Supplementary Fig. 19. Global binding fits of EPIC mutants to exatecan 59
Supplementary Fig. 20. Fluorescence anisotropy data and fit to a single-site binding model for EPIC G104M with exatecan 59
Supplementary Fig. 21. Size exclusion chromatograms of EPIC and its mutants show that each is monomeric 60
Supplementary Fig. 22. Asymmetric units of x-ray crystals of exatecan-bound EPIC and EPIC Q51N 60
Supplementary Fig. 23. Camptothecin-bound structure of human serum albumin 61
Supplementary Fig. 24. Time-resolved absorbance spectra of exatecan alone or with various proteins 62
Supplementary Fig. 25. Fits of time-resolved hydrolysis experiments 63
Supplementary Fig. 26. NISE jointly optimizes sequence and structure confidence using either RFAA or Boltz-1 64
Supplementary Fig. 27. Boltz-1 outperforms RFAA at predicting exatecan bound in the designed orientation 65
Supplementary Fig. 28. Apixaban conformers from 6w70 used in NISE design of apixaban binders 66
Supplementary Fig. 29. Boltz-2 P(bind) captures information about designed binding site quality 67
Supplementary Fig. 30. NISE-derived changes for APEX design 68
Supplementary Fig. 31. Flowchart depicting the design workflow that generated APEX 69
Supplementary Fig. 32. Filtering and ranking protocol for selection of apixaban binders from pooled NISE trajectories 70
Supplementary Fig. 33. Size exclusion chromatograms of purified NTF2 designs 71
Supplementary Fig. 34. APEX binds apixaban tightly and specifically 72
Supplementary Fig. 35. Binding sites of apixaban in natural and de novo proteins are dissimilar 73
Supplementary Fig. 36. Post-hoc RFAA predictions of experimentally verified apixaban binders 74
Supplementary Fig. 37. NISE optimizes Boltz-2 Score and LASErMPNN NLL for apixaban–NTF2 designs 75

Supplementary Fig. 38. NISE optimizes Boltz-2 Score and LigandMPNN NLL for apixaban–NTF2 designs 75
Supplementary Fig. 39. LigandMPNN designs larger residues in the binding pockets of apixaban–NTF2 designs................76
Supplementary Fig. 40. LigandMPNN generates overall sequences that are more overpacked than those from LASErMPNN 77
Supplementary Fig. 41. Ligand pLDDT and protein Cα pLDDT are positively correlated...78
Supplementary Fig. 42. Amino acids are used as "pseudoligands" to augment ligand training data for LASErMPNN 78
Supplementary Fig. 43. Protein nodes store equivariant vector representations..79
Supplementary Fig. 44. LASErMPNN and LigandMPNN models perform similarly by self-consistency RMSDs on a small test set 80
Supplementary Fig. 45. LASErMPNN and LigandMPNN models generate sequences with similar AlphaFold3 ligand pLDDT distributions on a small test set 81
Supplementary Fig. 46. LASErMPNN and LigandMPNN models generate sequences with similar sequence recoveries over a small test set...82
Supplementary Fig. 47. LASErMPNN generates sidechain conformations with low RMSD to crystallographic conformations 83
Supplementary Fig. 48. Neural networks for protein–ligand co-structure prediction are more efficient than Rosetta at optimizing LASErMPNN confidence...84
Supplementary Fig. 49. LASErMPNN and structure prediction can be used to rank poses that will lead to binders 85
Supplementary Fig. 50. Screening CARPdock poses with LigandMPNN nominates similar poses as with LASErMPNN....86

Supplementary Tables.. 87
Supplementary Table 1. Characterized exatecan-binding protein sequences..87
Supplementary Table 2. RFAA metrics for the EPIC lineage from the NISE trajectory 88
Supplementary Table 3. Boltz-2 metrics for the APEX lineage from the NISE trajectory..89
Supplementary Table 4. Characterized apixaban-binding protein sequences 90
Supplementary Table 5. NISE generated exatecan binder designs 91
Supplementary Table 6. Chemical structures of ligands used in binding experiments 92
Supplementary Table 7. Structural comparison of 6 top-ranked designs from NISE trajectory using LigandMPNN vs 6 top-ranked designs from NISE trajectory using LASErMPNN..93
Supplementary Table 8. Ranking ablations: top-ranked designs from NISE trajectory that generated EPIC 94
Supplementary Table 9. Structural comparison of top-ranked LigandMPNN CARPdock screen poses vs top-ranked LASErMPNN CARPdock screen poses 95

Supplementary References 96

Supplementary Text

LASErMPNN Overview

Ligand Aware Sequence Engineering Message Passing Neural Network (LASErMPNN) is an autoregressive SE(3)-equivariant graph neural network that takes as input a point cloud containing 3D coordinates encoding a protein backbone and any additional heteroatom coordinates, including metal, nucleic acid, or small-molecule ligand coordinates (Supp. Fig. 1). The model outputs a choice of amino-acid sequence and dihedral angles encoding sidechain orientations, which are used to build all-atom protein structures. The input point cloud is parameterized as a heterograph consisting of two distinct node types corresponding to the protein backbone and non-protein context atoms respectively. LASErMPNN (20,599,412 parameters) consists of three core modules: a pre-trainable ligand encoder, a heterograph encoder, and a heterograph decoder (Supp. Fig. 42).

Heterograph Construction

Protein backbone nodes are created for all backbone "frames" consisting of the N, Cα, C, O, and Cβ atom coordinates for a single residue (Cβ atom coordinates are created from idealized bond lengths and angles if absent). Ligand nodes are constructed for all atoms in the ligand including hydrogens. A heterograph representation of the input structure with distinct edge types for each node pairing (protein–protein, protein–ligand, and ligand–ligand) is constructed by combining the protein-protein and protein-ligand subgraphs. During model training and inference, all protein backbone frames in the input structure are idealized through the alignment of an idealized alanine backbone frame before graph construction, which removes crystallographic artifacts in the backbone that could bias training while also preserving sidechain dihedral angles for the prediction of sidechain conformation.

Protein node representations are initialized to a vector of zeros, and ligand nodes are initialized to concatenated one-hot encodings of the group and period in the periodic table for each atom's atomic number. The 5 nearest ligand-atom neighbors of each ligand atom are computed (including self-edges) for the ligand–ligand graph and distances between them are computed and embedded with Radial Basis Functions (RBFs) as described in ProteinMPNN with 75 bins encoding distances between 0.0 Å and 15 Å[1]. Protein-ligand edges are constructed only for protein nodes with Cα carbons within 20 Å of a ligand atom. The 48 nearest ligand heavy atoms or polar hydrogens are connected to these protein nodes and the distances between all backbone frame atoms and the ligand atom are computed and embedded in RBFs with 75 bins encoding distances between 0.0 Å and 15 Å. Protein-protein edges are constructed similarly with all 25 pairwise distances between backbone frame atoms computed to the nearest 48 protein nodes (including self-edges) and embedded in RBFs with 22 bins encoding distances between 2.0 Å and 22 Å as described in ProteinMPNN. These 3 subgraphs are concatenated by taking the union of all node and edge sets forming the final heterograph representation of a protein-ligand complex.

Ligand Encoder Pretraining

Due to potential limitations in the diversity of small molecules in the PDB, we hypothesized that we could improve the model by pre-training the ligand encoder module that constructs ligand-atom embeddings on a larger, more diverse dataset of 3D ligand conformers. To do this, we use the SPICE-2 dataset which contains over 100,000 protonated systems of small

molecules and ions ranging from 2 to 110 atoms[2]. Each training example has associated atomic partial charges and dipole vectors computed by density functional theory (DFT) calculations, which we use to train a neural network that predicts these features.

The inputs to the ligand encoder module are a nearest-neighbor graph constructed from the 3D ligand coordinates and the element of each atom as described above. We train a graph neural network to predict the partial charges (mbis_charges) and dipole vectors (mbis_dipoles) from the SPICE-2 dataset as well as molecular-connectivity derived features such as atomic hybridization, aromaticity labels, atomic formal charges, and bond orders computed using RDkit[3]. We freeze the weights of this module when training the full LASErMPNN model and use it to generate ligand atom embeddings, which are used downstream when performing ligand-to-protein message passing operations. Freezing these weights during training allows us to check the ligand encoder's understanding of novel ligands by predicting partial and formal charges, hybridizations, etc, and evaluating these predictions against the ground truth.

The ligand encoder graph neural network employs three rounds of GATv2 message passing to build up 256-dimensional node embeddings for each ligand atom[4,5]. From these embeddings, separate 3-layer multilayer perceptron (MLP) adapter layers are used to predict the partial charge, atomic hybridization, formal charge, the number of covalently attached hydrogens, the number of covalently bonded neighbors, and whether the atom is aromatic[4]. These tasks are treated as a classification over discrete bins, and cross-entropy loss is applied and summed across all classification tasks. Dipole vector regression is performed with a 3-layer Geometric Vector Perceptron (GVP) adapter layer[6]. Initial vector representations are computed from a weighted average of neighbor atom displacement vectors and are updated after each round of GATv2 message passing using GVP layers prior to the final dipole regression layer. Only scalar embeddings from the ligand encoder are used for protein-ligand message passing.

LASErMPNN Inputs and Outputs

LASErMPNN discards any pre-existing protein sequence information contained within the input coordinates (unless instructed otherwise) and applies iterative rounds of message passing with its encoder module to build up representations for each protein node. The decoder module takes as input a randomized decoding order which is ultimately used to select a choice of amino acid (aa) and sidechain dihedral angles for each protein node and conditionally updates embeddings from the encoder module with information (predicted aa identities and dihedral angles) about previously decoded nodes.

The output of LASErMPNN is a protein sequence and associated dihedral angles that are used to create a 3D model of a protein structure constructed from the idealized input backbone and any available context atoms, including ligand heteroatoms. The model produces predicted orientations for all sidechain atoms including rotatable and non-rotatable hydrogens placements enabling the evaluation of designed hydrogen bonding interactions directly from LASErMPNN outputs.

LASErMPNN HeteroGATv2 Message Passing

LASErMPNN extends the ProteinMPNN architecture with SE(3)-equivariant vector representations using Geometric Vector Perceptron (GVP) layers and a Graph Attention Network inspired heterograph message passing formulation. The GVP layers are used to place equivariant vector representations on all protein nodes, which are used to encode residue orientation (Supp. Fig. 43). We hypothesized that these vector representations would be useful for rotamer

prediction as this task inherently depends on a robust encoding of frame orientation. To extend the ProteinMPNN architecture to handle protein-ligand heterographs, we combine ProteinMPNN message passing layers with a heterograph generalization of the Graph Attention Network (GAT) architecture[4,5]. We apply this "Hetero-GATv2" architecture to jointly handle ligand-to-protein messages and protein-to-protein messages in both the encoder and decoder modules of LASErMPNN with message passing operations defined as follows:

$$\alpha_{ij} = \underset{j \in \mathcal{N}_i}{\textbf{softmax}} \left(a^T \textbf{LeakyReLU}(W[h_{i,scalar} || h_{j,scalar} || e_{ij}])\right)$$

with separate learnable a and W matrices for protein-protein and protein-ligand node pairs. $h_{i,scalar}$ and $h_{j,scalar}$ refer to the scalar representations stored in node i and node j respectively while e_{ij} refers to the edge information stored for the edge connecting nodes i and j. "||" denotes concatenation. A single softmax operation is applied over all pre-softmax attention coefficients so attention can be selectively paid to nodes of potentially different types in the neighborhood of a given node i. Node updates are computed as follows with W' which is a separate set of learnable parameters from W for a given node pair with l denoting the index of the current message passing layer:

$$h_{i,\ scalar}^{l+1,\ update} = \sum_{j \in \mathcal{N}_i} \alpha_{ij} W' [h_{i,scalar}^{l} || h_{j,scalar}^{l} || e_{ij}^{l}]$$

The final scalar representation update is then computed with residual connections as in the ProteinMPNN architecture:

$$h_{i,\ scalar}^{l+1} = \textbf{LayerNorm}\left(h_{i,\ scalar}^{l} + \textbf{dropout}(h_{i,\ scalar}^{l+1,\ update})\right)$$

Vector representation updates are computed separately from the scalar updates using a GVP layer:

$$h_{i,\ vector}^{l+1} = \textbf{GVP}\left(h_{i,\ scalar}^{l+1,\ update}, h_{i,\ vector}^{l}\right)$$

<u>LASErMPNN Loss Terms</u>

We hypothesized that we could improve the quality of incorrect predictions made by the model by introducing a loss based on statistics from multiple sequence alignments (MSAs), which we accessed from OpenProteinSet[7]. To do this, we add an additional cross-entropy term to the loss for residues whose highest probability is not the ground-truth. We compute a target distribution for these incorrectly predicted residues by computing per-column aa frequencies from the MSA and then compute the cross-entropy of the sequence logits produced by the model vs these frequencies. We dampen this additional loss term as a function of the depth of the MSA, linearly interpolating the weight between 0 for MSAs of depth 0 and 1 for MSAs of at least twice the median depth of 96 sequences. To prioritize sequence recovery, this loss term is further dampened by a factor of 0.1 relative to the main sequence classification loss.

To predict the dihedral angles of rotatable sidechains, we discretize the angles into 5-degree bins and encode these into a 72-bin circularly symmetric version of the RBF encoding used in the protein-protein edges to encode distance information (as described in ProteinMPNN). An offset between +/- 2.5 degrees within each bin is regressed with a mean squared error loss term and is summed with the angle represented by the predicted bin for a final dihedral angle

prediction. The choice of dihedral angles is conditioned on the embeddings used to produce the choice of aa and the identity of the selected aa. Zero to four dihedral angles are decoded for each residue, and previously decoded dihedral angles are used to compute the next angle until the rotamer is fully decoded. Angles which do not exist for a given amino acid are predicted but masked out from the loss during training. Overall, the loss used in training the LASErMPNN model is:

$$loss = seq\_loss + chi\_loss + (0.1 \times msa\_loss) + (0.5 \times chi\_offset\_loss)$$

where seq_loss refers to the cross-entropy term over the protein sequence, chi_loss refers to a cross entropy loss over the predicted dihedral angle bins, msa_loss refers to a cross entropy computed from the sequence logits to the frequencies computed from MSA frequency statistics, and chi_offset_loss refers to a mean squared error loss term applied to the offset between +/- 2.5 degrees of the predicted chi angle degrees within each 5 degree dihedral angle bin.

Training & Inference Differences

During training, the sequence and rotamers are teacher-forced to condition on the ground truth for any previously decoded states. This means that when predicting dihedral angles, regardless of what the highest predicted aa probability would be, the model is told what the ground truth aa is before the first dihedral angle is predicted ensuring ground truth values are available for the loss computation. We use causal masks to selectively provide information as it would be available during autoregressive decoding while allowing fast computations of logits for all residues in parallel during training. During inference, the sequence and rotamers are sampled in a fully autoregressive manner following the input decoding order. Starting at the first residue in the decoding order, a choice of aa is predicted, then its first sidechain dihedral angle is predicted conditioned on the sequence choice and decoder embeddings. The process repeats, conditioning on previous samples until each of the sidechain dihedral angles (up to four) are predicted. The model then moves to the next protein node in the decoding order and repeats the sampling until all residues are designed.

Protein Residue Frame Noising

Small changes in the relative positioning of the backbone coordinates can result in high RMSD (> 1.0 Å) when rebuilding large rotamers from sidechain dihedral angles due to the lever-arm effect. To noise the protein complex to increase model robustness while also ensuring the model may still learn to predict sensible dihedral angles, we apply noise at the level of the backbone frame rather than on each backbone atom independently. This keeps the relative positioning of the N, Cα, C, O, and Cβ coordinates constant and minimizes the lever-arm effect ensuring predicted dihedral angles remain close to the ground-truth before noising. We find that this strategy combined with backbone frame idealization removes any subtle information leakage from crystallographic artifacts contained in the backbone frame coordinates while improving the quality of the model's predicted rotamers over noising each atom independently. We train the LASErMPNN model with 0.1 Å Gaussian noise applied to the protein backbone frames and 0.05 Å Gaussian noise applied to each ligand atom independently. The protein backbone noise level of 0.1 Å was determined by sweeping sequence foldability as a function of noise for a monomeric test set (see Training noise levels and foldability optimization).

Training LASErMPNN

To train the LASErMPNN model, we treat the task of sequence prediction as a classification over the 20 natural amino acids and therefore penalize incorrect sequence predictions with a sum of cross-entropy loss normalized by the number of "valid" residues. To define a valid residue, we select only canonical residues from the chains that were sampled from the dataset to construct the current batch. We mask out from the loss any residues in contact with a non-canonical amino acid as well as those which were loaded as part of the same biological assembly as the sampled chain.

To define an epoch of training, we sample once from each 30% sequence identity cluster (see Dataset Curation and Splitting), selecting a subcluster at random (70% sequence identity), then choosing one sequence from within each subcluster. If the sampled sequence exists in multiple biological assemblies, we sample one at random. We use a similar chain masking strategy to that used to train ProteinMPNN and selectively mask information about similar chains based on structural homology thresholds. Specifically, any chain loaded as part of the same biological assembly as the sampled chain with a TM-score less than 0.7 is provided as sequence and structure context for conditional generation, otherwise the sequence and rotamer identities are masked and decoded with the sampled chain with cross-entropy loss only applied to the sampled chain.

Deep learning experiments were performed using PyTorch and PyTorch Geometric primarily on 4x NVIDIA A6000 GPUs. Models were trained until train and test sequence accuracy curves intersected (typically around 60,000 optimizer steps) using the NoamOpt optimizer with learning rate annealing as was described in ProteinMPNN. Models were trained with a node dimensionality of 256 and an edge dimensionality of 128 on complexes of less than 2,500 residues or ligand atoms.

van Der Mer (vdM)-Inspired Pseudoligand Sampling

To increase the diversity of binding pockets the model is trained on, we use a residue atomization strategy similar to that reported in recent work[8,9]. Briefly, we use individual amino acids that are part of the polypeptide chain as "pseudoligands" (Supp. Fig. 42). We choose pseudoligands with at least 3 contacting residues (heavy atoms within 5 Å) that are distant in the linear polypeptide sequence (greater than 6 residues in primary sequence). We drop the sequence-local residues from the protein graph to form synthetic binding sites. We compute phi and psi backbone dihedral angles for each residue and use these to align methyl caps onto the sampled residue backbone frame, ensuring the residue is built with explicit hydrogens and is thus in-distribution with the training data of the ligand encoder.

Protein Data Bank Dataset Curation

To construct the LASErMPNN training dataset, we began with all experimentally determined structures in the RCSB PDB deposited before 06/01/2022. We downloaded mmCIF formatted data for all biological assemblies. We filtered out structures with over 5,000 amino acids or ligand residues and selected structures generated by X-ray crystallography or cryo-electron microscopy with at least 3.5 Å resolution. We then ran Reduce on the dataset to predict explicit hydrogen locations, adjust protonation states, and to perform N/Q/H residue rotamer flips[10].

Covalently modified and non-canonical amino acids were recorded as "X" residues with masks stored which drop these and any contacting residues (heavy atoms within 5.0 Å) from the loss during training as "X" residues cannot be used for sequence design. We then idealize the backbone frames of all residues by aligning an ideal alanine onto each residue's crystallographic N, Cα, and C coordinates and rotate the idealized O atoms using the psi angle. Sidechain dihedral angles are then computed relative to these idealized frames, which are used as the ground-truth for rotamer decoding.

We tracked the full experimentally characterized sequences for each protein monomer within each biological assembly using information contained in the header of the mmCIF files and mapped these sequences to the OpenProteinSet MSA database which were used to compute an MSA-based loss term during training[7]. The dataset was then split into clusters of 30% sequence identity using MMseqs2 easy-cluster and then sub-clustered further at 70% sequence identity within each cluster (see MMseqs2 sequence clustering code)[11]. The full processed dataset can be found on Zenodo[12].

LigandMPNN Dataset Split Reconstruction

The LigandMPNN[9] training code and dataset are not currently publicly available. An exact reconstruction of the model is therefore difficult as the sequence clusters used in the LigandMPNN dataset were not reported, and the test/validation splits are reported at the level of PDB codes rather than at the level of protein chains. Since biological assemblies of a given PDB code may contain multiple protein chains belonging to different sequence clusters, there may be train set chains in the test set biological assemblies making direct comparison between architectures difficult. Subtle differences in training cutoffs and resolution thresholds may also further complicate these comparisons. For the fairest comparisons possible, we reconstruct the LigandMPNN training dataset starting from our curated PDB data.

To do this, we dropped any 30% sequence identity cluster from our dataset containing any chain with a PDB code in the reported LigandMPNN validation or test sets. We then ran MMseqs2 easy-search to check the remaining data available for training for any sequences over 30% similarity to those in the validation or test sets ensuring there was no contamination. We then reimplement the LigandMPNN architecture and retrain it on our reconstructed LigandMPNN training dataset and evaluate the performance of these models on the "small-molecule" test set PDB codes (as opposed to the nucleotide/metal test set PDB codes which, while still held-out from training, we did not seek to optimize performance for).

Streptavidin Hold-out Dataset Split Construction

We also sought to create a dataset split which rigorously holds out the streptavidin fold to evaluate model generalization to novel backbones. To do this, we hold out the 30% sequence identity cluster belonging to the streptavidin protein family and then further remove any sequences over 30% identity over at least 25 aligned residues to any sequence in the dropped streptavidin cluster using MMseqs2 easy-search. To check for structural contamination and streptavidin fusion proteins, we TM-aligned all members of the streptavidin sequence cluster against all remaining data available for training. To do this we took the first chain of all streptavidin cluster biological assemblies to check for structural homology to the monomer as well as the full biological assembly. We then dropped any structures from the training dataset with a monomer or biological assembly TM score above 0.5 with greater than 25 aligned residues. We searched the PDB for all complexes containing biotin (residue code BTN) and

visually inspected the set of structures containing BTN which were not yet dropped from the training dataset for the streptavidin fold, confirming the absence of any streptavidin-like folds bound to biotin. Finally, we checked the remaining dataset for any chains with CATH assignments of "Avidin-Like" as well as for circular permutants of the streptavidin sequence by permuting the PDB Code 4jnj sequence in 10 residue increments and checking the remaining dataset with MMseqs2 easy-search. These analyses confirmed that all streptavidin-like sequences and structures were dropped from the training dataset for the streptavidin held-out split.

NISE Protocol Implementation for Exatecan Binders

We devised an iterative "flexible-backbone" design loop (NISE) and applied it to an initial protein-exatecan binding pose generated by COMBS[13]. The NISE protocol uses LASErMPNN to design many proteins for the input pose (expansion) and uses protein–ligand co-structure prediction to generate ligand-bound structures for each sequence. Throughout the design loop, we keep a running list of up to 3 backbones sorted by highest backbone priority observed over all iterations of the design loop. In this work, we determined backbone priority as ligand pLDDT predicted by RoseTTAFold All-Atom (RFAA)[14]. By maximizing ligand confidence through ligand pLDDT, protein confidence (which can be tracked through protein Cα pLDDT) is implicitly also maximized as the two values are highly correlated (Supp. Fig. 41). We assigned the input backbone a priority of 1, ensuring it was used for sequence generation throughout the loop. As a consequence, two additional protein–ligand poses were considered for input to LASErMPNN for sequence generation at each iteration. (Using the input backbone within each sampling loop is not necessary for NISE optimization, and we omitted this in the design of apixaban binders.) Each protein–ligand pose was used to generate 1000 sequences that were then predicted by RFAA using 15 recycles. Sequences with high protein Cα pLDDT (> 0.8) and low backbone Cα and ligand heavy atom RMSDs (< 1.5 Å) to the previous round's input to LASErMPNN were ranked by ligand pLDDT. All ligand RMSDs were computed only over the heavy atoms in the ring system, excluding the rotatable carbon chain on the lactone ring of exatecan. New backbones (up to two) were chosen each round if ligand pLDDT surpassed that of a previously observed design. In principle, by computing self-consistency to the previous round (*vs* always to the initial input structure), the ligand could drift away from its initial position after many iterations; in practice, we see minimal drift, likely due to the strong positive correlation between ligand pLDDT and ligand burial. An early version of the LASErMPNN weights (prior to hyperparameter tuning) was used for sequence generation, the weights and inference code for which are available on GitHub under the name *epic_weights.pt*.

Design Filtering and Ranking

The NISE protocol was run for 35 iterations, at which point the ligand pLDDT had converged. We pooled the sequences across each iteration (100,000 sequences total) and filtered by self-consistency metrics (Supp. Fig. 11). Namely, we filtered out designs that did not satisfy any of the following criteria: i) protein Cα RMSD < 1.5 Å, ii) ligand heavy-atom RMSD < 1.0 Å, iii) ligand pLDDT > 0.8, iv) protein Cα pLDDT > 0.8, and v) a core packing metric (packstat) computed with the program Rosetta > 0.55 after sidechain and backbone minimization using the Rosetta energy function. Rosetta energy minimization of the protein-ligand complex (backbone, sidechain, and ligand) was applied using the TaskAwareMinMover and Ref15 score function to the RFAA output structures to produce protonated structures which could be evaluated for

designed hydrogen bonding interactions. Designs with protein and ligand RMSDs between the RFAA output and the energy minimized structure greater than 1.5 Å were also removed.

To check for binding site preorganization, we also applied Rosetta energy minimization in the absence of the ligand to allow relaxation of binding-site residues. We reintroduced the ligand into the unbound structure by superimposition of the bound structure onto the unbound structure via binding-site residues. We then counted heavy atom clashes with the ligand (sidechain atoms within 2 Å of any ligand heavy atom). We removed designs for which the minimized unbound protein had any clashes with the ligand. Designs containing cysteine residues or buried charged residues were also removed. We then ranked designs for experimental characterization based on the number of buried, non-H-bonded, polar atoms of the ligand using the Rosetta energy-minimized structures. Two copies of each designed protein sequence were predicted using AlphaFold and were visually inspected for domain swaps or low interface pAE (pAE < 14; residues within 5.0 Å in predicted multimer), which we used as a proxy for aggregation potential. Four of the top ten exatecan designs remained after this process and were chosen for experimental characterization. Reranking these designs using a linear combination of the number of buried, non-H-bonded ("unsatisfied"), polar atoms (BUNs) of the protein and ligand (rather than only using the contribution from the ligand) ranks the EPIC sequence 1st out of the remaining top 4 designs (Supp. Table 5). We used BUNs Score = 2x ligand BUNs + 1x protein BUNs.

<u>Benchmarking LASErMPNN with Streptavidin–Biotin complex</u>

We used LASErMPNN to design sequences for monomeric streptavidin bound to biotin. We used each chain in the asymmetric unit of PDB accession 4jnj to design 10,000 sequences (2,500 per chain). For this task, we used the LASErMPNN model trained on 0.1 Å noise with the strict streptavidin held-out dataset. We designed sequences using sampling temperature of 1.0 for the binding-site residues and a temperature of 0.1 for all other residues. We sampled sidechain dihedral angles using argmax sampling (1e-6 chi angle sampling temperature). The designed sequences had self-consistent backbones when folded in single-sequence mode by RFAA, but RFAA struggled to predict the correct binding mode of biotin in most cases. When the same sequences were folded using the Boltz-1 model[15] in single-sequence mode, the correct binding mode was observed in most designs, suggesting that alternative co-structure predictors may be more robust than RFAA (Supp. Fig. 3).

We filtered the Boltz-1 predicted structures for self-consistency (protein Cα RMSD < 2.0 Å and ligand heavy-atom RMSD < 2.0 Å). After ranking the remaining ~1700 designs by BUNs Score, we found the design with the highest binding-site sequence recovery in the top 25 designs (binding-site sequence defined as residues that are within 5 Å heavy-atom distance of a biotin heavy atom). We also saw a general correlation between ranking by BUNs and binding-site sequence recovery (Supp. Fig. 5). On average, designs with lower BUNs Score had a higher binding-site sequence recovery.

In the design with the highest binding-site sequence recovery, LASErMPNN chose two residues (His25, Trp130) that disagreed with the binding site of monomeric streptavidin (pdb 4jnj; Ser25, Phe130) but are present in a multiple sequence alignment (MSA). Thus, the sampled sequence is plausibly compatible with binding. The following are percentages of amino acids at these two positions in the MSA of monomeric streptavidin (pdb 4jnj; psiblast, 2 iterations, default parameters): <u>Residue 130:</u> {F:96.67, L:0.10, W:0.10, Y:3.13}, <u>Residue 25:</u> {A:0.71,

D:1.01, E:0.71, F:4.76, G:1.52, H:6.28, I:1.52, K:0.51, L:56.13, M:0.81, N:3.95, P:0.20, Q:0.30, R:11.65, S:5.57, T:0.10, V:0.41, Y:3.85}. The full MSA is provided in Zenodo[16].

Testing ranking protocol of LASErMPNN designs using rucaparib-PiB complex

We used a previously designed four-helix bundle, PiB (PARP-inhibitor binder), that tightly binds the drug rucaparib, as a relevant test case for ranking LASErMPNN designs (Supp. Fig. 6). We used chain A from the rucaparib-PiB crystal structure (PDB accession 8tn6) and the baseline LASErMPNN model (laser_weights_0p1A_noise_ligandmpnn_split.pt) trained with 0.1 Å frame noise. We generated 1000 sequences using a sequence sampling temperature of 0.2 with sidechain dihedral angles sampled at argmax temperature (1e-6) with Cys residues disabled.

To filter the designed sequences, we applied self-consistency filters of 1.5 Å for both protein Cα RMSD and ligand heavy-atom RMSD, as well as a ligand pLDDT filter of > 0.8 and a protein Cα pLDDT filter of > 0.85. A final preorganization filter was applied by removing the ligand and running fixed-backbone Rosetta sidechain energy minimization in its absence, then subsequently realigning the ligand into the repacked binding site. Clashes were defined as sidechain heavy atoms within 2.0 Å of the ligand heavy atoms and only the resulting 211 designs with 0 clashes were kept for the final ranking.

The BUNs Score described above for streptavidin-biotin was used to rank the remaining 211 designed sequences. PiB was ranked as the 4th best sequence (using ligand pLDDT to break ties between designs with the same BUNs Score). 6 designs generated by LASErMPNN scored the same as the PiB sequence by BUNs Score. The highest binding-site recovery design was 78.9% and ranked 32nd by BUNs Score, using pLDDT for tie breaking.

Details of ablation studies

We ablated several model features to quantify their contributions to the computational performance of LASErMPNN (Extended Data Table 1). Ablations were performed on a baseline model trained with node- and edge-embedding dimensions of 256, an optimizer warmup rate of 5,000 steps, backbone frame noise of 0.1 Å, and ligand noise of 0.05 Å. Models which use a pretrained ligand encoder (SPICE2 training dataset) use the same model with node dimension of 256 and edge embedding dimension of 128. Performance was analyzed over the "small-molecule" subset of our reconstructed LigandMPNN test set. All LASErMPNN models were trained to convergence using idealized backbone frames and frame-noising (train and validation sequence recovery curves intersecting, typically around 60,000 optimizer steps). We retrained a LigandMPNN model, which we trained using per-atom noising with idealized backbone frames, 2% residue atomization (rather than our pseudoligand sampling protocol), node/edge dimensionalities of 128, and 300,000 optimizer steps (as reported by its authors[9]). All evaluations were performed on the same test set of 317 small-molecule binding proteins from the LigandMPNN test set with idealized backbone frames to avoid information leakage from artifacts in the crystallographic backbone coordinates emulating the de novo design task.

For all sequence recovery and BLOSUM62 substitution score calculations, 5 sequences were generated by each model using argmax sampling (1e-6 temperature) for both sequence and sidechain dihedral angles (where applicable). Results were averaged over the selected residues. The LigandMPNN (retrained) model refers to a reimplementation of the LigandMPNN architecture. While the LigandMPNN (author weights) model refers to the weights trained by the LigandMPNN authors (ligandmpnn_v_32_010_25.pt) on their unreleased processed dataset and clustering of PDB chains. Differences in the clustering of the PDB chains used during training

could be responsible for the observed performance difference between the LigandMPNN public weights and our reimplementation. Our reimplementation allows for a direct architecture comparison as it was trained on the exact same dataset.

We computed BLOSUM62 substitution scores (A higher score is a better substitution.) to quantify the quality of the incorrect sequence predictions made by the models (Extended Data Table 1). After computing sequence recovery, any incorrectly predicted residues were scored using the BLOSUM62 matrix as a proxy for physiochemical similarity. The final BLOSUM62 score averages the substitution scores of incorrectly predicted residues over binding sites (residues with heavy atoms within 5.0 Å of a ligand heavy atom) or the full sequence.

The "LASErMPNN (add edge angle features) model" performs the best by sequence recovery metrics and BLOSUM62 substitution scores. This model extends the baseline LASErMPNN model by adding angular information about ligand atom placement relative to the backbone, inspired by the "_make_angle_features" function used in LigandMPNN, but implements the encoding of angular information using dot-products of the learned equivariant protein node vector representations with normalized ligand atom displacement vectors rather than using Gram-Schmidt decomposition to build local frames. The performance reported for all models is relative to the baseline LASErMPNN model which does not incorporate these ligand displacement angular features.

Ablating either the MSA loss or sidechain-dihedral-angle loss results in model performance similar to a reimplementation of the LigandMPNN architecture, which is expected as these models were trained with the same objective functions. Ablating the pretrained ligand encoder results in worse performance compared to the LigandMPNN architecture, indicating that the ligand encoder of LASErMPNN may be more difficult to optimize than the ligand encoder of LigandMPNN using the PDB alone. This result may be rationalized by the ligand encoder of LASErMPNN containing more optimizable parameters (~3,000,000 vs ~400,000 in LigandMPNN). Ablating the equivariant protein node vector representations, by initializing them with zeros, drops performance slightly, suggesting these have a small contribution to overall performance.

Neural proofreading protocol

We sought to identify single amino-acid mutations that could improve binding affinity of EPIC for exatecan. We used LASErMPNN in a process we call neural proofreading to determine if any residues near the ligand could be mutated to a higher probability residue, given the identities of the remainder of the EPIC sequence. An energy-minimized RoseTTAFold-All Atom prediction of the EPIC sequence (and exatecan ligand) was used as input to the proofreading protocol. Energy minimization was performed using OpenMM[17] and the Amber-14 forcefield with the SMIRNOFFTemplateGenerator to parameterize the ligand forcefield. The energy-minimized structure of exatecan-bound EPIC was fed into LASErMPNN, and the sequence identities of all residues were fixed.

The binding site residues were identified as those with heavy atoms within 5 Å of any ligand heavy atom. Each binding-site residue was independently unfrozen and sequence probabilities were computed conditioned on the identities of the remaining 147 residues of the EPIC sequence. LASErMPNN probabilities show a slight dependence on the decoding order (the order in which the sequence and dihedral angles are chosen), despite being trained using many random decoding orders. We therefore used multiple decoding orders to provide additional robustness to the computed leave-one-out probabilities. We also turned on dropout for additional

diversity in the sampled probability distributions. During proofreading, the model is additionally tasked with repacking the sidechains of all residues, since changes in neighboring rotamers may also influence proofreading probabilities. Proofreading probabilities were created by averaging over 10 decoding orders and 10 model dropouts then renormalizing the resulting distribution. We selected mutations in two buried positions that showed higher probabilities than the amino acid in the EPIC sequence, precluding any mutations that would introduce buried unsatisfied polar atoms. We chose a Gln to Asn mutation at position 51 ($EPIC^{Q51N}$) and a Met to Leu mutation at position 97 ($EPIC^{M97L}$), both of which showed higher probability through proofreading (Fig. 4b and Supp. Fig. 18). An early version of the LASErMPNN weights (prior to hyperparameter tuning) was used for sequence generation, the weights and inference code for which are available on GitHub under the name *epic_proofreading_weights.pt*.

Training noise levels and foldability optimization

To tune the hyperparameter that governs the amount of gaussian noise applied to the per-residue frames of the protein, we subsampled the LigandMPNN split validation set for 882 monomeric protein backbones with less than 300 amino acids. We swept the noise level applied to backbone frames and computed the Cα-LDDT between the AlphaFold2-predicted structures of designed sequences and the input crystal structures to LASErMPNN (Supp. Fig. 2). We identified 0.1 Å frame noise to maximize sequence recovery and AlphaFold2 success rates.

AlphaFold3 Self-Consistency Test Set Evaluation

We wished to uncover which version of LASErMPNN or LigandMPNN could best perform for the small-molecule-binding protein design task. For this, we used a proxy of achieving self-consistent protein-ligand co-structures with AlphaFold3 (AF3). To test this, we compare the LASErMPNN model to our retrained LigandMPNN model on i) a small set of monomeric proteins from the LigandMPNN test set, ii) the initial helical bundle backbone used as input to the NISE optimization loop and iii) a computationally generated beta barrel structure (b11_L3B_11c.pdb)[18]. Using each network, we generated 250 sequences for each input protein-ligand complex, and predicted protein-ligand co-structures with AF3 using 10 recycles and 5 diffusion samples.

Overall, the LASErMPNN architecture and LigandMPNN architectures perform similarly well for these tasks (Supp. Figs. 44–46. We saw a slightly lower binding-site and ligand RMSD from sequences generated by LASErMPNN for the de novo backbones (Supp. Figs. 44–46). Where performance between the models differed, we hypothesized that some of this difference may come from the backbone-frame noising strategy used to train the baseline LASErMPNN model (LASErMPNN Frame Noise). In this noising strategy we only idealize the frames and add translational noise (no rotational noise) to preserve the dihedral angle predictions. We therefore trained and tested a different version of LASErMPNN with a per-atom noising strategy on top of the idealized frames (LASErMPNN Per-Atom Noise). Indeed, in a structure where the baseline LASErMPNN model underperforms relative to the LigandMPNN architecture with respect to self-consistency RMSDs (PDB accession 5eng), the LASErMPNN Per-Atom noise model appears to bridge the performance gap (Supp. Fig. 44).

The models perform similarly when comparing the confidence of predicted structures and native sequence recovery (Supp. Figs. 45 and 46). The baseline LASErMPNN model generates slightly higher ligand pLDDT (AF3) sequences than the other models on the de novo backbones when considering only self-consistent sequences.

Computation of Buried, Non-H-bonded Atoms

To compute the number of buried, non-H-bonded, polar atoms in a design, we energy-minimized (sidechain and backbone) the predicted protein-ligand co-structures from RoseTTAFold-All Atom using RosettaScripts and the '-no_optH False' command line flag (see Rosetta code for fixed backbone sidechain minimization)[19]. The resulting protonated and energy-minimized structures were analyzed for buried, non-H-bonded, polar atoms. To determine atom burial, we compute a protein surface from a convex hull of Cα and Cβ coordinates using an alpha-sphere size of 9 Å to define the interior and exterior of the protein[13]. We calculate the distance of polar atoms to the convex hull to determine burial and calculate H-bonding partners by interatomic distance (2.5 – 3.5 Å heavy-atom distance).

Rotamer Analysis

We evaluated the sidechain packing performance of the baseline LASErMPNN model using the LigandMPNN test set (Supp. Fig. 47). We fixed each native protein sequence and generated 5 packing solutions with different decoding orders at argmax chi-angle sampling temperature for each protein complex. We group each residue into 3 burial categories based on the number of Cβ atoms within 10 Å of its Cβ atom. Sidechain heavy-atom RMSD was computed accounting for sidechain atom symmetries for ARG, ASP, GLU, PHE, and TYR.

Analysis of NISE Design Trajectory

Throughout the iterative selection and expansion algorithm, the confidence of the sequences generated at each iteration by the LASErMPNN model, P(sequence | structure, ligand), can be tracked by monitoring the negative log-likelihood (NLL). As an amino acid sequence is generated, the probability of each residue being the amino acid that was sampled during the decoding process can be tracked. This is computed as $NLL = \langle -\log(p_i) \rangle$, where p_i is the probability generated by LASErMPNN of residue i being the sampled amino acid with the mean taken over all residues in the protein. Lower values of the NLL correspond to more confident sequences generated by the model. The confidence of the ligand placement generated by structure-prediction methods like RFAA, which we loosely approximate as P(structure | sequence, ligand), can be approximated with the ligand pLDDT (higher values more confident). We track the optimization of pLDDT and sequence NLL throughout the optimization cycle initialized with the COMBS generated exatecan binder starting pose (Figs. 1c and Supp. Fig. 26). Plotting the mean pLDDT and NLL per iteration shows that the loop rapidly increases in both sequence and structure confidence for the generated datapoints and then plateaus. The optimization using RFAA appears noisier than an identical optimization using Boltz-1 as the structure-prediction network. The exact values to which the optimization converges are a function of the noisy optimization process and the starting pose as constraints on self-consistency restrain the space of structure that NISE can explore to structures that are similar to the input pose.

Rosetta Iterative Selection–Expansion Trajectory

An iterative selection–expansion trajectory was constructed which mimics that which generated EPIC while replacing the structure prediction network (RFAA) with Rosetta for backbone relaxation (Supp. Fig. 9 and Fig. 1c, right). In place of pLDDT from RFAA, we used Rosetta ligand energy as a readout of pose quality. To do this, the LASErMPNN model was used

to generate sequences and initial rotamer predictions which were then energy minimized in Rosetta. Energy minimization over the backbone and sidechains was performed using RosettaScripts with a fixed ligand conformer. The total ligand energy was extracted from the Rosetta output pdb file. For the next round of design, we selected the top-3 structures with lowest ligand energy, protein Cα RMSD < 1.5 Å, and ligand heavy atom RMSD < 1.5 Å, additionally requiring the ligand to remain buried in the convex hull of the protein (defined by the protein Cα coordinates). We repeated this iterative selection-expansion process for 70 iterations. Repeating this analysis with a flexible ligand conformer during energy minimization produces very similar results (Supp. Fig. 10). Using Rosetta ligand energy to guide an iterative selection–expansion trajectory is a less efficient optimizer of LASErMPNN sequence likelihood compared to using ligand pLDDT from the RFAA or Boltz-1 structure-prediction networks (Supp. Fig. 48). When using Rosetta instead of co-structure prediction, designs fail to optimize, suggesting that current empirical energy functions cannot fully capture the nuances of productive protein–ligand interactions. This might explain the limited success using LigandMPNN coupled to Rosetta energy minimization[20,21]. While extensive iterative sampling was not used in that work, our analysis suggests that an iterative selection–expansion algorithm using LigandMPNN and Rosetta might not have produced optimal designs. (In that work, apixaban was docked into thousands of NTF2 backbones, and three cycles of LigandMPNN-based sequence design and Rosetta minimization were applied. From 9,024 experimentally tested designs, four binders were found (K_d of 0.68, 3, 6, and 10 μM); of these, only one showed the intended 1:1 binding stoichiometry (K_d = 0.68 μM).) The mean pairwise sequence identity between our 6 experimentally tested NISE sequences (Fig. 6 and Extended Data Fig. 9) and the 0.68 μM binder (pdb 8vez) was 16%.

Correlation of Ligand pLDDT and Binding Affinity using AF3, Boltz-1, and RFAA

We wondered if ligand confidence of predicted structures would correlate with binding affinity between EPIC, its mutants, and various ligands (Extended Data Fig. 6). Protein-ligand co-structure prediction was performed using AF3 and Boltz-1 with default inference parameters (10 recycles, 5 diffusion samples vs 3 recycles, 1 diffusion sample). RFAA sampling was modified to use 100 recycles. For certain ligands and for $EPIC^{Q51N}$ (red circles, Extended Data Fig. 6), RFAA predicts a flipped orientation despite the large number of recycles.

Creation of Exatecan Conformers

We created two exatecan conformers starting from the coordinates of small-molecule crystal structure of a camptothecin derivative deposited in the Cambridge Structural Database (CSD, deposition number 1504071, 7-ethyl-10-hydroxycamptothecin monohydrate). We modified the structure using Avogadro software (by deleting atoms of the camptothecin derivative that do not belong in exatecan and adding atoms unique to exatecan) and minimized the newly built coordinates using the UFF forcefield in Avogadro, including all hydrogens (Supp. Fig. 7, boxes). All coordinates of the original camptothecin derivate that were shared with exatecan were frozen during energy minimization. The two exatecan conformers differed by a 220-degree rotation about the torsional angle of the rotatable ethyl substituent (Supp. Fig. 7, black arrows). The alternate torsional angle was observed from another small-molecule crystal structure of the same camptothecin derivative in the CSD (deposition number 1060443).

Small Molecules

Small molecules were purchased from commercial providers. Belotecan hydrochloride (Selleck Chemical, S6631) and irinotecan (SC-269253, Santa Cruz Technology) were dissolved at a concentration of 10 mM in DMSO. Exatecan mesylate (HY-13631A, MedChemExpress) was dissolved at a concentration of 3.76 mM in DMSO. Camptothecin (13637S, Cell Signaling Technology) was dissolved at a concentration of 5 mM in DMSO. FL118 (HY-12486, MedChemExpress) was dissolved at a concentration of 2.55 mM in DMSO. Apixaban (HY-50667, MedChemExpress) was dissolved at a concentration of 10 mM in DMSO. All small-molecule stock solutions were aliquoted and stored at -20 °C until use.

Family-wide Hallucination of Four-helix Bundles

We generated forty single-chain four-helix bundle proteins using a combination of parametric design, structural bioinformatics, generative AI for loop generation, and a graph neural network for designability assessment. We first computed 750 four-chain coiled coils, with each helix consisting of 40 residues, using the CCCP server (https://grigoryanlab.org/cccp/index.gen.php) and sampling Crick parameter ranges from designable distributions seen in natural proteins. We focused on coiled coils with large supercoil radius (> 7.6 Å and < 8.0 Å) to fit small-molecule ligands in the interior. For each coiled coil, we then used the program MASTER[22] to search for appropriate loop lengths to connect the helices, by searching a non-redundant database of the PDB with each protein separated into files containing individual, non-redundant chains (30% sequence identity). We used as a structural search query the terminal 7 residues of each helix (truncated to 28 residues each) to be connected by a loop and structural matches defined as a Cα RMSD < 0.8 Å. We varied the number of "gap residues" from 2 to 17 and chose the optimal loop lengths that summed to 36 residues, creating single chain proteins of 148 amino acids in length. The optimal number of 36 residues was chosen based on maximizing a score, averaged across each of the coiled coil structures, which favored many MASTER matches with low Cα RMSD (score = # of unique MASTER matches / <Cα RMSD>). We then ran RFdiffusion to generate loops with the chosen optimal lengths (which could be different lengths for different coiled coils, with the constraint that the total number of loop residues sums to 36). RFdiffusion generated 8 single-chain structures for each coiled coil. We then designed 16 sequences for each backbone using ProteinMPNN (v_48_020 model, T=0.15, no Cys allowed) and predicted their structures using AlphaFold2 (model 4, 1 recycle, single-sequence mode). We filtered the predicted structures using the following criteria: average Cα pLDDT > 0.9, average pAE < 5.0, Cα RMSD to the input structure (output from RFdiffusion) < 1.0 Å. Of these proteins, we selected designs whose input backbones to ProteinMPNN produced 8 or more (out of 16) sequences that passed these filters. Finally, we removed any designs with minimum Cα pLDDT of any non-terminal residue < 0.85. If 3 or more of the 16 sequences for a backbone remained after this final filtering, we added the protein to a final pool. We then clustered this final pool of designs by Cα RMSD (using a cutoff of 0.75 Å) after all-by-all pairwise superposition. 40 clusters were found, and we chose the centroids of each cluster as our hallucinated, highly designable ensemble to be used for design of small-molecule binders. Many of the final designs came from different parent parametric backbones (at most 5 from the same original parametric backbone with mostly singletons). For design, we directly used the AF2-predicted structures after removing the ProteinMPNN-designed sequence (by turning all residues to Gly or Ala using Rosetta). The ensemble of forty single-chain bundles,

including the ProteinMPNN-designed sequences along with the Ala/Gly versions, are available on Zenodo[16].

Fluorescence Polarization Experiments to Determine Binding Affinities

We performed fluorescence anisotropy experiments in a 384-well plate-reader format to determine the binding affinities of proteins with various ligands (Supp. Fig. 15 and Supp. Table 6). We serially diluted an initial solution containing a high concentration of protein while keeping a constant concentration of ligand (50 nM) in each well. The buffer for these assays was composed of 1x PBS, pH 7.4, and 0.1% w/v PEG-8000. Camptothecin-class molecules (exatecan, camptothecin, belotecan, FL118, irinotecan) were excited at a wavelength of 360 nm using a BMG LabTech PHERAstar FSX plate reader or a BMG LabTech CLARIOstar Plus plate reader. We detected the resulting parallel and perpendicularly polarized emission at 440 nm. Molecules containing a FITC fluorophore (apixaban-peg-FITC, dexamethasone-peg-FITC) were excited using 482 nm filter (16 nm bandwidth) using a BMG LabTech CLARIOstar Plus plate reader, and parallel and perpendicularly polarized emissions were detected through a 530 nm filter (40 nm bandwidth). Data was fit using the quadratic form of a single-site binding model. Gain settings for each molecule were calculated by auto-adjusting the focus on a well containing free ligand and buffer, setting the millipolarization (mP) of free ligand to mP = 35.

Fits to Fluorescence Anisotropy Data

Data from fluorescence anisotropy (r) experiments, which varied total protein concentration, $[P]_T$ for a fixed total ligand concentration, $[L]_T$, was fit to a single-site binding model (**Equations 1** and **2**, $N = 1$). Fits were consistent across at least 2 biological replicates. For EPIC and its mutants, confidence intervals of the fit parameters were determined from bootstrapping the residuals of the optimum fit (random residual selection with replacement) to create 1000 synthetic datasets, which were each separately fit to **Equations 1** and **2**. Parameter means and 95% confidence intervals were determined from the distribution of the parameters computed from the bootstrapped fits.

We performed a global fit for mutant EPIC proteins, which had affinities with exatecan comparable to the ligand concentration (Supp. Fig. 19). We performed separate protein titrations across three fixed exatecan concentrations (25, 12.5, and 5 nM) and measured fluorescence anisotropy of exatecan as a function of protein concentration. For each EPIC mutant, the dissociation constant (K_d) and anisotropy baseline (A_{min}) were shared parameters across the three exatecan concentrations. Anisotropy maxima (A_{max}) and ligand concentration ($[L]_T$) were fit locally. We fit the ligand concentrations locally (to within 15% of the target concentration) to account for experimental discrepancies. Code for performing the fitting is provided in Zenodo[16].

We also performed a global fit for the apixaban-binding protein, APEX, which had a high affinity with both apixaban-peg-FITC (apx-FITC) and apixaban, with dissociation constants comparable to or lower than the ligand concentration (Fig. 6e,f and Supp. Fig. 34). We performed separate protein titrations across three fixed apx-FITC concentrations (25, 10, and 5 nM) and measured fluorescence anisotropy of apx-FITC as a function of APEX concentration. For these same apx-FITC concentrations, we performed competition experiments with apixaban as the competitor. We used the protein titrations that we measured for binding to apx-FITC to choose protein concentrations for each apx-FITC concentration that produced an anisotropy between 70 – 90% of the maximum (fully bound) value. For these concentrations of protein and apx-FITC, we titrated apixaban, allowing the complexes to equilibrate overnight. We fit both the

apx-FITC binding data and the apixaban competition data simultaneously to a single-site binding model, with $K_d^{apx\text{-}FITC}$ and K_d^{apx} fit globally across the 3 separate apx-FITC concentrations. A_{max} was also fit globally for each apx-FITC concentration (across the APEX and apixaban titrations). Other parameters were fit locally but inspected to have converged to within 10% of each other. Code for performing the fitting is provided in Zenodo[16].

For APEX and other apixaban-binding designs (apx-2.2–2.6), confidence intervals of the fit parameters were determined from bootstrapping the residuals of the optimum fit (random residual selection with replacement) to create 500 synthetic datasets (1000 for APEX), which were each separately fit to coupled binding models (binding to apx-FITC and competition binding of apixaban, Extended Data Fig. 9). Parameter 95% confidence intervals were determined from the distribution of the parameters computed from the bootstrapped fits.

Equation 1

$$PL([P]_T, [L]_T, K_d, N) = \frac{1}{2}\left[K_d + [L]_T + \frac{[P]_T}{N} - \sqrt{\left(K_d + [L]_T + \frac{[P]_T}{N}\right)^2 - 4[L]_T\frac{[P]_T}{N}}\right]$$

Equation 2

$$r = \left(\frac{[PL]}{[L]_T}(A_{max} - A_{min})\right) + A_{min}$$

<u>Cloning, Protein Expression, and Protein Purification</u>

Cloning. Genes were ordered from Integrated DNA Technologies (IDT) and cloned into a peAIP32 plasmid using a Golden Gate reaction (cloning site BsaI). The plasmid also included a 6x His tag followed by a Cth Sumo protease cleavage sequence. We cloned the plasmid resulting from the Golden Gate reaction directly into competent BL21 *E. Coli* prepared with the Zymo Research Mix-n'-Go kit. Cells were then plated onto LB/kanamycin, and single colonies were selected for whole-plasmid nanopore sequencing (Quintara Biosciences). Once cells were verified to contain the correct plasmid, glycerol stocks were created and used for induction of subsequent growths.

Protein Expression. Growths (0.5 L each) were carried out in LB/kanamycin media containing 34.7 mM lactose for autoinduction. Cultures were allowed to grow overnight, for at least 16 hours, at 30 C shaking at 220 rpm.

Protein Purification. Following overnight growth, cells were centrifuged. Liquid media was discarded, and cell pellets were immediately resuspended in lysis buffer (PBS, 500 mM NaCl) then directly lysed on ice by sonication. Cell debris was removed by centrifugation at 16000 rpm (11600 x g) for 30 minutes. Protein was purified using magnetic Ni NTA beads (GenScript). A total of 1.5 mL of beads was incubated with each 0.5 L growth for at least an hour at 4 °C. The slurry was then added to a gravity column, and the flow-through was discarded. Washes performed included one 15 mL wash with wash buffer (PBS, 500 mM NaCl, 20 mM imidazole) and one 10 mL wash with PBS. The beads were then allowed to shake overnight at 4 C with Cth protease (1 µM) to cleave designed protein from the beads. Elution containing the purified cleaved protein was collected using a gravity column. The beads were washed with one column volume equivalent (1.5 mL) of PBS, which was collected and pooled with the elution. The resulting protein was stored in PBS buffer at 4 °C until use in experiments.

Size-exclusion Chromatography

Size exclusion chromatography (SEC) was carried out to determine the oligomerization state of each construct using a BioRad NGC system paired with a BioRad ENrich 650 chromatography column. Each protein eluted at column volumes that approximately matched their molecular weights (Supp. Figs. 14 and 21). Any constructs that were determined to have a minor oligomeric peak (only applicable to the COMBS/Rosetta designs) were purified via fractionation using the BioRad ENrich 650 column to isolate the major peak corresponding to the monomer.

Circular Dichroism to Determine Secondary Structure and Stability

We used circular dichroism (CD) spectroscopy to determine the secondary structure content and thermal stability of EPIC (Supp. Fig. 17). A sample of 0.1 mg/mL EPIC protein was purified by SEC into 1x PBS buffer. CD spectra were collected using a 0.1 cm path-length quartz cuvette (VWR) in a Jasco J-1500 CD Spectropolarimeter with temperature controlled at 25 °C using a Peltier temperature controller. Spectra were collected from 195 nm to 260 nm in continuous scanning mode, with a band width of 1 nm, scanning speed of 50 nm/min, data pitch of 0.1 nm, digital integration time of 1 second, and an average of 5 accumulations. Temperature-dependent spectra were similarly collected using a Peltier temperature controller to vary the sample temperature in the range of 25 °C to 95 °C with an interval of 5 °C, an increase rate of 10 °C/minute, and an average of 3 accumulations.

Hydrolysis Experiments

Exatecan has a labile lactone ring that hydrolyzes (opens) to a carboxylate at physiological pH. We spectroscopically monitored lactone ring opening by collecting time-dependent absorption spectra using an Agilent 8453 G1103A spectrophotometer and a 1 cm path-length quartz cuvette. Samples in a PBS solution (pH 7.4) with a final volume of 2 mL included 20 µM protein and 20 µM ligand (final concentration of DMSO at < 0.54%). Spectra between 190 and 900 nm were collected every half hour until spectral changes were no longer observed. For exatecan with $EPIC^{Q51N/M97L}$, we measured spectra until 49 h and observed no significant change in the absorbance spectrum of exatecan. Data was comparable across at least 2 biological replicates.

Binding to HSA is Compatible with Hydrolysis

To simulate conditions in human plasma, we tracked hydrolysis of exatecan via a time-resolved absorption experiment with a mixture of $EPIC^{Q51N/M97L}$ and human serum albumin (HSA). We first mixed EPIC (40 µM) and exatecan (40 uM) in PBS for a total volume of 1 mL and allowed the solution to equilibrate at room temperature for an hour. We then combined the solution with 1 mL of HSA (1 mM) in PBS, yielding a final solution containing 20 uM $EPIC^{Q51N/M97L}$, 20 uM exatecan, and 500 µM HSA. Absorbance spectra were collected every half hour using an Agilent 8453 G1103A spectrophotometer with a 3 mL 1 cm path-length quartz cuvette. A scattering component grew over time, presumably due to oxidation of the highly concentrated HSA. The scattering component was also observed in experiments with 500 µM HSA alone.

Analysis of Exatecan Hydrolysis Data

Absorption spectra of exatecan and exatecan–protein mixtures were collected at various time delays after initial mixing from a DMSO stock of ring-closed exatecan (Supp. Fig. 24). Spectra were globally fit to a two-state kinetic model that solves for both the forward and backward rate constants (ring-closed to open states) as well as the corresponding species-associated spectra (Supp. Fig. 25). Samples that included HSA showed a time dependent increase in scattering, which we could mostly remove by fitting a polynomial to the absorption spectrum between 450 and 850 nm. For consistency, we applied this baseline procedure to each dataset before fitting the kinetic model. Fits to the time-resolved absorbance data were generally very good using a two-state model (small and normally distributed residuals). Because there was little time evolution observed for exatecan when mixed with EPIC, the two species-associated spectra derived from the fit were nearly identical. We therefore constrained the fits to obey a final equilibrium distribution of 90% ring-closed for EPIC, computed from using a thermodynamic model that incorporated the measured K_d of EPIC for exatecan. Using the equilibrium constraint, the resulting species-associated spectra more closely resembled those of ring-open and ring-closed observed from fits of exatecan only and exatecan with HSA. A single-state model with no time evolution (initialized in 100% ring-closed state of exatecan) was sufficient to model the data of exatecan with $EPIC^{Q51N/M97L}$. A yield of < 1% for the ring-open form of exatecan was computed from a thermodynamic model that incorporated the measured K_d of $EPIC^{Q51N/M97L}$ with exatecan. Code for performing the fitting analysis is provided in Zenodo[16].

Design of Exatecan Binders via COMBS and Rosetta

We used an advanced version of COMBS (code provided), together with the two exatecan conformers and the 40 four-helix bundles, to simultaneously dock exatecan and design ligand-binding sites. COMBS uses van der Mers (vdMs) to first place fragments in favorable positions with respect to an input backbone. COMBS then docks the ligand to overlay these fragments with low heavy-atom RMSD. COMBS then uses a recursive H-bonding algorithm that treats polar, first-shell (i.e. contacting the ligand) sidechains as new "ligands" and finds second- and (sometimes) third-shell interactions that support the binding site in an H-bond network. The entire extended binding site is optimized using a statistical vdM energy function that considers steric clashing between sidechains and pairwise interactions modeled by vdMs. We also used a custom ligand constraint file in COMBS to only consider ligand positions that buried the lactone ring of exatecan and positioned the charged amine close to the protein surface. We additionally filtered any exatecan ligands that did not have at least 75% of apolar heavy atoms buried in the protein core (inside the convex hull created by the Cα atoms of the backbone). We also required that polar atoms of exatecan had an H-bonding interaction if the atoms were buried in the protein core. Lastly, we required that the exatecan A, B, or C rings (see chemical structure in Supp. Table 6 for ring labels) had at least one van der Waals contact from a designed residue. For each of the forty backbones, we used COMBS to output 75 designs with high variability in binding-site sequence and ligand location. We pooled these designs and selected 100 candidates based on COMBS statistical vdM energy and the number of buried polar atoms not participating in an H-bonding interaction. We then used a custom flexible backbone design algorithm implemented in Rosetta to design the remainder of the protein sequence. For this, the binding-site residue identities were frozen and designed H-bonding distances were constrained using a Rosetta constraint file. For each of the selected COMBS designs, we used Rosetta to fill in the remainder of the sequence, outputting 100 sequences each (which shared the same COMBS-derived

residues). We then used AF2 and RFAA to predict the unbound and ligand-bound structures of each of these designs (approximately 10,000 sequences), selecting designs for experimental interrogation by the following criteria: ligand heavy-atom RMSD < 2.0 Å, ligand heavy-atom RMSD over the ring system < 1.5 Å, binding-site Cα RMSD < 0.9 Å (RFAA, aligning on binding-site residues only, defined as those with heavy atoms within 5 Å of the ligand), overall Cα RMSD < 1.0 Å (RFAA, aligning over all residues), average Cα pLDDT > 0.80 (RFAA), average Cα pLDDT > 0.90 (AF2), average protein pAE < 5.0 (RFAA), average protein pAE < 5.0 (AF2), binding-site Cα RMSD < 0.9 Å (AF2, aligning on binding-site residues only), overall Cα RMSD < 1.0 Å (AF2, aligning over all residues), number of buried unsatisfied atoms computed by Rosetta (BuriedUnsatHbonds filter) < 2, minimum pLDDT (AF2) of any non-terminal residue > 0.7, and average interfacial pAE > 15 (AF2 multimer, two copies of the sequence). After this filtering, 64 designs remained. We grouped them by those that shared the same input to Rosetta and ranked designs by a heuristic score that favors low Cα RMSD over the binding-site residues [score = 0.5 ligand heavy-atom RMSD + 1.0 binding-site Cα RMSD (RFAA) + 0.75 binding-site Cα RMSD (AF2) + 0.25 overall Cα RMSD (RFAA) + 0.25 overall Cα RMSD (AF2)]. We chose the top 3 (lowest scoring) designs from each group, leading to 24 designs overall. We ordered synthesized DNA for these sequences from IDT (e-block) and experimentally tested 16 of them. We subsequently ordered two additional designs for the input pose to NISE, which we selected by score from the 100 outputs of Rosetta that used the same COMBS input. The computational models of the designs are available in Zenodo[16].

Initial COMBS Model for NISE design

The initial model for the input to the NISE design algorithm, CR4, was chosen based on the heuristic score described above. The input to NISE was the COMBS/Rosetta design that had the lowest heuristic score. It was also one of the top-ranked designs when using a strategy involving LASErMPNN design to design 5 sequences and co-structure prediction (and ranking by ligand pLDDT or ligand heavy-atom RMSD) (Supp. Fig. 49). Size exclusion chromatography showed that CR4 formed a higher order oligomer when expressed and purified from *E. coli.* While CR4 showed an increase in fluorescence polarization when mixed with exatecan, the data could not be fit to a single-site binding model, presumably due to the oligomeric nature of the protein.

Comparison of Input COMBS Model vs EPIC Design

The model used as input to NISE (CR4) was designed using van der Mers (vdMs) to both dock the ligand and establish many of the binding interactions, including a second-shell His-Glu interaction (Supp. Fig. 13). Residues derived from vdMs were frozen, and the remainder of the sequence was designed using a flexible-backbone design loop in Rosetta. The CR4 model was ranked the highest among the 16 models designed by COMBS and Rosetta when applying a heuristic self-consistency score. Although CR4 aggregated, it showed some signs of binding exatecan via fluorescence polarization experiments, but the fits were not consistent with a single-site model. A similar pose from traditional COMBS/Rosetta design, CR3, which used the same vdMs of the binding site, was monomeric and bound exatecan with an 8 μM dissociation constant. The NISE protocol not only resulted in sequences that were monomeric, but each of the four tested NISE designs bound exatecan, with EPIC binding the tightest (K_d = 0.12 μM). The EPIC binding site differed starkly from that of CR4 (Supp. Fig. 13). EPIC used fewer polar

residues and more aliphatic residues, which still engaging with key polar groups of exatecan, such as the hydroxyl and amine.

Selection of Additional Pair of COMBS Designs for Experimental Characterization

Because each of the four NISE designs bound exatecan, we sought to test more of the traditional designs that shared the same pose (backbone and ligand position) as the input to NISE. Two of the sixteen traditional designs share the same pose, one of which (CR4) aggregated and the other (CR3) bound exatecan with K_d = 8 μM. These two designs were the top 2 of 100 COMBS/Rosetta designs when ranked by a heuristic self-consistency score (see above). We selected the next two highest ranking designs from the remaining 98 using the same scoring function after applying the same filters described in section Design of Exatecan Binders via COMBS and Rosetta. These designs are CR5 and CR6 (Supp. Table 1).

Ranking of COMBS Designs by Metrics from Protein–ligand Co-structure Prediction

We used RFAA, Boltz-1, and AF3 to predict structures of the 16 designs from COMBS and Rosetta (see Supp. Table 1 for sequences). Both ligand confidence (pLDDT) and ligand self-consistency (heavy-atom RMSD) could be used to rank binders over non-binders (Supp. Fig. 16). RFAA ligand pLDDT and AF3 ligand RMSD showed the best ranking performance.

Ranking of Designable Poses by Metrics from Protein–ligand Co-structure prediction

We wondered if the most promising poses could be prioritized for input to NISE through a rapid computational screen using LASErMPNN and a structure-prediction method. To do this, we used the computational models of the 16 experimentally characterized COMBS/Rosetta sequences. For each COMBS/Rosetta design, we removed the sequence information and used only the backbone and ligand coordinates (pose) as input to LASErMPNN. If the COMBS/Rosetta design bound exatecan, we designated the pose as a binder. We designed 5 sequences using LASErMPNN and predicted their exatecan-bound structures using RFAA, Boltz-1, and AF3. AF3 showed the best performance, with ligand pLDDT and ligand heavy-atom RMSD both correlating with binding (Supp. Fig. 49). Both AF3 and Boltz-1 showed enrichment for binders when ranked by ligand RMSD after first filtering designs that had low self-consistency (Cα RMSD or ligand heavy-atom RMSD > 2 Å, Supp. Fig. 49b,c). The three experimentally verified binders from COMBS were top-ranked, suggesting that the backbone- and ligand coordinates were particularly designable (Supp. Fig. 49). In contrast to results using the designed COMBS/Rosetta sequences (Supp. Fig. 16), metrics from RFAA showed the worst performance, as neither variable correlated with binding (not shown). These results show that LASErMPNN and AF3/Boltz-1 can feasibly be used to rapidly screen for promising poses (ligands docked to a backbone by a variety of methods) as input into the more computationally intensive NISE algorithm for optimization.

X-ray Crystal Structures

EPIC and EPIC^{Q51N} were purified as described above. EPIC solutions were diluted so that exatecan from a DMSO stock (3.76 mM) could be added at a 1:1 molar ratio while keeping the total concentration of DMSO < 2.6% (to about 100 μM). After addition of exatecan, EPIC-exatecan solution was concentrated to 3.5 mM using 3k centrifugal filters (Millipore). We prepared the solution for co-crystallization at room temperature using the sitting drop vapor

method. Crystallization conditions screened included the sparse matrix screens MCSG-1, MCSG-2, MCSG-3, MCSG-4, PEG/Ion, Index, and JCSG Plus. EPIC crystals obtained from 7 µL EPIC-exatecan solution combined with 7 µL of MCSG-3 well D6 solution (0.2 M Zinc Acetate, 0.1 M MES: NaOH, pH 6, 10 % (w/v) PEG 8000) diffracted to a resolution of 2.0 Å. EPIC^{Q51N} crystals obtained from 7 µL EPIC–exatecan solution combined with 7 µL of MCSG-3 well A2 solution (0.1 M Sodium Phosphate Dibasic: Citric Acid, pH 4.2, 2 M Ammonium Sulfate) diffracted to a resolution of 2.2 Å.

Data was collected on a DECTRIS EIGER X 9M detector at the 17-ID-1 (AMX) beamline at the National Synchrotron Light Source II of Brookhaven National Laboratory (wavelength = 0.9201, collection temperature = 77K). Raw images were indexed and merged using autoPROC[23], and the structures were phased via molecular replacement in Phenix[24] using the design models of EPIC and EPIC^{Q51N}. The models were then refined in Phenix and Coot[25]. The composite-omit maps shown in the main article were produced in Phenix (version 1.20.1-4487-000, default parameters of Omit map method: refine). Table 1 statistics (Extended Data Table 2) were produced with the same version of Phenix. For the refined EPIC structure, Ramachandran statistics were 99.49% favored, 0.51% allowed, and 0.00% outliers. For the refined EPIC^{Q51N} structure, Ramachandran statistics were 98.97% favored, 0.68% allowed, and 0.34% outliers.

<u>Comparison of X-ray Crystal Structure and Results from Co-structure Predictors</u>

The structures of EPIC and EPIC^{Q51N} present an opportunity to assess the performance of modern protein–ligand co-structure predictors. Each of three structure predictors accurately computes the backbone of EPIC; the main differences occur at the ligand (Extended Data Fig. 5). While each program generally predicts the correct binding mode of exatecan, they struggle to compute some aspects of the ligand conformation, often producing physically distorted molecules. Boltz-1 and RFAA tend to flatten functional groups into the plane of the extended ring system, e.g. the sp^3 hybridized amine and the lactone. AF3 predicts the bound complex most accurately but still deviates slightly from the true ligand conformation. Interestingly, the ligand pLDDT of predicted co-structures by AF3 rank-orders the different camptothecin-class drugs by affinity (Extended Data Fig. 6c). To a lesser extent, ligand pLDDT from Boltz-1 also correlates with binding affinity ($R^2 = 0.61$, Extended Data Fig. 6). While RFAA was not able to predict a consistent binding mode for the other camptothecin-class ligands, it is slightly more confident in the placement of exatecan for EPIC$^{Q51N/M97L}$ *vs* EPIC (Extended Data Fig. 6).

<u>Creation of NTF2 Scaffold Library</u>

To create an NTF2 scaffold library, we began with 1,615 NTF2 backbones from ref [26]. We used the following protocol to select the most designable scaffolds for a sub-sampled library. We generated 3 sequences for each scaffold using LASErMPNN (no ligands) and predicted the structures of the LASErMPNN-generated sequences with Boltz-2. We filtered the scaffolds for those with which all 3 LASErMPNN sequences were predicted with Cα RMSD < 2.0 Å (predicted *vs* input structure to LASErMPNN), mean Cα pLDDT > 0.85 (excluding the first and last residue of the protein, which tend to be low pLDDT in general), and that the predicted structures had no residue (excluding the first and last residues) with Cα pLDDT < 0.6. This last condition ensures that the loops, which usually contain the residues with lowest pLDDT, are sufficiently designable. After this filtering, 523 scaffolds remained. From these, we generated a

similarity matrix using pairwise TM-alignment (using US-Align), and the resulting matrix was grouped into 50 clusters using the Spectral Clustering protocol from scikit-learn:

```
spectral = SpectralClustering(n_clusters=50, affinity='precomputed',
random_state=0,   assign_labels='cluster_qr')
labels = spectral.fit_predict(tm_align_sim_mtx)
```

We chose cluster representatives as the cluster member with highest average Cα pLDDT from Boltz-2 across the 3 LASErMPNN sequences. This resulted in a final NTF2 scaffold library of 50 designable backbones. These 50 backbones resulted from Boltz-2 structure prediction after LASErMPNN redesigned sequences for the initial 1,615 structures, so they do not have identical backbone coordinates to any of the scaffolds used in ref [26] or ref [21]. However, we ensure that they are highly similar to the initial 1,615 structures and span a range of pocket sizes by filtering by Cα RMSD and clustering by TM-score.

Design of Apixaban-Binding Proteins

All designs of apixaban (Apx)-binding proteins were performed using the following weights of LASErMPNN available on GitHub: laser_weights_0p1A_nothing_heldout.pt. See Supp. Fig. 31 for a visual overview of the design process.

To initialize the design process, we extracted two ligand conformers (which differ by a ring flip) from chains A and C, respectively, of the 6w70 crystal structure (Supp. Fig. 28). These conformers were input to a random ligand roto-translation docking protocol, which we call CARPdock (see Implementation of Rigid Protein-Ligand Docking Protocol). We sought to bias the binding orientations of apx so that a fluorophore-appended apx, apx-FITC (used for fluorescence polarization binding experiments), would have an exit vector where the fluorophore points out of the protein. We constrained the ligand's O2, O3, O4, and C25 atoms to be inside the convex hull, defined by the backbone used in the CARPdock protocol; orientations of the ligand which bury these atoms enrich for poses which expose the N3 atom, which is chemically appended to a peg-FITC. (By not explicitly constraining the N3 atom itself to be outside of the convex hull, poses can be sampled in which the N3 atom is buried but has an unobstructed exit vector that can be identified downstream using Solvent Accessible Surface Area (SASA) once a sequence is designed for the protein backbone.) We generated 100 ligand poses for each of the 2 apx conformers using CARPdock with the 50 backbones in our sub-sampled NTF2 library. We then generated 3 LASErMPNN sequences (sequence temperature = 1e-6) for each of the 10,000 resulting CARPdock poses and used Boltz-2 to predict co-structures from these sequences (default parameters, single sequence mode, using inference potentials).

We filtered the resulting Boltz-2 co-structure predictions by self-consistency (Cα RMSD < 2.0 Å, ligand heavy-atom RMSD < 2.0 Å), solvent accessible surface area (SASA, computed using FreeSASA) of the carboxamide functional group (which contains the N3 atom) greater than 10 Å^2, and a ligand SASA less than the median ligand SASA across all Boltz-2 predictions, which enriches for buried apx. 208 out of 15,000 LASErMPNN sequences passed this filtering for the apx conformer from the 6w70, chain A (apx-conf1), and 269 out of 15,000 LASErMPNN sequences passed the filtering for the apx conformer from 6w70, chain C (apx-conf2). We performed sequence design using LASErMPNN for the resulting 477 apx–NTF2 poses (5x sequences, sequence temperature 1e-6) and subsequent co-structure prediction by Boltz-2 to

nominate the highest confidence predicted co-structures as inputs to the NISE protocol. We selected the top 3 poses for each ligand conformer by the unweighted sum of two metrics from Boltz-2: ligand pLDDT and affinity_probability_binary (i.e. P(bind)). We define the sum of these terms as the "score". If any of the top 3 poses ranked by score were generated by the same input to LASErMPNN, we selected the next highest scoring pose to ensure structurally diverse starting points for NISE. The use of P(bind) tended to push highly confident predictions toward higher shape complementarity between the protein pocket and docked ligand (Supplementary Fig. 29).

We implemented the NISE protocol using Boltz-2 and LASErMPNN to further optimize the results of the CARPdock described above. NISE trajectories maximizing the score (sum of ligand pLDDT and affinity_probability_binary) from the Boltz-2 model were launched for each of the 6 backbones identified by CARPdock. Each trajectory ran to convergence (14 to 28 iterations), until a score > 1.98 was achieved and no change in top backbone score was observed for 5 NISE iterations. In the NISE protocol, we disabled Cys residue sampling and used a chi-angle sampling temperature of 1e-6. We used a general sequence-sampling temperature of 0.5, with the exception of binding-site residues (Ca atom within 10 Å of ligand heavy atoms), for which we used a sampling temperature of 0.7 to encourage diversity. During the NISE trajectory, we used all ligand heavy atoms for the ligand RMSD calculation and no atoms were constrained to be buried or exposed. To filter backbone candidates after each round in NISE, we used a ligand heavy atom self-consistency RMSD threshold of 2.5 Å and a protein Cα self-consistency RMSD threshold of 2.5 Å. Unlike the protocol that generated EPIC, we did not require that the input model be a representative in each round of NISE. The three representative models for each iteration were selected as follows: two were selected by sorting for the highest Boltz-2 score observed thus far in the trajectory, and the third was the model that *generated* the highest scoring model observed thus far in the trajectory (reasoning that additional sequences computed using this model may generate even higher scoring models).

We generated 64 LASErMPNN sequences for each of the 3 representative backbones per NISE iteration. This is significantly fewer than the 1000 sequences generated for each backbone when implementing NISE with RFAA. Fewer sequences were needed because we found that a larger percentage of LASErMPNN sequences were predicted with sufficient self-consistency by Boltz relative to RFAA (Supp. Fig. 3), ultimately making NISE more efficient. Throughout the NISE trajectories, we used constraints over the amino-acid composition to restrict the number of Ala (max of 2) and Gly (max of 0) residues that LASErMPNN could sample at surface residues in helices or beta strands, defined in the input backbone by DSSP. Because MPNNs like LASErMPNN, ProteinMPNN, and LigandMPNN occasionally sample sequences with many repeated Ala or Gly residues (more likely when a part of the input structure is undesignable, i.e. far from structures observed in the PDB training data), these sampling constraints prevent such spurious design.

We pooled designs generated by each NISE trajectory (across all iterations) and filtered for ligand pLDDT > 0.9, protein Cα self-consistency RMSD < 1.5 Å, ligand heavy atom self-consistency RMSD < 1.5 Å, and a threshold on affinity_probability_binary (P(bind)) of either 0.5 or 0.8 (using the stricter cutoff only in the case that an excess of 3,000 designs passed the less strict filter). We energy-minimized the sidechain conformations of designs using Rosetta (Ref15 score function) with fixed backbones and fixed ligand conformers and finally filtered for zero heavy atom clashes upon superposition of the ligand onto the ligand-free binding site of sidechain conformation energy-minimized structure (sidechain conformation only). We selected

the top 250 designs for each NISE trajectory by BUNs score, with rankings tie-broken by ligand pLDDT. A visualization of these filtering and ranking criteria applied to the trajectory which generated APEX is provided in Supp. Fig. 32.

To bias designs against potential oligomerization, we evaluated the top 250 designs from each NISE trajectory using AlphaFold2-multimer (AF2m). We predicted structures containing two copies of each designed sequence using AF2m (using colabfold_batch in single sequence mode, default parameters) for each of the 1,500 top sequences. We then filtered the top ranked AF2m prediction for each sequence (selected by ColabFold "multimer score" across the output from 5 different AF2m models) by requiring high (> 12) interfacial Predicted Aligned Error (iPAE, computed using only residues physically located at the interchain interface, i.e. interchain residues within 5 Å heavy-atom distance of each other), an average interchain Prediction Alignment Error (> 20, predicted across all residues in each chain), low (< 0.15) ipTM, an average Cα pLDDT > 80 for each of the chains, and low Cα RMSD (< 1.0 Å) of chain A of the dimer prediction to the corresponding Boltz-2 design model. 417 sequences passed these filters.

We then used RF3 to predict ligand-free dimer structures for the 417 sequences that passed AF2m filters (num_steps=50, diffusion_batch_size=1, early_stopping_plddt_threshold=null). We filtered predictions based on high (> 15) iPAE, low RMSD between chain A of the predicted dimer model and the Rosetta-energy minimized Boltz-2 prediction (Cα RMSD < 1.0 Å and heavy-atom RMSD of binding-site sidechains < 0.7 Å; binding-site residues defined as those with heavy atoms within 5.0 Å of any ligand heavy atom in the design model). Finally, we used RF3 to predict monomeric, ligand-bound co-structures of the remaining 33 designs and filtered by Cα RMSD < 1.0 Å and ligand heavy-atom RMSD < 1.0 Å to the corresponding ligand-bound Boltz-2 design model. We ranked the remaining designs by BUNs score (tie broken, if necessary, by ligand pLDDT from RF3) and selected the top 2 designs from each NISE trajectory. 4 of the 6 NISE trajectories had sequences that passed all filtering criteria (One of these had just one sequence.), leaving a total of 7 sequences. We dropped one of the final 7 designs after visual inspection showed that binding-site sidechain atoms would clash with the exit vector required for apx-FITC. For any sequence lacking a Trp residue (4 of 6), we prepended a Trp at the N terminus, to aid in quantification of protein concentration via absorbance at 280 nm. We ordered synthetic DNA for these final 6 sequences for experimental characterization.

We provide all scripts used to generate APEX and to filter and rank the sequences from the NISE trajectory in the archive file apex-design-scripts.zip in Zenodo[16].

<u>Implementation of Rigid Protein-Ligand Docking Protocol (CARPdock)</u>

To dock a ligand into a protein backbone, we begin with PDB files of ligand heavy atoms and an all-glycine protein backbone. The protein backbone coordinates are rotated to align its three principal axes (determined using Principal Component Analysis) with the Cartesian coordinate frame. This alignment reduces the number of test points needed for the fixed-width grid used to search ligand translations by making the protein's bounding box as tight and axis-aligned as possible, so the fixed-spacing grid that spans this box contains fewer total test points. We then construct a 3D grid of test points with 0.5 Å spacing to span the max and min Cα coordinate along each of the Cartesian (X, Y, and Z) axes in the rotated backbone. We then filter the test grid points for those which lie within the convex hull formed by the protein Cα coordinates.

Using the input conformer, the ligand's heavy-atom center of mass is translated to each grid point. 1000 random ligand rotations are evaluated at each grid point and tested for steric clashes by calculating distances between the ligand heavy atoms and protein backbone atoms. Ligand poses with heavy-atom clashes (atomic distances below 2.5 Å) are discarded. We describe the remaining poses in terms of their rotation relative to the input ligand conformer and translation on the test grid. A 6-dimensional (6D) vector is constructed from concatenating the 3 translational coordinates of the ligand's center of mass and the 3 rotational parameters (in axis-angle format) describing the orientation of the ligand relative to its input conformer. To reduce redundancy, K-Means Clustering is applied to the 6D vectors describing each rototranslation with K=100. The ligand rototranslation representing the centroid of each cluster is output as a PDB file.

All CARPdock code is open-source and available in github along with an interactive Google Colab notebook demonstrating a minimal version of pose generation by CARPdock and subsequent quality assessment:

CARPdock GitHub Repository:
https://github.com/benf549/CARPdock

Google Colab Notebook:
https://colab.research.google.com/github/benf549/CARPdock/blob/main/run_CARPdock.ipynb

Correlation of Ligand pLDDT with other Confidence Metrics from Boltz-2

In NISE, we use ligand pLDDT as a confidence metric by which to optimize designs. We also included Boltz-2's P(bind) in the NISE designs of apixaban binders. Here, we analyze the correlation of ligand pLDDT from Boltz-2 with other confidence metrics. We used co-structure predictions from 30,000 apx–NTF2 designs, where ligands were docked via CARPdock and sequences were generated by LASErMPNN (see Design of Apixaban Binding Proteins). We randomly sampled 10 percent of the designed sequences (3,000 total) and predicted co-structures with Boltz-2's affinity module (single sequence mode, default parameters, using inference potentials) to extract ligand pLDDT, P(bind), interfacial predicted aligned error (iPAE), ipTM, and protein Cα pLDDT for each prediction. To compute protein Cα pLDDT and ligand pLDDT values, we averaged the values over the relevant atoms extracted from the b-factor column of the output PDB files. We extracted ipTM and P(bind) values from the Boltz-2 output metadata JSON files. iPAE was computed by averaging the values of the PAE submatrix defined between the protein residues at the interface (Cα atoms of chain A within 8 Å of ligand heavy atoms) including all ligand atoms. This data shows that ligand pLDDT is highly correlated with iPAE, ipTM, and protein Cα pLDDT, but not with P(bind) (Extended Data Fig. 8). We therefore reasoned that the joint metric (the unweighted sum) of ligand pLDDT and P(bind) could be synergistic in optimization of designs within NISE trajectories performed with Boltz-2.

LigandMPNN Evaluation on Streptavidin Holdout Split

We retrained the LigandMPNN architecture on the same streptavidin holdout split. The model was trained using 0.1 Å per-atom noise over idealized backbone frames and ligand heavy atoms, 2% residue atomization, and node/edge dimensionalities of 128. It was trained for 100,000 optimizer steps, at which point the train dataset loss and sequence recovery curves had

plateaued. To evaluate the models trained on the streptavidin holdout split, we generated 10,000 sequences using both the retrained LigandMPNN and LASErMPNN (trained on the same dataset) at a sequence sampling temperature of 0.1 and a binding-site temperature of 1.0. We provided each model with the ligand-bound and ligand-free structures for which to design sequences. We designed 2,500 sequences for each of 4 biological assemblies (monomers) in for monomeric streptavidin (PDB 4jnj). The results showed that LASErMPNN outperformed LigandMPNN with respect to sequence recovery for both the ligand-bound and ligand-free structures used as input (Extended Data Fig. 2). We predicted protein–ligand co-structures using Boltz-1 for each designed sequence of the ligand-bound structures. The distributions of Cα RMSD and ligand heavy-atom RMSD (predicted vs crystallographic structures) across the 10,000 sequences generated by each model showed that more sequences designed by LASErMPNN had accurately predicted structures (Supp. Fig. 4). We note that this analysis is only a case study of performance of the two models, where we rigorously held out an entire protein fold (streptavidin); while LASErMPNN outperformed LigandMPNN here, this may not be the general case across all such folds treated in this way.

Comparing LASErMPNN and LigandMPNN within NISE

To evaluate the performance of LigandMPNN as a drop-in replacement of LASErMPNN within NISE, we reimplemented the NISE trajectory that generated APEX using the same starting structure. We used the LigandMPNN model found at https://github.com/dauparas/LigandMPNN with weights ligandmpnn_v_32_010_25.pt. During the trajectory, we sampled 64 sequences per backbone at temperature of 0.5 (LigandMPNN does not implement a binding-site temperature parameter). We observed that, like the NISE trajectory that generated APEX using LASErMPNN (64 sequences sampled per backbone, temperature 0.5, binding site temperature 0.7), the use of LigandMPNN is similarly able to optimize sequence NLL and Boltz-2 score (Supp. Figs. 37 and 38).

We compared the 6 top-ranked designs from the NISE trajectories run with LigandMPNN or LASErMPNN, after repeating the filtering and ranking criteria described in Design of Apixaban-Binding Proteins (Supp. Table 7). Using the predicted co-structures of the apx–NTF2 designs (Boltz-2, sidechain energy-minimized by Rosetta), we quantified the structure and sequence similarity of the closest LigandMPNN design to each LASErMPNN design (as measured by sequence identity over buried residues, Supp. Table 7). We found that protein and ligand structures are generally similar between the LASErMPNN and LigandMPNN designs, with protein Cα RMSD and ligand heavy-atom RMSD around 1.0 Å. Sequence identity of buried residues for all designs is at most 74%, implying LASErMPNN and LigandMPNN mostly agree about the sequence of core-packing residues, with LigandMPNN tending to overpack the cores (Extended Data Fig. 1 and Supp. Fig. 40).

Re-screening CARPdock Poses with LigandMPNN

To evaluate the performance of LigandMPNN to nominate CARPdock outputs as designable inputs to the NISE protocol, we used LigandMPNN to generate 3 sequences at temperature 1e-6 for each of the same 10,000 poses generated by CARPdock that we had previously asessed with LASErMPNN. Sequence design and sidechain packing with LigandMPNN were run using the code released at https://github.com/dauparas/LigandMPNN with weights ligandmpnn_v_32_010_25.pt, executed with the following command:

```
python run.py --pdb_path_multi lmpnn_inputs.json –model ‘ligand_mpnn’ --
temperature 1e-6 --out_folder ./lmpnn_outputs/ --pack_side_chains 1 --
number_of_packs_per_design 1 --pack_with_ligand_context 1 --batch_size 3 --
number_of_batches 1
```

We performed an identical co-structure prediction, filtering, and ranking procedure to evaluate the CARPdock poses as described in Design of Apixaban Binding Proteins, using self-consistency filtering and Boltz-2 score to guide selection of the top poses for each apx conformer. After two rounds of sequence design and structure prediction with Boltz-2, the six top-ranked poses identified by LigandMPNN were qualitatively similar to those identified by LASErMPNN, which we used as input to NISE, resulting in experimentally verified binders (Supp. Fig. 50 and Supp. Table 9). LigandMPNN ranked (by Boltz-2 score) the input pose that lead to APEX as the 4th best CARPdock pose, suggesting that LigandMPNN could be used to identify high-quality starting structures for NISE.

Comparing LASErMPNN and LigandMPNN Designs using Rosetta Energy

We compared the 6 top-ranked apx–NTF2 designs from the NISE trajectories using either LigandMPNN or LASErMPNN (see Comparing LASErMPNN and LigandMPNN within NISE). The designed core residues from LigandMPNN were mostly similar to those from LASErMPNN, but where the two designs differed, LigandMPNN tended to place larger residues, even around the ligand. The binding sites from LigandMPNN looked like they could possibly be overpacked. To probe this phenomenon more systematically and quantitatively, we evaluated the designs generated using both LigandMPNN and LASErMPNN for the CARPdock-generated poses of apx docked into a variety of NTF2 folds (see Design of Apixaban Binding Proteins and Re-screening CARPdock Poses with LigandMPNN). For 30,000 LigandMPNN-designed sequences, we used the direct outputs of LigandMPNN's packing model, which generates sidechain coordinates for a given sequence. We also used the direct output of 30,000 LASErMPNN designs for the same inputs. These 30,000 designs were generated using a diverse number (10,000) of CARPdock poses. Using the direct outputs from each model as well as a sidechain-relaxed structures (Rosetta Ref15 energy function, fixed backbone and ligand), we computed the number of protein heavy atoms within 5 Å of a ligand heavy atom (Extended Data Fig. 1a,b). [Note that, because LigandMPNN does not output structures with hydrogens (though it keeps them on the ligand if they were provided in the input PDB file), while LASErMPNN does, we stripped hydrogen atoms from both MPNN outputs before running the Rosetta Relax script.] We observed that LigandMPNN indeed places on average 5 more atoms within a 5 Å-neighborhood of the ligand than LASErMPNN (Student's t-test $p < 1e\text{-}16$). We suspected that LigandMPNN was overpacking the binding sites, as would be indicated by a higher overall repulsive van der Waals energy between the ligand and protein residues in the first-shell around the ligand (first shell defined as heavy atoms with in 5.0 Å of any ligand heavy atom). To assess this, we used the Rosetta score_jd2 script to compute per-residue energies for each of the direct MPNN outputs, and we extracted per-residue energies from the structures with Rosetta-relaxed sidechains using the energy table contained in PDB files produced by the fixed-backbone, fixed-ligand relax protocol. We inspected the total Rosetta Ref15 energies and the attractive and repulsive van der Waals components of the energies (fa_atr and fa_rep, respectively) for both the

ligand (Extended Data Fig. 1c–h) and the overall pose (Supp. Fig. 40). We observed that LigandMPNN designs have significantly higher total Rosetta energies (both ligand and overall structure) than LASErMPNN designs due to higher repulsive van der Waals energies, suggestive of overpacking by LigandMPNN.

To assess whether the differences between LASErMPNN and LigandMPNN were statistically significant for each quantity of interest (heavy-atom counts around the ligand, total Rosetta energy, fa_atr, and fa_rep), we first compared sample means using a Student's t-test. For all variables, the difference in means was highly significant ($p < 1e\text{-}16$). We then compared the full empirical distributions using both the Mann–Whitney U test and the Kolmogorov–Smirnov test. For every variable, these tests also indicated that the distributions for LASErMPNN and LigandMPNN differ significantly, with $p < 1e\text{-}16$ in all cases.

Rosetta scoring of the MPNN outputs and sidechain-only (fixed-backbone, fixed-ligand) Relax protocols were performed with the following commands:

```
score_jd2.linuxgccrelease -l inputs.txt -extra_res_fa GG2.params -out:pdb -
out:path:score=/dev/null/ -no_optH false -out:path:all ./outputs_dir/

relax.linuxgccrelease -l inputs.txt -extra_res_fa GG2.params -relax:quick -
relax:bb_move=0 -relax:respect_resfile -packing:resfile fix_gg2.resfile -
out:path:all ./outputs_dir/ -out:path:score /dev/null/
```

Where fix_gg2.resfile contains the following:

```
NATRO

START
* A NATAA
* B NATRO
```

Interpretation of NISE Design Success

We attribute success of zero-shot design to *i*) the ability to computationally nominate the most designable backbone–ligand structures as input to NISE – poses generated via COMBS or even brute-force rigid-body docking with a rapid assessment of self-consistency, *ii*) the ability to subsequently optimize the three-dimensional co-structure and sequence, making each iteratively more compatible with the other through selection–expansion, and *iii*) filtering pooled designs by self-consistency, model confidence, and ultimately by a biophysical parameter, i.e. the number of buried, H-bonded polar atoms of ligand and protein.

The only requirement of NISE is an initial structure of a backbone–ligand pair that can be modeled self-consistently by the two reciprocal neural networks. NISE will then optimize the initial structure and sequence. Specialized docking methods, such as RIFdock[18,20,21] or COMBS[13,27], are not necessary for design success. We used COMBS to dock exatecan into four-helix bundles; and we used brute-force rigid-body docking of apixaban within NTF2 folds. Brute-force docking was enabled by subsequent filtering via self-consistency and model. Such filtering is made possible by the rapid speed in which autoregressive graph neural networks like LASErMPNN can design sequences and models like Boltz-2 can predict co-structures. In this

work, we built ligand conformers (for initial docking) based on crystal structures (Supplementary Figs. 7 and 28) but this is not strictly necessary. Ligand conformers could also be built from SMILES strings using standard chemistry software packages, such as RDKit, and subsequently chosen via empirical energy functions[28] or optimized quantum chemically[29].

We found that accuracy of LASErMPNN improved through pretraining its ligand encoder on a large corpus of synthetic ligands[2,30] not seen in the PDB, providing a useful workaround for the lack of chemical diversity in deposited structures. Due to this extensive pretraining, we expect LASErMPNN to adeptly handle a diverse set of ligands, even those not well represented in the PDB, such as exatecan. Moreover, the direct output of the ligand encoder—a ligand's predicted partial charges—can be used to assess the model's understanding of exotic ligands, providing a unique capability relative to LigandMPNN. (Should partial charges differ from those expected, the ligand encoder can be finetuned with a set of synthetic molecules that resemble the target, followed by fast retraining of LASErMPNN.)

NISE is a general algorithm that is agnostic to the particular networks used for sequence design and co-structure prediction. For example, it is straightforward to replace LASErMPNN with LigandMPNN. A similar performance could reasonably be expected because both models were trained on the same underlying dataset (the PDB) and perform similarly with respect to fixed-backbone sequence recovery on a test set of proteins (Fig. 2b and Supplementary Figs. 44–46). However, differences in their training weights and network architectures might make using one model preferable for a given fold or ligand on a case-by-case basis. Using the same apixaban–NTF2 starting point for APEX, we ran a NISE trajectory using LigandMPNN and Boltz-2, which resulted in similar aggregate values of optimized parameters (ligand pLDDT and P(bind), Supplementary Figs. 37 and 38.). However, a closer look at the designed binding-site residues revealed some differences between top designs from each method. Where the binding sites differed, LigandMPNN generally designed larger residues near the ligand (Supplementary Fig. 39). The general overpacking of structures by LigandMPNN (Extended Data Fig. 1 and Supplementary Fig. 40) might be attributed to its disjoint sampling of sequence and sidechain dihedrals: the entire sequence is designed first, followed by all sidechain dihedrals. By contrast, LASErMPNN jointly determines sequence and sidechain dihedrals within the same autoregressive decoding trajectory (Fig. 2a), perhaps affording a more nuanced awareness of sidechain packing that, in turn, influences sequence design.

As co-structure predictors continue to improve, so will NISE. In our initial implementation of NISE for the design of an exatecan binder, we used RFAA for co-structure prediction to good effect, but also found that using next-generation co-structure predictors like Boltz-1/2 helped to more accurately model the ligand and efficiently optimize designs. Indeed, RFAA-based assessment of self-consistency and ligand pLDDT of APEX and other experimentally verified apixaban-binding proteins would have resulted in these designs being discarded (Supplementary Fig. 36); the use of Boltz-2 was important to nominate them for experimental testing, and we advise using Boltz-2 or an equivalent model within NISE going forward. Our work with Boltz-1/2 as a drop-in for co-structure prediction also emphasizes the modularity of NISE: as models for sequence design or co-structure prediction become more capable, their use within NISE is straightforward to implement.

The most striking improvements to designs are made early in NISE trajectories, but we recommend performing iterations even past the plateau of the optimization metric (i.e. ligand pLDDT or score). This is because expansion of sequences and subtle refinement of designs helps

increase the number of designs that pass additional, more computationally expensive, filtering metrics that are applied only after pooling all designs from the trajectory. In our work, we considered, e.g., buried non-H-bonded polar atoms, steric clashing of the ligand after superposition of the ligand-bound protein with a ligand-free protein, and model confidence between predicted structures of homodimers (using high iPAE to bias for monomeric designs). Indeed, the sequences of both EPIC and APEX came from later iterations beyond the plateau in ligand pLDDT or score, emphasizing the utility of performing more iterations. Although co-structure prediction is the bottleneck in NISE (sequence generation using LASErMPNN is very fast), longer trajectories are not prohibitively slow to compute, especially with increased efficiency from modern co-structure predictors like Boltz-2. Using LASErMPNN and Boltz-2, a typical NISE trajectory of 14 iterations takes approximately 5 hours on four A6000 GPUs.

We focused initially on optimization of ligand pLDDT during NISE trajectories. This parameter is highly correlated with other metrics, such as protein Cα pLDDT and iPAE, which is commonly used for protein-binder design[31,32] (Extended Data Fig. 8 and Supplementary Fig. 41). Selecting for improved ligand pLDDT thus also selects for improved confidence in protein structure. We found that ligand pLDDT from Boltz-1/2 and AlphaFold3 was correlated with experimentally determined binding affinity (Extended Data Figs. 6 and 7). Yet, optimizing ligand pLDDT alone is likely insufficient for achieving high affinity binders, as some poses with high ligand pLDDT still had buried, non-H-bonded polar atoms, necessitating further filtering by this biophysical parameter. We observed only a weak correlation with experiment for a dedicated affinity-prediction module of Boltz-2 (Extended Data Fig. 7). As binding-affinity prediction improves, dialing-in of target affinities through NISE should become possible. In the design of APEX, we extended beyond ligand pLDDT by including Boltz-2's P(bind), two uncorrelated metrics that are closely aligned with our design goal. While P(bind) was a good binary predictor of binding *vs* not binding, ligand pLDDT was a better predictor of relative affinity between similar ligands (Extended Data Fig. 7). The use of P(bind) tended to push highly confident predictions toward higher shape complementarity between the protein pocket and docked ligand.

EPIC and APEX NISE Trajectory Lineage Analysis

Starting with the EPIC and APEX sequences generated by NISE, we worked backwards though each NISE iteration, tracing each backbone that LASErMPNN used to generate sequences starting from the NISE input to the final EPIC and APEX sequences (which we call EPIC and APEX design "lineages"). We recorded the evolution of model confidence from co-structure prediction (RFAA's ligand pLDDT for the EPIC lineage and Boltz-2's score for the APEX lineage) for each backbone sampled on the way to the final design. We also recorded the self-consistency RMSDs (protein Cα and ligand heavy atom) and sequence identities to the previous backbone (both overall and buried residues) for each sequence in the lineage. To assess the emergence of specificity throughout the NISE trajectory, we predicted co-structures with off-target ligands (irinotecan and camptothecin for EPIC lineage and exatecan for APEX lineage), providing ligand pLDDT from RFAA for each sequence in the EPIC lineage (Supp. Table 2) and Boltz-2's ligand pLDDT and P(bind) values for each sequence in the APEX lineage (Supp. Table 3). For the NISE trajectory leading to EPIC, camptothecin and irinotecan have lower ligand pLDDT values from RFAA than that of exatecan after the first NISE iteration. Indeed, irinotecan has the lowest ligand pLDDT of the three and was shown experimentally not to bind EPIC (Fig 3g). Similarly, in the NISE trajectory leading to APEX, the ligand pLDDT and P(bind) of

exatecan (no experimentally observed binding) are lower at all NISE iterations than the pLDDT and P(bind) of apixaban.

Ablations of Filtering Metrics

To assess the importance of various combined filtering metrics derived from RFAA confidence metrics (ligand and protein Cα pLDDT) and self-consistency RMSDs for the protein and ligand, we ranked all the LASErMPNN sequences from the NISE trajectory that generated EPIC by each metric independently and report the values of all metrics for top-ranked designs chosen by each individual metric (Supp. Table 8). We also report the BUNs score of each design calculated from the RFAA output and note that EPIC has the lowest BUNs score of all designs identified by ranking via a single metric. Note that our self-consistency RMSD cutoffs for NISE were chosen such that locations of the ligand during sequence design and after structure prediction could still plausibly form the intended molecular interactions.

Prediction of Apixaban-Binding Proteins with RFAA

We predicted protein–ligand co-structures for our six experimentally validated apx-binding proteins using RFAA with 15, 35, and 100 recycles. At higher recycle counts (>35), RFAA predicted protein backbones with Cα RMSD at or below 2.0 Å to Boltz-2 predictions of the same co-structures (single sequence mode, using inference potentials, default parameters). Although ligand centers of mass typically had RMSD values < 2.0 Å, RFAA did not recover the designed binding orientation, with no prediction achieving a ligand heavy-atom RMSD < 5.0 Å relative to the Boltz-2 design model (Supp. Fig. 36). These results suggest that improvements in protein–ligand co-structure prediction models, as implemented in Boltz-2, enabled us to identify designs that we would likely have discarded if we had used RFAA self-consistency to screen LASErMPNN-designed sequences.

Statistical Bootstrapping NISE Confidence Metrics for Uncertainty Quantification

For each parameter of interest, we estimated a 95% confidence interval for the upper quartile of its values (the third quartile for RFAA ligand pLDDT and Boltz-2 score, and the first quartile for MPNN NLL) using nonparametric bootstrap resampling. Given a list of values for that parameter, we drew 100,000 bootstrap samples (with replacement, each of the same length as the original list), computed the corresponding quartile for each bootstrap sample, and then took the 2.5th and 97.5th percentiles of this bootstrap distribution as the bounds of a 95% confidence interval around the empirical quartile estimate.

Evaluating H-Bond Networks for Calculation of Buried, Non-H-bonded Polar Atoms

The code we used to analyze H-bonding to compute “buried, unsatisfied polar atoms” (BUNs) for the design of apixaban-binding proteins is available as a standalone python module at https://github.com/polizzilab/bunsalyze.

NTF2 ligand-binding experiments

We performed fluorescence anisotropy experiments in a 384-well plate-reader format to determine the binding affinities of proteins to apx-FITC (Fig. 6e, Extended Data Fig. 9, and Supp. Fig. 34a). We serially diluted an initial solution containing a high concentration of protein while keeping a constant concentration of apx-FITC (25, 10, 5 nM for APEX experiments and 50

nM for other NTF2 designs) or exatecan (50 nM) in each well. We used a buffer of 1x PBS, pH 7.4, and 0.1% w/v PEG-8000. Measurements were taken after overnight incubation (in a sealed plate) at room temperature to allow equilibration (and after 30 min for exatecan to limit hydrolysis of its lactone to the carboxylate form). The FITC fluorophore in apx-FITC was selectively excited at 482 nm (16 nm bandwidth, set using the linear variable filter monochromator of the instrument) using a BMG LabTech CLARIOstar Plus plate reader, and parallel and perpendicularly polarized emission was detected through a 530 nm filter (40 nm bandwidth). We measured exatecan FP using its intrinsic fluorescence (excitation at 360 nm, 20 nm bandwidth, emission at 440 nm, 10 nm bandwidth filter). Data was fit using the quadratic form of a single-site binding model. The K_d for apx-FITC was fit simultaneously with the K_d for apixaban from competition experiments (see below) using shared parameters of maximum anisotropy. Fits were consistent across at least 2 biological replicates. Gain settings for each experiment were calculated by auto-adjusting the focus on a well containing free ligand and buffer only, setting the millipolarization (mP) of free ligand to mP = 35.

Fluorescence polarization competition experiments for NTF2 designs

We performed fluorescence polarization competition experiments in a 384-well plate-reader format to determine the binding affinities of NTF2 proteins to unlabeled apixaban (Fig. 6f, Extended Data Fig. 9, and Supp. Fig. 34b). Experiments contained a constant concentration of apx-FITC (see above for specific concentrations), a constant concentration of protein (corresponding to 70-90% measured maximum anisotropy in binding experiments with apx-FITC), and a buffer of 1x PBS, pH 7.4, and 0.1% w/v PEG-8000. The apx-FITC/protein solution was incubated at room temperature for 3 hours, followed by addition of apixaban serially diluted across the wells. The sealed plate was then incubated overnight at room temperature. Fluorescence anisotropy of FITC in apx-FITC was measured using a 482 nm excitation (16 nm bandwidth) with a BMG LabTech CLARIOstar Plus plate reader, and parallel and perpendicularly polarized emission was detected through a 530 nm filter (40 nm bandwidth). Gain settings for each experiment were calculated by auto-adjusting the focus on a well containing free apx-FITC and buffer only, setting the millipolarization (mP) of free ligand to mP = 35.

Size-Exclusion Chromatography of NTF2 designs

SEC of the NTF2 designs showed that each design was monomeric. Purified NTF2 designs were injected on a Biorad NGC instrument at a concentration greater than 100 µM and run on a Biorad 650 ENrich chromatography column in PBS pH 7.4 buffer. Constructs 2.4 and 2.5 run slightly larger than the expected molecular weight (~13 kDa), but the results are consistent with monomers (Supp. Fig. 33).

Runtime Quantification

Running in CPU inference mode with 32 CPU cores, LASErMPNN generates 100 designs for a 148-residue protein in around 50 sec. On a NVIDIA A4500 GPU, with the same inference settings, 100 designs can be generated in around 34 sec. One NVIDIA A4500 can run an iteration of the NISE algorithm over 1500 sampled sequences (500 sequences per backbone) using RoseTTAFold-All Atom with 15 recycles in around 6 hr for a 148-residue protein. Running an analogous NISE loop with Boltz-1 (default inference settings) on a single NVIDIA A4500 similarly takes around 6 hr. Using Boltz-1, we noticed that the number of sequences sampled per

NISE iteration can be reduced to around 150 sequences per iteration (50 sequences per backbone) to observe similar quality sequence/structure optimization taking around 40 min per iteration on one NVIDIA A4500. Boltz-1 is more successful at predicting self-consistent structures (Supp. Figs. 3, 8, 27) and is therefore more efficient for NISE optimization. The main time bottleneck in NISE is the structure prediction step, so we used between 4 and 8 GPUs to parallelize the structure-prediction computations while developing the algorithm. Further time savings can be made by reducing the number of recycles or diffusion steps (where applicable) used by the structure-prediction networks. For the design of apixaban-binding proteins using LASErMPNN and Boltz-2, running one NISE trajectory (14 iterations) took approximately 5 hr on 4 NVIDIA A6000 GPUs.

Code availability

All model inference code, training code, processed datasets, and train/test/validation split information can be found at https://github.com/polizzilab/LASErMPNN. An implementation of the NISE protocol using Boltz-1/2 can be found at https://github.com/polizzilab/NISE. Neural networks were trained with Python v3.11.11, PyTorch v2.0.1, NVIDIA CUDA v12.4, NumPy v1.26.4, pytorch-cluster v1.6.3, pytorch-scatter v2.1.2. MMseqs2 v15.6f452 was used to cluster PDB chains, ProDy v2.4.0 was used for PDB structure analysis, RDkit v2024.03.5 was used for small molecule analysis, Reduce 4.14.230914 was used to protonate the training dataset. Data analysis used Python v3.11.11, Matplotlib v3.10.1, Seaborn 0.13.2, NumPy v1.26.4. Structure visualizations were created in PyMol v2.6.2. All statistical tests were performed using SciPy v1.16.1. Design and analysis code for the Neural Iterative Selection Expansion algorithm can be found at doi:10.5281/ZENODO.18308430

NISE pseudocode

Notation Key

G = LASErMPNN // neural network for sequence and dihedral prediction (this work)
F = RFAA // neural network for protein-ligand co-structure prediction
$\mathbf{D}_i = [\mathbf{r}^{bb}{}_i, \mathbf{r}^{lig}{}_i]$ // LASEr input (coordinates of protein backbone and ligand, cycle *i*)
$G(\mathbf{D}_i) : [\mathbf{a}^N{}_i, \mathbf{d}^N{}_i] = \mathbf{s}_i$ // LASEr output (amino-acid identities and sidechain dihedrals)
$\mathbf{Z}_i = [\mathbf{a}^N{}_i, L]$ // RFAA input (amino-acid identities and ligand smiles string)
$F(\mathbf{Z}_i) : [\mathbf{r}^{sc}{}_i, \mathbf{r}^{bb}{}_i, \mathbf{r}^{lig}{}_i] = \mathbf{x}_i$ // RFAA output (coordinates of protein sidechain, backbone, ligand)

NISE Design Algorithm

1) Initialize ligand-docked structure using COMBS or other method:
   $\text{COMBS}(\mathbf{r}^{bb}, \mathbf{r}^{lig}) \rightarrow \mathbf{D}_0$
2) Design *m* sequences using LASErMPNN:
   $G(\mathbf{D}_0) \rightarrow \{\mathbf{s}_0{}^{(0)}, \mathbf{s}_0{}^{(1)}, \ldots, \mathbf{s}_0{}^{(m)}\}$
3) Predict *m* structures from sequences using RFAA:
   $\{F(\mathbf{Z}_0{}^{(0)}), F(\mathbf{Z}_0{}^{(1)}), \ldots, F(\mathbf{Z}_0{}^{(m)})\} \rightarrow \{\mathbf{x}_1{}^{(0)}, \mathbf{x}_1{}^{(1)}, \ldots, \mathbf{x}_1{}^{(m)}\}$
4) Do $i = \{1..p\}$ cycles:
   A) Select top-*k* structures by ligand pLDDT and remove sidechain information:
      $\text{Top-}k(\{\mathbf{x}_i\}) \rightarrow \{\mathbf{D}_i{}^{(k\text{-}2)}, \mathbf{D}_i{}^{(k\text{-}1)}, \mathbf{D}_i{}^{(k)}\}$
   B) Design *m* sequences for each top-*k* structure (including $\mathbf{D}_0$) using LASErMPNN:
      $\{G(\mathbf{D}_0), G(\mathbf{D}_i{}^{(k\text{-}2)}), G(\mathbf{D}_i{}^{(k\text{-}1)}), G(\mathbf{D}_i{}^{(k)})\} \rightarrow \{\mathbf{s}_i{}^{(0)}, \mathbf{s}_i{}^{(1)}, \ldots, \mathbf{s}_i{}^{(m\,*\,(k+1))}\}$
   C) Predict $m * (k+1)$ structures using RFAA:

$\{F(\mathbf{Z}_i^{(0)}), F(\mathbf{Z}_i^{(1)}), \ldots, F(\mathbf{Z}_i^{(m * k+1)})\} \rightarrow \{\mathbf{x}_{i+1}^{(0)}, \mathbf{x}_{i+1}^{(1)}, \ldots, \mathbf{x}_{i+1}^{(m * (k+1))}\}$

5) Pool structures from all cycles, rank with additional criteria, select top-j for expression.

In this work, $m = 1000$, $p = 35$, $k = 3$, and $j = 4$ for exatecan binders using RFAA; $m = 64$, $p = 14 – 28$, $k = 3$, and $j = 6$ for apixaban binders using Boltz-2.

Energy minimization code for Rosetta iterative selection-expansion trajectory

```
<ROSETTASCRIPTS>
      <SCOREFXNS>
            <ScoreFunction name="ref15" weights="ref2015"></ScoreFunction>
      </SCOREFXNS>
      <RESIDUE_SELECTORS>
          <Chain name="chA" chains="A"/>
      </RESIDUE_SELECTORS>
      <TASKOPERATIONS>
            <InitializeFromCommandline name="ifcl"/>
            <OperateOnResidueSubset name="FreezeLigand">
                  <Not selector="chA"/>
                  <PreventRepackingRLT/>
            </OperateOnResidueSubset>
      </TASKOPERATIONS>
      <FILTERS>
            <PackStat name="pack_stat" confidence="0" threshold="0"
      repeats="10"/>
      </FILTERS>
      <MOVERS>
            <TaskAwareMinMover name="minimize_protein" chi="1" bb="1"
      max_iter="10000" tolerance="0.000001"
      task_operations="ifcl,FreezeLigand" scorefxn="ref15" cartesian="0"/>
      </MOVERS>
      <APPLY_TO_POSE>
      </APPLY_TO_POSE>
      <PROTOCOLS>
            <Add mover_name="minimize_protein"/>
            <Add filter_name="pack_stat"/>
      </PROTOCOLS>
      <OUTPUT scorefxn="ref15"/>
</ROSETTASCRIPTS>
```

Rosetta code for fixed backbone sidechain minimization (for BUNs calculation)

```
<ROSETTASCRIPTS>
      <SCOREFXNS>
            <ScoreFunction name="ref15" weights="ref2015">
            </ScoreFunction>
      </SCOREFXNS>
      <RESIDUE_SELECTORS>
      </RESIDUE_SELECTORS>
      <TASKOPERATIONS>
            <InitializeFromCommandline name="ifcl"/>
      </TASKOPERATIONS>
      <FILTERS>
            <PackStat name="pack_stat" confidence="0" threshold="0"
      repeats="10"/>
      </FILTERS>
      <MOVERS>
            <TaskAwareMinMover name="minmovetaskaw" chi="1" bb="1"
      max_iter="1000000" tolerance="0.000001" task_operations="ifcl"/>
```

```
      </MOVERS>
      <APPLY_TO_POSE>
      </APPLY_TO_POSE>
      <PROTOCOLS>
            <Add mover_name="minmovetaskaw"/>
            <Add filter_name="pack_stat"/>
      </PROTOCOLS>
            <OUTPUT scorefxn="ref15"/>
</ROSETTASCRIPTS>
```

<u>Rosetta apo structure repacking code</u>

```
<ROSETTASCRIPTS>
      <SCOREFXNS>
            <ScoreFunction name="ref15" weights="ref2015">
            </ScoreFunction>
      </SCOREFXNS>
      <TASKOPERATIONS>
            <InitializeFromCommandline name="ifcl"/>
            <RestrictToRepacking name="repack_only" />
      </TASKOPERATIONS>
      <FILTERS>
      </FILTERS>
      <MOVERS>
            <PackRotamersMover name="packer"
      task_operations="ifcl,repack_only"/>
            <TaskAwareMinMover name="minmovetaskaw" chi="1" bb="0"
      max_iter="1000000" tolerance="0.000001" task_operations="ifcl"/>
      </MOVERS>
      <APPLY_TO_POSE>
      </APPLY_TO_POSE>
      <PROTOCOLS>
            <Add mover="packer"/>
            <Add mover="minmovetaskaw"/>
      </PROTOCOLS>
            <OUTPUT scorefxn="ref15"/>
</ROSETTASCRIPTS>
```

<u>Rosetta ligand params file generation code</u>

```
import io
from rdkit_to_params import Params
from rdkit import Chem
from rdkit.Chem import AllChem
import prody as pr

protein = pr.parsePDB(input_pdb)
ligand = protein.select('hetero').copy()
lignames_set = set(ligand.getResnames())

pdb_stream = io.StringIO()
pr.writePDBStream(pdb_stream, ligand)
ligand_string = pdb_stream.getvalue()

# Load the ligand PDB and convert it to an RDKit molecule, correct the bond
      orders and re-add hydrogens.
mol = Chem.MolFromPDBBlock(ligand_string)
smiles_mol = Chem.MolFromSmiles(smiles_string)
mol = AllChem.AssignBondOrdersFromTemplate(smiles_mol, mol)
mol = AllChem.AddHs(mol, addCoords=True)
AllChem.ComputeGasteigerCharges(mol)
Chem.MolToMolFile(mol, sdf_output_path)
```

```
ligname = lignames_set.pop()
p = Params.from_mol(mol, name='LG1') # RFAA outputs are always LG1 for the
      ligand name.
p.dump(params_output_path)
```

AMBER energy minimization code

```
import io
import openmm
from openmm import Platform
import matplotlib.pyplot as plt
from openmm.app import Simulation
from openmmforcefields.generators import SMIRNOFFTemplateGenerator

# Runs constant temperature minimization with AMBER forcefield.
# Define ligand forcefield with SMIRNOFF and add to AMBER.
smirnoff = SMIRNOFFTemplateGenerator(molecules=mol)
forcefield = ForceField('amber14-all.xml', 'amber14/tip3pfb.xml')

forcefield.registerTemplateGenerator(smirnoff.generator)

# Create a system with the protein and the ligand.
system = forcefield.createSystem(
    topology=modeller.topology,
    nonbondedCutoff=0.9 * openmm.unit.nanometer,
    switchDistance=0.8 * openmm.unit.nanometer,
)
integrator = openmm.LangevinMiddleIntegrator(300 * openmm.unit.kelvin, 1 /
openmm.unit.picosecond, 0.001 * openmm.unit.picosecond)

platform = Platform.getPlatformByName('CUDA')
properties = {'DeviceIndex': '2', 'Precision': 'mixed'}
simulation = Simulation(modeller.topology, system, integrator, platform,
properties)
simulation.context.setPositions(modeller.positions)

# Record the state before energy minimization as unrelaxed.pdb
simulation_state = simulation.context.getState(getPositions=True)
positions = simulation_state.getPositions()
PDBFile.writeFile(simulation.topology, positions, open('unrelaxed.pdb', 'w'))

# Run Minimization
simulation.minimizeEnergy()

simulation_state = simulation.context.getState(getPositions=True,
getForces=True, getEnergy=True)
relaxed_positions = simulation_state.getPositions()
PDBFile.writeFile(simulation.topology, relaxed_positions, open('relaxed.pdb',
'w'))
```

MMseqs2 sequence-clustering code

```
import subprocess
import pandas as pd

from tqdm.notebook import tqdm
# Run 30% sequence identity clustering with MMSEQS
path_to_sequence_fasta = '…/all_sequences_fasta.fa'
output_prefix_path = '…/30pctID_0p8_coverage'
mmseqs_command = f'mmseqs easy-cluster {path_to_sequence_fasta}
{output_prefix_path}
```

```
/scratch/bfry/sequence_clustering/tmp_30pctID_0p8_coverage --min-seq-id 0.3 -
c 0.8 --cov-mode 5 --cluster-mode 3'
subprocess.run(mmseqs_command, shell=True, stdout=subprocess.DEVNULL)

# Load output clusters
output_tsv_path = output_prefix_path + '_cluster.tsv'
sequence_clusters = pd.read_csv(output_tsv_path, sep='\t', header=None,
names=['cluster_representative', 'chain'])
mmseqs_cluster_keys =
sorted(list(sequence_clusters.cluster_representative.unique()))

# Map from cluster to list of chains
chains_to_cluster =
sequence_clusters.set_index('chain').to_dict()['cluster_representative']
cluster_to_chains = invert_dict(chains_to_cluster)

# Perform 70% identity subclustering
import os
import subprocess
import tempfile
from tqdm.notebook import tqdm
import multiprocess

num_workers = 32
num_threads_per_worker = 4
cpu_regions = [f'{i * num_threads_per_worker}-{(num_threads_per_worker) +
i*num_threads_per_worker - 1}' for i in range(num_workers)]

def run_mmseqs(cluster, chain_to_sequence_dict, cpu_region):
    with tempfile.TemporaryDirectory() as tempdir:
        try:
            count = 0
            unique_sequences = set()
            fasta_path = os.path.join(tempdir, 'sequences.fa')
            with open(fasta_path, 'w') as f:
                for chain in chain_to_sequence_dict:
                    if len(chain_to_sequence_dict[chain]) <= 14:
                        continue
                    f.write(f">{chain}\n{chain_to_sequence_dict[chain]}\n")
                    unique_sequences.add(chain_to_sequence_dict[chain])
                    count += 1

            if count <= 1 or len(unique_sequences) <= 1:
                return {list(chain_to_sequence_dict.keys())[0]:
list(chain_to_sequence_dict.keys())}

            output_prefix_path = os.path.join(tempdir, f'cluster_output')
            mmseqs_command = f'taskset -c {cpu_region} mmseqs easy-cluster
{fasta_path} {output_prefix_path} {os.path.join(tempdir, "tmp")} --min-seq-id
0.8 -c 0.8 --cov-mode 5 --cluster-mode 2'
            subprocess.run(mmseqs_command, shell=True,
stdout=subprocess.DEVNULL)
            chain_to_sequence_dict = pd.read_csv(output_prefix_path +
'_cluster.tsv', sep='\t', header=None, names=['cluster_representative',
'chain'])
            return
invert_dict(chain_to_sequence_dict.set_index('chain').to_dict()['cluster_repr
esentative'])
        except:
            print(f'Failed to run {cluster}')
            return {list(chain_to_sequence_dict.keys())[0]:
list(chain_to_sequence_dict.keys())}

def worker_submit(tup):
```

```
    cluster, cpu_id = tup
    # Remap the chain to sequence.
    all_chain_sequences = {chain: all_sequences[chain] for chain in
cluster_to_chains[cluster]}
    sub_clusters = run_mmseqs(cluster, all_chain_sequences, cpu_id)
    return cluster, sub_clusters

clusters_to_subcluster_data = {}
with multiprocess.Pool(num_workers) as p:
    for cluster, subclusters in tqdm(p.imap_unordered(worker_submit, [(x,
cpu_regions[idx % len(cpu_regions)]) for idx, x in
enumerate(cluster_to_chains.keys())]), total=len(cluster_to_chains)):
        clusters_to_subcluster_data[cluster] = subclusters
```

Supplementary Figures

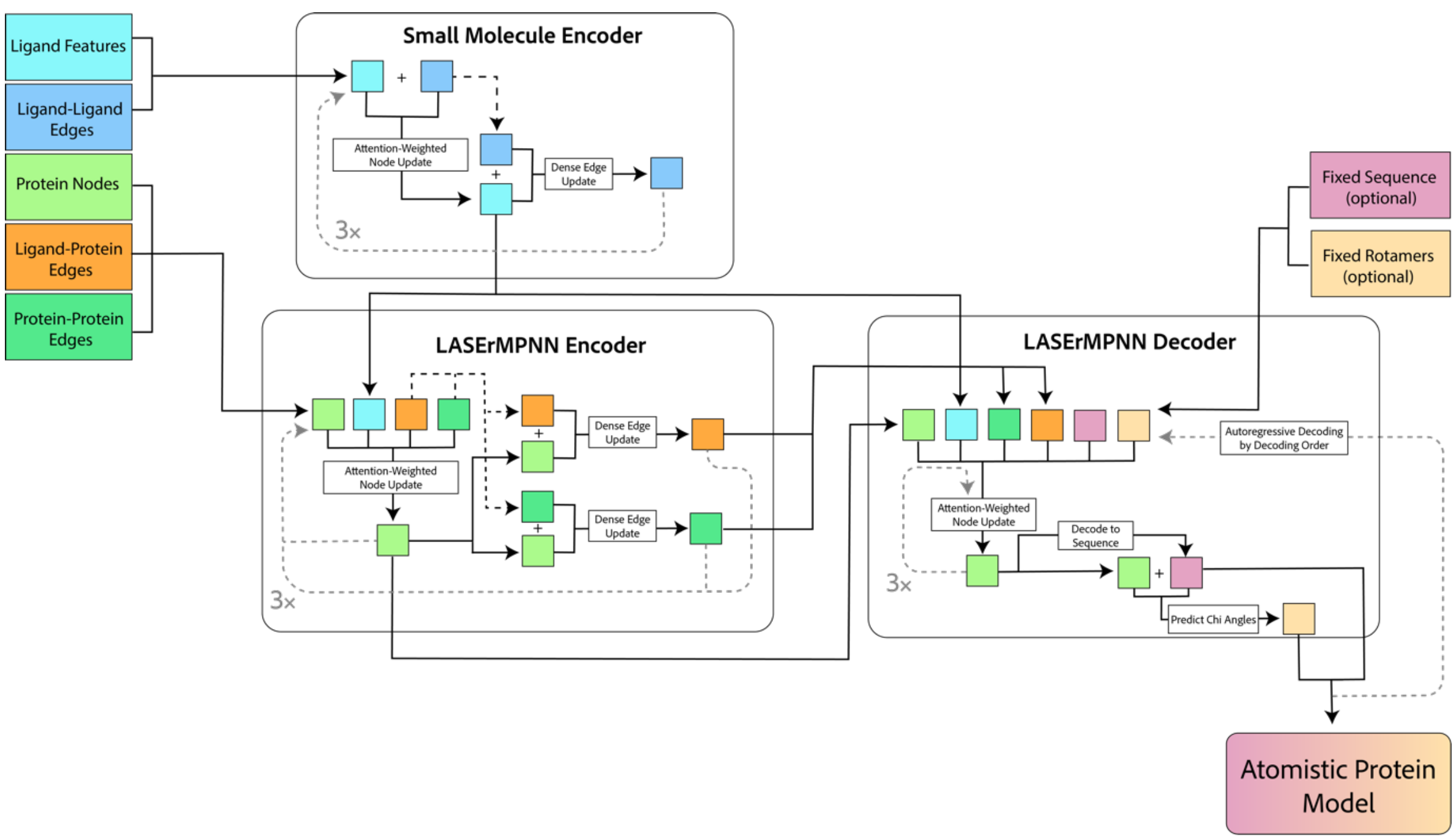

Supplementary Fig. 1. Block diagram of information flow through the LASErMPNN architecture. The LASErMPNN small-molecule encoder is a distinct pre-trainable module used to generate per-atom embeddings of each ligand atom from the input ligand cartesian coordinates and atomic numbers. These embeddings are frozen and used as input to the LASErMPNN Encoder and Decoder modules, which use a heterograph message passing formulation to build up embeddings for protein nodes, which are initialized to zeros from edge distance information. These embeddings are decoded to both a choice of amino acid identity and sidechain dihedral angles.

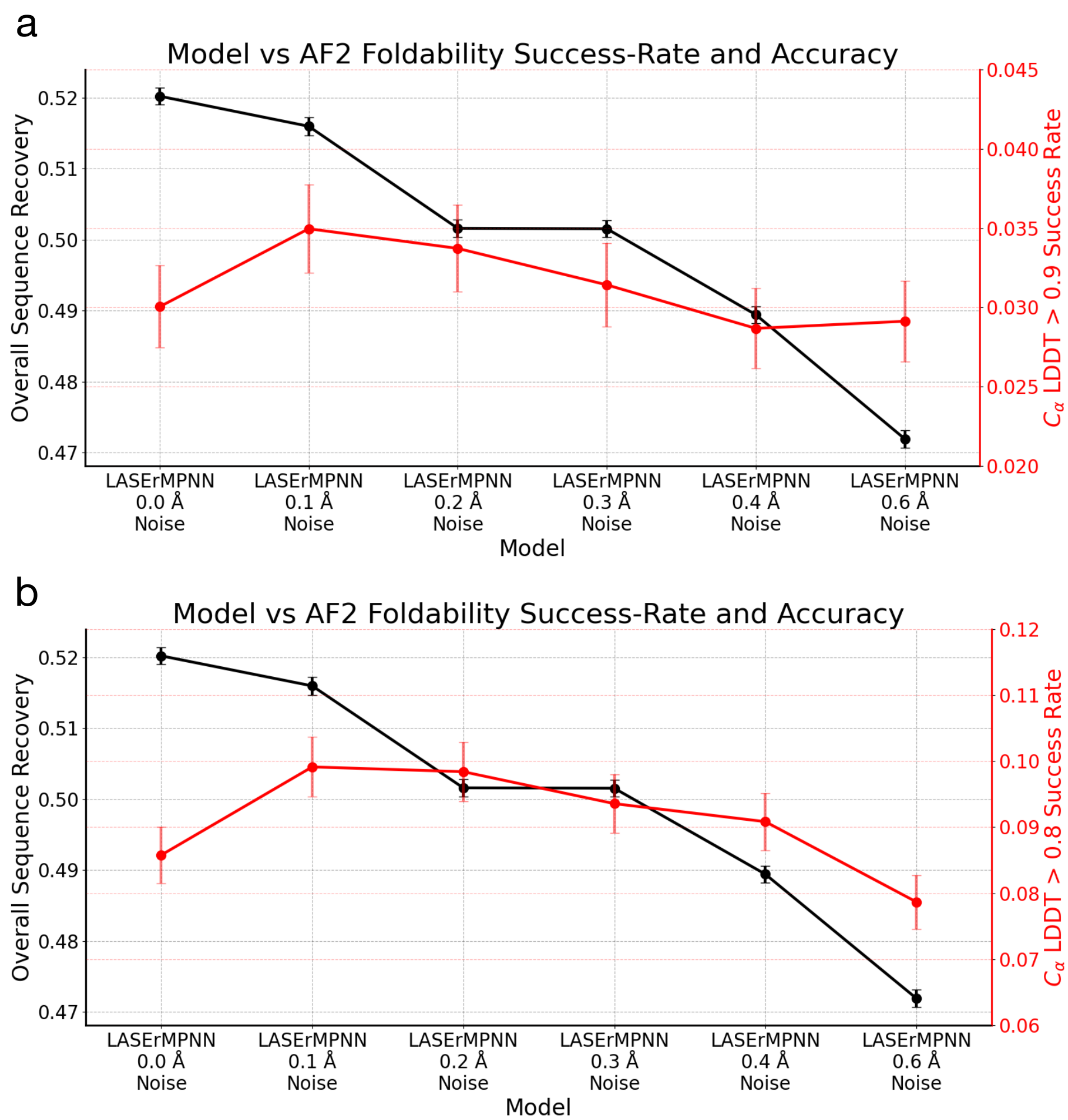

Supplementary Fig. 2. Using AF2 foldability to identify optimal amount of training noise. **a,** Similar to the approach taken with ProteinMPNN, we monitor, as a function of training noise level, the fraction of sequences designed by LASErMPNN that have Cα local distance difference test (LDDT) over a certain threshold. This process identified 0.1 Å as an optimum noise level on residue frames during training that maximizes overall sequence recovery and the fraction of a monomeric validation protein set (N=882) predicted by AF2 with Cα LDDT > 0.9. **b,** We repeat this analysis with a success rate threshold of 0.8 which also identifies 0.1 Å as an optimum maximizing sequence recovery and foldability. The presented values and uncertainties are means and standard deviations computed by bootstrapping (N=250,000) the respective metric uniformly over the 5 sampled sequences for each validation protein.

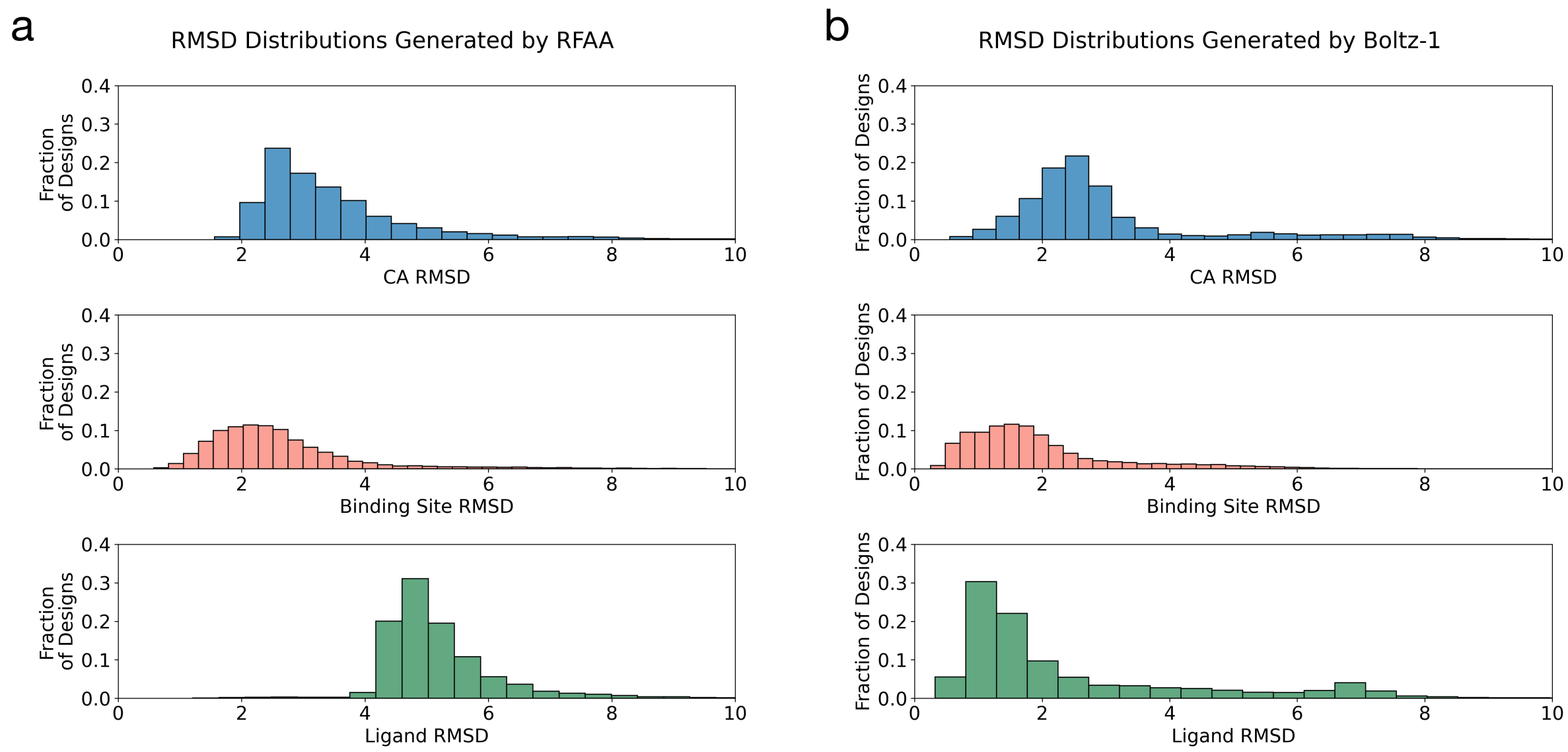

Supplementary Fig. 3. Distributions of various RMSD metrics for LASErMPNN-designed sequences of streptavidin-biotin (pdb 4jnj), using predicted protein–ligand co-structures from RFAA or Boltz-1. The same designed sequences (N=10,000) were folded using RFAA (**a**) and Boltz-1 (**b**). CA RMSD refers to the RMSD of all protein backbone Cα atoms after superposition onto the input 4jnj structure. Binding Site RMSD refers to the RMSD of protein backbone Cα atoms of residues with heavy atoms within 5.0 Å of biotin in the input crystal structure. Ligand RMSD refers to RMSD taken over the ligand heavy atoms after superposition on binding-site residues. There was a 65% success rate for Boltz-1 vs 0.4% for RFAA; protein Cα and ligand heavy-atom RMSD < 2.5 Å.

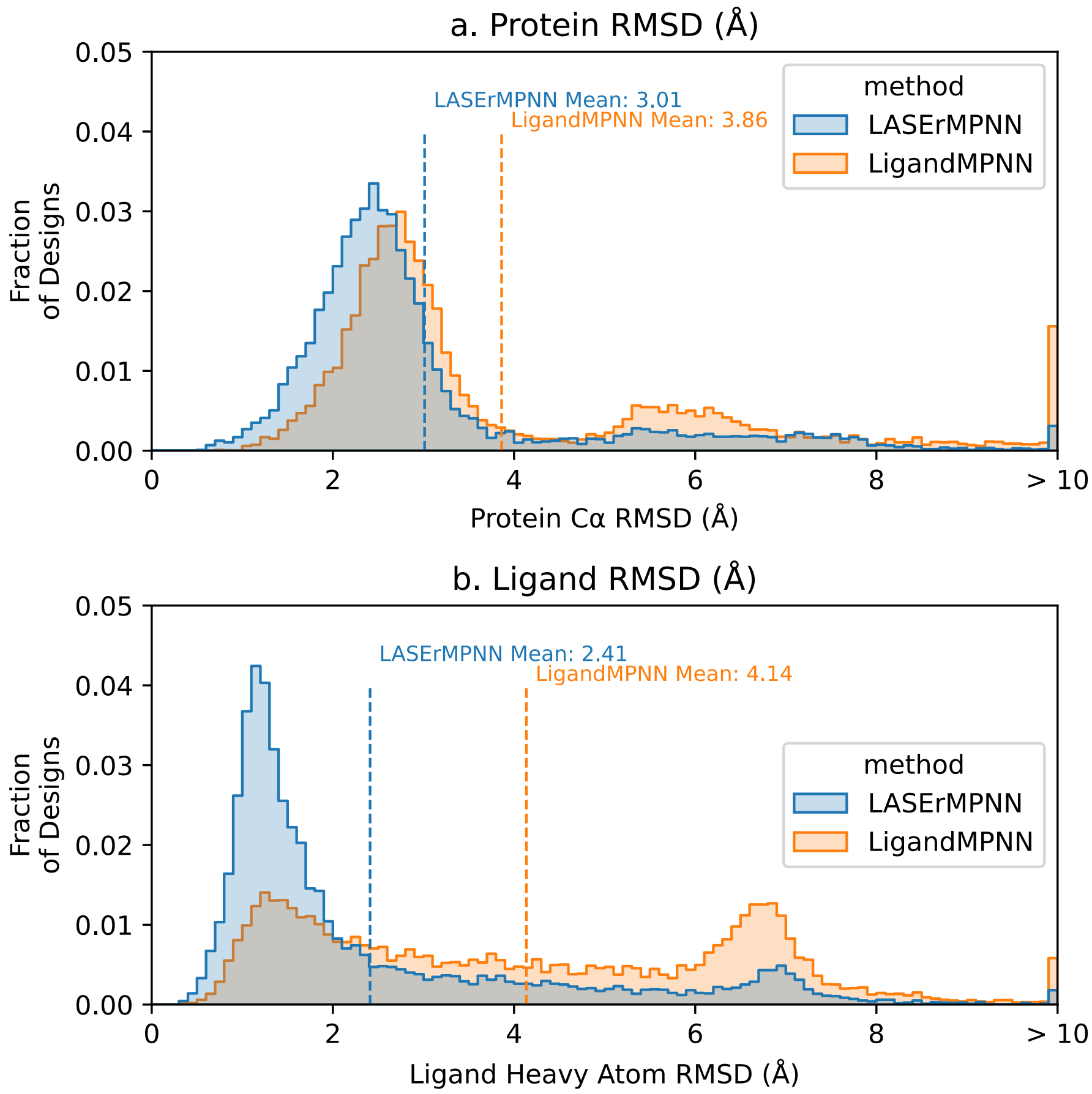

Supplementary Fig. 4. Co-structure predictions of sequences designed by LASErMPNN more closely resemble the monomeric streptavidin structure than those from LigandMPNN. We used Boltz-1 to predict co-structures of sequences designed by LASErMPNN and LigandMPNN using the monomeric streptavidin structure from PDB 4jnj as input. Designs from LASErMPNN show lower protein Cα RMSD (top) and lower biotin heavy-atom RMSD (bottom) to the 4jnj crystal structure than those from LigandMPNN (retrained on the same streptavidin holdout dataset as LASErMPNN). We sampled 10,000 sequences using each model at overall temperature 0.1 and binding-site temperature 1.0 using the backbone and biotin ligand coordinates from 4jnj as input. Specifically, we designed 2,500 sequences for each biological assembly (monomer) in PDB 4jnj. Co-structures for each sequence were predicted using Boltz-1 in single-sequence mode with default parameters and inference potentials. Vertical dashed lines denote distribution means. Considering protein Cα and ligand heavy-atom RMSD < 2.5 Å, LASErMPNN designs had a 65% success rate *vs* a 13% success rate for LigandMPNN designs.

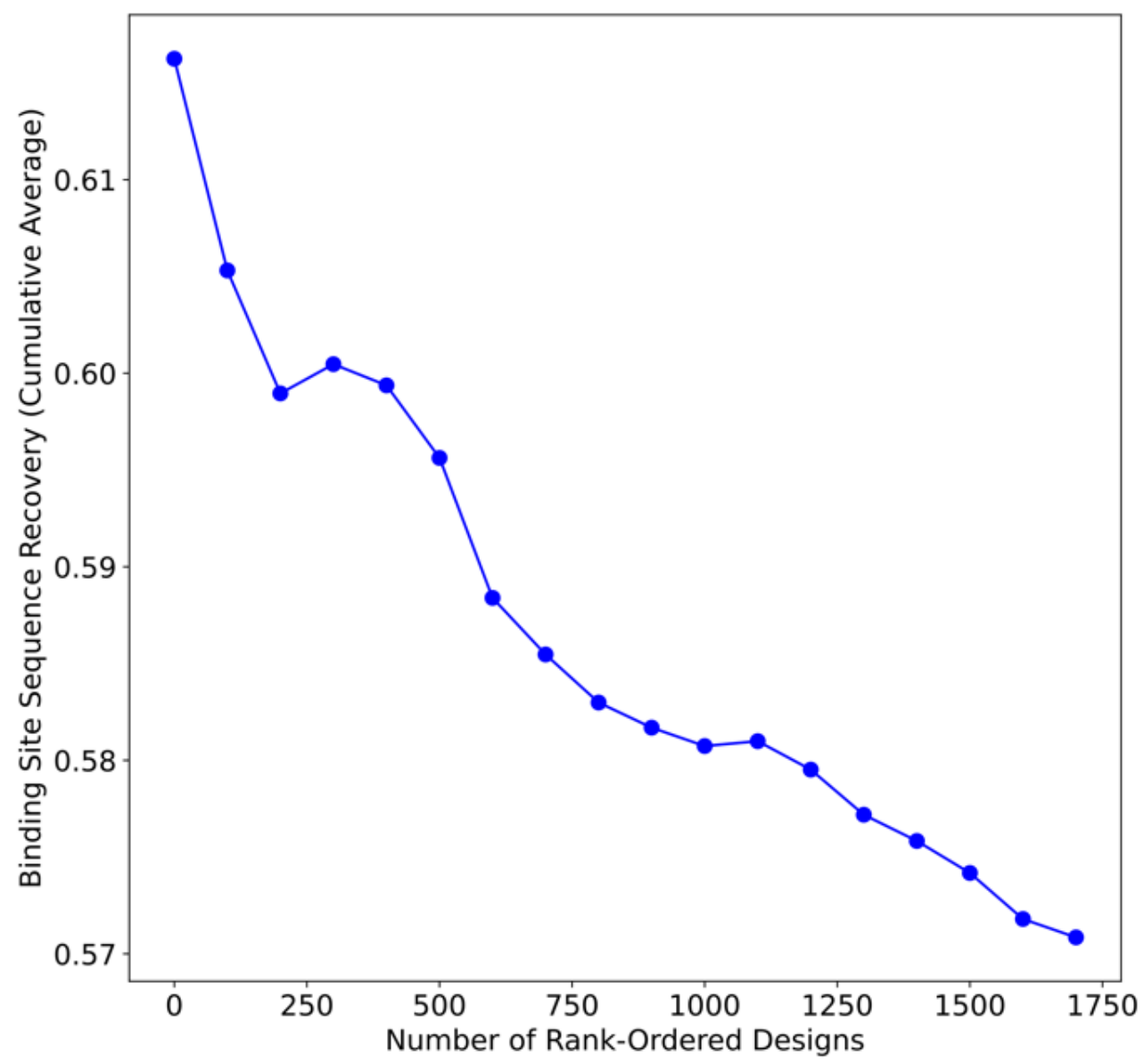

Supplementary Fig. 5. Binding-site sequence recovery is inversely correlated with the number of buried, non-H-bonded, polar atoms. Data shown is an analysis from 10,000 designed sequences of monomeric streptavidin and biotin complex (pdb 4jnj). Structures were predicted by Boltz-1 for each of the sequences designed by LASErMPNN, which were then energy-minimized (backbone and sidechains) using Rosetta (Ref15 energy function). We then computed the number of buried, non-H-bonded ("unsatisfied") polar atoms (BUNs) of the ligand and first-shell interacting residues. We sorted the designs by a formula that is 2x first-shell BUNs + 1x ligand BUNs. Then we compute a cumulative average of binding-site sequence recovery after inclusion of the next-best (by BUNs) 100 designs (circles). The plot shows that average binding-site sequence recovery is highest for the top-ranked designs by BUNs and decreases as number of BUNs increases.

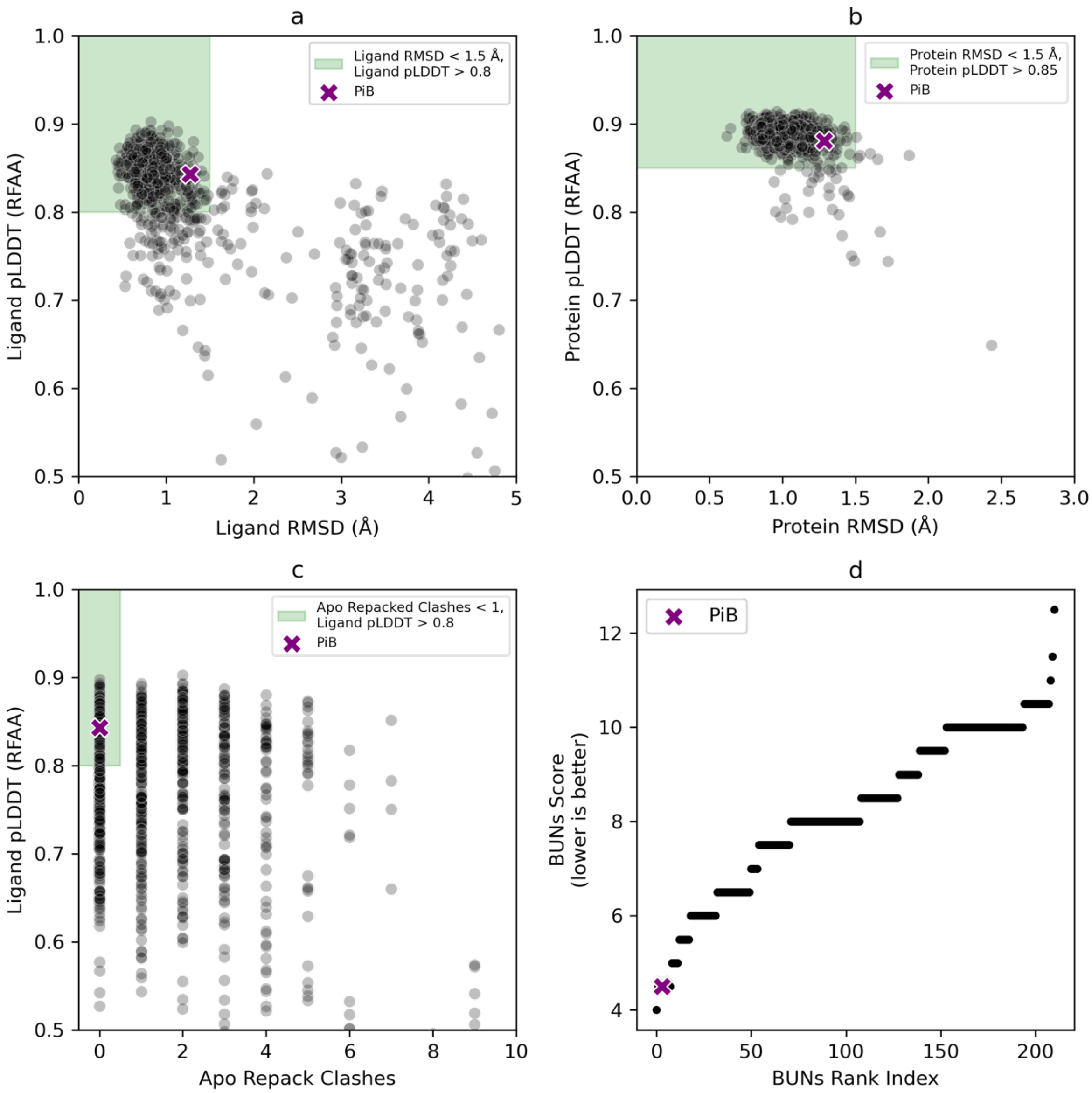

Supplementary Fig. 6. Filtering and ranking protocol of LASErMPNN-designed sequences ranks PiB favorably. We used LASErMPNN to design 1000 sequences for the rucaparib-bound PiB helical bundle (pdb 8tn6). The sequence of PiB (< 5 nM dissociation constant with rucaparib) was added to the set of LASErMPNN-design sequences and is denoted with a purple "x" in each plot. Sequences were filtered by **a,** ligand heavy-atom RMSD < 1.5 Å and ligand pLDDT > 0.8, **b,** protein Cα RMSD < 1.5 Å and protein Cα pLDDT > 0.85. **c,** We performed a calculation that approximates the protein's preorganization for binding, by computing sidechain clashes with the ligand after binding-site sidechains were repacked in the absence of the ligand. We selected designs with no clashes. **d,** We ranked remaining designs by BUNs Score (2x ligand BUNs + 1x protein BUNs). The PiB sequence ranks 4th out of the total 1001 sequences (using a subsequent ranking by ligand pLDDT to break degeneracy of designs with equal BUNs Score).

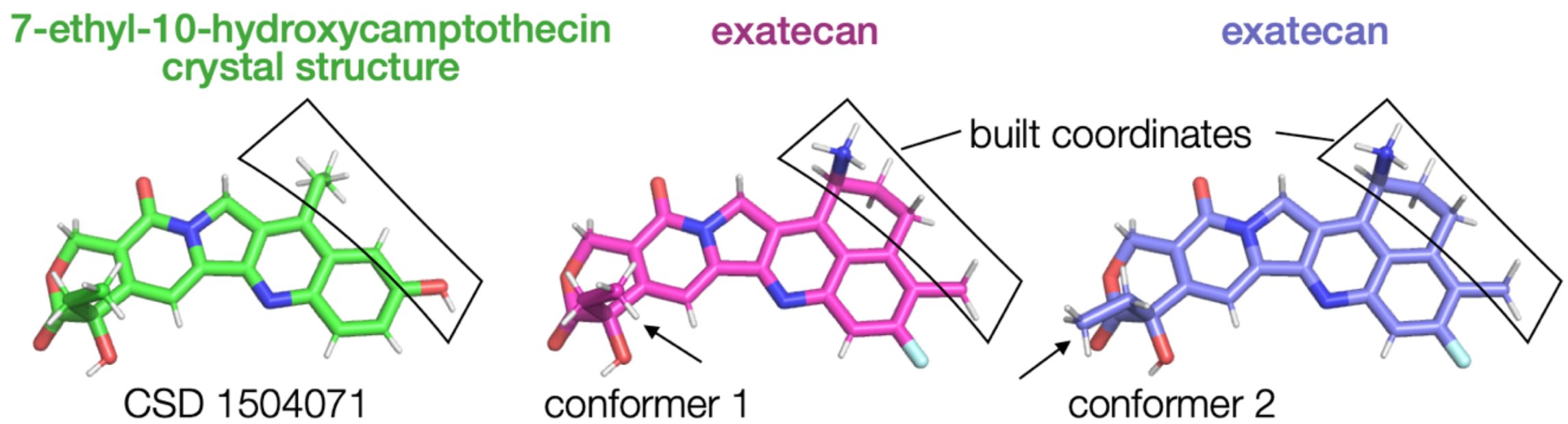

Supplementary Fig. 7. Building conformers of exatecan. A crystal structure of 7-ethyl-10-hydroxycamptothecin (Cambridge Structural Database, CSD, deposition number 1504071) was used as a starting point for building exatecan conformers. We kept the coordinates from the crystal structure that were shared with exatecan and used the program Avogadro to add atoms specific to exatecan and make new bonds (see boxes). The two conformers of exatecan differed by a rotation about an ethyl torsional angle (black arrow), using dihedral angles observed in small-molecule crystal structures for camptothecin derivatives.

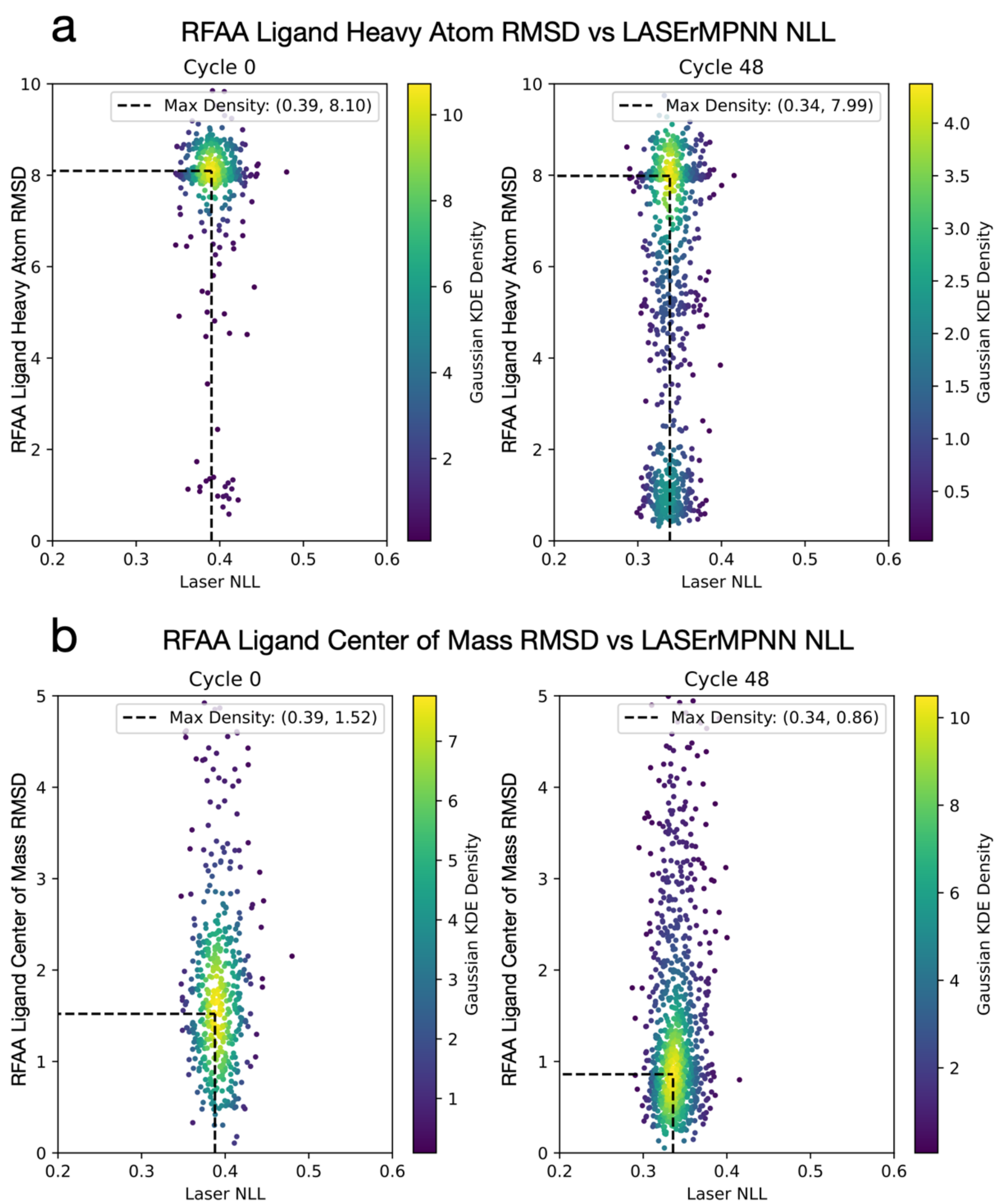

Supplementary Fig. 8. NISE improves the self-consistency of the predicted ligand and the negative log likelihood (NLL) of designed sequences. We ran the NISE protocol on the CR4 pose with exatecan (all sequence information was removed). We plot ligand heavy-atom and center-of-mass RMSD vs NLL of the designed sequences, computed for the first and a later iteration of NISE. NISE improves both the ligand RMSD and NLL, shown by the distribution shift to lower values for each variable at the later iteration (right side). **a,** Ligand heavy atom RMSD (Å) between input to LASErMPNN and RFAA-predicted structure vs NLL of LASErMPNN designed sequences for first NISE iteration and the 48th NISE iteration. **b,** Ligand center of mass RMSD between input to LASErMPNN and RFAA predicted structure vs NLL of LASErMPNN designed sequences for first NISE iteration and 48th NISE iteration. Throughout the trajectory, RFAA is generally able to place exatecan's center of mass within 2.0 Å of the input but struggles to predict the correct orientation of exatecan (high heavy-atom RMSD). However, the NISE protocol is able to increase the number of RFAA-predicted structures with the correct ligand orientation (low ligand heavy-atom RMSD).

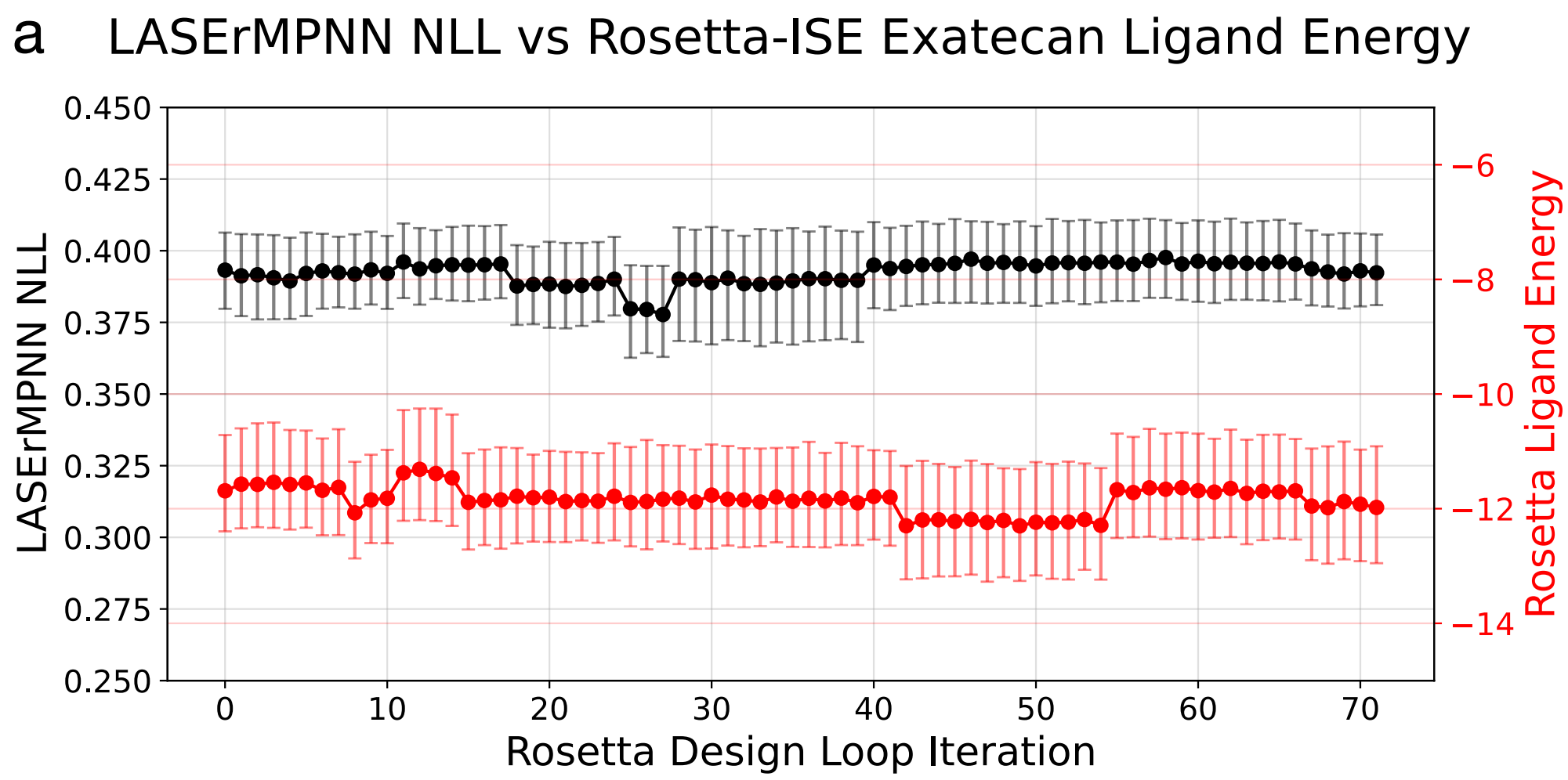

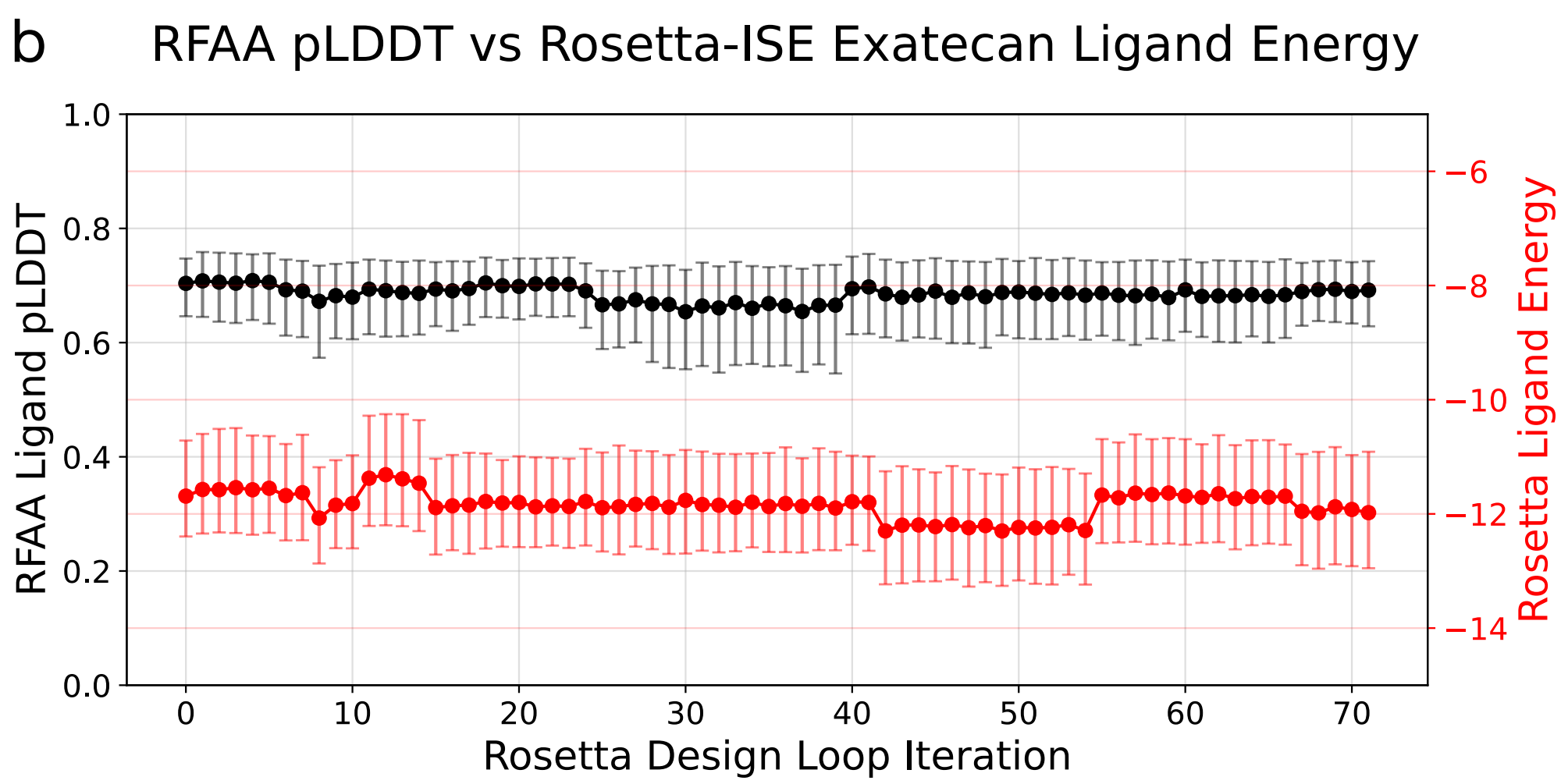

Supplementary Fig. 9. Energy-based selection–expansion is unable to optimize sequence NLL or ligand pLDDT. We ran an iterative selection–expansion (ISE) protocol using Rosetta instead of RFAA to sample new backbone–ligand co-structures from the LASErMPNN-designed sequences. We selected the top 3 Rosetta-minimized backbone–ligand complexes by the Rosetta energy of the ligand. **a,** Median NLL of sequences and median Rosetta ligand energy as a function of ISE design cycle number. **b,** After we ran the entire trajectory (which did not use RFAA), we used RFAA to predict the structures of the sequences designed during each round. We plot the median ligand pLDDT from RFAA for these sequences and the median Rosetta ligand energy as a function of ISE design cycle number. Error bars represent first and third quartiles. N = 1500 sequences per loop iteration.

a
b
c
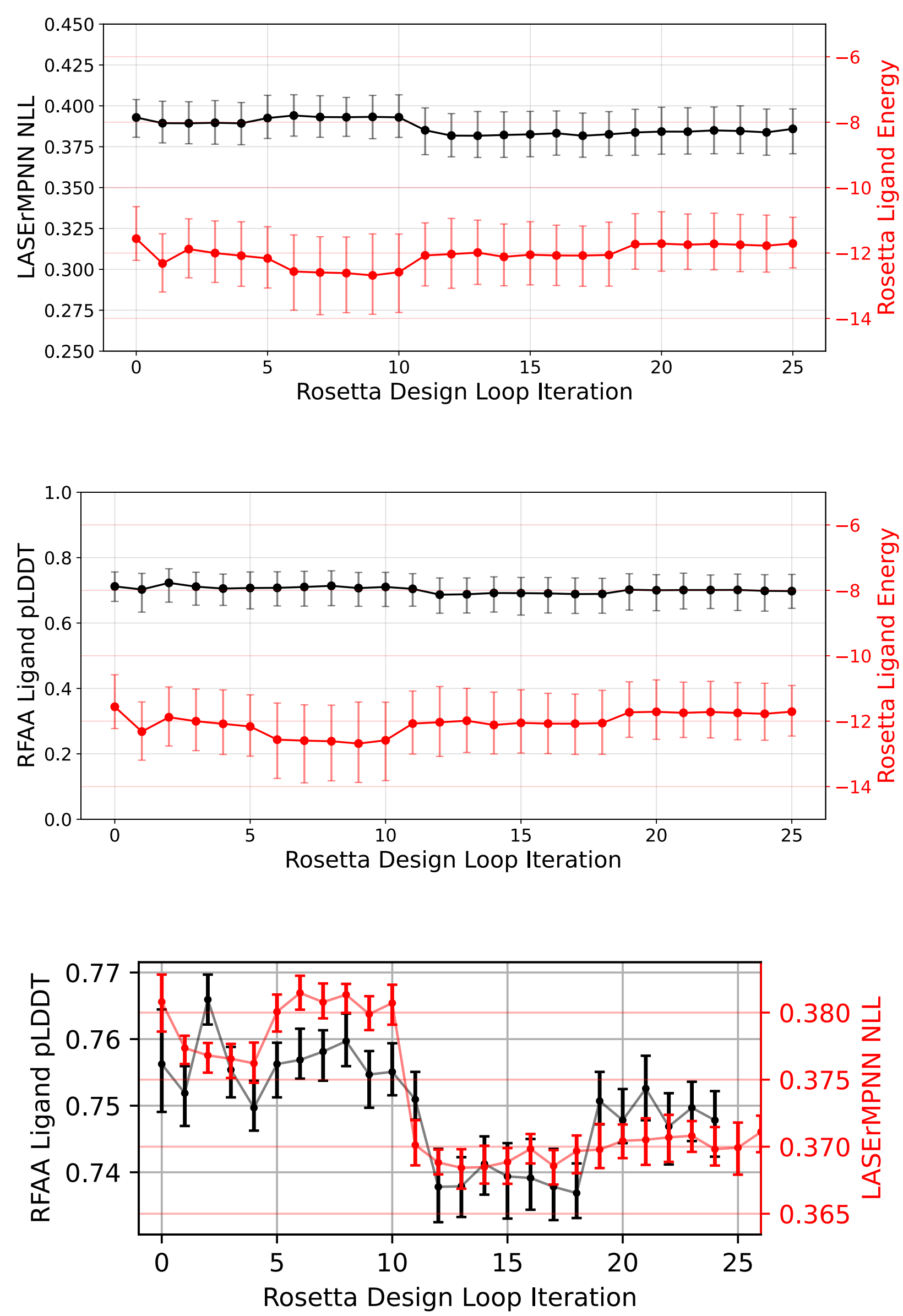

Supplementary Fig. 10. An energy-based iterative selection–expansion protocol using Rosetta does not optimize neural network confidence. We performed iterative selection–expansion starting from the same input backbone and exatecan location as in Fig. 3c (input pose for NISE design of EPIC). We swapped out RFAA for energy minimization (of protein and ligand coordinates) with Rosetta (Ref15 energy function). **a, b,** Median values of LASErMPNN negative log likelihood (NLL) of designed sequences, ligand pLDDT from RFAA, and Rosetta ligand energy with error bars displaying the first and third quartiles for each iteration. **c,** Data points show the value of the third quartile for sampled pLDDT and first quartile for NLL, with error bars representing 95% confidence intervals for these values computed by bootstrapping (N=100,000) the sampled data for each iteration (see <u>Statistical Bootstrapping NISE Confidence Metrics for Uncertainty Quantification</u>).

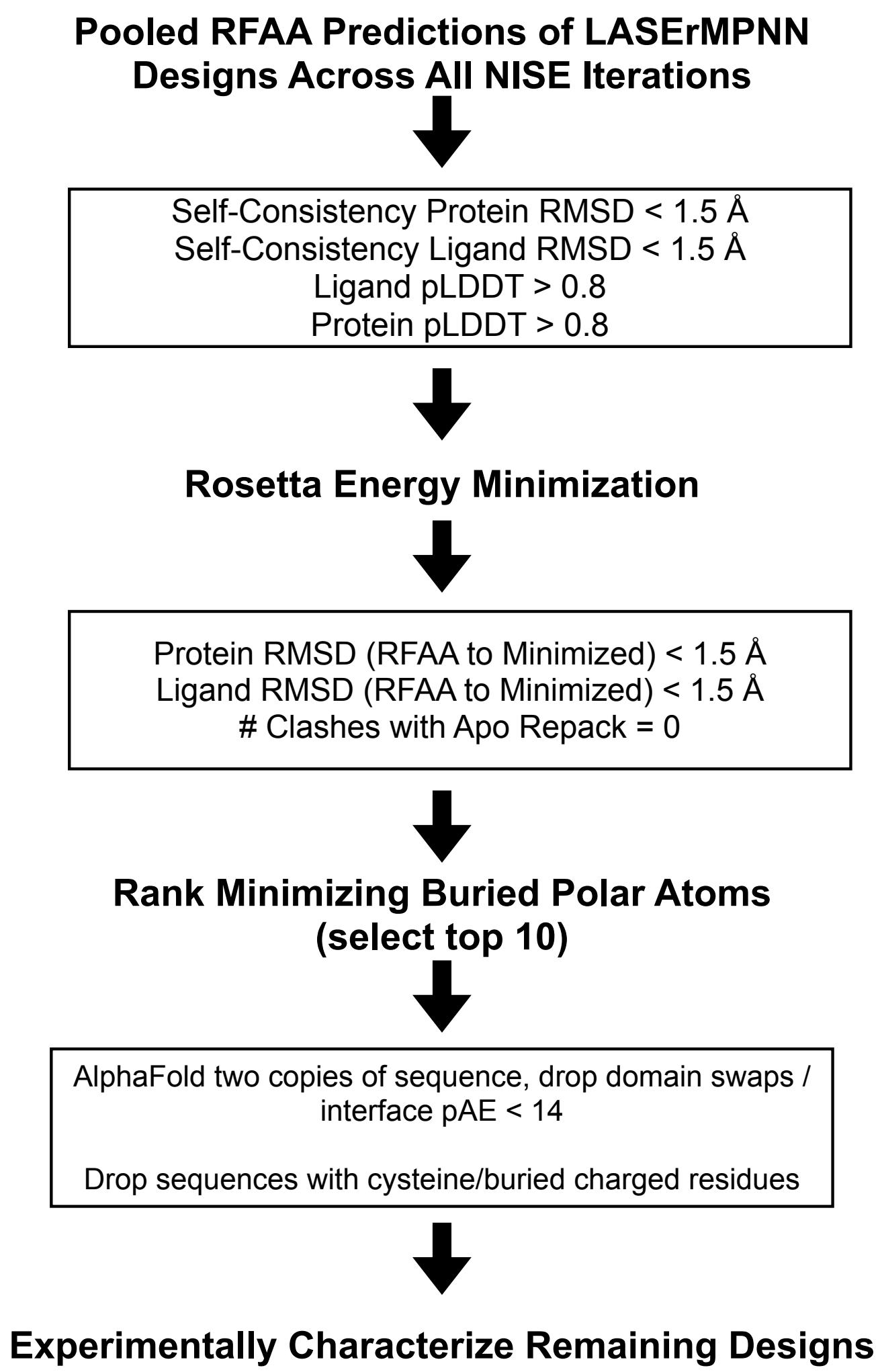

Supplementary Fig. 11. NISE filtering and ranking protocol for selecting exatecan-binding proteins for experimental characterization. We pooled the designed sequences across all NISE iterations and used the RFAA-predicted co-structures to filter out designs with low self-consistency or low confidence. The remaining sequences were energy minimized using Rosetta, and additional RMSD filters were applied that compared the structures before and after minimization. For remaining structures, we checked for preorganization by performing energy minimization of the protein in the absence of the ligand. We then re-introduce the ligand and check if any relaxed sidechains in the binding site now clash with the ligand. Sequences with no clashes were then ranked by the number of buried, non-H-bonded polar atoms of the ligand. The top 10 ranked designs were finally filtered to remove sequences with buried charged or cysteine residues and for domain-swapping or aggregation propensity using AlphaFold.

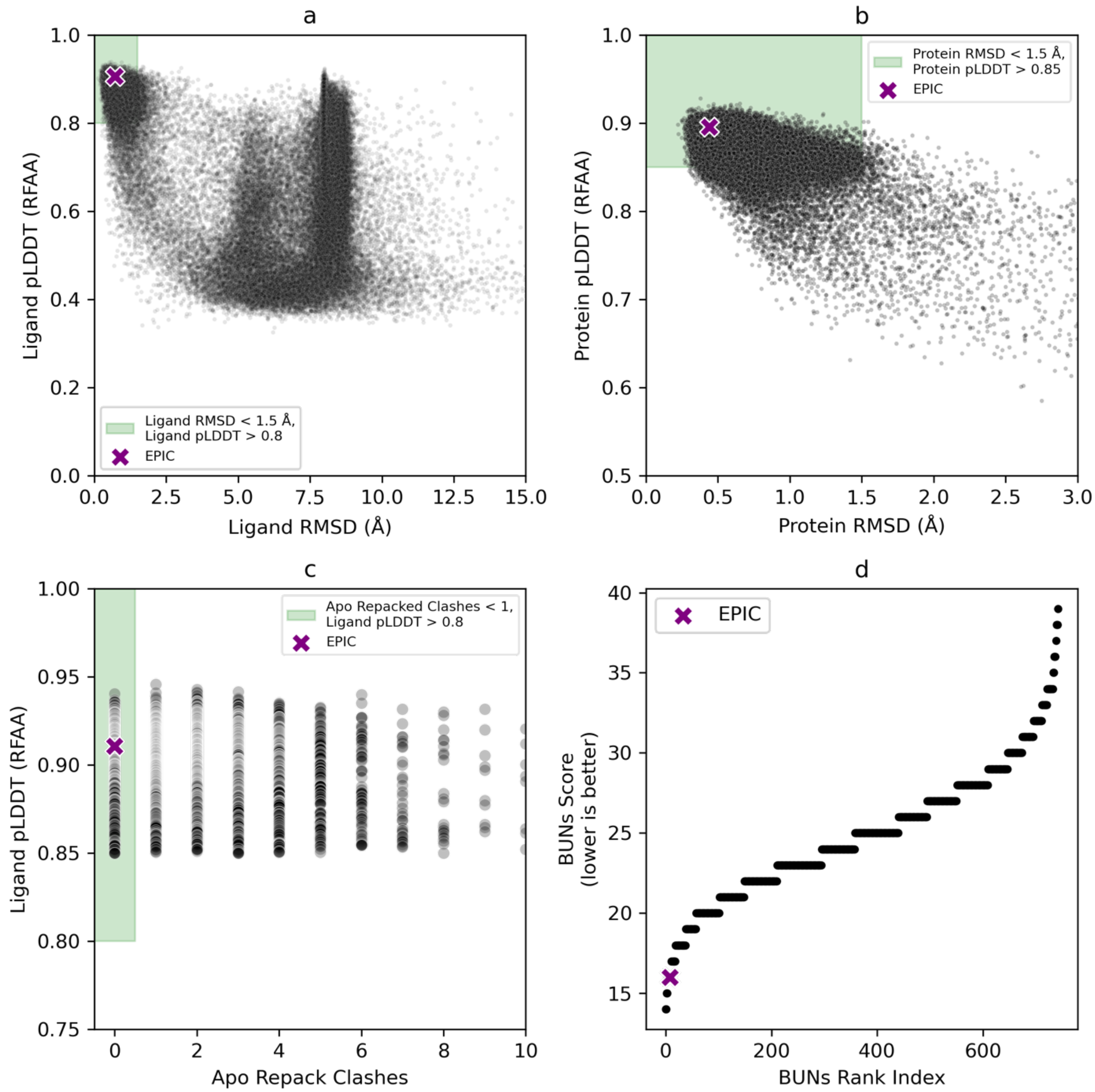

Supplementary Fig. 12. NISE filtering and ranking protocol for selection of exatecan binders. a, b, LASErMPNN-designed sequences were filtered by ligand heavy-atom and protein Cα self-consistency RMSD < 1.5 Å, ligand pLDDT > 0.8, and protein pLDDT > 0.85. **c**, We repacked the unbound proteins and then computed clashes with the re-introduced ligand, selecting designs with no clashes (green). **d**, We ranked remaining designs by the number of buried non-H-bonded polar atoms (BUNs) in the ligand, which places EPIC (purple "x") as 8th out of the total 72,501 sequences (using ligand pLDDT to break degeneracy of designed with equal BUNs).

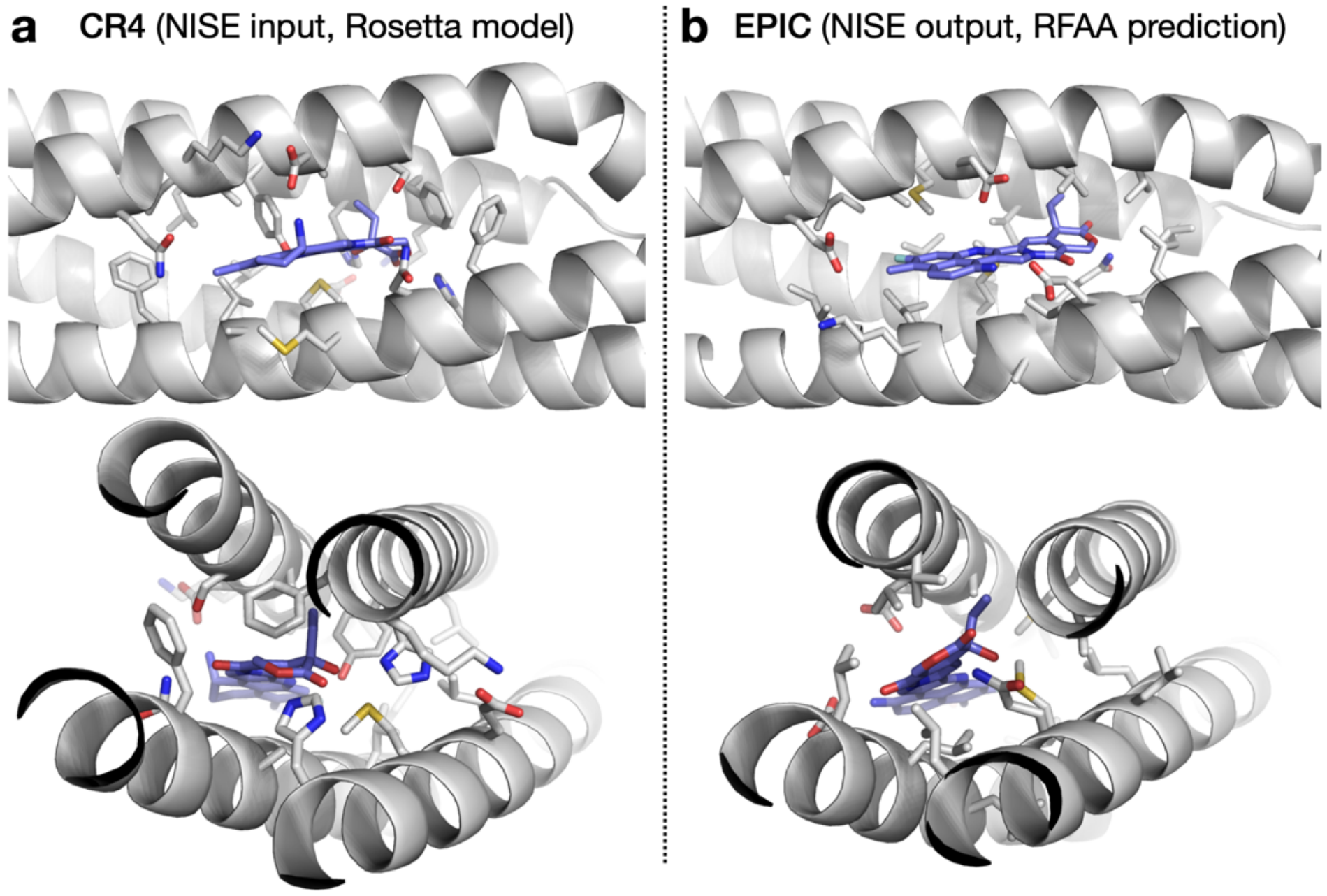

Supplementary Fig. 13. Comparison of traditional design model (COMBS and Rosetta) with output from NISE (EPIC model). **a**, Rosetta model of an exatecan-binding protein, CR4, that was designed with a traditional approach using COMBS and Rosetta. This model, after its sequence was removed, was input (backbone and ligand coordinates only) to the NISE protocol that lead to EPIC. Exatecan is shown in blue. Sidechains near the binding site are shown as stick representation. CR4 is designed to engage with exatecan using two polar His residues and Tyr, Asn, Ser, Asp residues. Aromatic Phe residues also surround the ligand. **b**, RFAA-predicted exatecan-protein co-structure of EPIC, which was designed via NISE (iterative selection-expansion using LASErMPNN and RFAA). LASErMPNN designed a binding site of EPIC that is less polar than CR4 but still engages the ligand with H-bonds from Gln51 and Asp132. Packing around the ligand is more extensive than in CR4 and uses smaller aliphatic sidechains such as Val, Ile, and Leu. The structures were aligned via their Cα coordinates (1.4 Å RMSD), and the views shown are from the same vantage point after alignment.

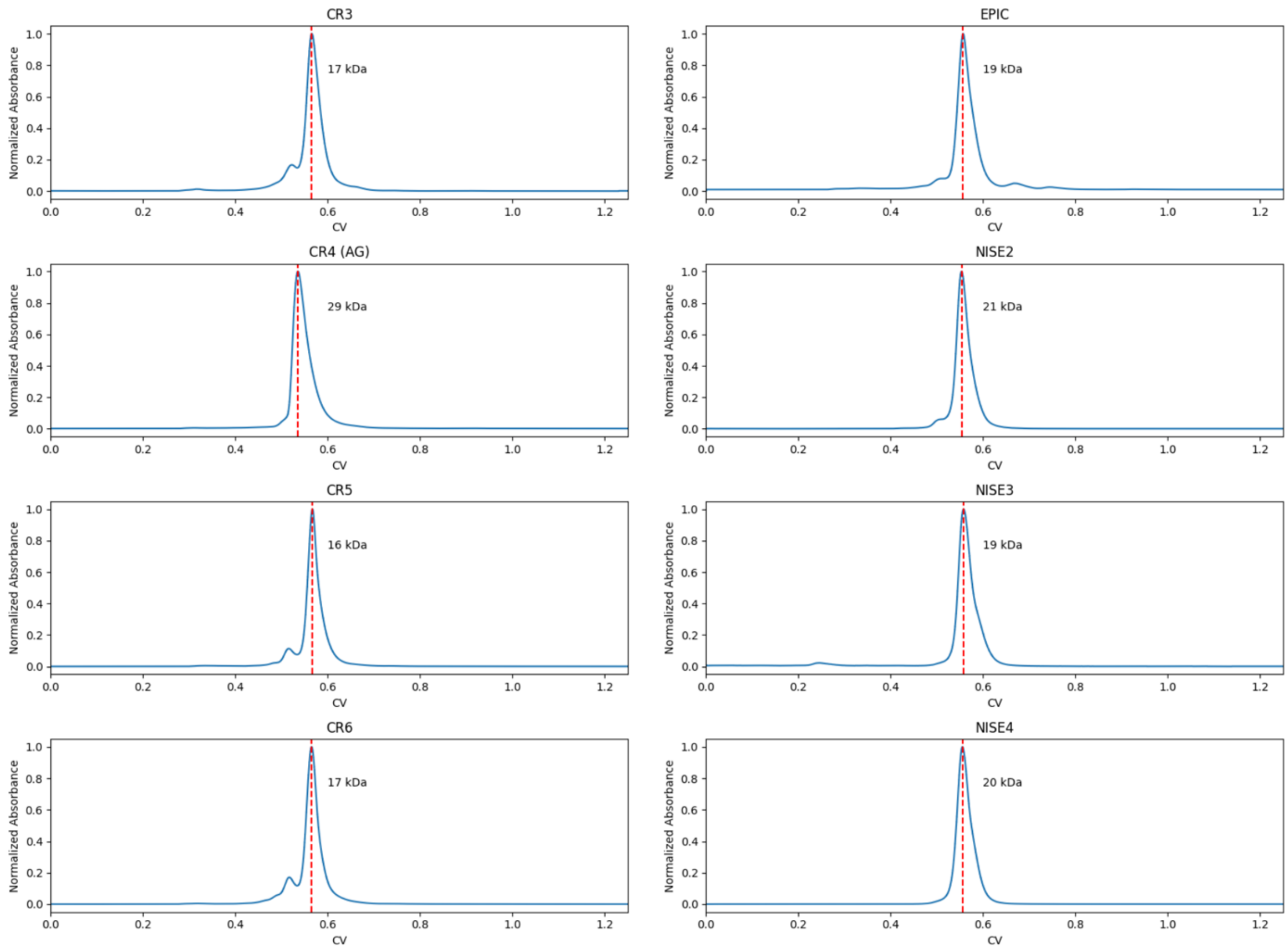

Supplementary Fig. 14. Size exclusion chromatograms of various exatecan-binder designs. Purified COMBS/Rosetta- (left) and NISE-generated (right) protein constructs were injected on a Biorad NGC system at a concentration of > 100 µM and run on a Biorad 650 ENrich chromatography column in PBS pH 7.4 buffer. The proteins run slightly larger than the expected molecular weight (~17 kDa), but the results are consistent with monomers.

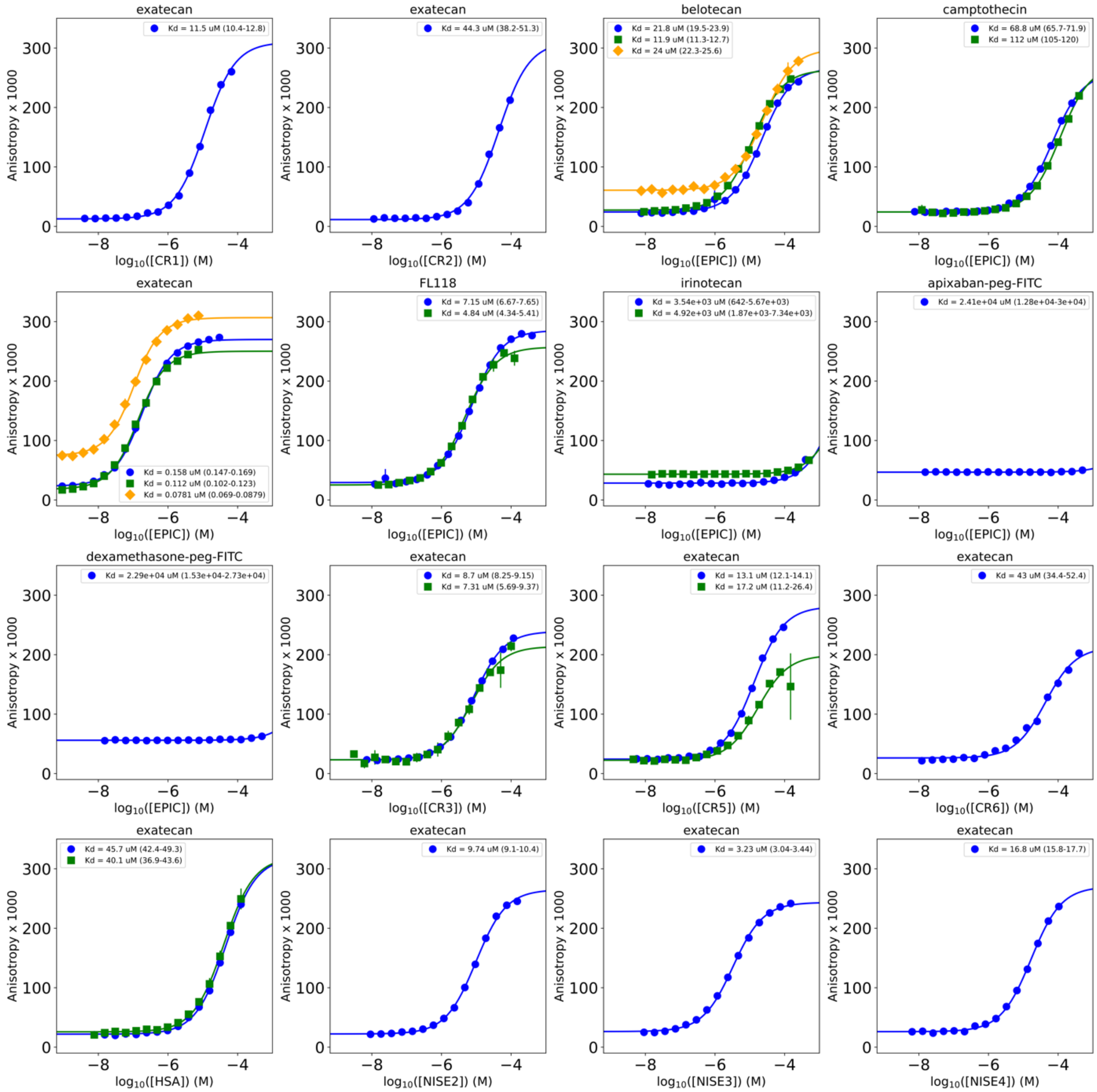

Supplementary Fig. 15. Fluorescence anisotropy data and fits to a single-site binding model. Each plot shows an individual protein (labeled on x-axis) and an individual ligand (plot title). Multiple curves are biological replicates. The plot legends show each K_d (units of μM) fit from a single-site model. The K_d shown is the mean of 1000 fits performed with bootstrapping of the optimal fit residuals. The parentheses show the lower and upper bounds of 95% confidence intervals derived from the bootstrapped fits. Error bars on the points denote standard deviations from technical replicates. Each experiment was performed with 3 technical replicates. Any systematic baseline shifts in anisotropy values between experiments are a result of slightly different gain settings on the plate reader. See Supp. Table 1 to match protein names of designed proteins with sequences. HSA is human serum albumin. See Supp. Table 6 to match ligand names to chemical structures. Buffer conditions: PBS, pH 7.4, 0.1% w/v PEG-8000.

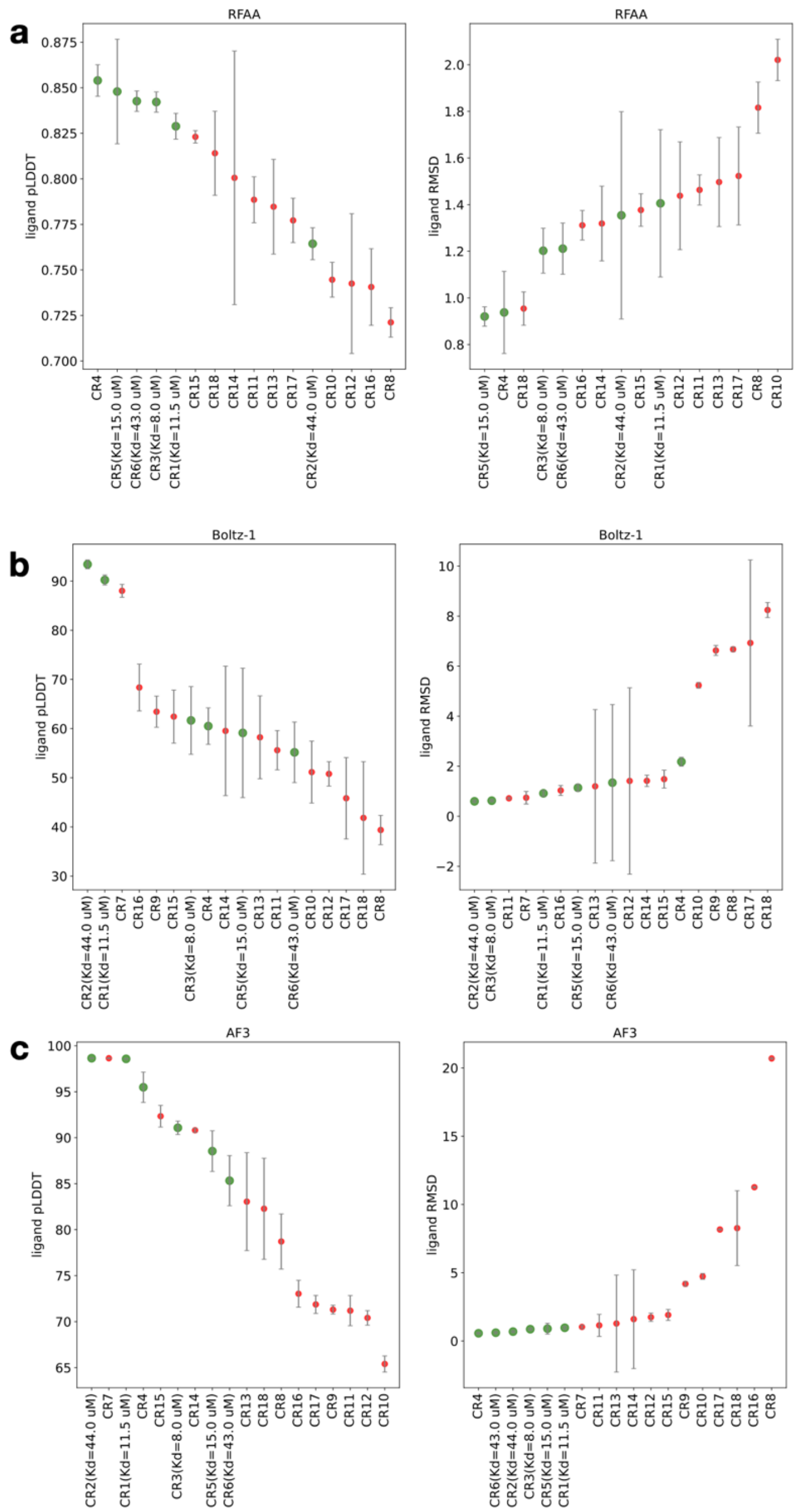

Supplementary Fig. 16. Ligand confidence and self-consistency are correlated with experimental outcomes of binding. Designs are sorted by mean ligand pLDDT (left) and ligand heavy-atom RMSD (right, RMSD with respect to the COMBS/Rosetta model after aligning on binding-site residues by Cα atoms) using output from **a**, RoseTTAFold-All Atom (RFAA), **b**, Boltz-1, and **c**, AlphaFold3 (AF3). We used sequences computed from COMBS and Rosetta that we experimentally tested for binding to exatecan. The designs are labeled on the x-axis (along with any measured dissociation constants). Markers for designs that bound exatecan are colored green and non-binders are colored red. Error bars denote the standard deviation of results computed across 5 different seeds. RFAA shows a clear correlation between ligand pLDDT and binding and a slightly weaker correlation for ligand RMSD. Boltz-1 shows the weakest correlations but both variables show some enrichment for binders. AF3 results correlate strongly with binding, with ligand RMSD particularly successful at ranking binders over non-binders.

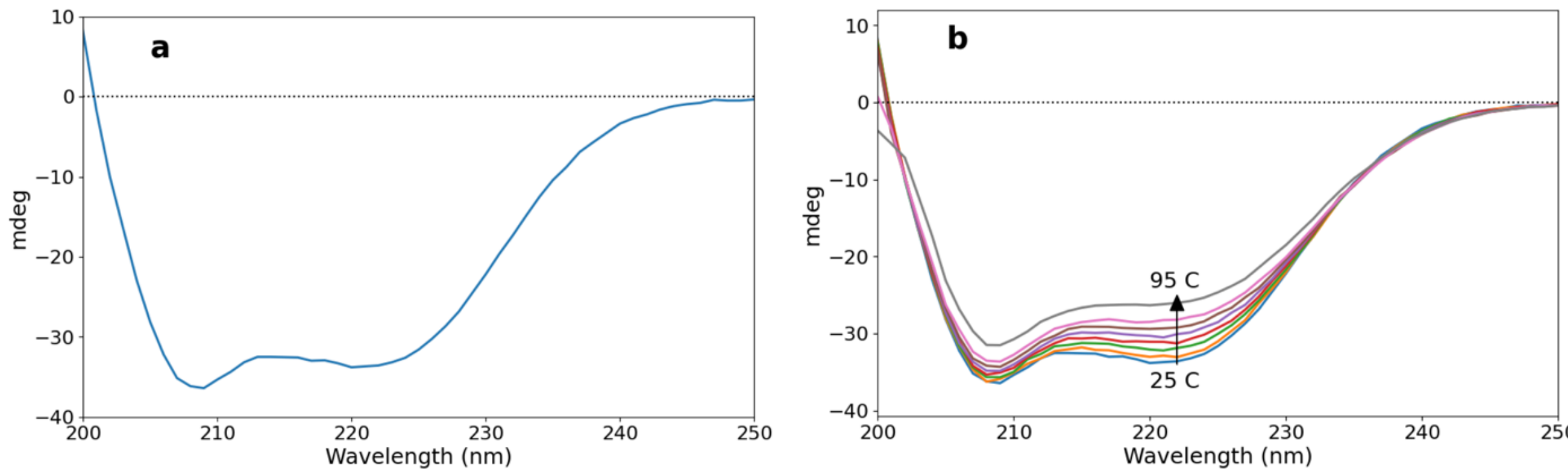

Supplementary Fig. 17. Determination of secondary structure content and thermal stability of EPIC by circular dichroism (CD) spectroscopy. CD spectra of ligand-free EPIC collected at **a,** 25 °C or **b,** varying temperatures ranging from 25 to 95 °C. No melting transition was observed. Conditions: PBS buffer, pH 7.4, protein concentration of 0.1 mg/mL (5.93 µM).

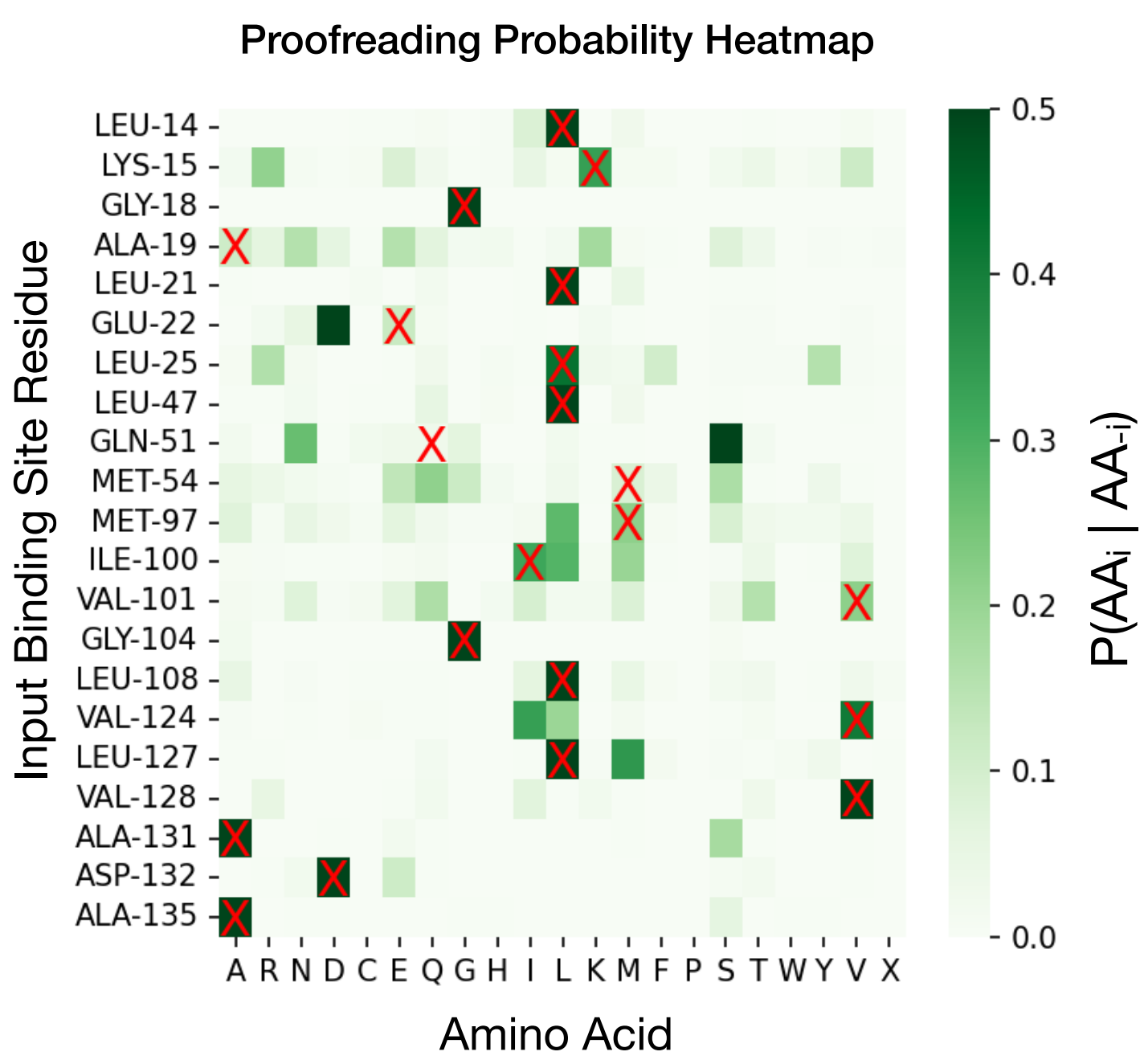

Supplementary Fig. 18. Heatmap of probabilities after proofreading of binding-site residues using the Amber-minimized RFAA prediction of exatecan-bound EPIC as the input structure to LASErMPNN. The residues in the original EPIC sequence are denoted with a red "X", and all residues with heavy atoms within 5.0 Å of ligand heavy atoms are shown. Proofreading identifies GLN-51 to N mutation and MET-97 to L mutation, as these mutants have higher probabilities than the originally designed residue. Color bar denotes probability of the amino acid at that position conditioned on the structure and identities of all other residues in the EPIC sequence, $P(AA_i \mid AA_{-i})$.

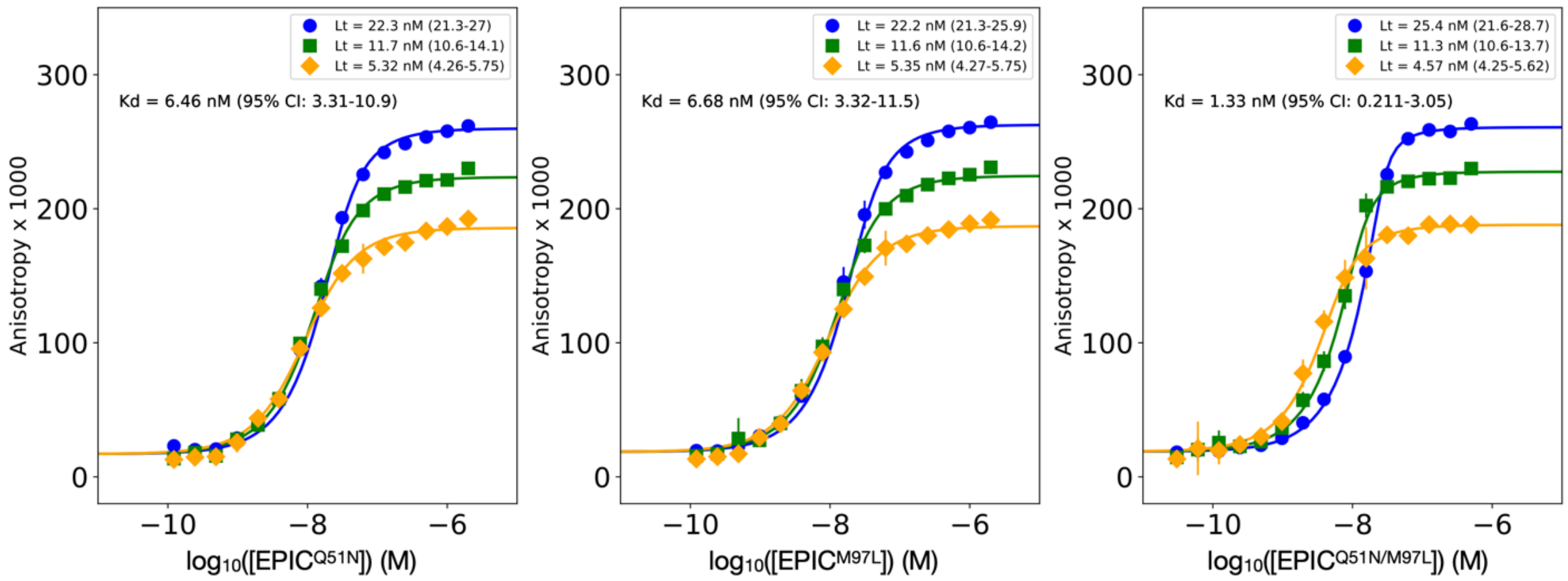

Supplementary Fig. 19. Global binding fits of EPIC mutants to exatecan. A single-site binding model with shared dissociation constant (K_d) was fit globally across three fluorescence anisotropy experiments with varying concentrations of exatecan ligand for each mutant protein (labeled on x-axis). Parameters fit locally to each trace were anisotropy maximum and total ligand concentration (Lt). The anisotropy baseline was fit globally per mutant. The per-mutant globally fit K_d is shown in the plot. K_d (units of nM) is the mean from 1000 bootstrapped fits, shown with 95% confidence intervals in parentheses. The fits of total ligand concentration are shown in the plot legend with 95% confidence intervals in parentheses (derived from 1000 bootstrapped fits). Lt was allowed to vary by 15% of the known ligand concentration to account for experimental errors (shown in insets). Note that the anisotropy maxima differ across ligand concentrations because the anisotropy data are not baseline corrected, which should not affect the fit K_d values. Buffer conditions: PBS, pH 7.4, 0.1% w/v PEG-8000. K_d and error bars in Supp. Table 1 are mean and standard deviation of optimal fits for 3 biological replicates.

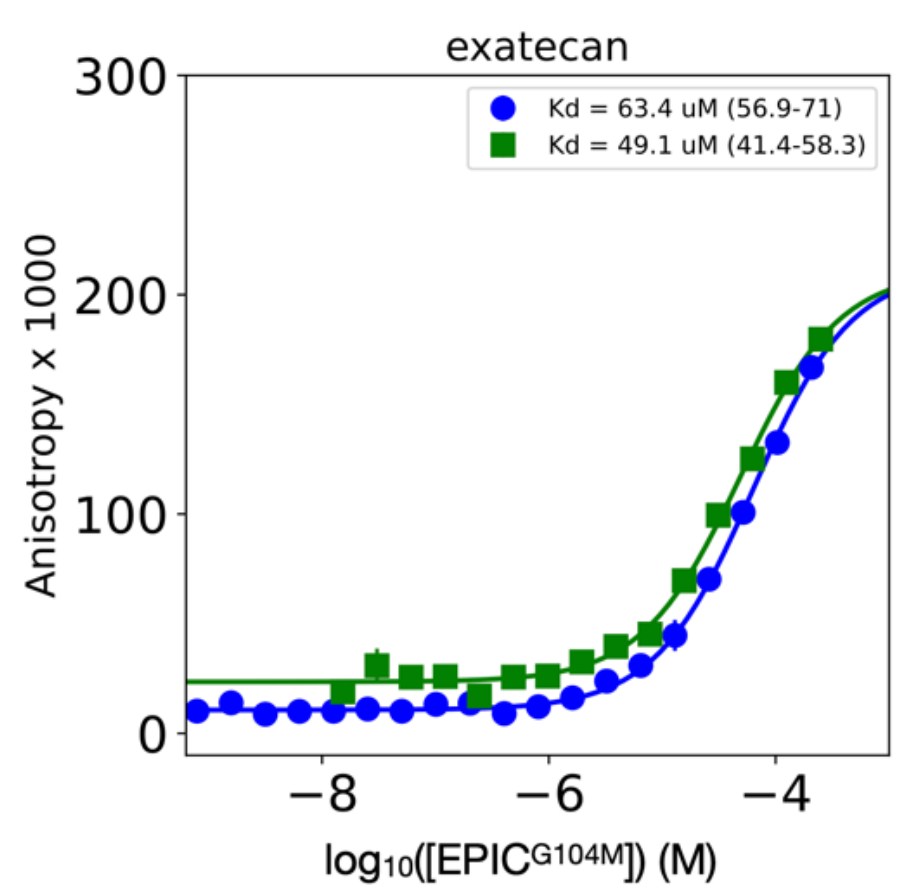

Supplementary Fig. 20. Fluorescence anisotropy data and fit to a single-site binding model for EPIC^{G104M} with exatecan. The K_d (units of μM) shown is the mean of 1000 fits performed with bootstrapping of the optimal fit residuals. The parentheses show the lower and upper bounds of 95% confidence intervals derived from the bootstrapped fits. The experiment was performed with 3 technical replicates. Buffer conditions: PBS, pH 7.4, 0.1% w/v PEG-8000.

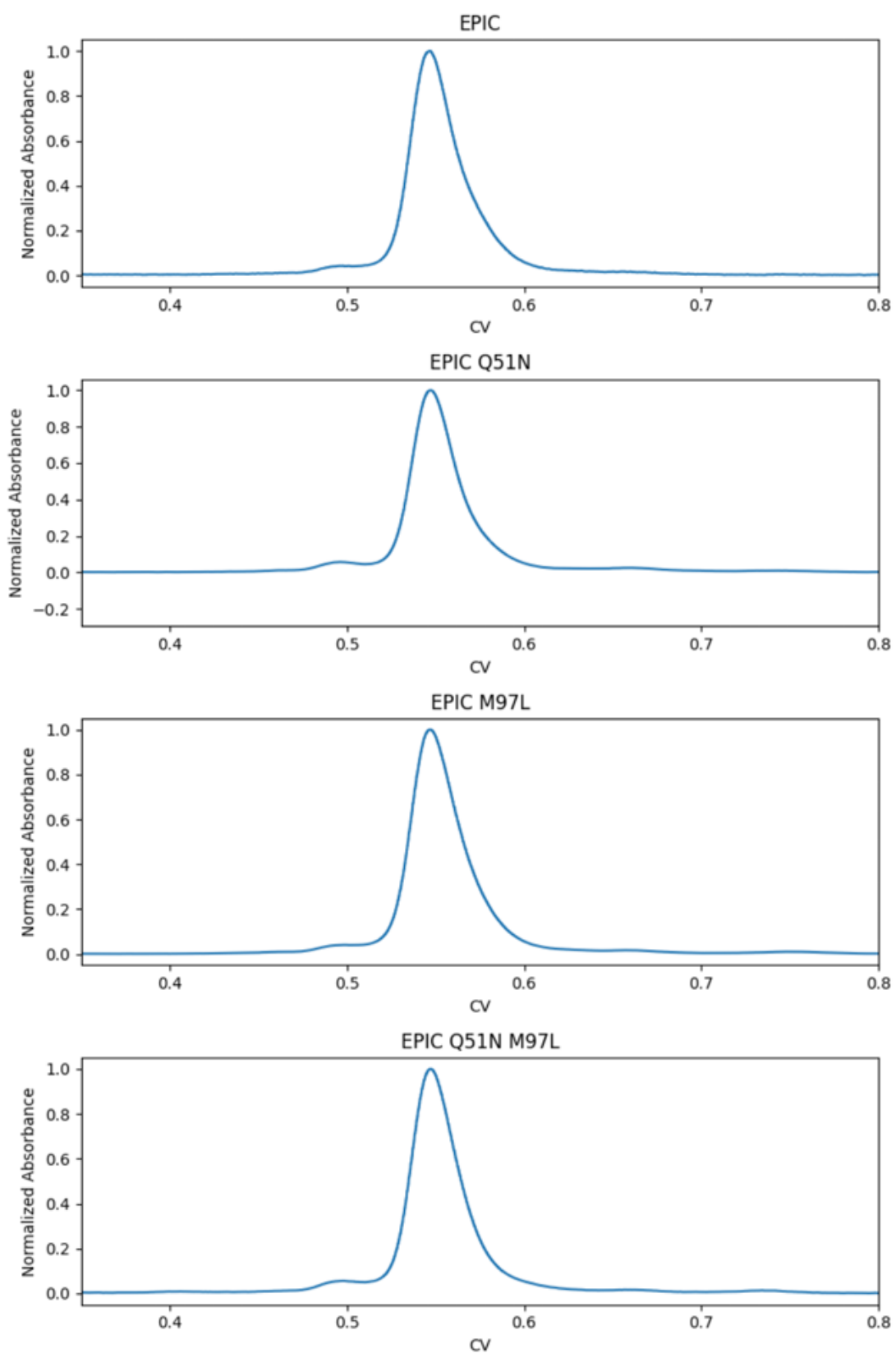

Supplementary Fig. 21. Size exclusion chromatograms of EPIC and its mutants show that each is monomeric. Ligand-free protein samples were injected at a concentration of > 100 µM and run on a Biorad 650 ENrich chromatography column in PBS buffer (pH 7.4). The proteins run slightly larger than the expected molecular weight (~17 kDa), but the results are consistent with monomers.

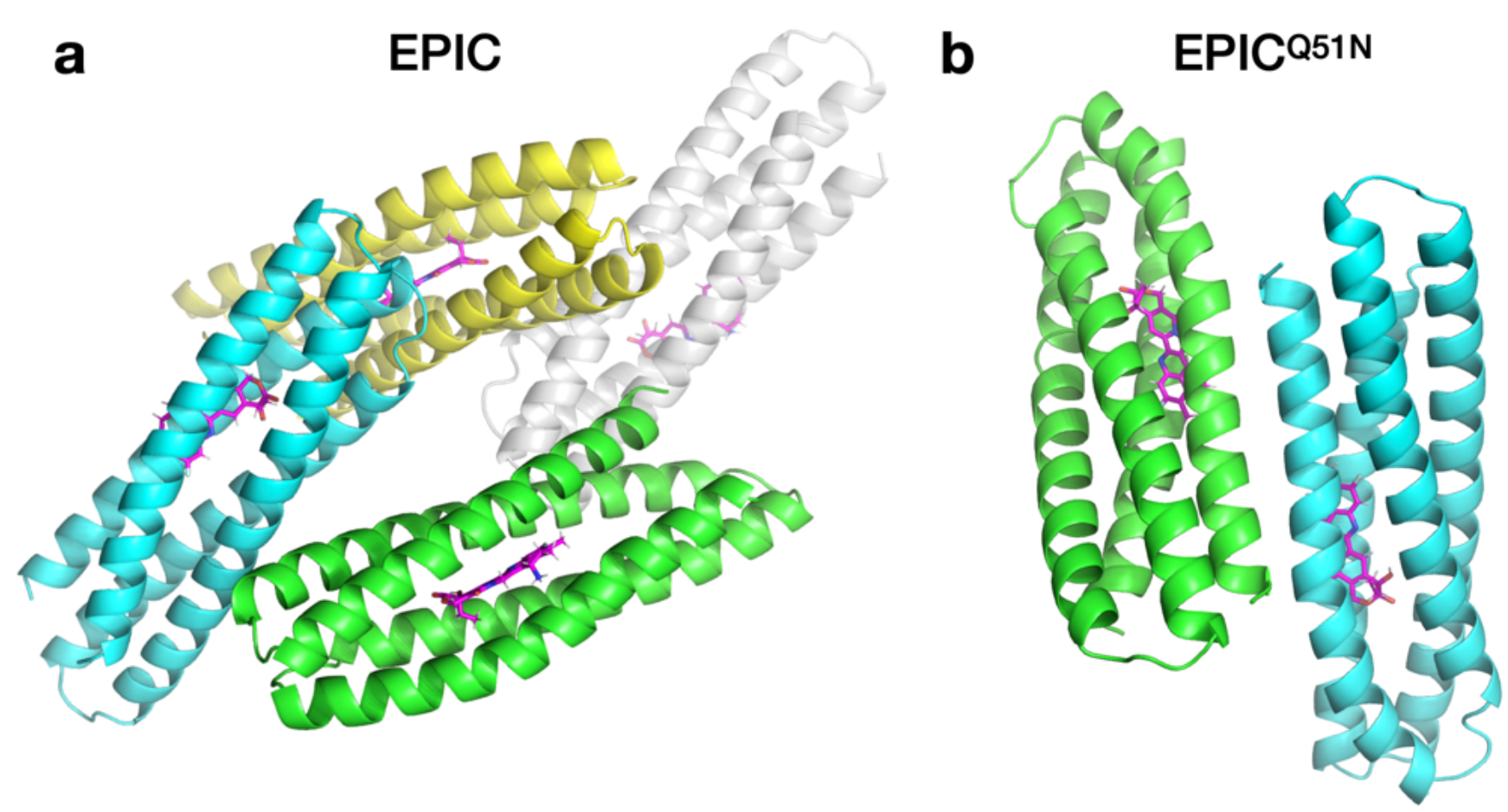

Supplementary Fig. 22. Asymmetric units of x-ray crystals of exatecan-bound EPIC and EPIC^{Q51N}. **a**, EPIC was solved with four chains in the asymmetric unit, and **b**, EPIC^{Q51N} with two chains. The crystals were formed under different crystallization conditions (see supplementary methods), and the proteins crystalized in different space groups. Exatecan is colored magenta and is bound in each chain with 100% occupancy.

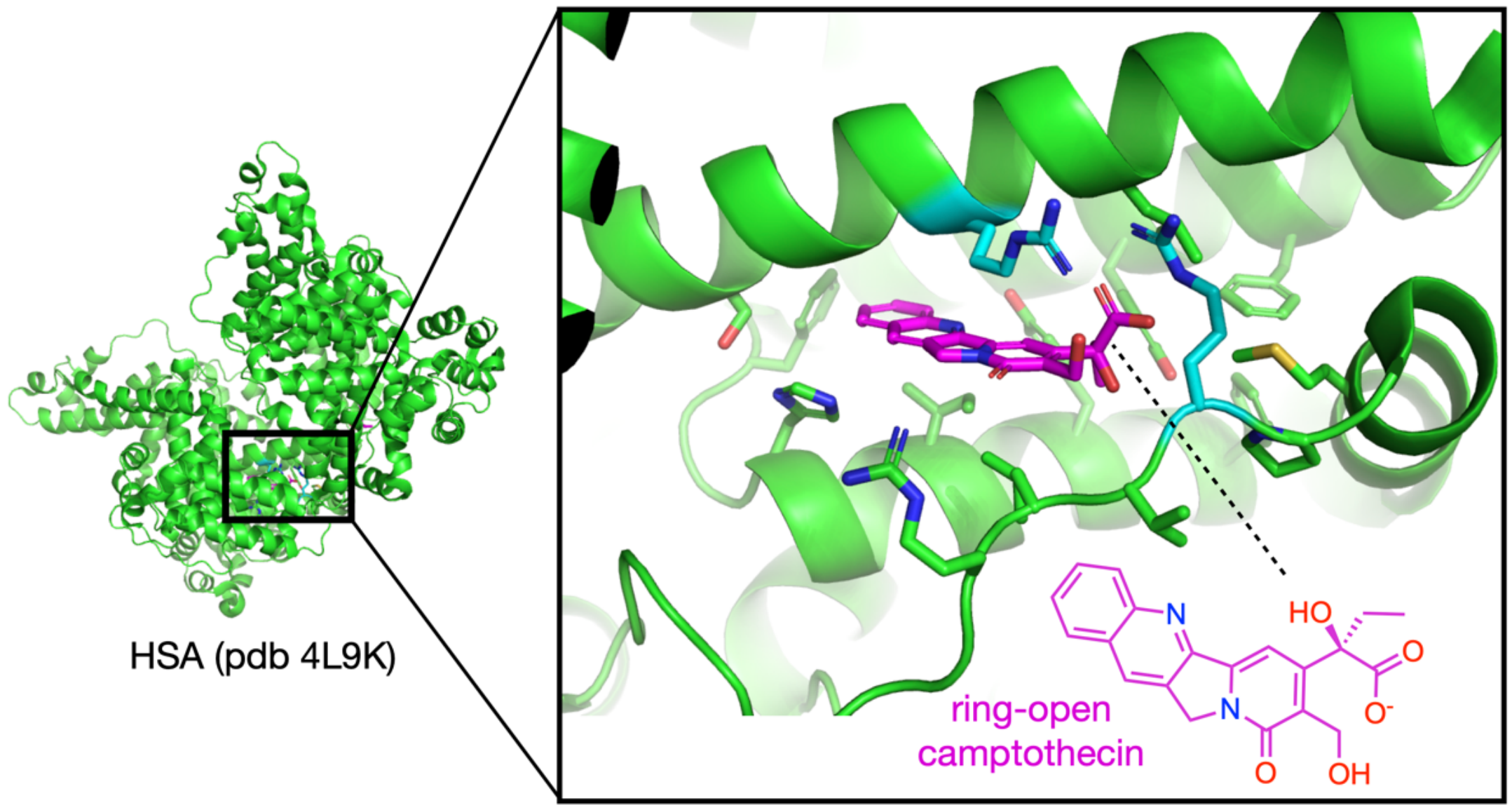

Supplementary Fig. 23. Camptothecin-bound structure of human serum albumin. Camptothecin (magenta) is bound to human serum albumin (HSA) in its ring-open carboxylate form, stabilized by nearby positively charged Arg residues (cyan). PDB accession code: 4L9K.

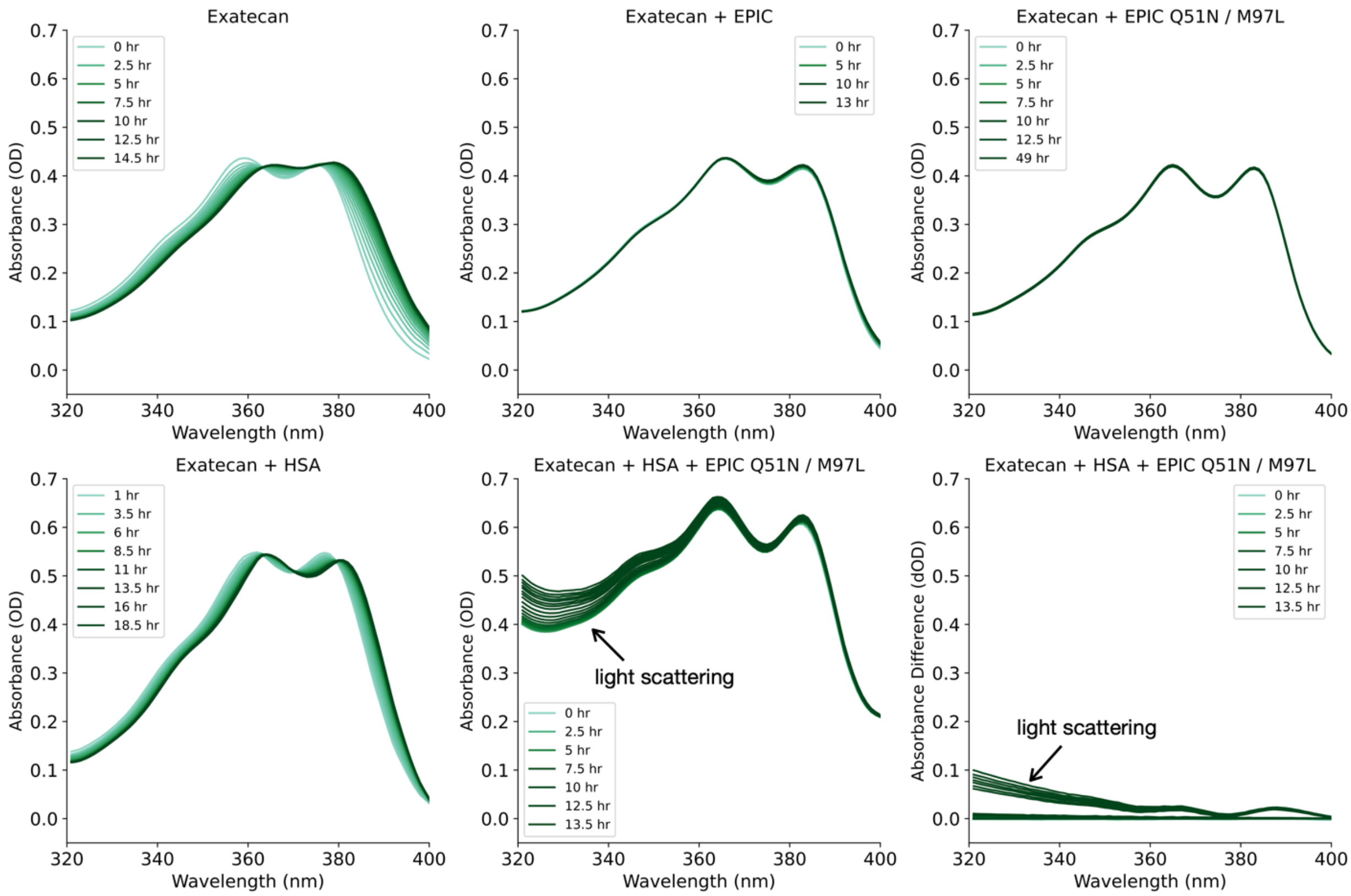

Supplementary Fig. 24. Time-resolved absorbance spectra of exatecan alone or with various proteins. Experiments were initiated from a ring-closed form of exatecan from a DMSO stock and spectrally monitored for ring opening. Experiments were performed at room temperature (20°C) in phosphate-buffered saline (pH 7.4) with 20 µM exatecan. Spectra for each 30 min time interval are shown, but only some spectra with various time delays are labeled in the plot legend to avoid cluttering. Designed proteins (EPIC and EPIC$^{Q51N/M97L}$) were included at equimolar concentrations (20 µM), whereas human serum albumin (HSA) was included at physiological concentration (500 µM), where most exatecan is expected to be bound. The high concentration of HSA resulted in some light scattering, which increased as a function of time. We predominantly removed the scattering component of the measured spectra through baseline correction (fitting and extrapolating a polynomial to wavelengths 450-850 nm). Parts of the spectra that show residual contributions from scattering (after correction) are labeled with an arrow in the lower right plots. The spectral dynamics observed in the HSA and EPIC$^{Q51N/M97L}$ mixture are consistent with a 100% scattering component. The lower right plot shows the absorbance difference of the data to the left (the time = 0 hr spectrum was subtracted for each subsequent spectrum).

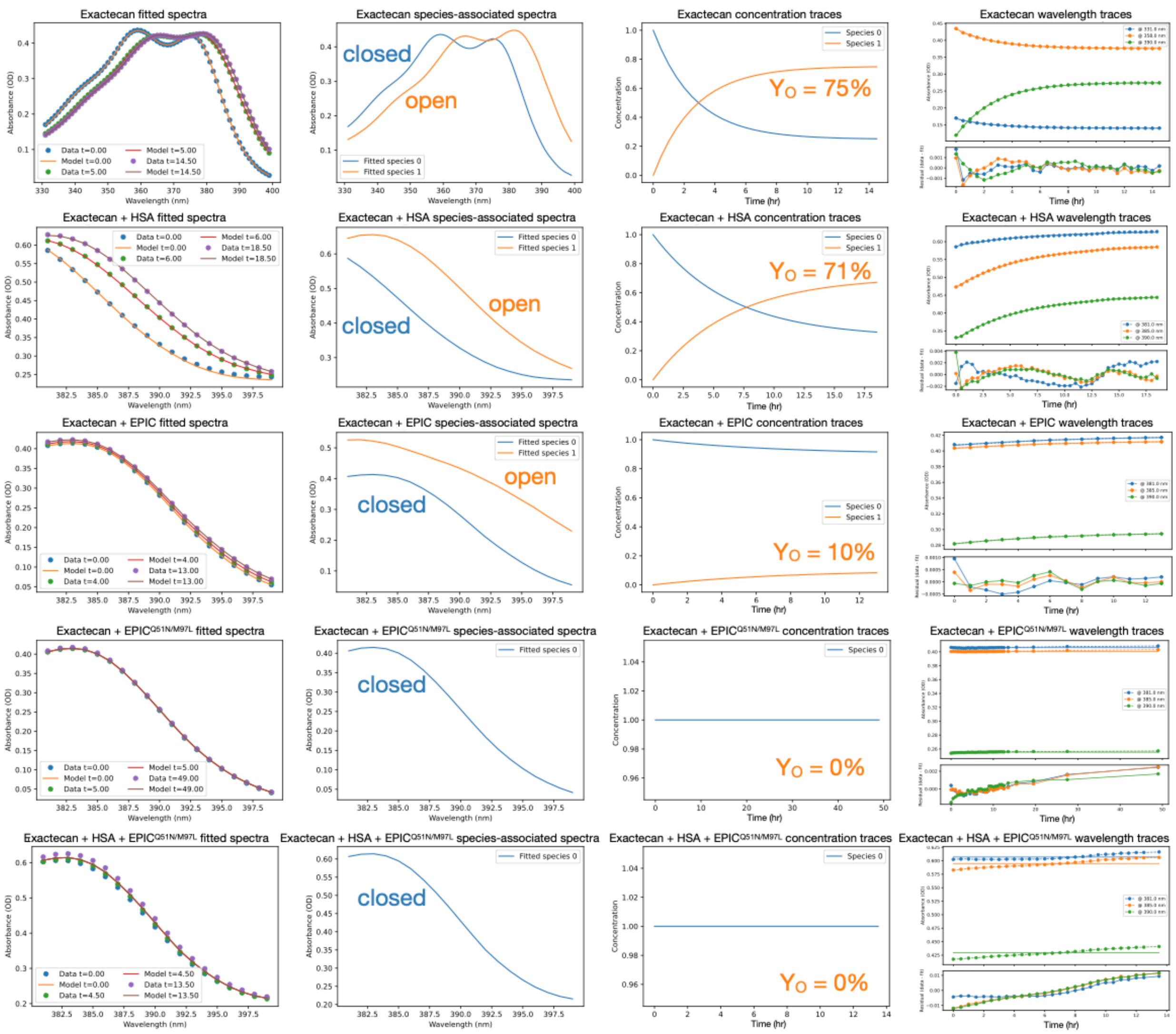

Supplementary Fig. 25. Fits of time-resolved hydrolysis experiments. Spectral ranges of exatecan absorbance spectra were fit to a one- or two-state kinetic model. The one-state model was a constant non-evolving species. The two-state model (0 or 1 in inset) was a reversible model with forward and reverse rate constants, with initial condition that all population starts in state 0. State 0 (blue) corresponds to the exatecan lactone-ring-closed state. State 1 (orange) corresponds to exatecan lactone-ring-open state. From left to right is shown i) a few spectra (circles) with overlayed fits (lines) at various time delays (units of hr), ii) the species-associated spectra resulting from the fit of the kinetic model, iii) the concentration profiles of species 0 and 1 from the fits, and iv) a few time traces at select wavelengths (markers) overlayed with the fits (lines), with fit residuals shown below (for a much narrower range of y-axis. Note the residuals are all very small relative to the magnitude of the fit data.). Experiments of exatecan that include EPIC$^{Q51N/M97L}$ are well-approximated by a single-state (constant) model of ring-closed exatecan with no time evolution. Equilibrium yields of ring-open exatecan (Y_O), computed from a thermodynamic model using the fit rate constants, are shown in the plots of concentration profiles. Fits were performed on data from Supp. Fig. 24. See Supp. Fig. 24 for experimental details.

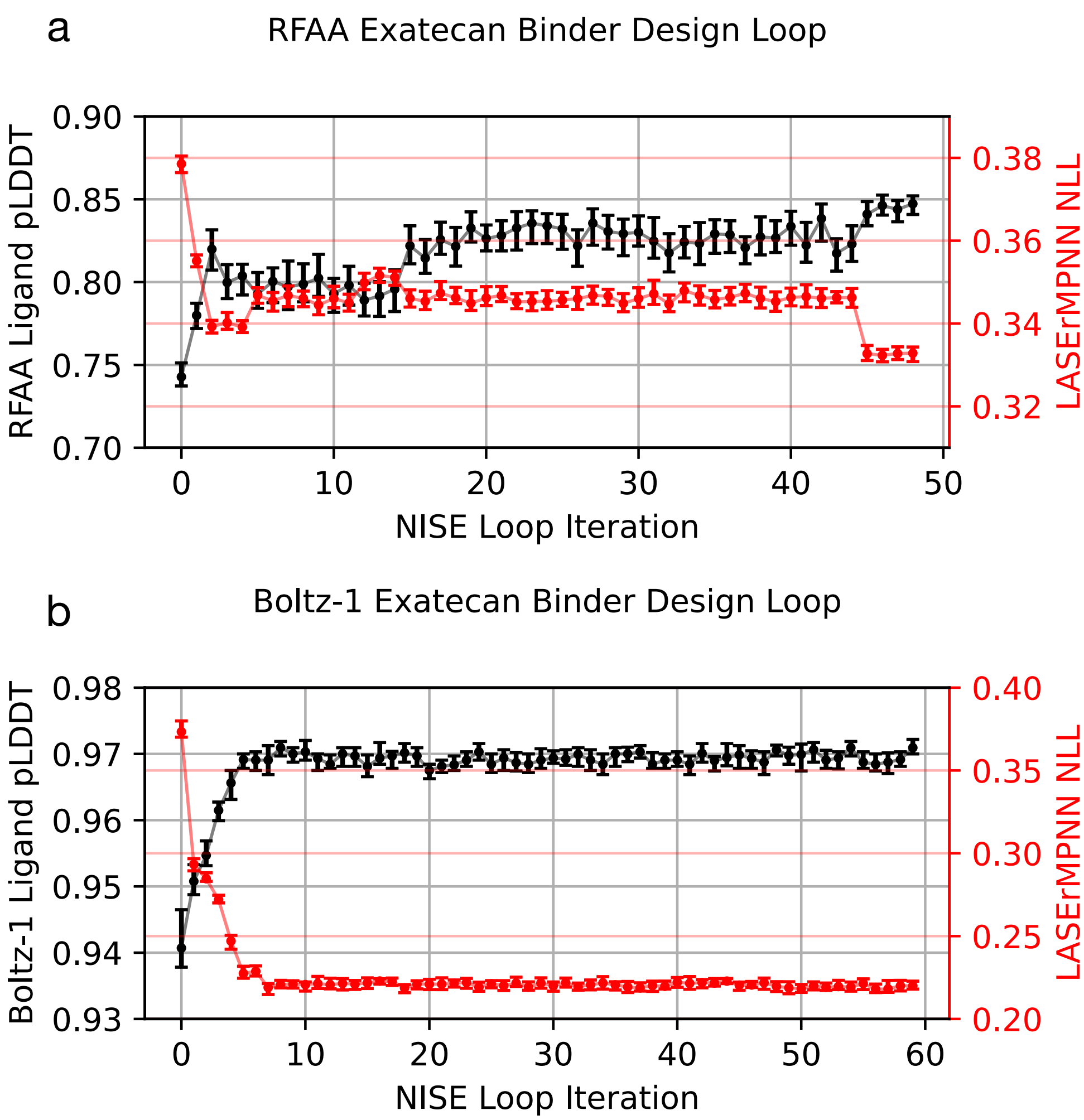

Supplementary Fig. 26. NISE jointly optimizes sequence and structure confidence using either RFAA or Boltz-1. a, Ligand pLDDT vs LASErMPNN Negative Log-Likelihood (NLL) for a NISE design loop mimicking that which generated EPIC using ligand pLDDT from RFAA as a proxy for structure confidence. **b,** Ligand pLDDT vs LASErMPNN NLL for a NISE design loop mimicking that which generated EPIC using Boltz-1 in place of RFAA. Boltz-1 appears to optimize joint confidence more efficiently than RFAA but both models jointly optimize the sequence and structure confidence. Data points show the value of the third quartile for sampled pLDDT and first quartile for NLL, with error bars representing 95% confidence intervals for these values computed by bootstrapping (N=100,000) the sampled data (see Statistical Bootstrapping NISE Confidence Metrics for Uncertainty Quantification). Metrics are computed over 1,500 LASErMPNN sequences per RFAA datapoint and 300 LASErMPNN sequences per Boltz-1 datapoint.

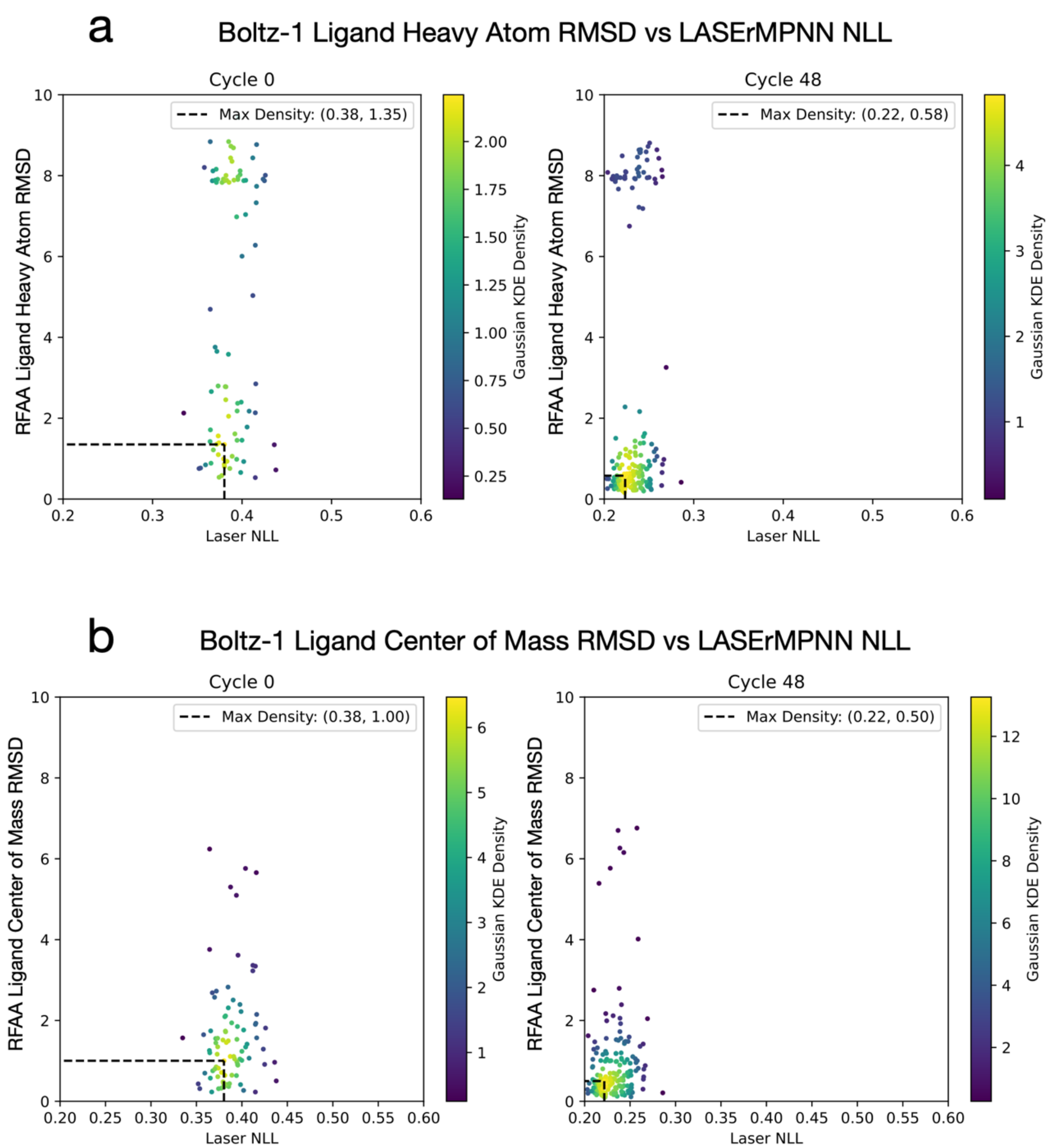

Supplementary Fig. 27. Boltz-1 outperforms RFAA at predicting exatecan bound in the designed orientation. a, Ligand heavy atom RMSD between input to LASErMPNN and Boltz-1 predicted structure vs LASErMPNN negative log-likelihood (NLL) for first NISE iteration (input backbone-ligand pair only) and the 48th NISE iteration. **b**, Ligand center-of-mass RMSD between input to LASErMPNN and Boltz-1 predicted structure vs LASErMPNN negative log-likelihood for first NISE iteration and 48th NISE iteration. Throughout the NISE design trajectory, Boltz-1 is generally able to place the ligand's center of mass within 2.0 Å of the binding site. Compared to RFAA (see Supp. Fig. 8), Boltz-1 shows better success at predicting the designed binding mode.

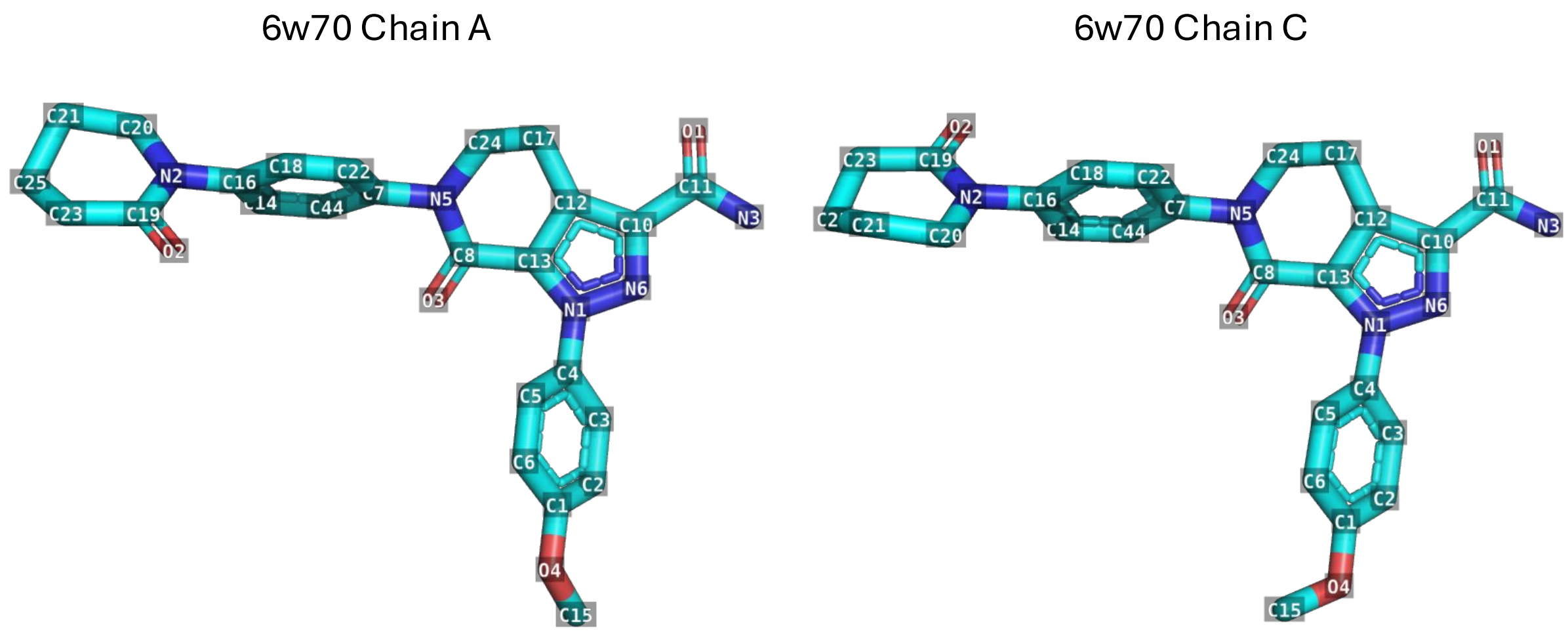

Supplementary Fig. 28. Apixaban conformers from 6w70 used in NISE design of apixaban binders. Structures are labelled by atom names from PDB 6w70 and differ primarily by rotation about the N2–C16 bond. O2, O3, O4, and C25 atoms were constrained inside the convex hull defining the surface of NTF2 backbones to generate CARPdock poses in which the N3 atom remains capable of accommodating a peg-FITC for binding Apixaban-FITC ligand.

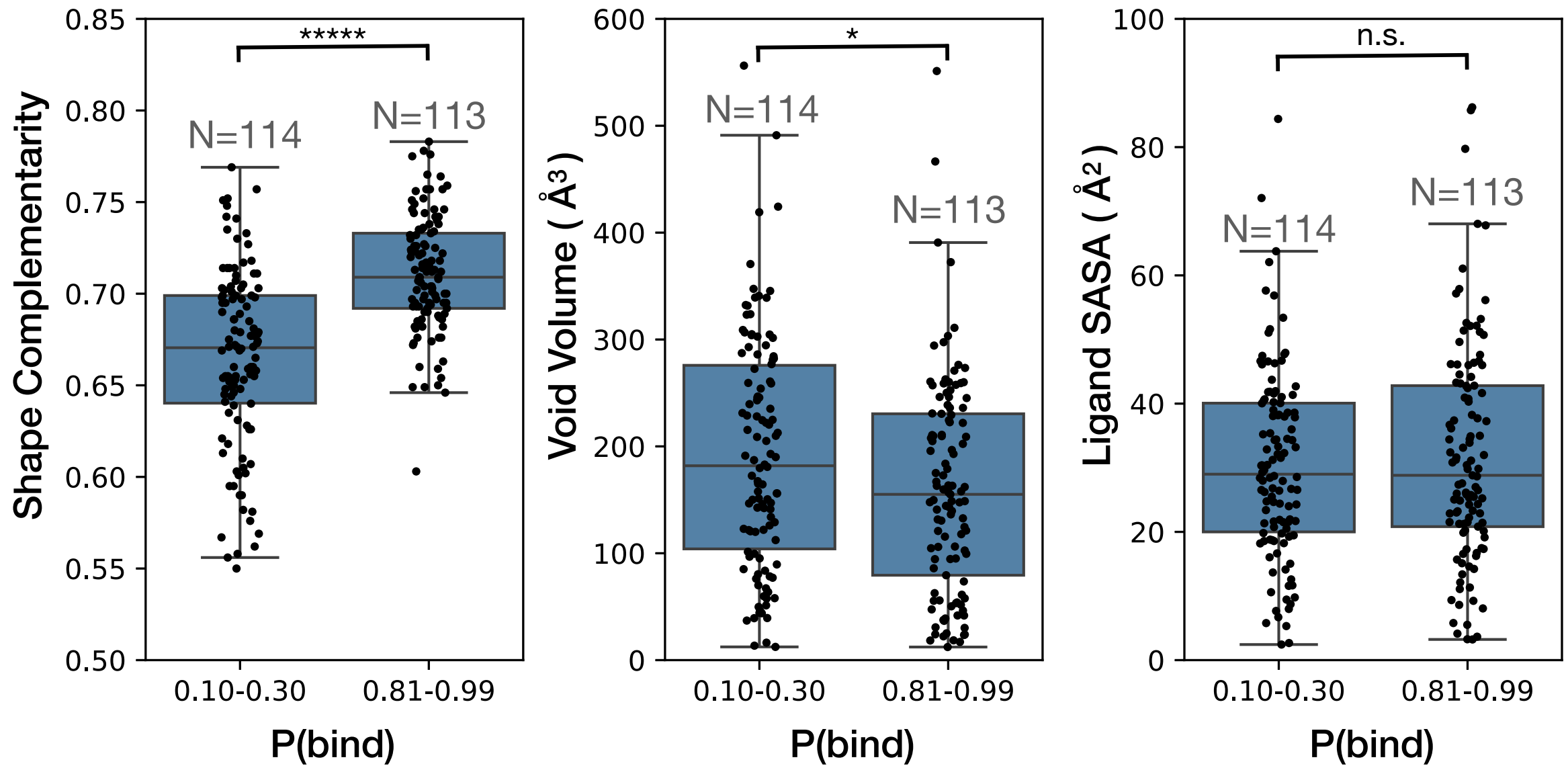

Supplementary Fig 29. Boltz-2 P(bind) captures information about designed binding site quality. To ascertain how maximizing the sum of ligand pLDDT and Boltz-2 P(bind) influences the quality of designed binding sites compared to maximizing ligand pLDDT alone, we filtered predicted structures from the apixaban-NTF2 CARPdock pose screening (see Design of Apixaban-Binding Proteins) with ligand pLDDT > 0.9 and computed Rosetta Shape Complementarity, Void Volume, and Ligand SASA for all designed sequences in the top and bottom deciles of Boltz-2 P(bind). High P(bind) poses have higher (better) Rosetta Shape Complementarity for the protein-ligand interface compared to low P(bind) poses (statistically significant two-sided Mann-Whitney U p-value < 1e-12). High P(bind) poses also possess lower void volume within the core of the protein core compared to those with low P(bind) (statistically significant, two-sided Mann-Whitney U p-value = 0.022). There is no significant difference in ligand solvent accessible surface area (SASA) between low P(bind) and high P(bind) poses (two-sided Mann-Whitney U p-value = 0.724). Box-and-whisker plots show the median (center line), the 25th and 75th percentiles (box limits), and the most extreme data points within 1.5× the interquartile range from the box (whiskers). Each of the points with ligand pLDDT > 0.9 in the top / bottom deciles of P(bind) is shown as a strip plot on top of the box-and-whisker plot (N = 113 and 114 for top and bottom deciles respectively).

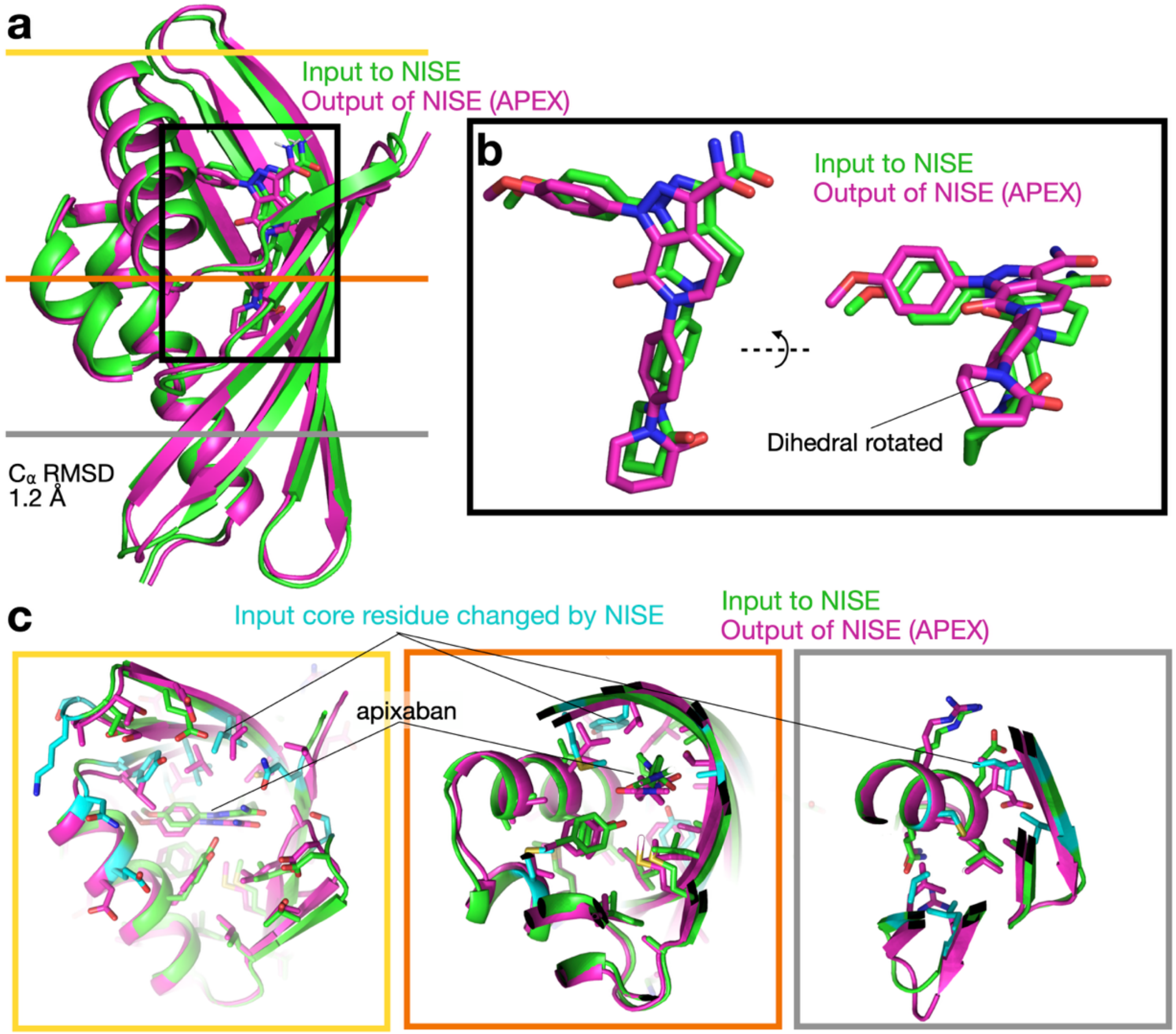

Supplementary Fig. 30. NISE-derived changes for APEX design. **a**, Input (green) and output (magenta) protein–ligand co-structures (predicted by Boltz-2) from the NISE trajectory of APEX (apixaban–protein complex). The structures are superimposed by C_α atoms. **b**, Changes in apixaban conformer and location as a result of NISE. Coordinates were extracted from co-structures after superposition by C_α atoms of the proteins. **c**, Slices of the superimposed co-structures, looking down from above the structures shown in **a**. The color of each box in **c** corresponds to the color of the slice in **a**. Cyan-colored residues of the input structure were mutated as a result of NISE to the final output structure and sequence of APEX (magenta). Sidechains of most solvent-exposed residues are not shown for clarity.

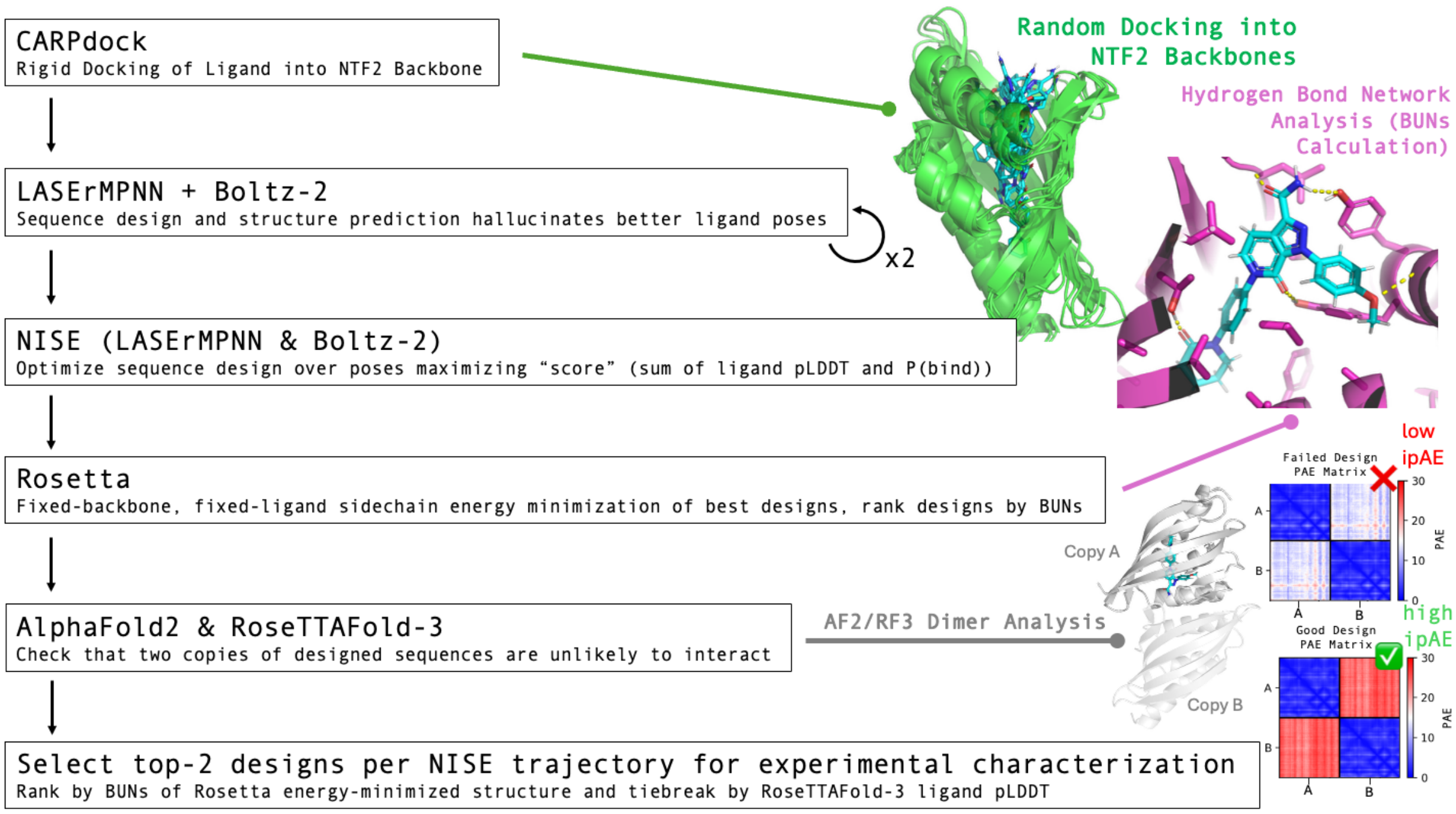

Supplementary Fig. 31. Flowchart depicting the design workflow that generated APEX. Initial starting points were generated using CARPdock for rigid protein-ligand docking into the NTF2 backbone. We perform two rounds of LASErMPNN sequence design and Boltz-2 structure prediction to identify promising starting points for the NISE protocol. We then run NISE trajectories which maximize the score of the sampled sequences per iteration (score defined by the sum of Ligand pLDDT and predicted_affinity_binary). Rosetta is used to energy minimize backbones passing RMSD and confidence filters to evaluate hydrogen bonding networks formed by the predicted structures (ranking them by BUNs). AlphaFold2-Multimer and RoseTTAFold-3 (RF3) were used as final filters for structural consistency with the Boltz-2 design model and the top-2 designs from each NISE trajectory with designs passing filters were selected as those with the lowest BUNs (tiebreaking by RF3 ligand pLDDT). See Design of Apixaban-Binding Proteins for information on specific filtering criteria.

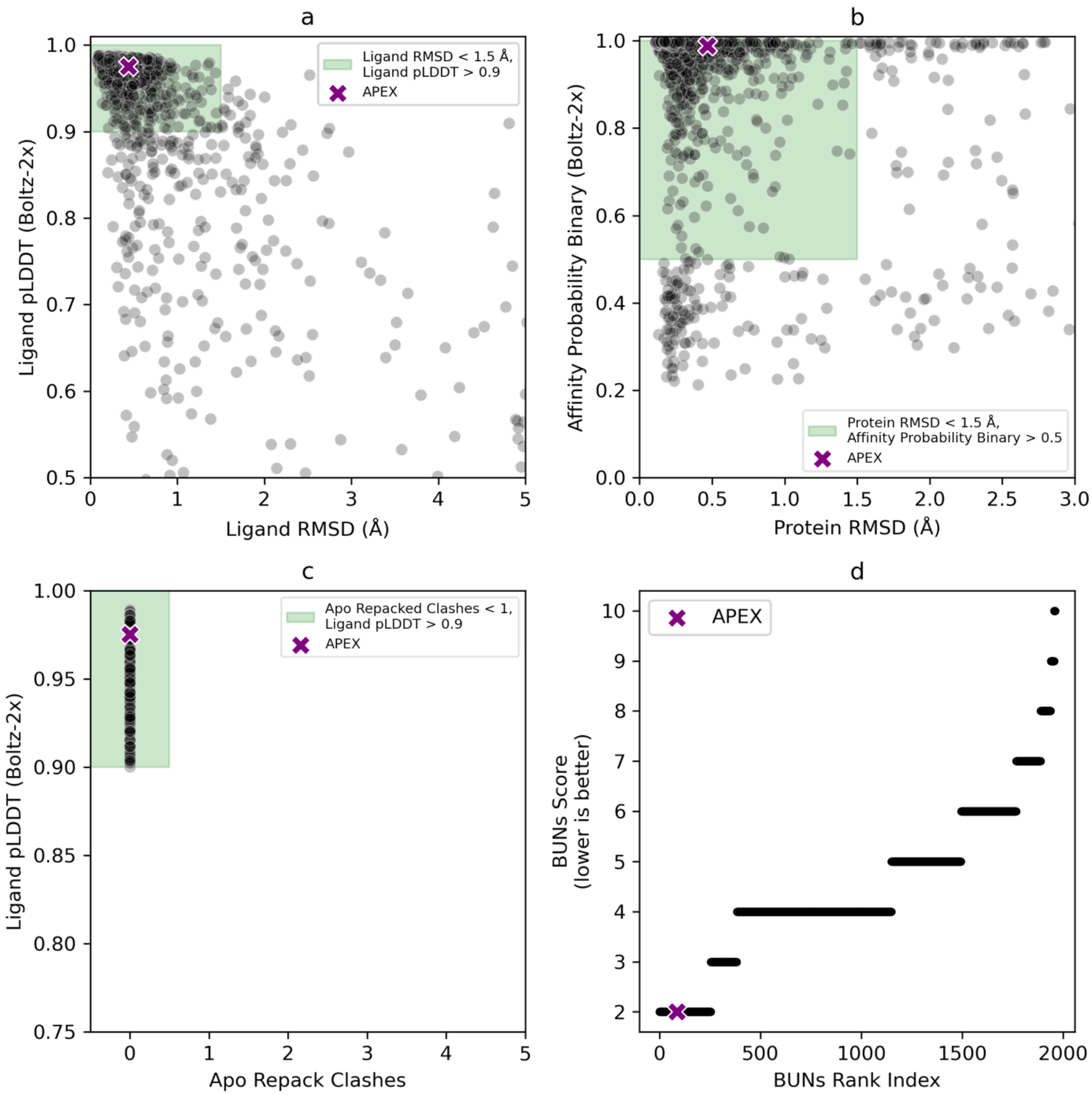

Supplementary Fig. 32. Filtering and ranking protocol for selection of apixaban binders from pooled NISE trajectories. a, **b**, LASErMPNN-designed sequences were filtered by ligand heavy-atom and protein Cα self-consistency RMSD < 1.5 Å, ligand pLDDT > 0.9, and affinity_probability_binary > 0.5 computed using Boltz-2. Selected regions are highlighted in green. APEX is marked with a purple X. **c**, We repacked the ligand-free proteins and then computed clashes with the reintroduced ligand (protein heavy atoms within 2.0 Å of ligand heavy atoms), selecting designs with no clashes (green). (Note that no designs after self-consistency and confidence filtering had predicted clashes.) **d**, We ranked remaining designs by the number of buried non-H-bonded polar atoms (BUNs) in the ligand. APEX was tied with other designs for the lowest BUNs score. It was 87th out of the total 2,560 sequences when using ligand pLDDT to break degeneracy of designs with equal BUNs. We predicted structures of the top 250 designs with AlphaFold2 and RoseTTAFold-3, and additional filtering based on these predicted structures left APEX as the top-ranking design (see Design of Apixaban Binding Proteins).

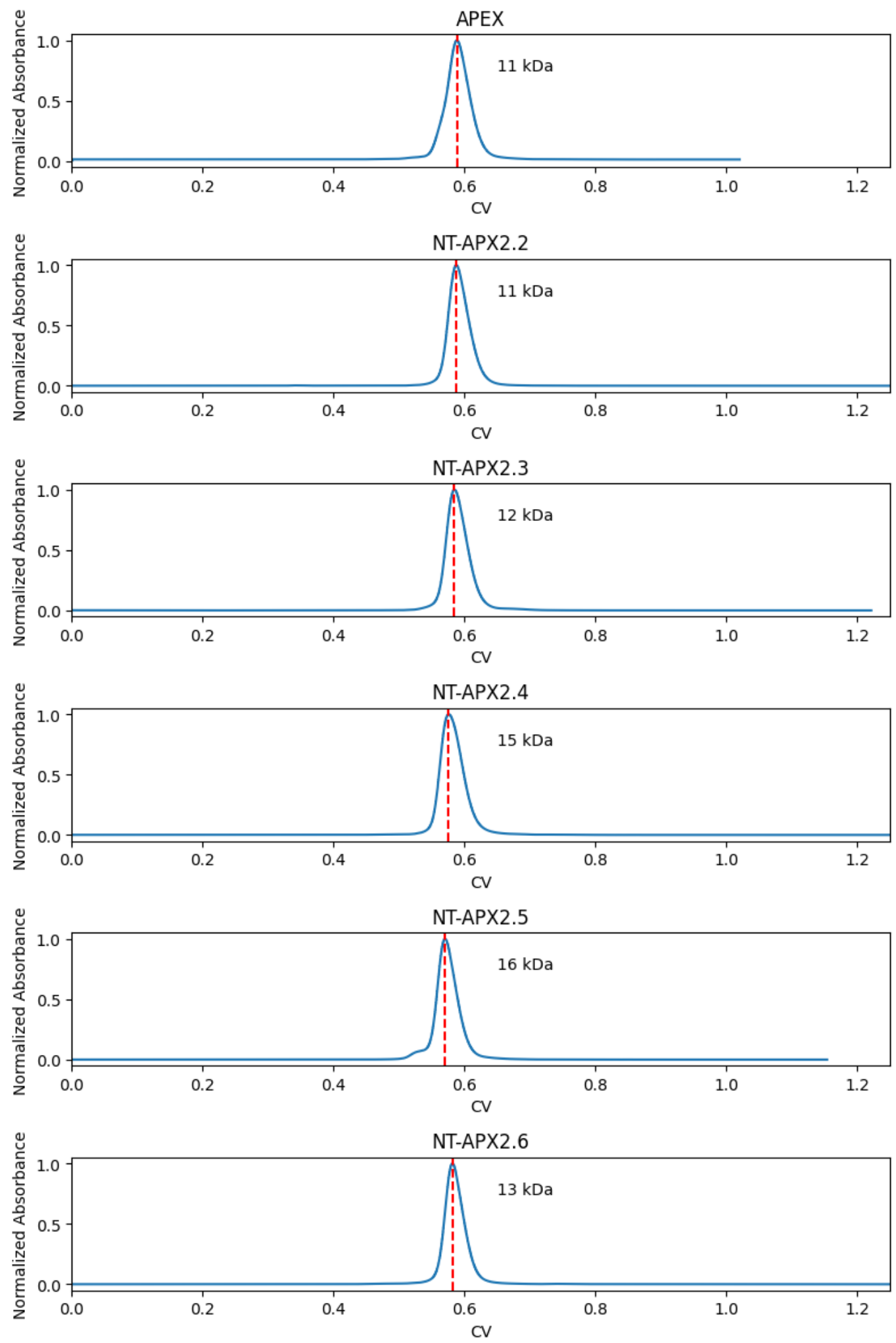

Supplementary Fig. 33. Size exclusion chromatograms of purified NTF2 designs. Proteins were injected onto a Biorad 650 ENrich chromatography column in PBS pH 7.4 buffer at greater than 100 µM concentration. Each peak is labeled with its expected molecular weight based on a standard curve.

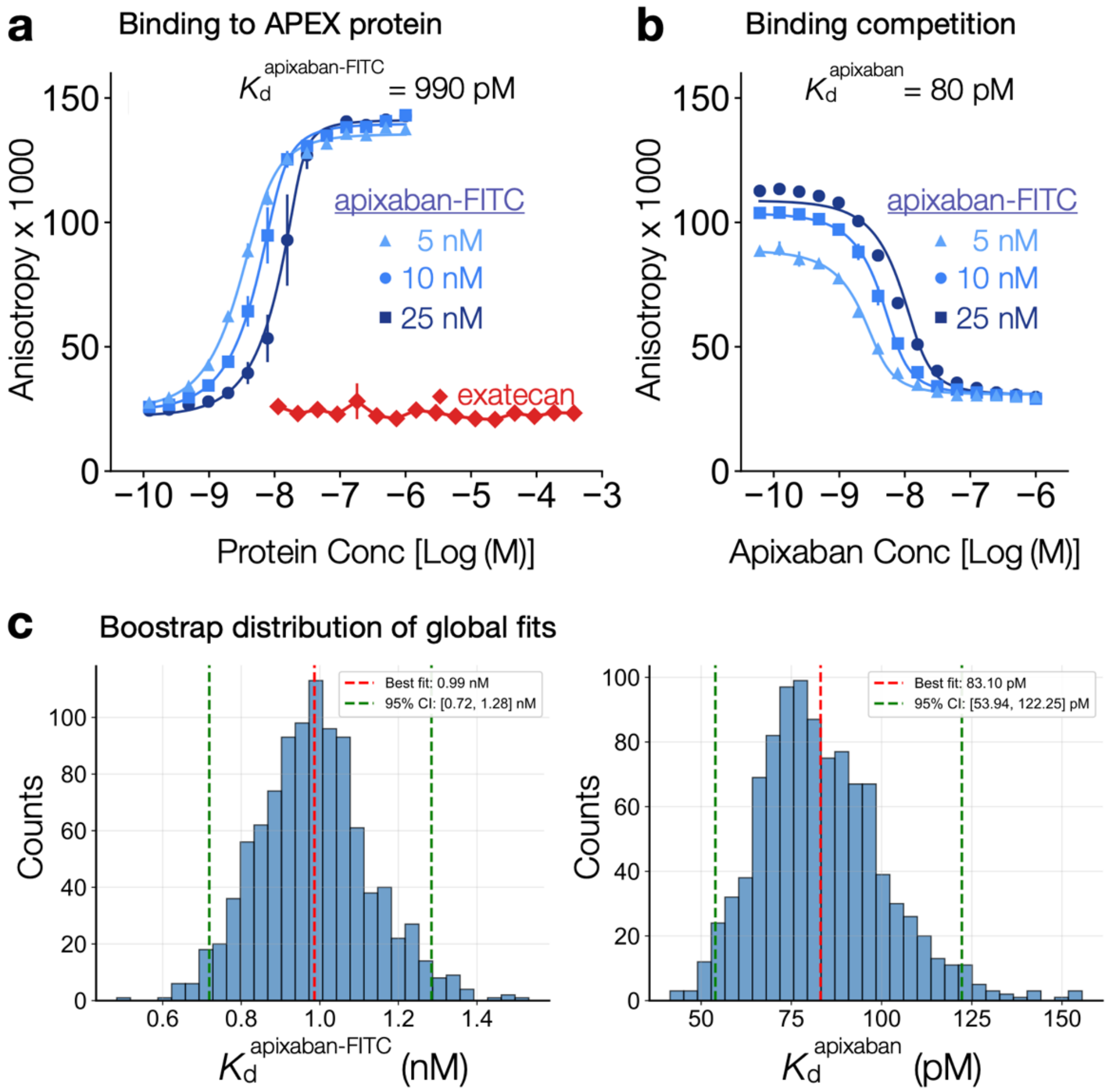

Supplementary Fig. 34. APEX binds apixaban tightly and specifically. a, Binding curves monitoring fluorescence polarization of apixaban-FITC as a function of protein concentration. Titrations across three concentrations of apixaban-FITC were globally fit for **a** and **b** together to derive dissociation constants. Fluorescence polarization of exatecan was also monitored as a function of APEX concentration (red); no binding was observed. **b**, Competition binding experiments monitoring fluorescence polarization of apixaban-FITC (5, 10, and 25 nM) for constant protein concentration and varying apixaban concentration. All data (blue markers) are fit globally (solid lines). The dissociation constants for apixaban-FITC and apixaban are fit simultaneously to a 1-to-1 binding model, sharing maximum anisotropy values. Minimum anisotropy was allowed to float locally but values did not differ significantly. **c**, Confidence intervals (95%, green dashed lines) of the fit dissociation constants are derived from bootstrapped distributions of the fits for 1000 trials. Value of the optimum fit is shown as red dashed lines.

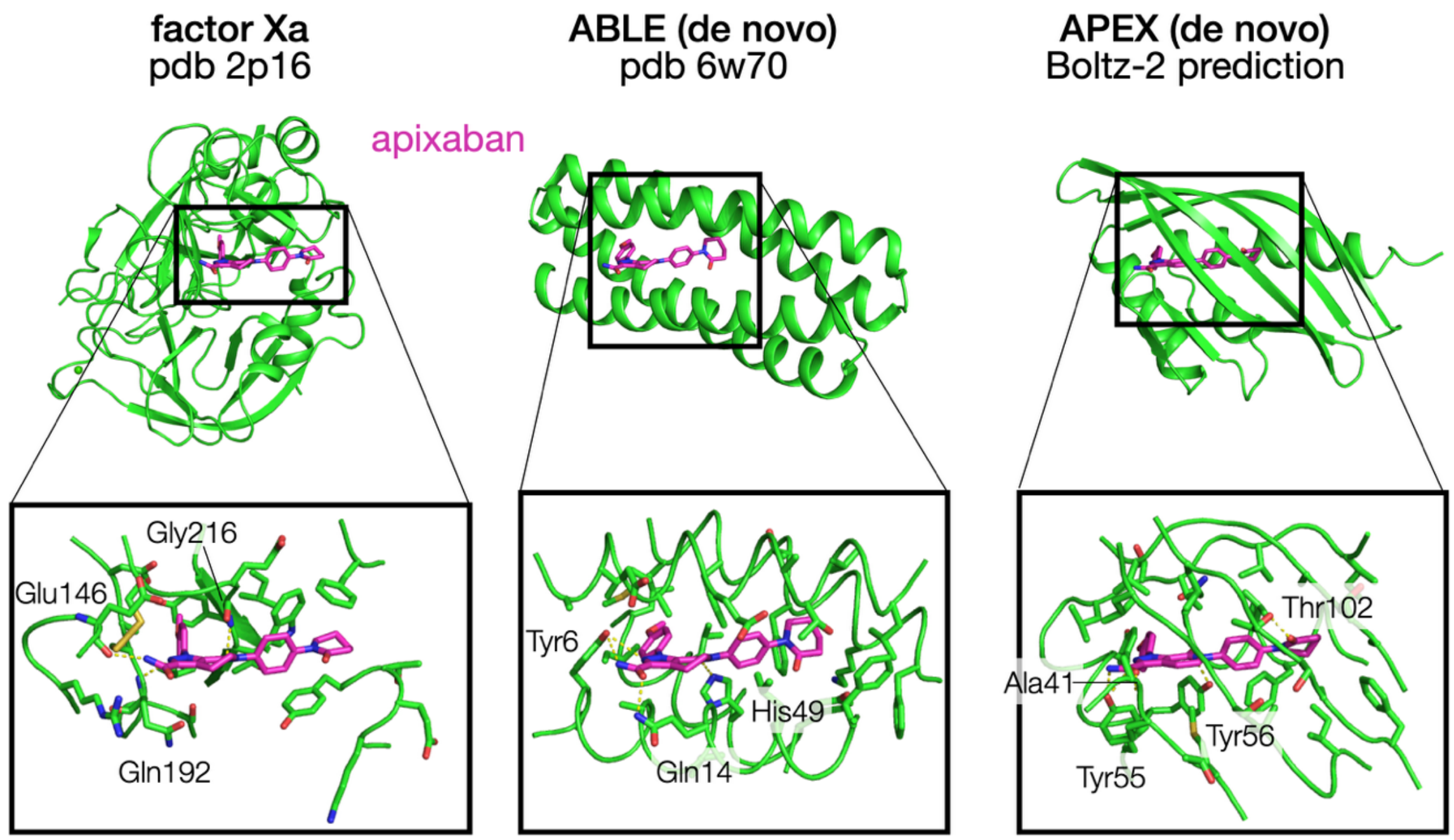

Supplementary Fig. 35. Binding sites of apixaban in natural and de novo proteins are dissimilar. The crystal **s**tructures of factor Xa (left, K_i = 80–700 pM), ABLE (middle, K_d = 7 μM), and the Boltz-2 predicted structure of APEX (right, K_d = 80 pM) were aligned by ring atoms in apixaban (magenta). Each box shows the same view of apixaban. Residues with heavy atoms within 5 Å of apixaban heavy atoms are shown as stick representation. Residues that participate in H-bonding interactions with the ligand are labeled. Factor Xa forms H-bonds with apixaban using only mainchain atoms and predominantly loop regions; ABLE uses only sidechain atoms and helical residues; and APEX uses both mainchain (Ala41) and sidechain atoms within β-sheet, helix, and loop regions.

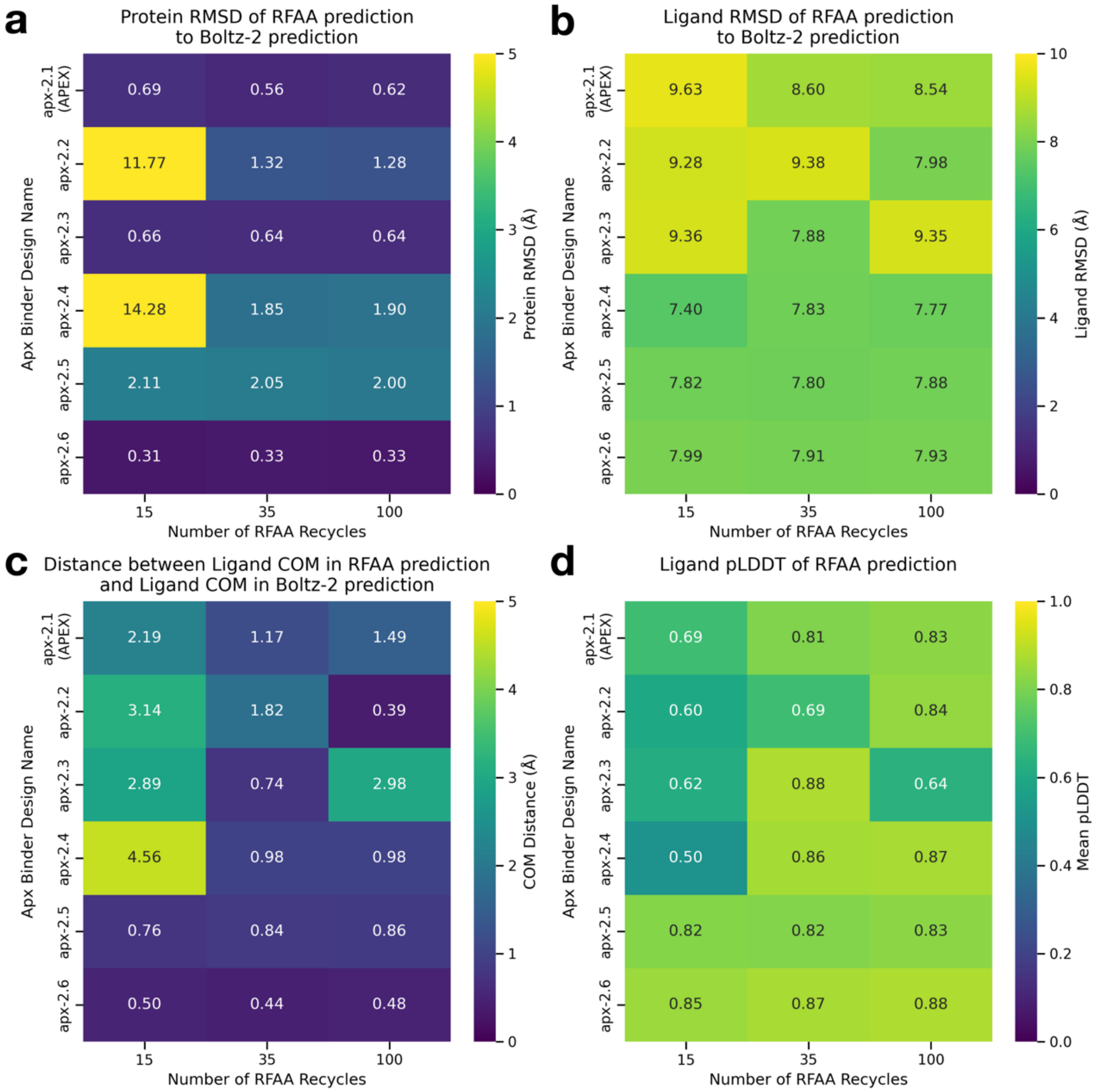

Supplementary Fig. 36. Post-hoc RFAA predictions of experimentally verified apixaban binders. Protein–ligand co-structure prediction by RFAA for each experimentally tested apixaban-binding protein (all but apx-2.6 bind apixaban with K_d < 50 nM) showing **a**, protein Cα RMSD of the RFAA prediction compared to the Boltz-2 prediction as a function of recycles within the RFAA structure module; **b**, **c**, ligand heavy-atom RMSD and distance between ligand heavy-atom center of mass, respectively, of the RFAA prediction compared to the Boltz-2 prediction, after superposition of the RFAA-predicted protein onto the Boltz-2-predicted protein; **d**, ligand pLDDT of apixaban from RFAA co-structure prediction, averaged across ligand heavy atoms. Note that these designs had high self-consistency between the intended co-structure (input to LASErMPNN for sequence design) and the predicted co-structure from Boltz-2, so disagreement between ligand placement between RFAA and Boltz-2 co-structure predictions (**b**) indicates low self-consistency of the designs using RFAA predictions. As such, and in addition to ligand pLDDT < 90 from RFAA, these experimentally verified designs would not have passed filters for ordering synthetic DNA if using RFAA for co-structure prediction. These results highlight that use of Boltz-2 was important for nominating these binders for experiments, and we recommend using Boltz-2 (or equivalent models) with NISE moving forward.

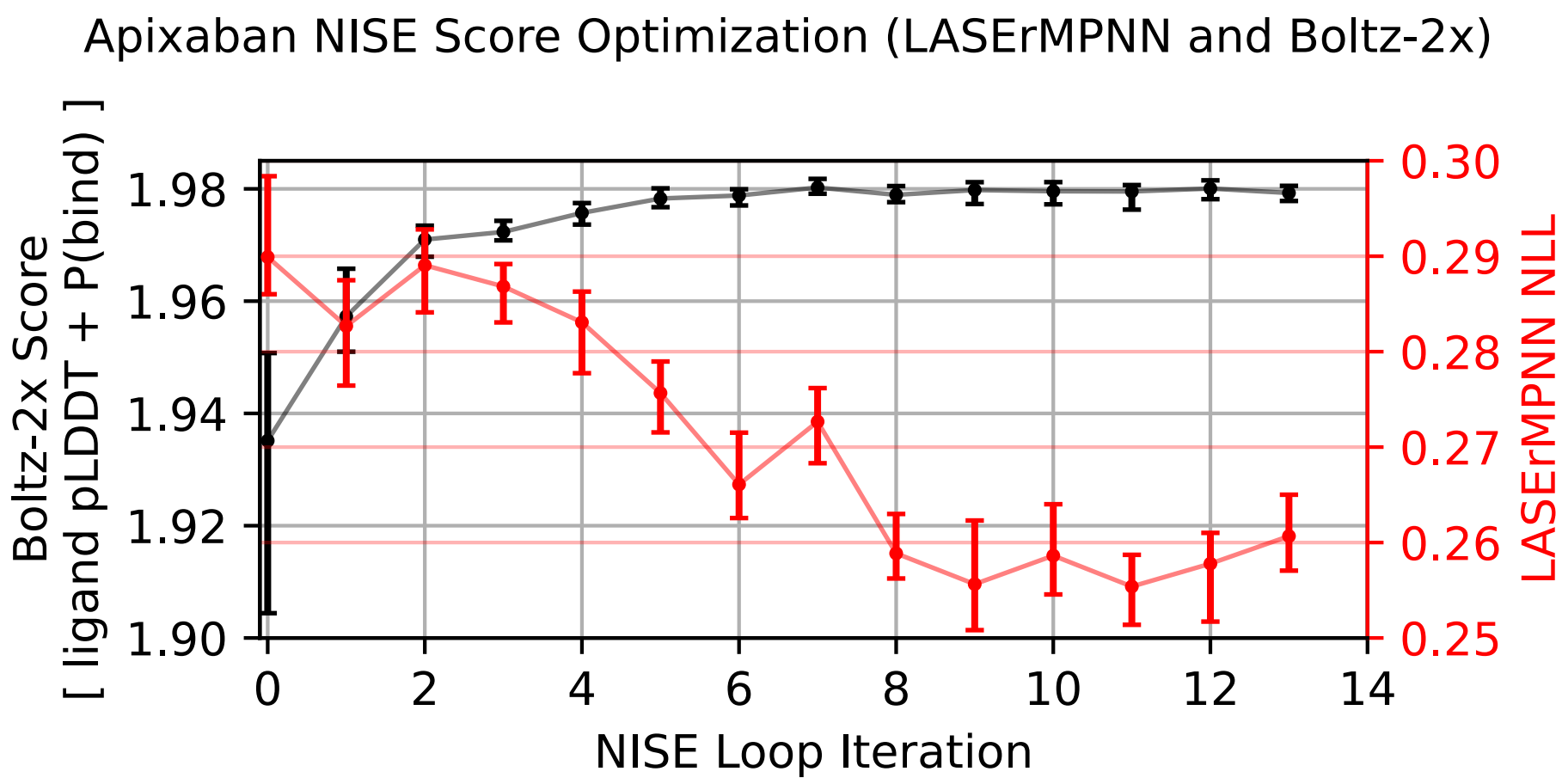

Supplementary Fig. 37. NISE optimizes Boltz-2 Score and LASErMPNN NLL for apixaban–NTF2 designs. We plot the "score" (sum of ligand pLDDT and affinity_probability_binary, P(bind)) from Boltz-2 and sequence negative log likelihood (NLL) from LASErMPNN per iteration of the NISE trajectory that generated APEX (64 sequences sampled per backbone, temperature 0.5, binding site temperature 0.7; the APEX sequence was generated on iteration index 8). The score increases and the NLL decreases as the NISE trajectory progresses, showing that these values are being optimized. Data points show the value of the third quartile for sampled score (black) and first quartile for NLL (red) across 192 LASErMPNN sequences per iteration (64 per sampled backbone), with error bars representing 95% confidence intervals for these values computed by bootstrapping (N = 100,000) the sampled data (see Statistical Bootstrapping NISE Confidence Metrics for Uncertainty Quantification).

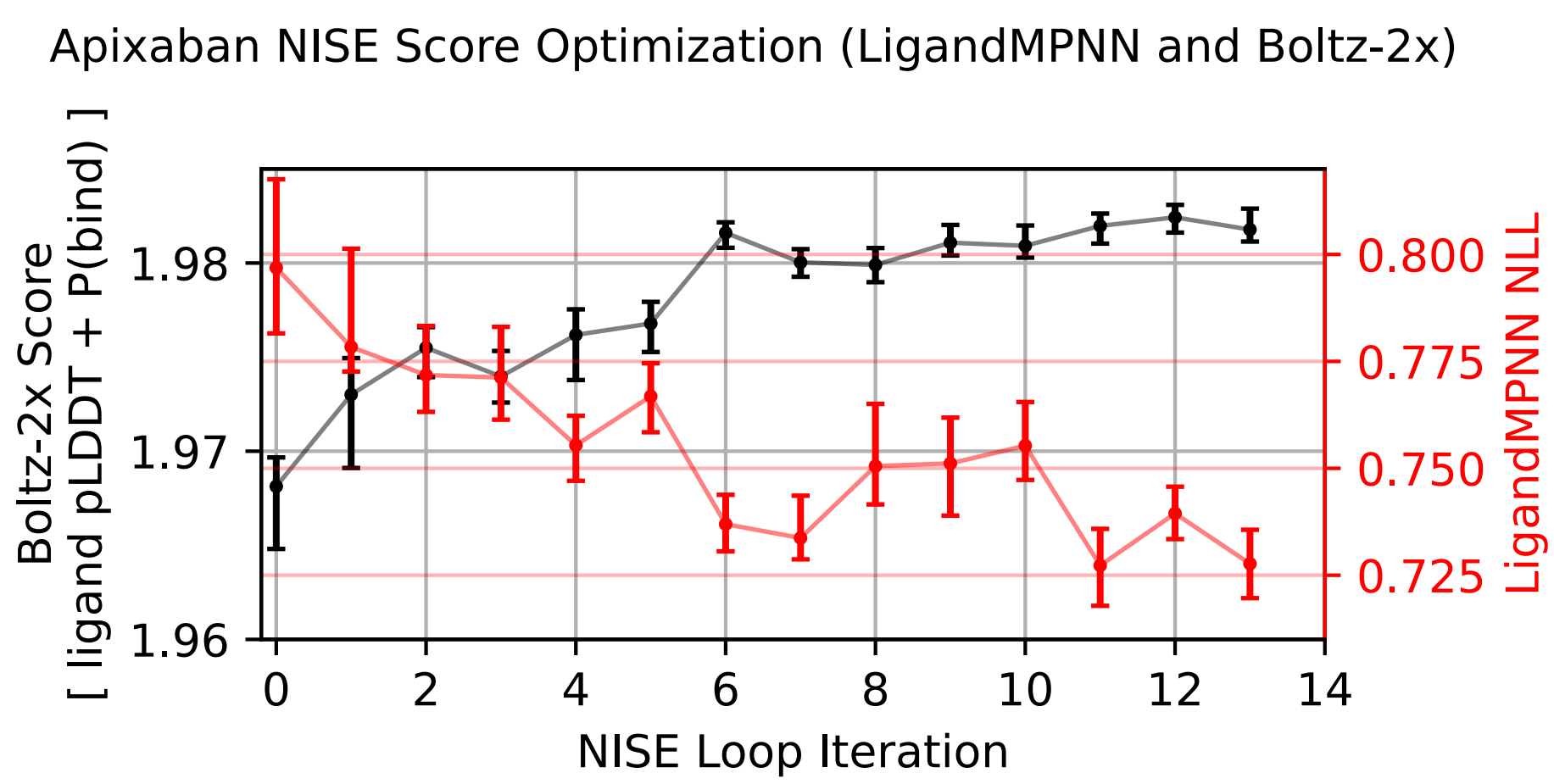

Supplementary Fig. 38. NISE optimizes Boltz-2 Score and LigandMPNN NLL for apixaban–NTF2 designs. We plot the "score" (sum of ligand pLDDT and affinity_probability_binary, P(bind)) from Boltz-2 and sequence negative log likelihood (NLL) from LigandMPNN per iteration of a NISE trajectory, using the same starting apixaban–NTF2 structure as the trajectory which produced APEX. NISE was reimplemented using LigandMPNN (with weights ligandmpnn_v_32_010_25.pt), sampling 64 sequences per backbone at temperature 0.5, with otherwise the same configuration as the trajectory which generated APEX. LigandMPNN NLL was computed as the negative $\log_{10}$ of LigandMPNN overall_confidence for each designed sequence. Data points show the value of the third quartile for sampled score (black) and first quartile for NLL (red), with error bars representing 95% confidence intervals for these values computed by bootstrapping (N = 100,000) the sampled data (see Statistical Bootstrapping NISE Confidence Metrics for Uncertainty Quantification).

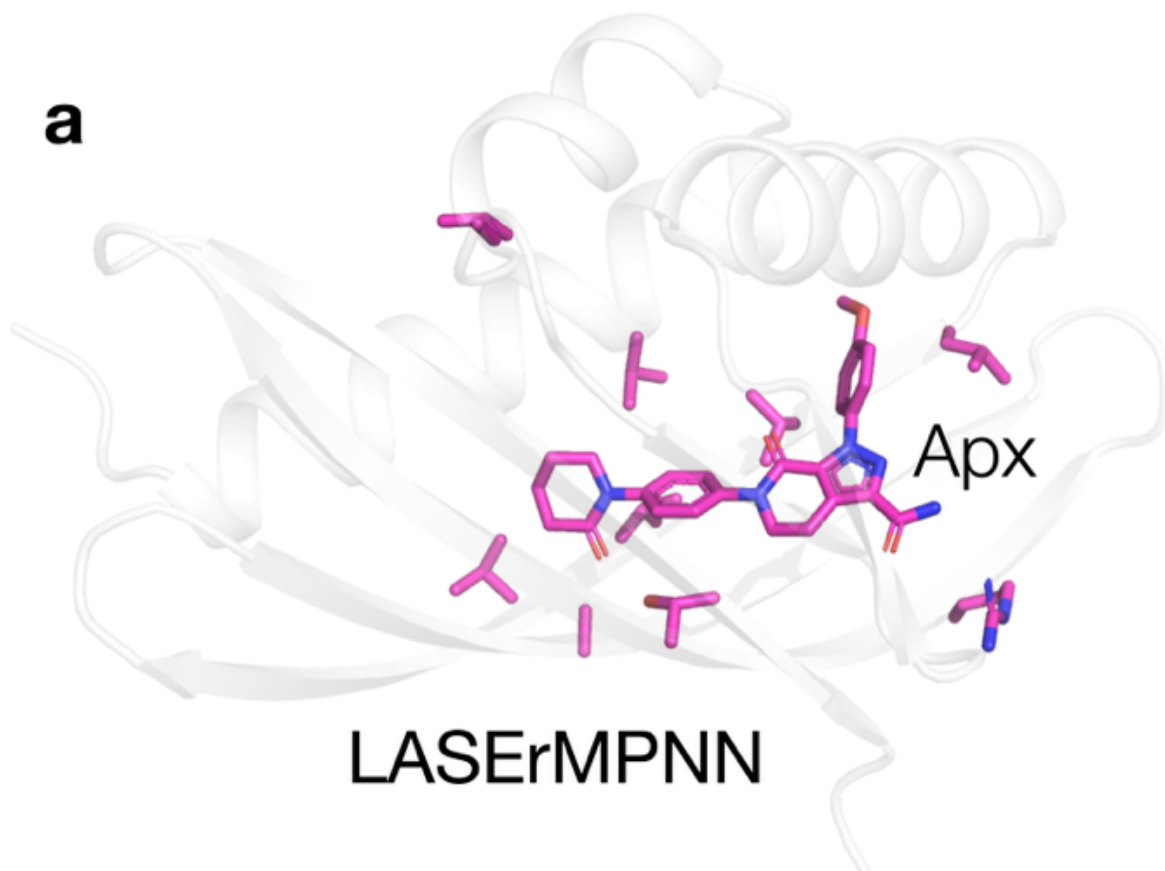

Supplementary Fig. 39. LigandMPNN designs larger residues in the binding pockets of apixaban–NTF2 designs. **a**, Top design from NISE using LASErMPNN and Boltz-2. **b**, Top design from NISE using LigandMPNN and Boltz-2. Sidechains are shown for residues that differ in the binding pocket or nearby protein core between the two designs in **a** and **b**. NISE using LigandMPNN results in designs with larger residues near the ligand, such as Phe, Leu, and Ile (green), whereas NISE using LASErMPNN results in designs with smaller residues near the ligand, such as Val, Thr, and Ala (magenta).

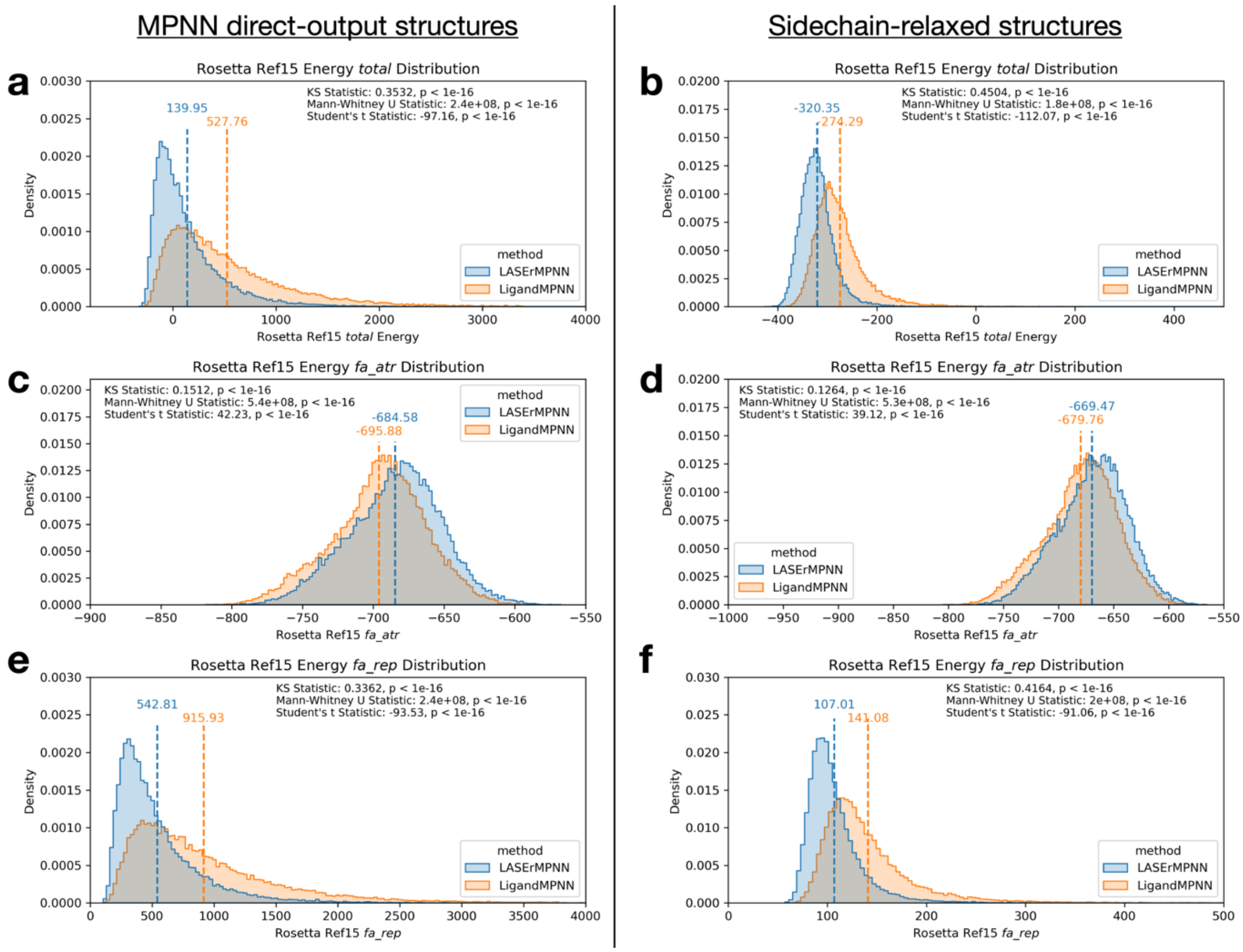

Supplementary Fig. 40. LigandMPNN generates overall sequences that are more overpacked than those from LASErMPNN. We assessed direct outputs and Rosetta-relaxed (sidechains only) outputs from both LASErMPNN and LigandMPNN on a diverse set of apixaban–NTF2 docked poses generated by CARPdock (30,000 generated sequences total across 10,000 poses). **a**,**b**, LASErMPNN sequences have lower (more favorable) total pose energies than LigandMPNN sequences before and after a sidechain-only Rosetta Relax protocol (fixed ligand, fixed backbone). While LigandMPNN sequences have lower overall attractive van der Waals energies (fa_atr) than LASErMPNN sequences before and after a sidechain-only Rosetta Relax protocol (**e**,**f**), LigandMPNN sequences have substantially higher overall repulsive van der Waals energies (fa_rep) than LASErMPNN sequences both before and after a sidechain-only Rosetta Relax protocol (**g**,**h**). This makes the packed proteins from LigandMPNN higher energy on average (i.e. more energetically unfavorable) than those from LASErMPNN (**c**,**d**). For these computations, hydrogens were stripped from all protein and ligand structures. Both LASErMPNN and LigandMPNN models output packed sidechain coordinates directly. Direct MPNN outputs and Rosetta-Relaxed outputs were scored by Rosetta score_jd2 and Relax protocols, respectively. Differences in both means and empirical distributions are statistically significant for all panels (Student's t-test, Mann-Whitney U-test, and Kolmogorov-Smirnov test; $p < 1e-16$; all tests two-sided).

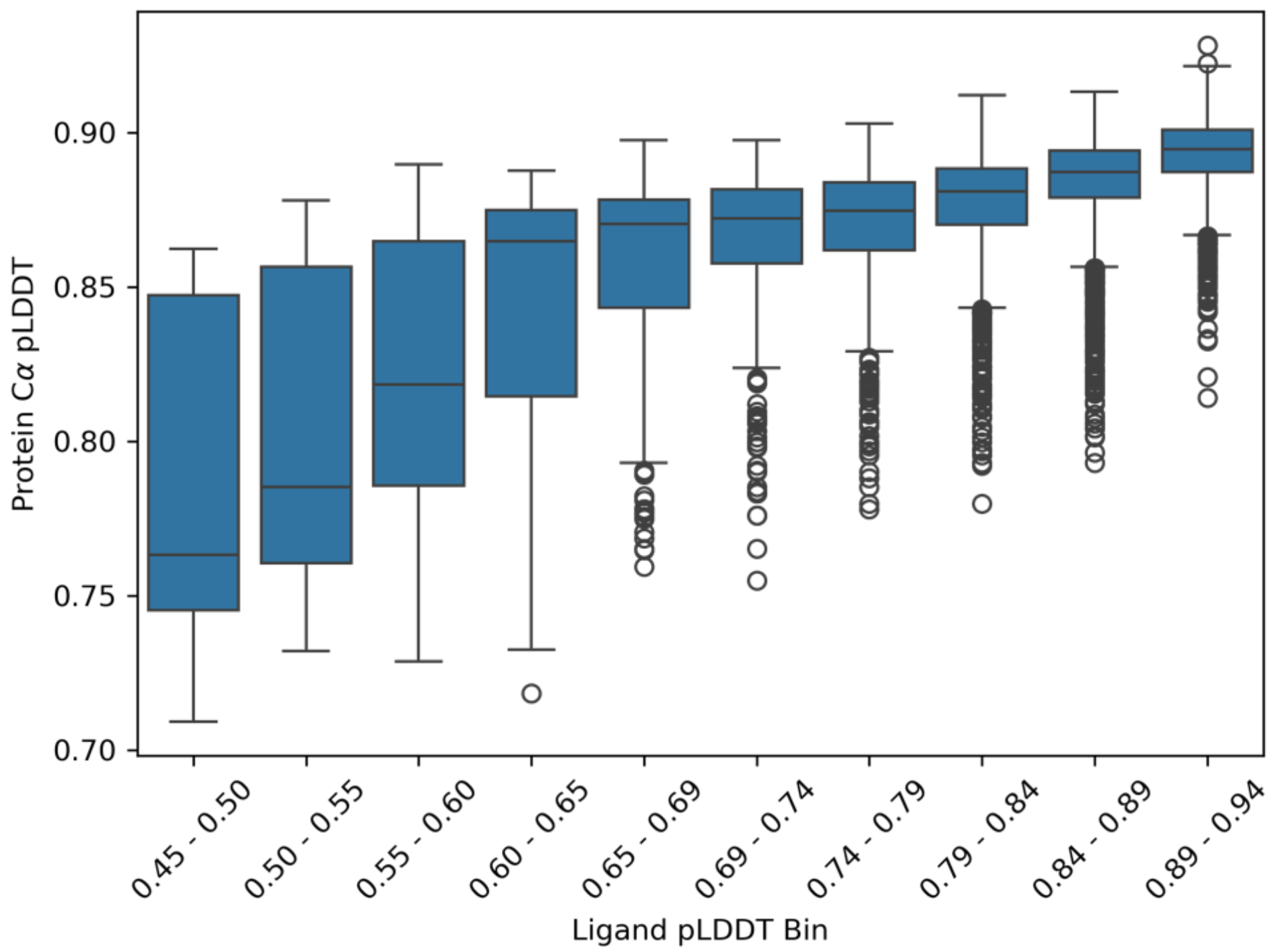

Supplementary Fig. 41. Ligand pLDDT and protein Cα pLDDT are positively correlated. We plot pooled ligand and protein pLDDT values generated by RoseTTAFold-All Atom (RFAA) over 48 iterations of NISE using the CR4 exatecan-bound co-structure as input (N=15,137 datapoints from NISE trajectory with ligand heavy atom RMSD < 1.5 Å and protein Cα RMSD < 1.5 Å). The box plots show protein Cα pLDDT statistics of designs with ligand pLDDT falling in the labeled bin range. The data show that by maximizing ligand pLDDT, NISE also implicitly maximizes protein Cα pLDDT.

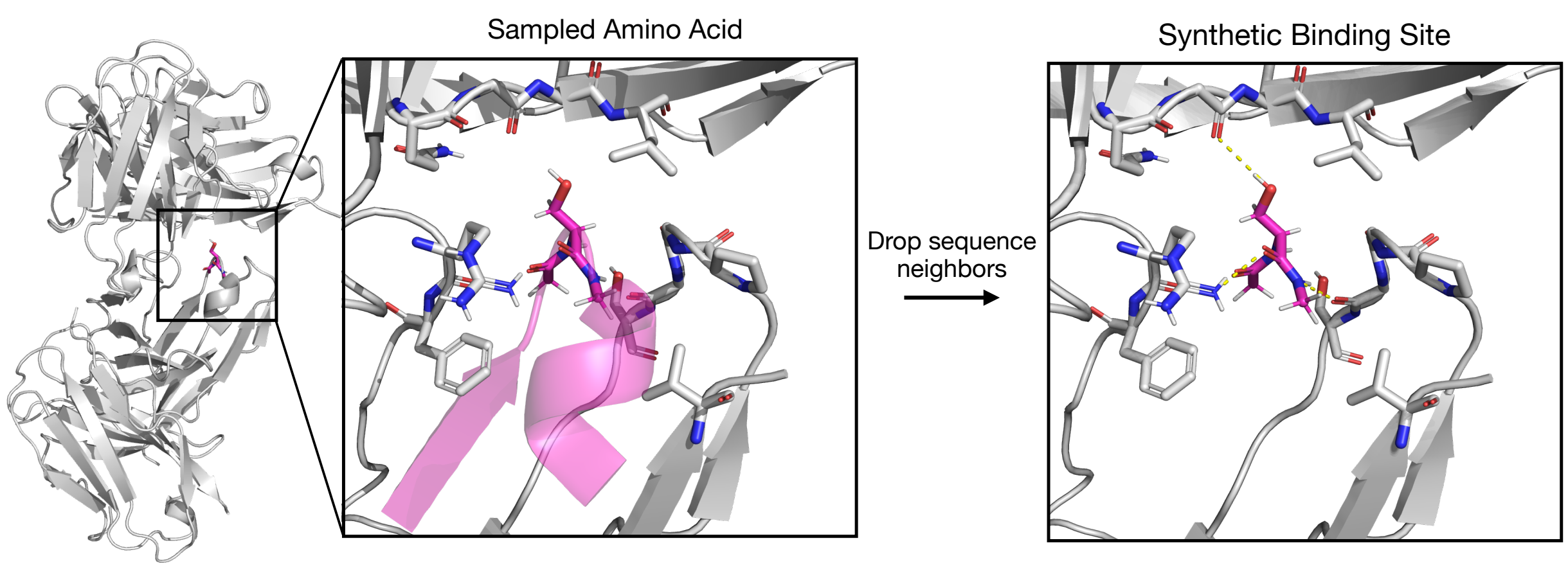

Supplementary Fig. 42. Amino acids are used as "pseudoligands" to augment ligand training data for LASErMPNN. An example pseudoligand is shown, using a serine residue. A synthetic binding site is generated around a serine pseudoligand (magenta sticks) sampled within the protein complex with PDB Code 7w7q. Methyl caps are applied to ensure the pseudoligand is physically plausible and in distribution of the pre-trained ligand encoder. Sequence-local residues, which are dropped from the protein graph to form the synthetic binding site,

are depicted as a transparent magenta cartoon in the first panel. Sidechains within 5.0 Å of the synthetic ligand forming a synthetic binding site are shown as grey sticks.

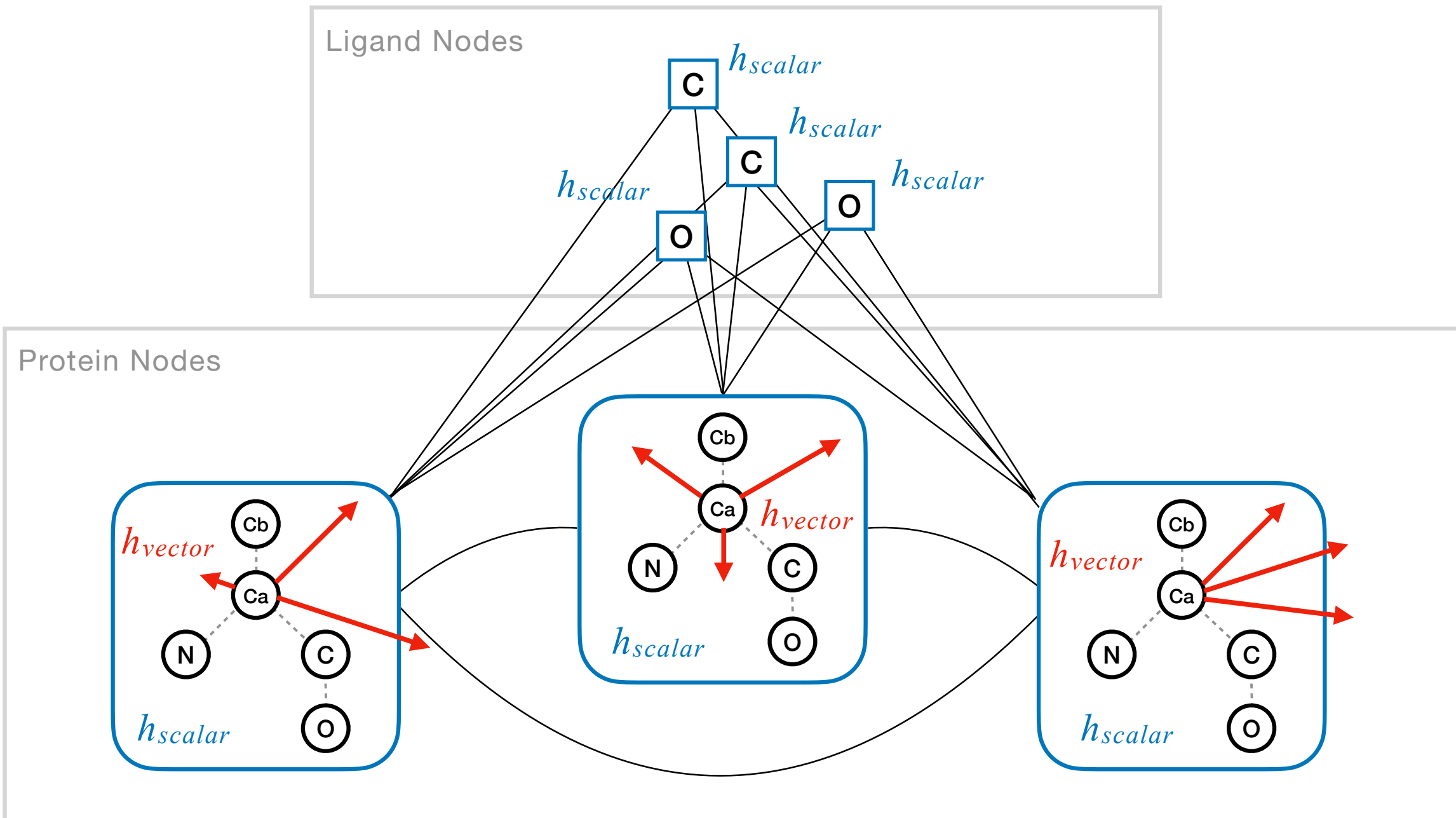

Supplementary Fig. 43. Protein nodes store equivariant vector representations. Geometric vector perceptron (GVP) layers are used to learn both scalar and vector representations to encode residue orientation on all protein nodes in the LASErMPNN architecture. The vector orientations are initialized using the normalized displacement vectors pointing from the Cα carbon to the N, Cβ, C, and O atoms within each frame and are updated by each encoder and decoder layer. Ligand nodes store only scalar representations. Edges denoted as black lines store a scalar edge embedding e_{ij} which are initialized with RBF encoded distances between connected nodes.

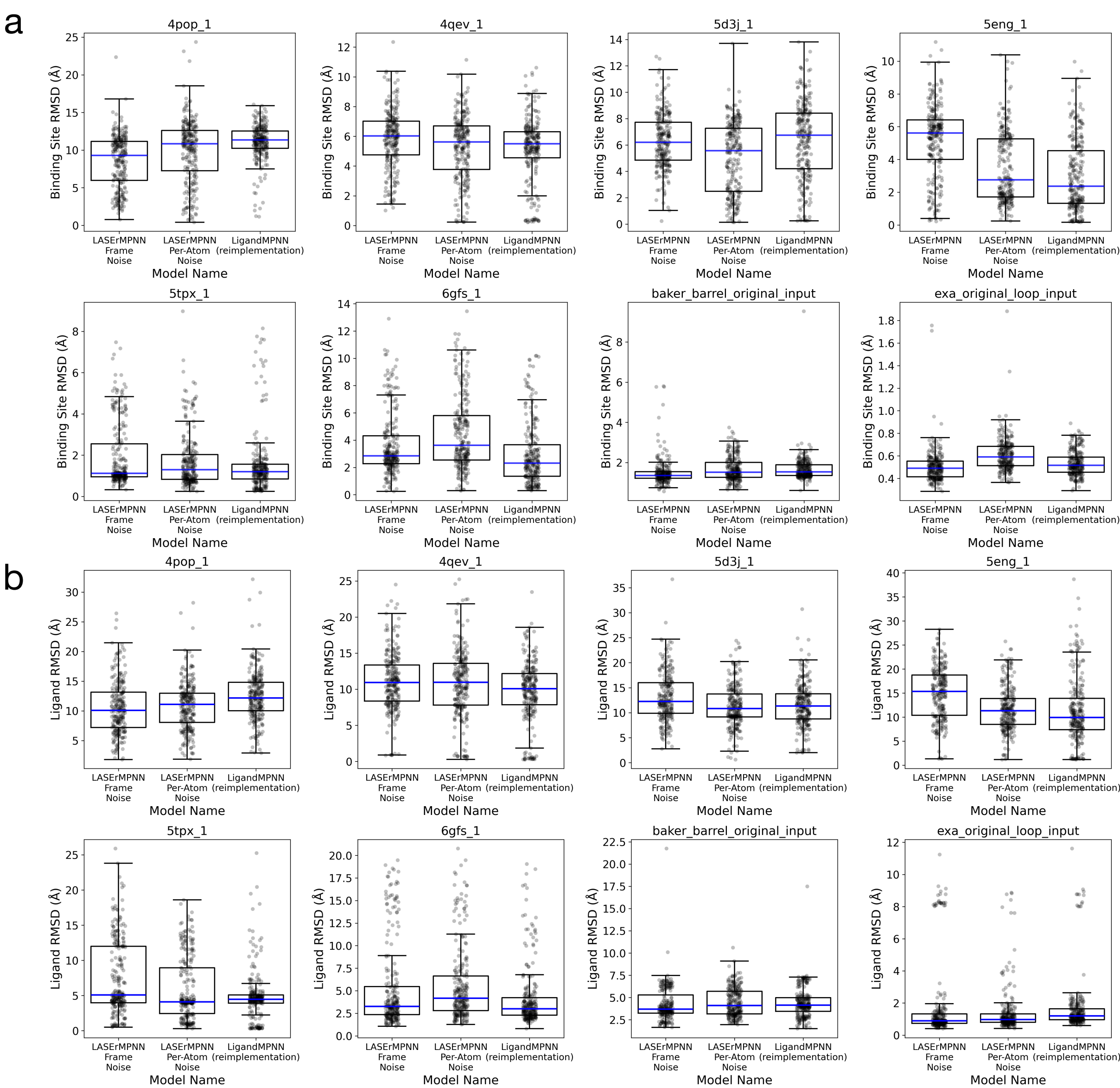

Supplementary Fig. 44. LASErMPNN and LigandMPNN models perform similarly by self-consistency RMSDs on a small test set. a, AlphaFold3 predicted binding site residue Cα RMSD distributions for LASErMPNN models trained with different protein backbone noising strategies and a reimplementation of the LigandMPNN architecture trained on the same dataset. **b**, AlphaFold3 predicted ligand RMSD distributions for LASErMPNN models trained with different protein backbone noising strategies and a reimplementation of the LigandMPNN architecture trained on the same dataset. Median values are highlighted in blue. Baker_barrel_original_input is the published design model used as input for the design of the de novo β-barrel that bound DHFBI ligand[18]. N=250 sequences were sampled per target from each sequence design model.

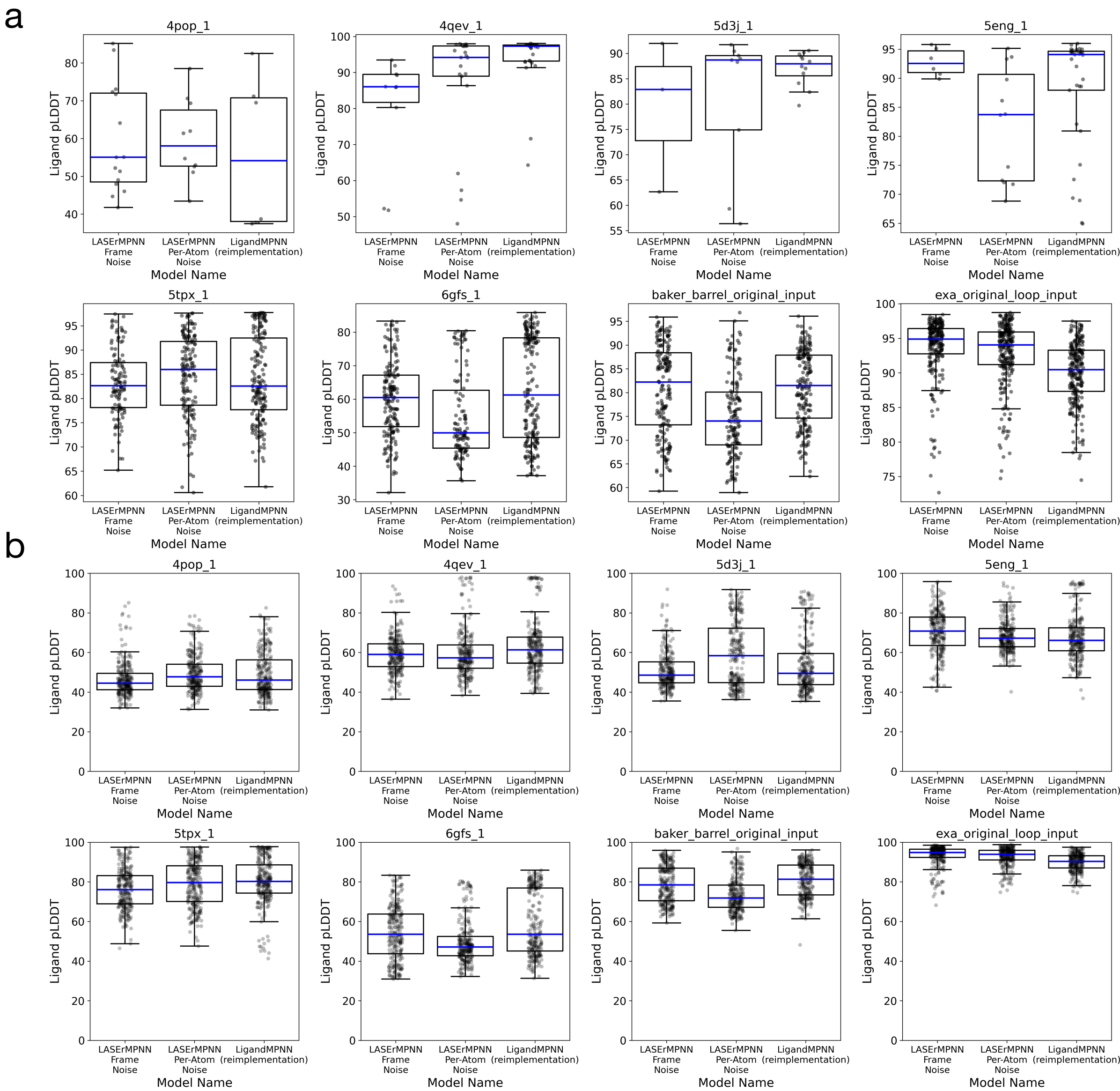

Supplementary Fig. 45. LASErMPNN and LigandMPNN models generate sequences with similar AlphaFold3 ligand pLDDT distributions on a small test set. **a**, AlphaFold3 predicted ligand pLDDT distributions only over self-consistent predictions (ligand RMSD < 5.0 Å, binding site Cα RMSD < 5.0 Å). **b**, AlphaFold3 predicted ligand pLDDT distributions for LASErMPNN models trained with different protein backbone noising strategies and a reimplementation of the LigandMPNN architecture trained on the same dataset. Median values are highlighted in blue. Baker_barrel_original_input is the published design model used as input for the design of the de novo β-barrel that bound DHFBI ligand[18]. N=250 sequences were sampled per target from each sequence design model.

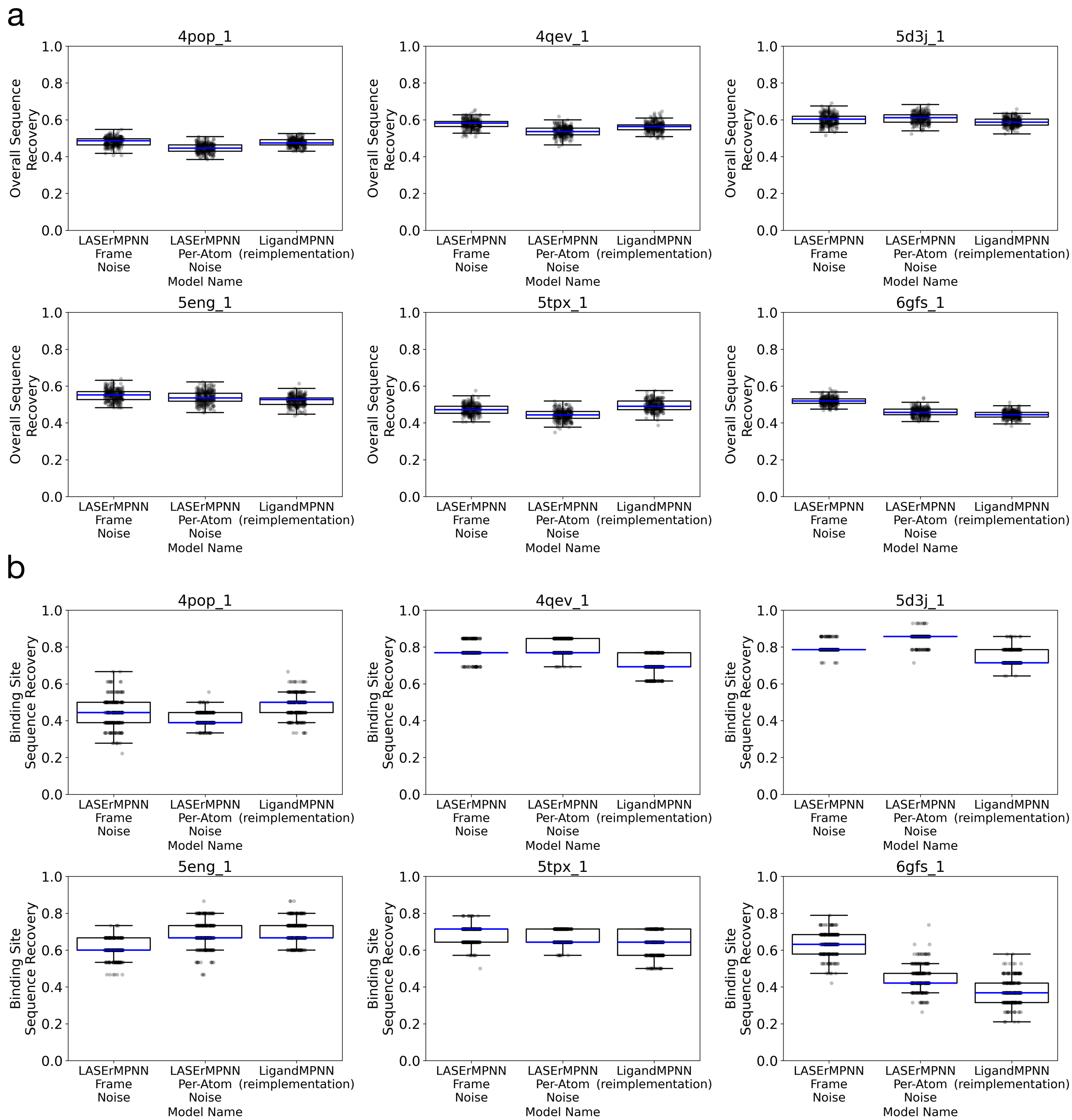

Supplementary Fig. 46. LASErMPNN and LigandMPNN models generate sequences with similar sequence recoveries over a small test set. a, Overall sequence recovery distributions for single chain test set of ligand binding proteins. **b**, Distributions of binding-site sequence recovery of single-chain test set of ligand-binding proteins. Median values are highlighted in blue. N=250 sequences were sampled per target from each sequence design model.

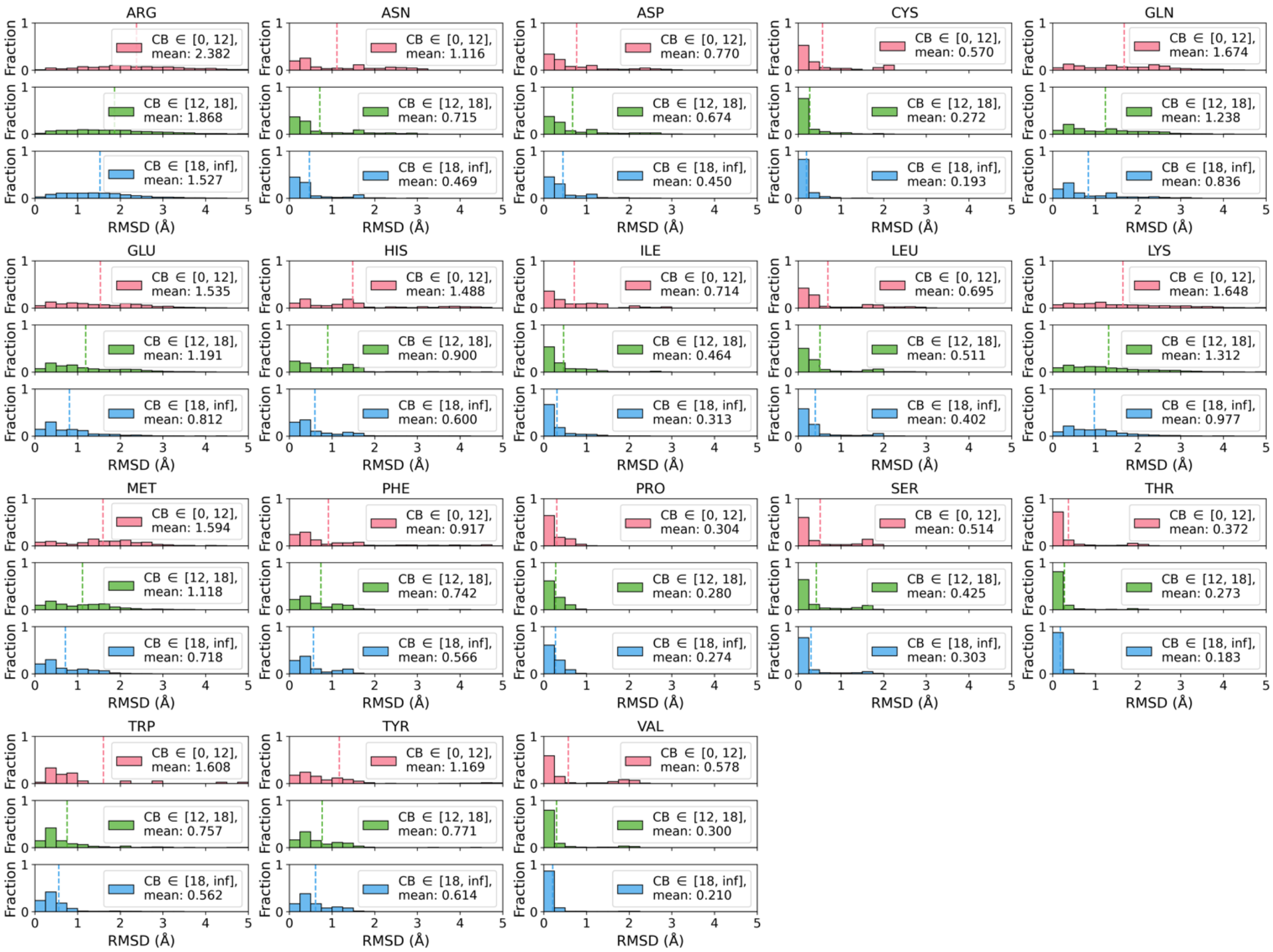

Supplementary Fig. 47. LASErMPNN generates sidechain conformations with low RMSD to crystallographic conformations. LASErMPNN repacked sidechain RMSD distributions as a function of neighboring Cβ atoms (used as a proxy for residue burial) computed over the test set of the LigandMPNN data split (N=317). For all residues, sidechain RMSD tends to decrease as the sidechain becomes more buried.

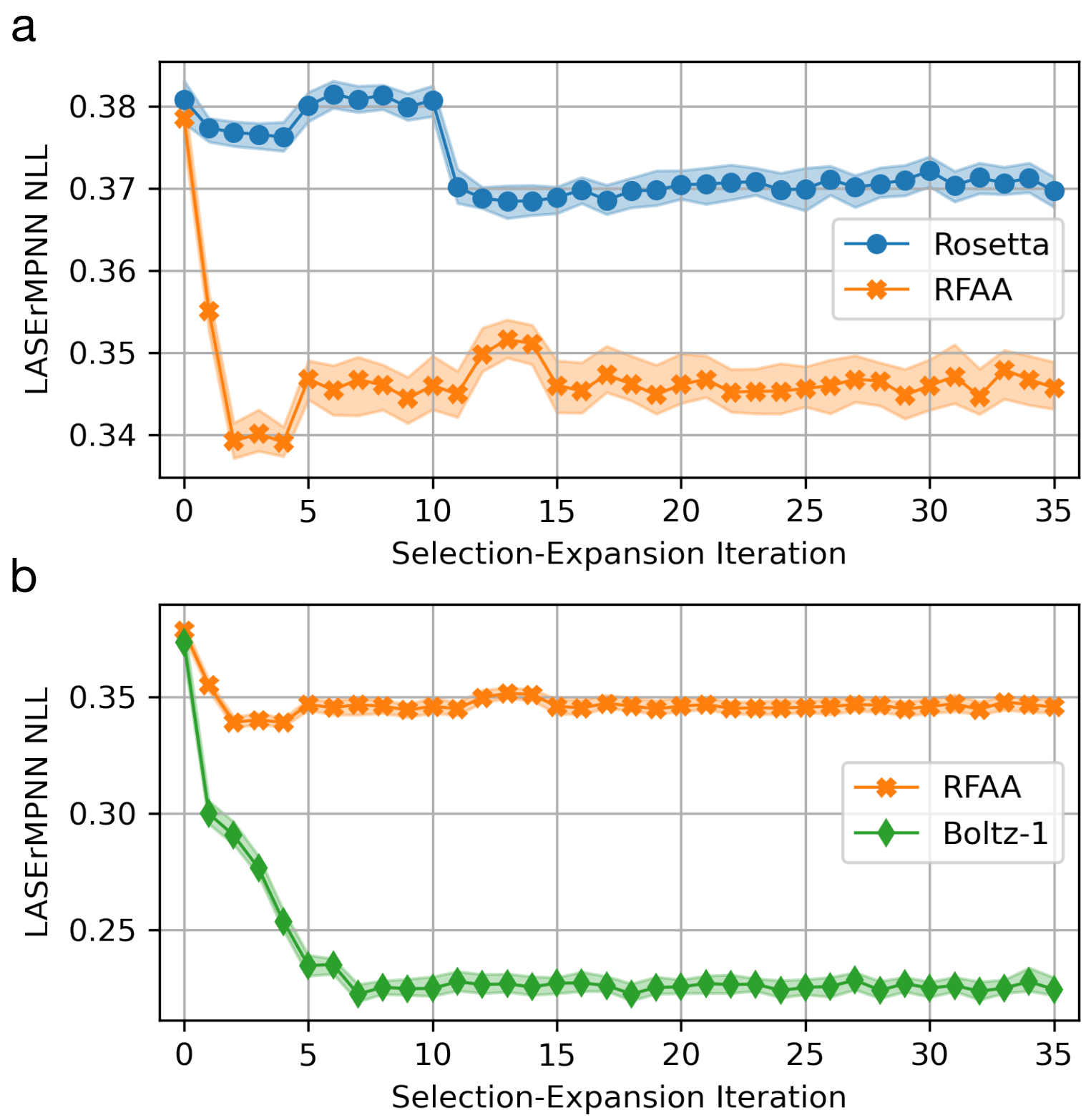

Supplementary Fig. 48. Neural networks for protein–ligand co-structure prediction are more efficient than Rosetta at optimizing LASErMPNN confidence. a, Comparison of LASErMPNN's negative log likelihood (NLL) of designed sequences of a selection–expansion algorithm (using design CR4 as input), which selects the top three poses per iteration by minimum Rosetta ligand energy (flexible protein backbone and ligand trajectory) *vs* a selection-expansion algorithm which maximizes ligand pLDDT from RFAA **b,** Comparison of LASErMPNN's NLL of designed sequences of a selection–expansion algorithm, which selects the top three poses per iteration by maximum ligand pLDDT from RFAA *vs* maximum ligand pLDDT from Boltz-1. All datapoints show the value of the first quartile of LASErMPNN NLL for each selection–expansion trajectory, and shaded regions show 99% confidence intervals on the value of the first quartile computed by bootstrapping (N=100,000) the sampled data for each iteration.

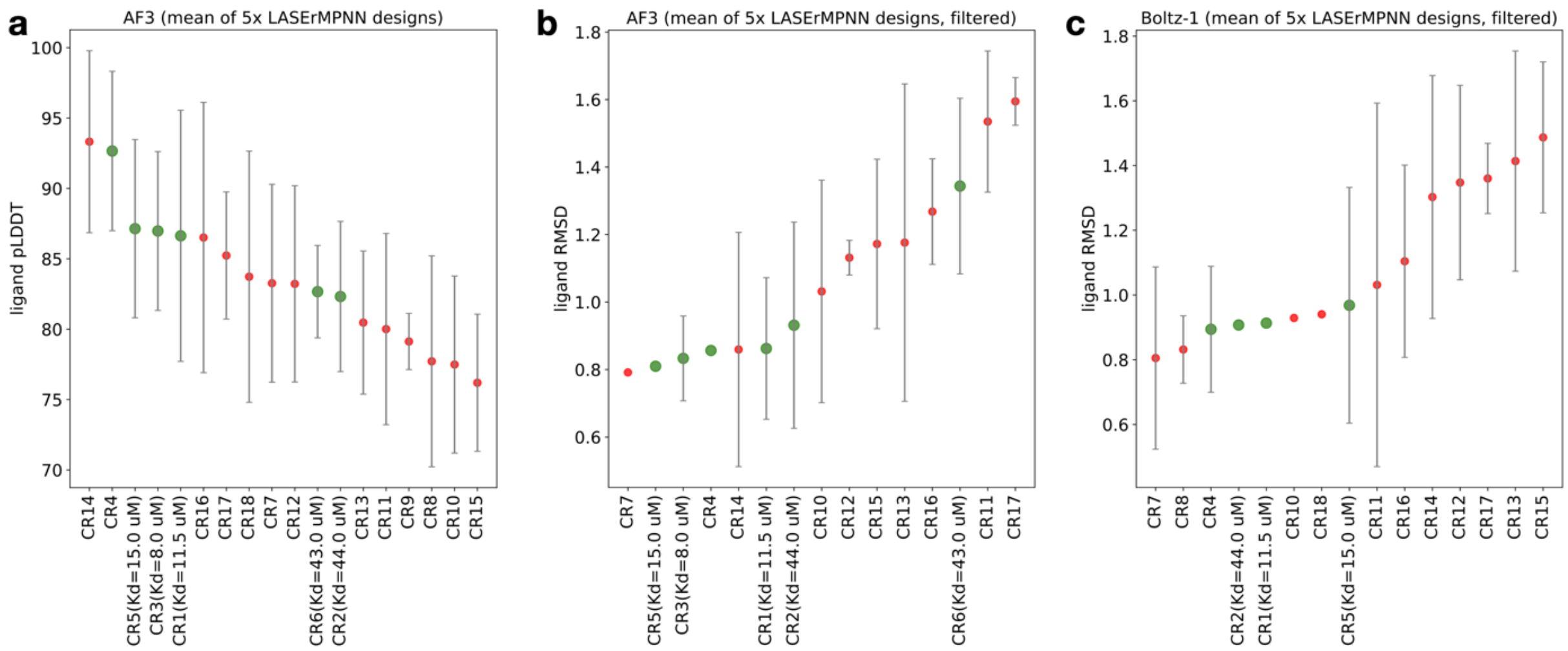

Supplementary Fig. 49. LASErMPNN and structure prediction can be used to rank poses that will lead to binders. Designs from LASErMPNN are sorted by mean ligand pLDDT (left) and ligand heavy-atom RMSD (right, RMSD with respect to the COMBS/Rosetta model after aligning on binding-site residues by Cα atoms) using output from **a**, RoseTTAFold-All Atom (RFAA), **b**, Boltz-1, and **c**, AlphaFold3 (AF3). These results are means and standard deviations from 5 LASErMPNN designs using the COMBS/Rosetta backbones and ligand positions (poses) as input. We discarded the COMBS/Rosetta sequences and used LASErMPNN to design 5 sequences each, which were then folded using the three protein-ligand co-structure prediction models. The designs are labeled on the x-axis (along with any measured dissociation constants). Markers for designs that bound exatecan are colored green and non-binders are colored red. **a**, The mean of ligand pLDDT across structures predicted by AF3 for 5 designed LASErMPNN sequences correlates with binding. **b**, **c**, Mean ligand RMSD computed from AF3 and Boltz-1 also enriches for binders, after removing any predicted structures with low self-consistency (Cα RMSD or ligand RMSD > 2 Å). These metrics can be used to prioritize poses for input into the more computationally intensive NISE algorithm for optimization.

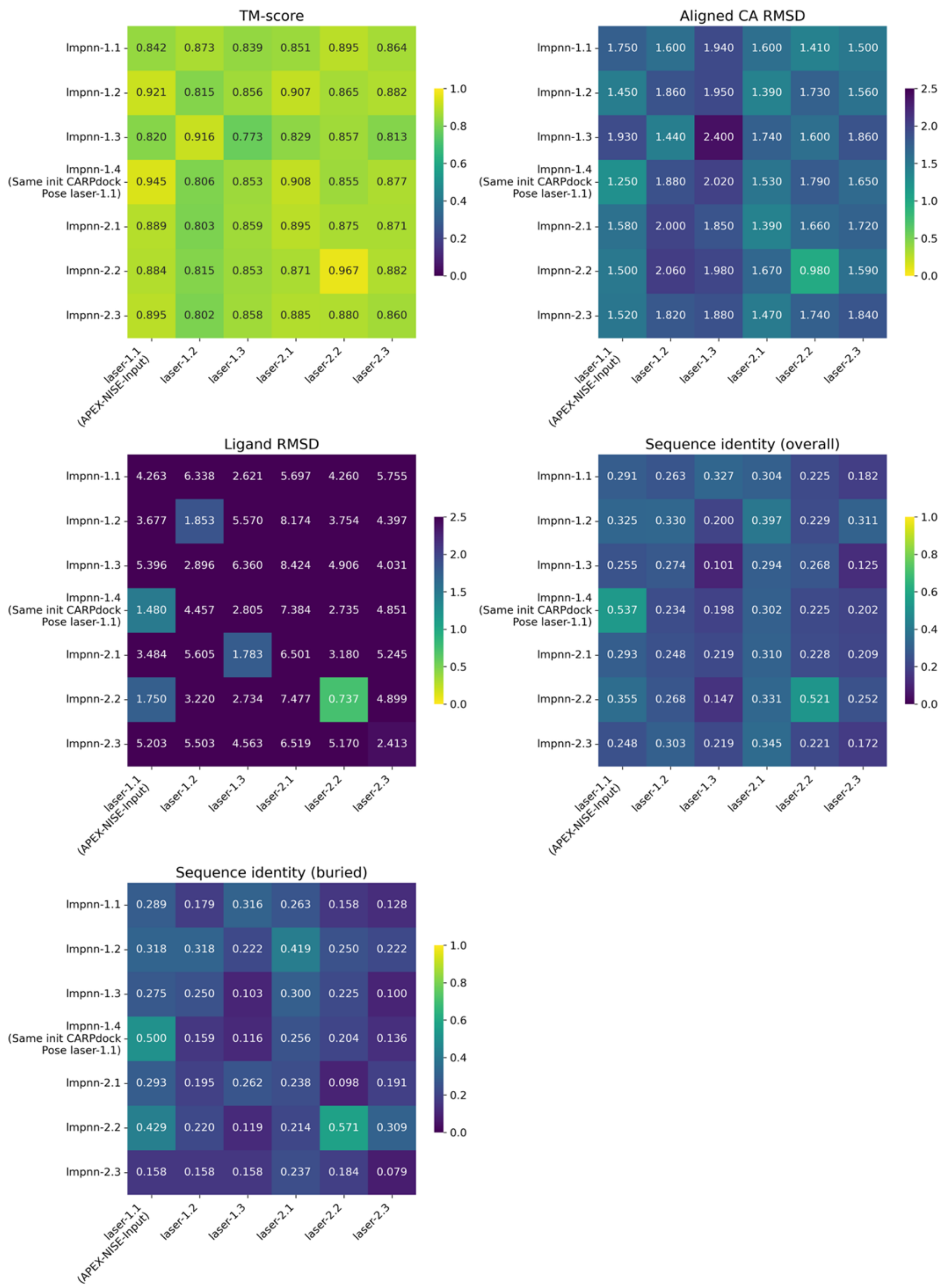

Supplementary Fig. 50. Screening CARPdock poses with LigandMPNN nominates similar poses as with LASErMPNN. Structure and sequence similarity analysis between the top-ranked outputs (ranked by Boltz-2 score) of CARPdock-generated poses for sequences generated by LigandMPNN and LASErMPNN for each of 2 apx conformers. Poses named lmpnn-1.x were generated by LigandMPNN using a CARPdock pose generated by the apx conformer from PDB 6w70 chain C while those named lmpnn-2.x were generated by LigandMPNN using a CARPdock pose generated by the apx conformer from PDB 6w70 chain A. Poses named laser-1.x were generated by LASErMPNN using a CARPdock pose generated by the apx conformer from PDB 6w70 chain C while those named laser-2.x were created by LASErMPNN using a CARPdock pose generated using the apx conformer from PDB 6w70 chain A. The fourth ranked pose generated by LigandMPNN was generated from the same CARPdock pose as laser-1.1, which was the input to the NISE trajectory that generated APEX, indicating LigandMPNN is similarly able to nominate high quality poses generated by CARPdock. Aligned Cα RMSD refers to Cα RMSD taken over residues that have been matched by TM-align since the apx–NTF2 poses from CARPdock have varied amino acid sequence lengths due to docking into different NTF2 backbones.

Supplementary Tables

Supplementary Table 1. Characterized exatecan-binding protein sequences

Design name	Design method	Binding to exatecan (K_d)*	Protein sequence
CR1	COMBS/Rosetta	Yes, 11.5 (10.4–12.8) μM	DKLMEELERALREAEKASEELEKLARTASSVTTLEELLEMAKKMGEVGKKLQEIIKKI<br>ETLWRRIWKEVAAQGNEDALKVLAEFYAALKKWKEVGEKGGKIGEKLLKALNAGNREE<br>AKEALEEYHTAQAEADEARTWLKQAFKKLQEA
CR2	COMBS/Rosetta	Yes, 44 (38–51) μM	DKLLEELERALREAEKASRELEKLARYLESLTTLEEILEAIYKMGAVGKKLQEILKKI<br>KEIWEKIWKEVAAQGNEKALKVLKEFYEALEKWERVGEKGGKIGDKMLKAVQAGNKEE<br>VKEAIEEYKTAQAEADEARADLWTALEKLKRA
CR3	COMBS/Rosetta	Yes, 7.9 ± 0.7 μM	DELLETLIKLFETLAKLGMEANAVFRAAEEAARRGDQDALTYYKKVHQEIAKKIEEVL<br>EKIWRVLETIERALRSINEPRAEKMLELLEKLKKALAKYETIHEQAKRVFEAARDYEE<br>FLALLKIMQKLSTLADQAAKEMKTLLDQLLNT
CR4<br>(NISE input)	COMBS/Rosetta	Yes (aggregated)	DELLEALEKAFRELAKVGMTANKIFREMEEAARRGDEDRLTDGKKKHQDAAKKMEAIL<br>EKIWRILETIERWLRNIDEERAATMLELLTKLKRALEKYERIHETFKRIIEAARTAEE<br>FLALLKILQELSKAADEAAKEIQYLFTQLLNT
CR5	COMBS/Rosetta	Yes, 15.0 ± 1.9 μM	DELWETLIKLFTELAKAGMEANKVFREAEEYARRGDQDALTKALKIHKAIAKKIEEIL<br>EKIWRVLETIERMLRSINEPTAEKLLEVLAKLKKALAKYEKIHEEAIRIFDAARDYEE<br>FLALLKEMQTASTKADQAAKEIKAILDTLLNI
CR6	COMBS/Rosetta	Yes, 43 (34–52) μM	DELLKTLIALFKALAEAGMYLNTIFRQAEEAARRGDEDALTKYLKIHQEGAKKAEEIL<br>EKIWRVLETIERTLRSINEPRAEKMLELLEKLKKALEKYERIHEKAKRIFEAARTAEE<br>FLALLKEMQTASKEADEAAKEIKTLLDQLLST
CR7	COMBS/Rosetta	No	DKLWEELEKLLRYAEKLSETLEKLARYLESLTELDAMREAAKKMGEVGKRLQEVIKAI<br>ETAWEKIWKEAAAKGDEDLLKVLKEFYEALEKWKRAGEKGGKIGEKLKKAIDSGDRKA<br>ATEAIEEYKEAQAEADEARTWLKQAFEKAQRA
CR8	COMBS/Rosetta	No	DELMKRIEEWLKKLWEVFNTMVKTADEVNKITTEEEARKGSDKLLEIVKEMLEILKTI<br>ETLYRQLKEELDKRVANGDEKAEKALKALEKAIRALQKVQELVTKYEKASRRAKKNPD<br>DEEAVKAGSEIGKQIETALEELQRAFEELKRT
CR9	COMBS/Rosetta	No	EELMRRIEELLKRLKKLFEEMVKTADEVNKITTEEEALKGSWKLFNILQEMLTVVKQI<br>KELANQLYEELAKRANNGDEKAYKALKALEKFITALERLEDALTKYEKASRRALKNPH<br>DEEAVKTGSELGKEIEKLLEELLRAFEELKRT
CR10	COMBS/Rosetta	No	EEIMRKIEEWLKRLIKVFEELVKTADEVNKITTEEEARKGSDKLLKILKTMLDIIYTI<br>LALARELEEELAKRKNNGDEKADKMLKALAKFISALYKLLELAQKYEEASRRAKENPD<br>DEEAVKTGSELGKQMETALKEALKAFEELKRT
CR11	COMBS/Rosetta	No	DELLKRLEEIMRRIEEVIRTLGELAERAQRAETLEELLTYSKQLKEHAKELEELLKEA<br>LTVLKQIEKSLEPEQISKAADAIKKLKDALKEMLKILEAWKKASTEGEKAWRSGDEEK<br>ASKISELGDRLGEALLRALAKALEALEKLLRI
CR12	COMBS/Rosetta	No	DEILKRMEEVLRTIEAVMKKLGELAERAQRAETLEELLSYSKELKEEAKYLEELIKTL<br>LKLLAAFKNSLEPEQKKKMSEALTRLEQYIRALLKALKEWKEASKQGEEAWRSGDEEK<br>ASKISELGDRIGEKMLRILAAAFEAFELLVRI
CR13	COMBS/Rosetta	No	DELVKRLEELIRRVEALMKKLGELGEAAQRAQTLEELLTLSKQLKEAAKELEELLKEI<br>LKVIYEIIKSLEPDQIKRMSEAITKALTALTELMKLLKEWKKLSKEGEEAWRSGDEET<br>ASKISELGDRAGEKMLRALEKLKKAFEEFVRA
CR14	COMBS/Rosetta	No	MDIWKALEEALRELDKLLQELGKAGQAAAEAQNEEEYSDWDEKIERILQKLLKALEKI<br>KRLLEAAKKALESIKSKDPYAGKILEELLKTFEELLKWFETLLRKYISIHEEGRRKGA<br>TPELQKQADEAGKALEHALALLKELLKRIKDT
CR15	COMBS/Rosetta	No	LDIWKAMEKALRELDKALKELGEAGQAAAEAQEEEEYSKYDERIERILQWILKLLELL<br>KRLLEAAQRSADEIKNKDPTRGKIIEELLTTFEDLLRKFETALRKYIAIHHRGRNEGA<br>TPELQSQADKAGKELEHYLKLLKELMKRIKET
CR16	COMBS/Rosetta	No	DKLLETLEKLSRYLEKLAKELEELITYMFSLKTLEEILKAIEQILEVLKKLGEAGKKI<br>QEVLKTIEKELRNQGNEEALKALKKYKEAGKEGSEAGKEIWEALKKAEEAARSGNWEE<br>LTEAFEEIKTGLTKYKQAHTKAQQALEELKRA
CR17	COMBS/Rosetta	No	DKLMETLEKLSRELEAAAKEMEELIKYMESLKTIEEMLKAIKKILEALKKLGEIGKKI<br>QEVWETIWKEAANQGNEELLKALKKYYEAGKEGSEAGKELEEALTKLKKALESGDEDA<br>AKDALRLIREALTKYKQAHTKGQQALEEAKRA
CR18	COMBS/Rosetta	No	DKLLEELEKASRELEKLAKELEEAISYMESLKTIEEMLKAIKKILEILKKLGEVGKKI<br>QEILKKIWEMLARQGNEEALKAAKKYMDAGKKGSDAGKEIEEALKKLEEALRSGDEES<br>AKDALRLIKEGLTKYKQAHAEAQEALEDLKRA
EPIC	NISE	Yes, 0.12 ± 0.03 μM	EDLIKKIKEIIKKLKELGAELEKALDRLKKAAEAKDIEAAEEALAELLKLQEEMLVLL<br>EELKATVEKLLEEVRKIDTPAATKKVEVLEKLLDAINKMIDIVKEGLKILKEVKDLEE<br>LEEAIKEVEVLVKEADKYAAEIEEEYAKYLAI
NISE2	NISE	Yes, 9.7 (9.1–10.4) μM	VDLNQKIGEILSRLEKLGGEMTEALEKLKNAVKEKDIEAAIKALKQLLELQAETLKLL<br>NELLETVKQLLEQVKKIDTPKAQKKVEILEKFIESVEKLIAVNQEGLKILEEVKDLEE<br>LAEAIKKVEELRKEADVLAGKVIQDYEAYQAI
NISE3	NISE	Yes, 3.2 (3.0–3.4) μM	SDLIKKIDEILSKLKELGAELEQALRELEDAVRARDRAAADEALARLLALQREQLKLL<br>RELLDTVKELKKIVEEIDTPLAKEKVKILTQLLTKIEQLIAINERGLAILEATRDLAE<br>LRAAIAEVRRLVVRADEKADQILAEYDKFKAL
NISE4	NISE	Yes, 16.8 (15.8–17.7) μM	KKVLDQLKESLEKLKEVGEYAKALIDAAFKAAEDGDQAAYDAAKAAIAQAQAKQLSII<br>DKIKEILEALTKELKEIDSEKAEEKLILLSELQAALAKYEAIIVKTQAIVAESKDTET<br>FLKNKEKIEKLVDEADKAAAEIMELYDRLLEA
EPIC Q51N	NISE	Yes, 8.0 ± 1.6 nM	EDLIKKIKEIIKKLKELGAELEKALDRLKKAAEAKDIEAAEEALAELLKLNEEMLVLL<br>EELKATVEKLLEEVRKIDTPAATKKVEVLEKLLDAINKMIDIVKEGLKILKEVKDLEE<br>LEEAIKEVEVLVKEADKYAAEIEEEYAKYLAI
EPIC M97L	NISE	Yes, 7.4 ± 0.7 nM	EDLIKKIKEIIKKLKELGAELEKALDRLKKAAEAKDIEAAEEALAELLKLQEEMLVLL<br>EELKATVEKLLEEVRKIDTPAATKKVEVLEKLLDAINKLIDIVKEGLKILKEVKDLEE<br>LEEAIKEVEVLVKEADKYAAEIEEEYAKYLAI
EPIC Q51N<br>M97L	NISE	Yes, 1.2 ± 0.2 nM	EDLIKKIKEIIKKLKELGAELEKALDRLKKAAEAKDIEAAEEALAELLKLNEEMLVLL<br>EELKATVEKLLEEVRKIDTPAATKKVEVLEKLLDAINKLIDIVKEGLKILKEVKDLEE<br>LEEAIKEVEVLVKEADKYAAEIEEEYAKYLAI

* Parentheses indicate 95% confidence intervals of 1000 bootstrapped fits (residuals of optimal fit sampled with replacement). Other error bars are standard deviation of optimal fits from biological replicates.

Supplementary Table 2. RFAA metrics for the EPIC lineage from the NISE trajectory

NISE Iter	Sequence	Protein pLDDT	Ligand (Exatecan) pLDDT	Protein RMSD (Å)	Ligand RMSD (Å)	Sequence Identity to Prev (Overall)	Sequence Identity to Prev (Buried)	Camptothecin pLDDT	Irinotecan pLDDT
**NISE Input**	DELLEALEKAFRELAK<br>VGMTANKIFREMEEAA<br>RRGDEDRLTDGKKKHQ<br>DAAKKMEAILEKIWRI<br>LETIERWLRNIDEERA<br>ATMLELLTKLKRALEK<br>YERIHETFKRIIEAAR<br>TAEEFLALLKILQELS<br>KAADEAAKEIQYLFTQ<br>LLNT	0.820	0.556	-	-	-	-	0.845	0.597
**0**	TSLLQELKKLLEELKA<br>QGEKAGKLVNRAKIAA<br>AEGNEEELEECKEALK<br>KAIAEEEAILEEIKKT<br>LEEINNYLLKINSEEA<br>ENQIKLLDDLIAALEE<br>FENIVKKAKALIEKSK<br>NCEEFLKLEKEIDELT<br>KKADEAAKKIIKLYNE<br>LLSE	0.872	0.878	0.949	0.864	0.399	0.586	0.435	0.504
**7**	NELLEELKNLLEELKE<br>VGEKAKELVNAAKVAA<br>ENGDQEAFNACKEELK<br>KAVKEMKSILKEIEEK<br>LTSLREHLESIDSDKA<br>AAQLKLLDELEKALAK<br>FKAIVDKTVALIEESK<br>DCETALKLLDDINELV<br>KEADEAAAEIMELFTK<br>LLDT	0.878	0.870	0.843	0.751	0.520	0.741	0.422	0.697
**10**	KKLLEQLDEEIEEVKE<br>IGEKLEELLGELKQAA<br>REGDEEKAQECLTKIR<br>EGLKEMIEKIKKIDSI<br>IKQIQEKLKKDKSKEA<br>KQKLVILGELKDALKR<br>YKAIEARAKEIIDASK<br>DVDQAKEAIGEIEELV<br>AAAEREADEILQLWKE<br>LKET	0.901	0.877	0.539	0.657	0.365	0.483	0.754	0.807
**11**	AKKMAKVDRIIAELDK<br>IGKELETALEKLEKAA<br>QKKDEEAAKKALKELQ<br>GLIAKMNVLLDELDEE<br>IQEILEEEKKKNTPKA<br>QQKIKVLEELQAAIGQ<br>LKELVQKGQSILAETK<br>DIKELLEAIEKIKGLV<br>VQANAAAAEVQAKFAE<br>LMAI	0.918	0.892	0.609	0.550	0.311	0.466	0.759	0.596
**12**	VDKEKQIDEIIAELTE<br>IGAELEKALEELKEAV<br>ENRDIAEAEKALKKLL<br>ELIEEQLVLLEELNET<br>VESLKKEVEKEETEKA<br>KEKVEILDKLLESIEK<br>LQAIVEEGKKILEEVK<br>DLDELAEAIGEVEELV<br>VKADAEAKKVVDEYKK<br>LKAI	0.943	0.900	0.692	0.429	0.432	0.707	0.697	0.731
**22 (EPIC)**	EDLIKKIKEIIKKLKE<br>LGAELEKALDRLKKAA<br>EAKDIEAAEEALAELL<br>KLQEEMLVLLEELKAT<br>VEKLLEEVRKIDTPAA<br>TKKVEVLEKLLDAINK<br>MIDIVKEGLKILKEVK<br>DLEELEEAIKEVEVLV<br>KEADKYAAEIEEEYAK<br>YLAI	0.911	0.896	0.440	0.726	0.581	0.776	0.777	0.716

Highlighted residues in ‘Sequence’ column indicate residues that switched identity from previous sequence in lineage.

Supplementary Table 3. Boltz-2 metrics for the APEX lineage from the NISE trajectory										
**NISE Iter**	**Sequence**	**Protein pLDDT**	**Ligand (Apixaban) pLDDT**	**Protein RMSD (Å)**	**Ligand RMSD (Å)**	**Apixaban P(bind)**	**Sequence Identity to Prev (Overall)**	**Sequence Identity to Prev (Buried)**	**Exatecan pLDDT**	**Exatecan P(bind)**
**NISE Input**	APSLEEQCIALGREF<br>VAAANAGDVEAMRSM<br>LAPDATMTLSDGTKL<br>TKKEDIVAYYKEGYE<br>NGYRIEVDILSVEVI<br>GDNEAVATAKGTIKS<br>SKTGEVIELETTAKF<br>VLVDGRVYIKKLEGK<br>ELP	0.9790	0.9782	-	-	0.9903	-	-	0.5732	0.1862
**0**	APSLQSQAETLGKEF<br>IDAANEGDVEKMRSM<br>LAPDATMKLDDGTEL<br>KKREDIVEYYKDGYE<br>NGYKVEVTIESVEVV<br>GSDRAVATAEGTVRS<br>TKTGEVVKLRTKAEF<br>RLVNGQVYILRLEGE<br>RLP	0.9793	0.9826	0.2721	0.3098	0.9873	0.6666	0.8780	0.9198	0.2037
**1**	SPSLKDQAEQLGKEF<br>VDAANEGDTEKVKSM<br>LAPDATMTLSDGTEL<br>KKKEDIVDYYSKGYK<br>EGKKVTVDIKSVKVI<br>GRNKALAEADGTVTS<br>TKTGEVVKLRTKAEF<br>ILKEGKVFIKKLEGE<br>ELP	0.9816	0.9844	0.1719	0.2762	0.9928	0.6991	0.8536	0.5598	0.2725
**3**	SPSLRERARDLGREF<br>VEAANAGDTERVSSM<br>LAPDATMTLQDGTKL<br>SRKDDIVQFYKDGYE<br>AGLKVTVDIQSVVVL<br>GSDRALAEADGTVTS<br>KKTGEIVHLKTSAVF<br>RLLNGRVYIRELSGE<br>RLP	0.9783	0.9857	0.2378	0.4266	0.9982	0.6504	0.8780	0.5124	0.2821
**4**	TPSLEERARNLGQEF<br>VEAANSGDTETVRSM<br>LSPDATMTLADGTKL<br>TEKDQIVEYYRQGHE<br>AGLTVEVQIQSVEQL<br>GPDEALATADGVVRS<br>RKTGQVTHLRTSARF<br>KLINGQVYIRELSGK<br>QQP	0.9781	0.9864	0.2270	0.2193	0.9978	0.6829	0.9024	0.7537	0.2891
**5**	TPSLEDQARELGRQF<br>VEAANSGDTEKVESM<br>LSPDATMVLADGTQL<br>TKKEQIVDYYKRGYE<br>AGLTVKVEIQSVQSL<br>GPDTALATADGVVES<br>KKTGEVKKLQTSAVF<br>RRINGKLYIRELSGK<br>DQP	0.9831	0.9877	0.2418	0.1524	0.9982	0.7398	0.9024	0.5002	0.4082
**7**	EPSLEEQARDLGRDF<br>VAAANSGDTEKVRSM<br>LSPDATMTLADGTEL<br>NKKEEIVDYYREGYL<br>GGLTVRVDVQSVDSL<br>GPDVALATARGVVRS<br>AATGEVLRLRTSARF<br>EKIDGRLYIKELSGE<br>LLP	0.9821	0.9880	0.1751	0.1661	0.9986	0.7154	0.9512	0.5178	0.3683
**8 (APEX)**	LPSLEDQARTLGKKF<br>VQAANSGNTDQVRSM<br>LAPDATMTLADGTQL<br>NKRDDIVNYYRDGYE<br>GGLTVEVSIQSVESL<br>GPDRALATARGTVRS<br>AATGEVLTLETSAEF<br>VRIDGTLYIKKLSGT<br>LLP	0.9725	0.9753	0.4662	0.4364	0.9890	0.7479	0.9024	0.5289	0.2582
Highlighted residues in ‘Sequence’ column indicate residues that switched identity from previous sequence in lineage.										

Supplementary Table 4. Characterized apixaban-binding protein sequences

Design name	Design method	Binding to apixaban-FITC (K_d)*	Binding to apixaban (K_d)*	Protein sequence
APEX	NISE	0.99 (0.72–1.28) nM	80 (54–122) pM	LPSLEDQARTLGKKFVQAAN<br>SGNTDQVRSMLAPDATMTLA<br>DGTQLNKRDDIVNYYRDGYE<br>GGLTVEVSIQSVESLGPDRA<br>LATARGTVRSAATGEVLTLE<br>TSAEFVRIDGTLYIKKLSGT<br>LLP
apx2.2	NISE	250 (215–320) nM	12 (0.26–20) nM	APSLQSQAETLGKEFIDAAN<br>EGDVEKMRSMLAPDATMKLD<br>DGTELKKREDIVEYYKDGYE<br>NGYKVEVTIESVEVVGSDRA<br>VATAEGTVRSTKTGEVVKLR<br>TKAEFRLVNGQVYILRLEGE<br>RLP
apx2.3	NISE	83 (69–108) nM	8.6 (1.9–13.7) nM	MSESEQRQIALDYAQALNDG<br>DVESLLSLFAPDAVITLADG<br>TKLTSLEQMRQAALAFFSQN<br>KDVKVEVLSLSFDGSTVTGT<br>AKMTYHVGDTKYEADVTGTF<br>TFENGKIKTLTYTQTTKEV
apx2.4	NISE	367 (334–458) nM	9.6 (0.001–25) nM	PSNEEMREIAKEYAQALNDG<br>DIDALLSLFAPDATIVTSDG<br>TVLTSLEEMRKHAEDFFDEH<br>KNVKVEILSLEFKGNTVTGT<br>ARMTFDVGDDRYEAQVTATF<br>VFRDGKIQKMEYTQTTKHE
apx2.5	NISE	99 (88–126) nM	54 (38–66) nM	MTKEELEKRAVEMVHQFFDA<br>VNEGDIEEVRRILAEDYEMV<br>DGTGKKYSKEEAIEIWEEAA<br>ANGIKAKVTEVKIIKSTEDE<br>VVARIDVEIYNGATGETTTR<br>TFEATFKLVDNELKGVQGRL<br>VKEP
apx2.6	NISE	> 100 μM	n.d.	MSEEEALKLLRTFVDAVNSG<br>NLELLRSMADPNATVTLPDG<br>SVLSTVDQGLEFVQKFYEEG<br>WHLTVRDITVEHLPDSVKIS<br>GVGIGTNRKTGATSEMRGVI<br>YYDPVSKKILSTVLEDLP

*Parentheses indicate 95% confidence intervals of 1000 bootstrapped fits (residuals of optimal fit sampled with replacement). Uncertainty at low K_d values for apx2.4 is likely due to the few data points at the sharp transition in the competition experiment (see Extended Data Fig. 9).

Supplementary Table 5. NISE generated exatecan binder designs.								
**Design**	**Ligand pLDDT**	**Protein pLDDT**	**Ligand RMSD**	**Protein RMSD**	**Rosetta PackStat**	**# Ligand BUNs**	**BUNs Score**	**K_d**
**NISE 2**	0.908	0.902	0.272	0.280	0.641	2.0	23.0	9.7 μM
**NISE 3**	0.929	0.903	0.626	0.177	0.593	3.0	17.0	3.2 μM
**NISE 4**	0.859	0.875	0.672	0.959	0.596	3.0	28.0	16.8 μM
**EPIC**	0.911	0.896	0.440	0.726	0.649	4.0	16.0	0.12 μM

Supplementary Table 6. Chemical structures of ligands used in binding experiments
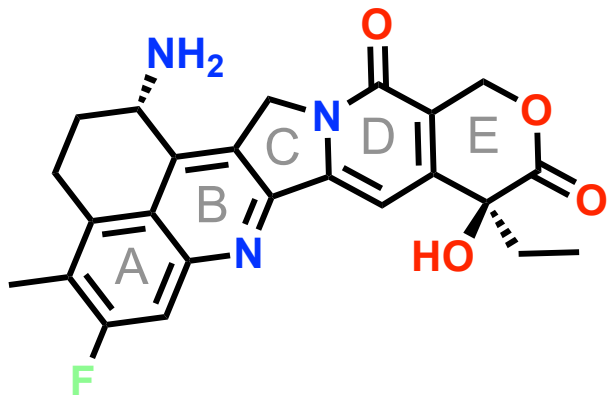 exatecan
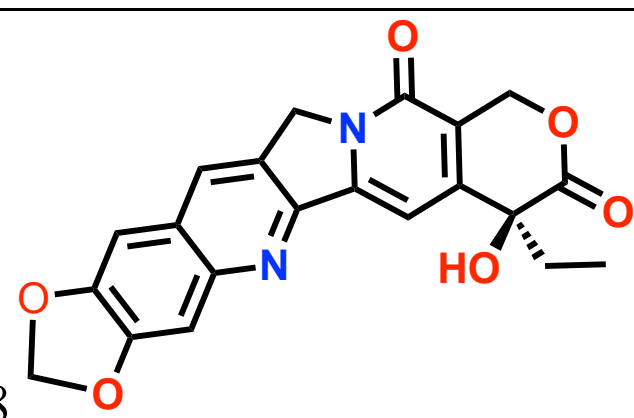 FL118
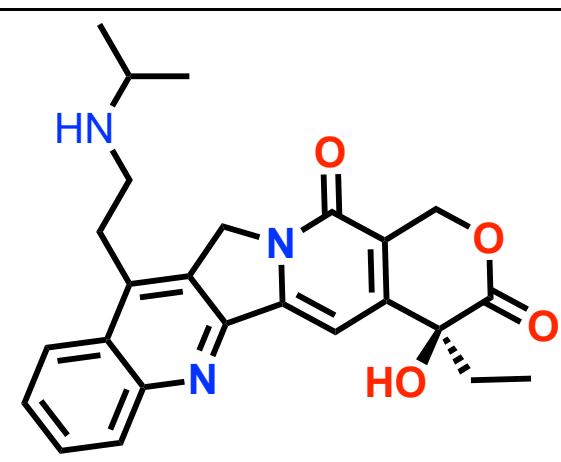 belotecan
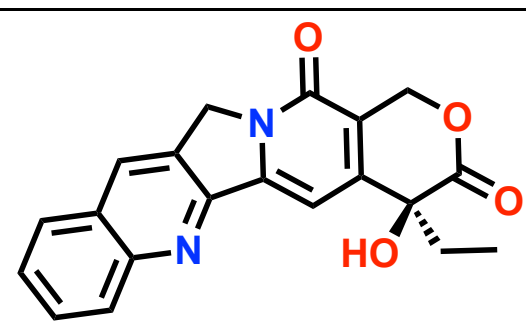 camptothecin
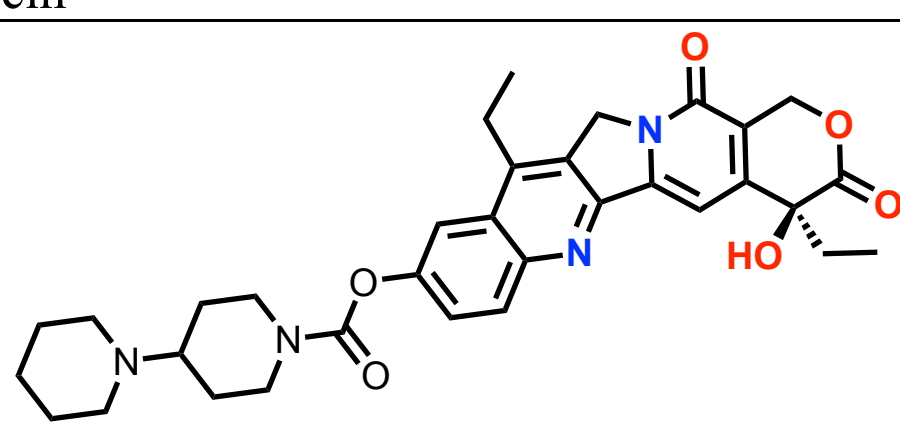 irinotecan
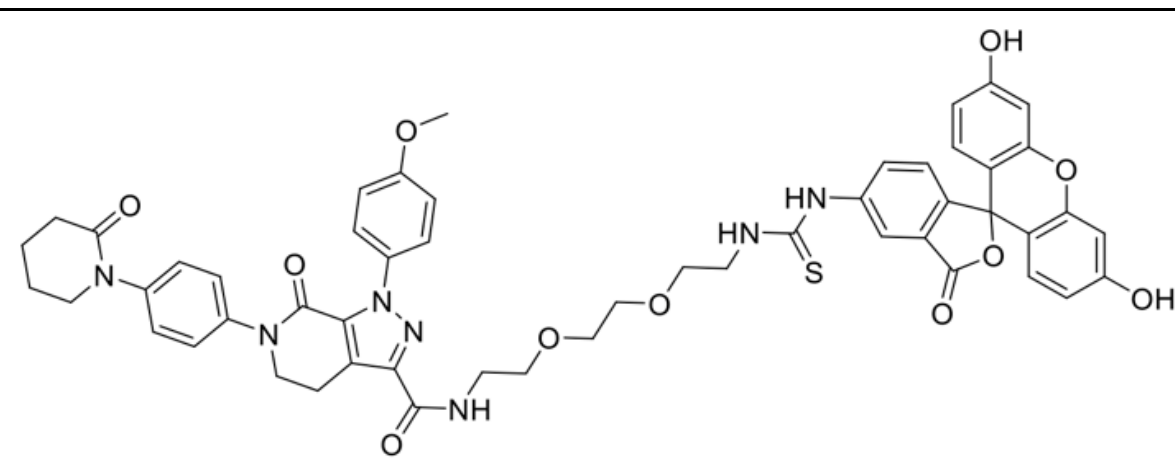 apixaban-peg-fitc
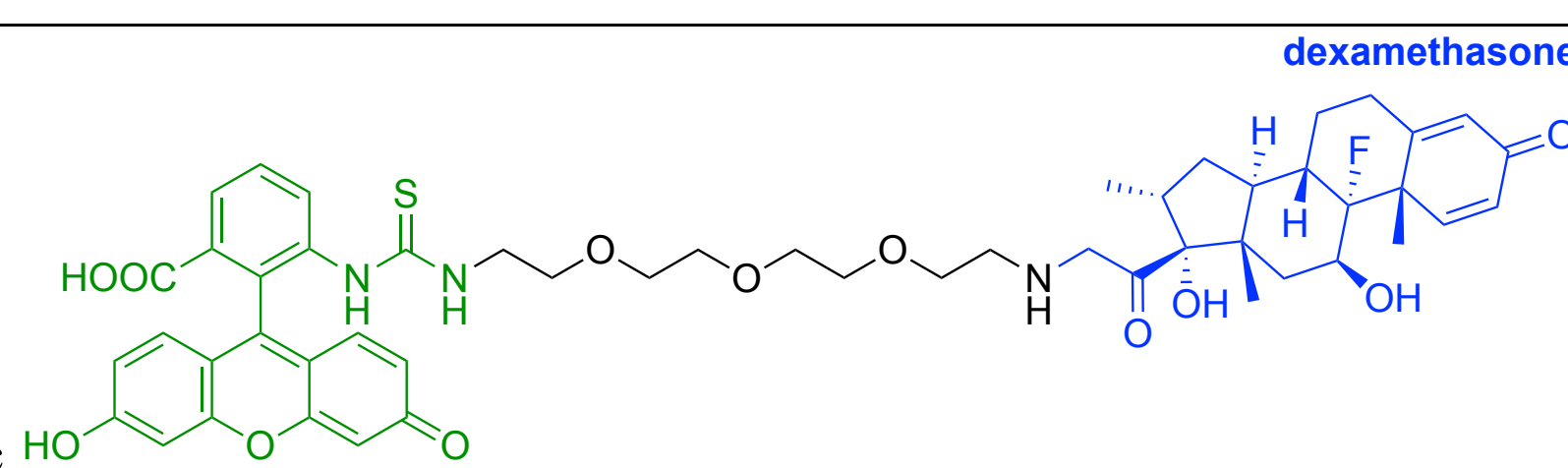 dexamethasone-peg-fitc

**Supplementary Table 7. Structural comparison of 6 top-ranked designs from NISE trajectory using LigandMPNN vs 6 top-ranked designs from NISE trajectory using LASErMPNN (starting from the same input pose that produced APEX)**						
**Top 6 LASErMPNN Design**	**Closest Top 6 LigandMPNN Design***	**TM Score**	**Ca RMSD (Å) over TM-Aligned Residues**	**Ligand RMSD (Å)**	**Sequence Identity (Overall)**	**Sequence Identity (Buried)**
**rank_001**	rank_006	0.9739	0.69	0.834	0.5041	0.7273
**rank_002 (APEX)**	rank_006	0.9748	0.68	0.7426	0.5041	0.7045
**rank_003 (apx-2.2)**	rank_003	0.9661	0.83	0.6166	0.5772	0.7442
**rank_004**	rank_006	0.9515	1.02	1.1889	0.4797	0.6591
**rank_005**	rank_002	0.9684	0.78	0.7914	0.5447	0.6977
**rank_006**	rank_006	0.9722	0.71	0.8156	0.4797	0.6364
* The closest LigandMPNN design in the top 6 was selected as the design with the highest buried residue sequence identity to each LASErMPNN design ranked in the top 6. See Comparing LASErMPNN and LigandMPNN within NISE for more information.						

Supplementary Table 8. Ranking ablations: top-ranked designs from NISE trajectory that generated EPIC, selected by re-ranking designs independently by RFAA confidence or self-consistency metrics.

criterion-rank	Ligand RMSD (Å)	Ligand pLDDT	Ca RMSD (Å)	Protein pLDDT	BUNs Score	sequences
**ligand_rmsd-1**	0.136	0.916	0.678	0.895	14	SDLEKEIDELLTKLKELGKELEEALAALRKAVAEKD<br>REAAEAARDRLLALQDETLILLKELKETVEKLKREV<br>QKIDTPRAEEKVRILDDLLKSIEKLIDIVKEGRTIL<br>QDVKDLEELAKALEKVEKLVEEANKEAKNVVKNHDK<br>YKEI
**ligand_rmsd-2**	0.141	0.932	0.387	0.9	14	VDLEEKIDEIIEKLIEIGKELEQALKDLTKAVEEQD<br>VEKAKKALKALLELLEEELKLLGELEETVKELLEKI<br>KKIKTPEAKEKVEILEKMLEAIEKLTGIVKKGLEIL<br>KEVKNLEELQKAVKQVEKLVKEADIEAEKVIKLHDE<br>FKAI
**ligand_rmsd-3**	0.148	0.923	0.583	0.885	11	KDLEEEIDKILDELKKLGKKLEEALEKLKKAVEEEN<br>IEAAEKALKELIELQEKELELLEELKKTVESLLEEI<br>KKKDTPEAKEKVLILENLLKAVDKLIKIVKKGLEII<br>EKTKDMEELKEAIKEIEELVEKANIEAEKIIEEHNK<br>LKSI
**ligand_plddt-1**	0.91	0.946	0.561	0.877	11	KELLEEIGRIINKLIVIGQKLETAYSKLKTAAEKNN<br>IEAAEKALAELKKLIDELTVELEKLKKLLEELLEKQ<br>KELNSEEAEKYVKLIEEALDAVEQLEKIVDQGKKII<br>EKVTDLEELKTALNEIEVLQNQANVEAAKLVELYNE<br>LLKY
**ligand_plddt-2**	0.429	0.943	0.692	0.9	10	VDKEKQIDEIIAELTEIGAELEKALEELKEAVENRD<br>IAEAEKALKKLLELIEEQLVLLEELNETVESLKKEV<br>EKEETEKAKEKVEILDKLLESIEKLQAIVEEGKKIL<br>EEVKDLDELAEAIGEVEELVVKADAEAKKVVDEYKK<br>LKAI
**ligand_plddt-3**	0.282	0.941	0.598	0.907	13	VDKIKKIGEILKKLIELGKELEAALKALKEACEEEN<br>TTAAEAALEKLLALIEEELVLLGELKKTVEELLEEI<br>KAIDTPEAKQKVEILDELIKAIEKLIEIVEEGLKIL<br>KETSDLTELREAIKKVEELVVEADIEANKIIDYYKE<br>FKAI
**protein_rmsd-1**	0.373	0.915	0.207	0.893	10	VDLDAKVDSIVSELTEIGKKLEKALEKLKKAVDNKD<br>VQEAEKALKELLELIKKLLELLKELKKTVEKLLEEV<br>KKISSPEAKQKVEILEKLLKAIKKLIEIVEEGLKIL<br>EECKNLEELKAAIKEVEELVKEADKEAKKVVEELEK<br>FKAL
**protein_rmsd-2**	0.369	0.896	0.213	0.898	10	VDKEAVIDDILDKLQTLGKELTQALKDLEKAVEEKN<br>VDEAEKALKKLLALIDETLVLLGELRKTVEDLLEEV<br>LKIESPKAQAKVEVLTQMLKAIDKLIDIVKKGKQIL<br>EEVKNLDELKEAIKKVQKLKIEADQEADKVLREWAA<br>YKAL
**protein_rmsd-3**	0.428	0.917	0.218	0.899	11	IDLLAEIDEILKHLKKLGKEAEQALEELKQACEQDD<br>VDKAKEALAKLLALIDEMLDLLDKLKETVEKLLEKI<br>KKINSPEAKKKVEILEKLLEAIDKLIDIVKEGKDIL<br>EKVKDIDELRIAIKEVEKLVEEANVEAKKVIELYNE<br>LQAI
**protein_plddt-1**	0.508	0.936	0.303	0.918	11	EALLEKIDTILDELKLIGEELEVALAELKAAAEAED<br>VEAAAAAKAKLLALIKEQKVLLDELKAAVEQLLAQE<br>QQLDTEEARQKVEVLQELLAAIDDLEVIVDIGQAIL<br>EATKDLAELKEAIAIVEKLVVEADKQADKVLKLYDE<br>LKAI
**protein_plddt-2**	0.39	0.924	0.377	0.916	12	VDLESEIGSILNQLEELGSQLSAALAALTAAVAAKD<br>KAAADAALAELLALIEQELILLSQLLETVKQLKEKV<br>LKINSPKANKKVEILDKMLEAIEKLIDIVQEGLTIL<br>REVKDLAELAAAIERVAALVAEADVIAKEVLDNYKK<br>FKDI
**protein_plddt-3**	0.323	0.933	0.302	0.915	12	VDLDAVIDRILEELQTLGEEAEAALAELKAAVAEKD<br>VAAAEAALARLLAIQQRMLTLLGELHATVSKKLEEI<br>KKINSPAAKKRVEILEALLEAIAALIGINQQGLEIL<br>QAVRDLEELAEAIKQVEELVAQADEHAAAVIALYDA<br>DKQI
**EPIC**	0.726	0.911	0.44	0.896	9	EDLIKKIKEIIKKLKELGAELEKALDRLKKAAEAKD<br>IEAAEEALAELLKLQEEMLVLLEELKATVEKLLEEV<br>RKIDTPAATKKVEVLEKLLDAINKMIDIVKEGLKIL<br>KEVKDLEELEEAIKEVEVLVKEADKYAAEIEEEYAK<br>YLAI

Supplementary Table 9. Structural comparison of top-ranked LigandMPNN CARPdock screen poses vs top-ranked LASErMPNN CARPdock screen poses

LASErMPNN Pose	Closest LigandMPNN Pose*	TM-Score	Aligned Ca RMSD (Å)	Ligand RMSD (Å)	Sequence Identity (overall)	Sequence Identity (buried)
**laser-1.1 (APEX-NISE-Input)**	lmpnn-1.4 (same initial CARPdock pose as laser-1.1)	0.9453	1.250	1.480	0.5372	0.5000
**laser-1.2**	lmpnn-1.2	0.8154	1.860	1.853	0.3303	0.3182
**laser-1.3**	lmpnn-1.1	0.8386	1.940	2.621	0.3271	0.3158
**laser-2.1**	lmpnn-1.2	0.9073	1.390	8.174	0.3966	0.4186
**laser-2.2**	lmpnn-2.2	0.9665	0.980	0.737	0.5207	0.5714
**laser-2.3**	lmpnn-2.2	0.8817	1.590	4.899	0.2521	0.3095

* The closest LigandMPNN pose was selected as the design with the highest buried residue sequence identity to each LASErMPNN pose (from among the poses analyzed in Supp. Fig. 50). See Re-screening CARPdock Poses with LigandMPNN for more information.

Supplementary References

1. Dauparas, J., Anishchenko, I., Bennett, N., Bai, H., Ragotte, R. J., Milles, L. F., Wicky, B. I. M., Courbet, A., de Haas, R. J., Bethel, N., Leung, P. J. Y., Huddy, T. F., Pellock, S., Tischer, D., Chan, F., Koepnick, B., Nguyen, H., Kang, A., Sankaran, B., Bera, A. K., King, N. P. & Baker, D. Robust deep learning-based protein sequence design using ProteinMPNN. *Science* **378**, 49–56 (2022).
2. Eastman, P., Behara, P. K., Dotson, D. L., Galvelis, R., Herr, J. E., Horton, J. T., Mao, Y., Chodera, J. D., Pritchard, B. P., Wang, Y., De Fabritiis, G. & Markland, T. E. SPICE, A dataset of drug-like molecules and peptides for training machine learning potentials. *Sci. Data* **10**, 11 (2023).
3. Landrum, G., Tosco, P., Kelley, B., Rodriguez, R., Cosgrove, D., Vianello, R., Gedeck, P., Jones, G., NadineSchneider, Kawashima, E., Nealschneider, D., Dalke, A., Swain, M., Cole, B., Turk, S., Savelev, A., tadhurst-cdd, Vaucher, A., Wójcikowski, M., Take, I., Walker, R., Scalfani, V. F., Faara, H., Ujihara, K., Probst, D., Lehtivarjo, J., Godin, G., Pahl, A. & Monat, J. rdkit/rdkit: 2025_03_1 (Q1 2025) Release. Preprint at https://doi.org/10.5281/zenodo.15115844.
4. Brody, S., Alon, U. & Yahav, E. How Attentive are Graph Attention Networks? *arXiv [cs.LG]* (2021).
5. Veličković, P., Cucurull, G., Casanova, A., Romero, A., Liò, P. & Bengio, Y. Graph Attention Networks. *arXiv [stat.ML]* (2017).
6. Jing, B., Eismann, S., Suriana, P., Townshend, R. J. L. & Dror, R. Learning from protein structure with geometric vector perceptrons. *arXiv [q-bio.BM]* (2020).
7. Ahdritz, G., Bouatta, N., Kadyan, S., Jarosch, L., Berenberg, D., Fisk, I., Watkins, A. M., Ra, S., Bonneau, R. & AlQuraishi, M. OpenProteinSet: Training data for structural biology at scale. *arXiv [q-bio.BM]* (2023).
8. Corso, G., Deng, A., Fry, B., Polizzi, N., Barzilay, R. & Jaakkola, T. Deep confident steps to new pockets: Strategies for docking generalization. *arXiv [q-bio.BM]* (2024).
9. Dauparas, J., Lee, G. R., Pecoraro, R., An, L., Anishchenko, I., Glasscock, C. & Baker, D. Atomic context-conditioned protein sequence design using LigandMPNN. *Nat. Methods* 1–7 (2025).
10. Word, J. M., Lovell, S. C., Richardson, J. S. & Richardson, D. C. Asparagine and glutamine: using hydrogen atom contacts in the choice of side-chain amide orientation. *J. Mol. Biol.* **285**, 1735–1747 (1999).
11. Steinegger, M. & Söding, J. MMseqs2 enables sensitive protein sequence searching for the analysis of massive data sets. *Nat. Biotechnol.* **35**, 1026–1028 (2017).
12. Fry, B. & Polizzi, N. LASErMPNN Training Dataset (REDUCE Protonated PDB). (2025) doi:10.5281/ZENODO.15035128.
13. Polizzi, N. F. & DeGrado, W. F. A defined structural unit enables de novo design of small-molecule-binding proteins. *Science* **369**, 1227–1233 (2020).
14. Krishna, R., Wang, J., Ahern, W., Sturmfels, P., Venkatesh, P., Kalvet, I., Lee, G. R., Morey-Burrows, F. S., Anishchenko, I., Humphreys, I. R., McHugh, R., Vafeados, D., Li, X., Sutherland, G. A., Hitchcock, A., Hunter, C. N., Kang, A., Brackenbrough, E., Bera, A. K., Baek, M., DiMaio, F. & Baker, D. Generalized biomolecular modeling and design with RoseTTAFold All-Atom. *Science* **384**, eadl2528 (2024).

15. Wohlwend, J., Corso, G., Passaro, S., Reveiz, M., Leidal, K., Swiderski, W., Portnoi, T., Chinn, I., Silterra, J., Jaakkola, T. & Barzilay, R. Boltz-1 democratizing biomolecular interaction modeling. *bioRxiv* 2024.11.19.624167 (2024) doi:10.1101/2024.11.19.624167.
16. Polizzi, N. *Designs and analysis code for Neural Iterative Selection-Expansion paper*. (Zenodo, 2026). doi:10.5281/ZENODO.18308430.
17. Eastman, P., Galvelis, R., Peláez, R. P., Abreu, C. R. A., Farr, S. E., Gallicchio, E., Gorenko, A., Henry, M. M., Hu, F., Huang, J., Krämer, A., Michel, J., Mitchell, J. A., Pande, V. S., Rodrigues, J. P., Rodriguez-Guerra, J., Simmonett, A. C., Singh, S., Swails, J., Turner, P., Wang, Y., Zhang, I., Chodera, J. D., De Fabritiis, G. & Markland, T. E. OpenMM 8: Molecular dynamics simulation with machine learning potentials. *J. Phys. Chem. B* **128**, 109–116 (2024).
18. Dou, J., Vorobieva, A. A., Sheffler, W., Doyle, L. A., Park, H., Bick, M. J., Mao, B., Foight, G. W., Lee, M. Y., Gagnon, L. A., Carter, L., Sankaran, B., Ovchinnikov, S., Marcos, E., Huang, P.-S., Vaughan, J. C., Stoddard, B. L. & Baker, D. De novo design of a fluorescence-activating β-barrel. *Nature* **561**, 485–491 (2018).
19. Fleishman, S. J., Leaver-Fay, A., Corn, J. E., Strauch, E.-M., Khare, S. D., Koga, N., Ashworth, J., Murphy, P., Richter, F., Lemmon, G., Meiler, J. & Baker, D. RosettaScripts: a scripting language interface to the Rosetta macromolecular modeling suite. *PLoS One* **6**, e20161 (2011).
20. An, L., Said, M., Tran, L., Majumder, S., Goreshnik, I., Lee, G. R., Juergens, D., Dauparas, J., Anishchenko, I., Coventry, B., Bera, A. K., Kang, A., Levine, P. M., Alvarez, V., Pillai, A., Norn, C., Feldman, D., Zorine, D., Hicks, D. R., Li, X., Sanchez, M. G., Vafeados, D. K., Salveson, P. J., Vorobieva, A. A. & Baker, D. Binding and sensing diverse small molecules using shape-complementary pseudocycles. *Science* **385**, 276–282 (2024).
21. Lee, G. R., Pellock, S. J., Norn, C., Tischer, D., Dauparas, J., Anishchenko, I., Mercer, J. A. M., Kang, A., Bera, A. K., Nguyen, H., Brackenbrough, E., Sankaran, B., Goreshnik, I., Vafeados, D., Roullier, N., Han, H. L., Coventry, B., Haddox, H. K., Liu, D. R., Yeh, A. H.-W. & Baker, D. Small-molecule binding and sensing with a designed protein family. *Nat. Commun.* 1–12 (2026).
22. Zhou, J. & Grigoryan, G. Rapid search for tertiary fragments reveals protein sequence-structure relationships. *Protein Sci.* **24**, 508–524 (2015).
23. Vonrhein, C., Flensburg, C., Keller, P., Sharff, A., Smart, O., Paciorek, W., Womack, T. & Bricogne, G. Data processing and analysis with the autoPROC toolbox. *Acta Crystallogr. D Biol. Crystallogr.* **67**, 293–302 (2011).
24. Adams, P. D., Afonine, P. V., Bunkóczi, G., Chen, V. B., Echols, N., Headd, J. J., Hung, L.-W., Jain, S., Kapral, G. J., Grosse Kunstleve, R. W., McCoy, A. J., Moriarty, N. W., Oeffner, R. D., Read, R. J., Richardson, D. C., Richardson, J. S., Terwilliger, T. C. & Zwart, P. H. The Phenix software for automated determination of macromolecular structures. *Methods* **55**, 94–106 (2011).
25. Casañal, A., Lohkamp, B. & Emsley, P. Current developments in Coot for macromolecular model building of Electron Cryo-microscopy and Crystallographic Data. *Protein Sci.* **29**, 1069–1078 (2020).
26. Yeh, A. H.-W., Norn, C., Kipnis, Y., Tischer, D., Pellock, S. J., Evans, D., Ma, P., Lee, G. R., Zhang, J. Z., Anishchenko, I., Coventry, B., Cao, L., Dauparas, J., Halabiya, S., DeWitt, M., Carter, L., Houk, K. N. & Baker, D. De novo design of luciferases using deep learning. *Nature* **614**, 774–780 (2023).

27. Lu, L., Gou, X., Tan, S. K., Mann, S. I., Yang, H., Zhong, X., Gazgalis, D., Valdiviezo, J., Jo, H., Wu, Y., Diolaiti, M. E., Ashworth, A., Polizzi, N. F. & DeGrado, W. F. De novo design of drug-binding proteins with predictable binding energy and specificity. *Science* **384**, 106–112 (2024).
28. Gu, S., Smith, M. S., Yang, Y., Irwin, J. J. & Shoichet, B. K. Ligand strain energy in large library docking. *J. Chem. Inf. Model.* **61**, 4331–4341 (2021).
29. Polizzi, N. F., Wu, Y., Lemmin, T., Maxwell, A. M., Zhang, S.-Q., Rawson, J., Beratan, D. N., Therien, M. J. & DeGrado, W. F. De novo design of a hyperstable non-natural protein-ligand complex with sub-Å accuracy. *Nat. Chem.* **9**, 1157–1164 (2017).
30. Eastman, P., Pritchard, B. P., Chodera, J. D. & Markland, T. E. Nutmeg and SPICE: Models and data for biomolecular machine learning. *J. Chem. Theory Comput.* **20**, 8583–8593 (2024).
31. Watson, J. L., Juergens, D., Bennett, N. R., Trippe, B. L., Yim, J., Eisenach, H. E., Ahern, W., Borst, A. J., Ragotte, R. J., Milles, L. F., Wicky, B. I. M., Hanikel, N., Pellock, S. J., Courbet, A., Sheffler, W., Wang, J., Venkatesh, P., Sappington, I., Torres, S. V., Lauko, A., De Bortoli, V., Mathieu, E., Ovchinnikov, S., Barzilay, R., Jaakkola, T. S., DiMaio, F., Baek, M. & Baker, D. De novo design of protein structure and function with RFdiffusion. *Nature* **620**, 1089–1100 (2023).
32. Pacesa, M., Nickel, L., Schellhaas, C., Schmidt, J., Pyatova, E., Kissling, L., Barendse, P., Choudhury, J., Kapoor, S., Alcaraz-Serna, A., Cho, Y., Ghamary, K. H., Vinué, L., Yachnin, B. J., Wollacott, A. M., Buckley, S., Westphal, A. H., Lindhoud, S., Georgeon, S., Goverde, C. A., Hatzopoulos, G. N., Gönczy, P., Muller, Y. D., Schwank, G., Swarts, D. C., Vecchio, A. J., Schneider, B. L., Ovchinnikov, S. & Correia, B. E. One-shot design of functional protein binders with BindCraft. *Nature* **646**, 483–492 (2025).
