# Supplementary material for: Zero-shot design of drug-binding proteins via neural iterative selection−expansion
Source: Nature. 2026 Jun 24;656(8126):237–49. doi: 10.1038/s41586-026-10670-w (PMC13441969; doi:10.1038/s41586-026-10670-w)
Supplement: Supplementary file 2 [file 41586_2026_10670_MOESM2_ESM.pdf]

Corresponding author(s): Nicholas F Polizzi

Last updated by author(s): 05/11/2026

Reporting Summary

Nature Portfolio wishes to improve the reproducibility of the work that we publish. This form provides structure for consistency and transparency in reporting. For further information on Nature Portfolio policies, see our Editorial Policies and the Editorial Policy Checklist.

Statistics

For all statistical analyses, confirm that the following items are present in the figure legend, table legend, main text, or Methods section.

n/a	Confirmed	
☐	☒	The exact sample size (*n*) for each experimental group/condition, given as a discrete number and unit of measurement
☐	☒	A statement on whether measurements were taken from distinct samples or whether the same sample was measured repeatedly
☐	☒	The statistical test(s) used AND whether they are one- or two-sided<br>*Only common tests should be described solely by name; describe more complex techniques in the Methods section.*
☒	☐	A description of all covariates tested
☒	☐	A description of any assumptions or corrections, such as tests of normality and adjustment for multiple comparisons
☐	☒	A full description of the statistical parameters including central tendency (e.g. means) or other basic estimates (e.g. regression coefficient) AND variation (e.g. standard deviation) or associated estimates of uncertainty (e.g. confidence intervals)
☐	☒	For null hypothesis testing, the test statistic (e.g. *F*, *t*, *r*) with confidence intervals, effect sizes, degrees of freedom and *P* value noted<br>*Give P values as exact values whenever suitable.*
☒	☐	For Bayesian analysis, information on the choice of priors and Markov chain Monte Carlo settings
☒	☐	For hierarchical and complex designs, identification of the appropriate level for tests and full reporting of outcomes
☒	☐	Estimates of effect sizes (e.g. Cohen's *d*, Pearson's *r*), indicating how they were calculated

Our web collection on statistics for biologists contains articles on many of the points above.

Software and code

Policy information about availability of computer code

Data collection	Neural networks were trained with Python v3.11.11, PyTorch v2.0.1, NVIDIA CUDA v12.4, NumPy v1.26.4, pytorch-cluster v1.6.3, pytorch-scatter v2.1.2. MMseqs2 v15.6f452 was used to cluster PDB chains, ProDy v2.4.0 was used for PDB structure analysis, RDkit v2024.03.5 was used for small molecule analysis, Reduce 4.14.230914 was used to protonate the training dataset. LASErMPNN training and inference code is available at github.com/polizzilab/LASErMPNN. NISE inputs for apixaban binders were generated by CARPdock using the implementation at https://github.com/benf549/CARPdock. Exatecan conformers were built using Avogadro v1.2.0. LASErMPNN code and weights used in this study can be found at doi:10.5281/ZENODO.15297591.
Data analysis	Data analysis used Python v3.11.11, Matplotlib v3.10.1, Seaborn 0.13.2, NumPy v1.26.4. Structure visualizations were created in PyMol v2.6.2. All statistical tests were performed using SciPy v1.16.1. Design and analysis code for the Neural Iterative Selection Expansion algorithm can be found at doi:10.5281/ZENODO.18308430

For manuscripts utilizing custom algorithms or software that are central to the research but not yet described in published literature, software must be made available to editors and reviewers. We strongly encourage code deposition in a community repository (e.g. GitHub). See the Nature Portfolio guidelines for submitting code & software for further information.

Data

Policy information about availability of data

All manuscripts must include a data availability statement. This statement should provide the following information, where applicable:

- Accession codes, unique identifiers, or web links for publicly available datasets
- A description of any restrictions on data availability
- For clinical datasets or third party data, please ensure that the statement adheres to our policy

Coordinates and data files for X-ray crystal structures of exatecan-bound EPIC and exatecan-bound EPIC-Q51N have been deposited in the PDB with accession codes 9NZE (EPIC) and 9NZG (EPIC-Q51N). Other referenced PDB IDs include 6W70, 4JNJ, 4L9K, and 8TN6. All data used to train LASErMPNN are publicly available from the PDB (https://www.rcsb.org/) or the SPICE v2.0.1 dataset (https://zenodo.org/records/10975225). The processed datasets used to train the LASErMPNN model are available at https://zenodo.org/records/17990180 and https://zenodo.org/records/15035128.

Research involving human participants, their data, or biological material

Policy information about studies with human participants or human data. See also policy information about sex, gender (identity/presentation), and sexual orientation and race, ethnicity and racism.

Reporting on sex and gender	N/A
Reporting on race, ethnicity, or other socially relevant groupings	N/A
Population characteristics	N/A
Recruitment	N/A
Ethics oversight	N/A

Note that full information on the approval of the study protocol must also be provided in the manuscript.

Field-specific reporting

Please select the one below that is the best fit for your research. If you are not sure, read the appropriate sections before making your selection.

☒ Life sciences ☐ Behavioural & social sciences ☐ Ecological, evolutionary & environmental sciences

For a reference copy of the document with all sections, see nature.com/documents/nr-reporting-summary-flat.pdf

Life sciences study design

All studies must disclose on these points even when the disclosure is negative.

Sample size	We selected designs based on computational metrics and filters. For NISE, we selected a small set of designs (either 4 or 6) to test if high success rates could be achieved with only a small number of samples. For COMBS designs, we made 16 to sample more broadly, and added 2 more designs using the same input pose that fed NISE for the EPIC binder. The latter 2 were to ensure we tested the idea that the input pose itself wasn't the main contributor to success. Binding experiments reported were either three technical replicates of a single sample or an average of 3 biological replicates. For EPIC and its mutants (Fig. 4c), sample size of biological replicates for binding experiments was n=3 to provide robust estimates of fit parameters. Fig 6 (APEX binder of apixaban) shows data of one sample with global fitting across three separate ligand conditions. Replication of this experiment was successful.
Data exclusions	Beginning with all experimentally determined structures in the RCSB PDB deposited before 06/01/2022, we downloaded mmCIF formatted data for all biological assemblies. We filtered out structures with over 5,000 amino acids or ligand residues and selected structures generated by X-ray crystallography or cryo-electron microscopy with at least 3.5 Å resolution.<br><br>The dataset was then split into clusters of 30% sequence identity using MMseqs2 to reduce redundancy.
Replication	All attempts at replication of binding experiments were successful.
Randomization	No randomization was necessary for our binding experiments.
Blinding	Blinding was not relevant to the binding experiments reported in this study.

Reporting for specific materials, systems and methods

We require information from authors about some types of materials, experimental systems and methods used in many studies. Here, indicate whether each material, system or method listed is relevant to your study. If you are not sure if a list item applies to your research, read the appropriate section before selecting a response.

Materials & experimental systems

n/a	Involved in the study
☒	☐ Antibodies
☒	☐ Eukaryotic cell lines
☒	☐ Palaeontology and archaeology
☒	☐ Animals and other organisms
☒	☐ Clinical data
☒	☐ Dual use research of concern
☒	☐ Plants

Methods

n/a	Involved in the study
☒	☐ ChIP-seq
☒	☐ Flow cytometry
☒	☐ MRI-based neuroimaging

Plants

Seed stocks	*Report on the source of all seed stocks or other plant material used. If applicable, state the seed stock centre and catalogue number. If plant specimens were collected from the field, describe the collection location, date and sampling procedures.*
Novel plant genotypes	*Describe the methods by which all novel plant genotypes were produced. This includes those generated by transgenic approaches, gene editing, chemical/radiation-based mutagenesis and hybridization. For transgenic lines, describe the transformation method, the number of independent lines analyzed and the generation upon which experiments were performed. For gene-edited lines, describe the editor used, the endogenous sequence targeted for editing, the targeting guide RNA sequence (if applicable) and how the editor was applied.*
Authentication	*Describe any authentication procedures for each seed stock used or novel genotype generated. Describe any experiments used to assess the effect of a mutation and, where applicable, how potential secondary effects (e.g. second site T-DNA insertions, mosiacism, off-target gene editing) were examined.*
