# Supplementary material for: Zero-shot design of drug-binding proteins via neural iterative selection−expansion
Source: Nature. 2026 Jun 24;656(8126):237–49. doi: 10.1038/s41586-026-10670-w (PMC13441969; doi:10.1038/s41586-026-10670-w)
Supplement: Supplementary file 3 [file 41586_2026_10670_MOESM3_ESM.pdf]

nature portfolio

# Peer Review File

# Zero-shot design of drug-binding proteins via neural iterative selection-expansion

Corresponding Author: Professor Nicholas Polizzi

Any redactions in this file are there to maintain patient confidentiality, the confidentiality of unpublished data, or to remove third-party material.

**This file contains all reviewer reports in order by version, followed by all author rebuttals in order by version.**

**Attachments originally included by the reviewers as part of their assessment can be found at the end of this file.**

Version 0:

Reviewer comments:

Referee #2

(Remarks to the Author)
Summary:

In their article “Zero-shot design of drug-binding proteins via neural selection-expansion”, the authors Benjamin Fry, Kaia Slaw, and Nicholas F. Polizzi present a convincing extension to the design of proteins for small molecule binding, which is accompanied by a partial abandonment of confidence metrics such as ipTM, ipAE or similar (apart from ligand pLDDT) that are firmly anchored in the literature. Leveraging the self-consistency concept (named in line 52), their method repeatedly switches between a bespoke sequence-predicting MPNN and a standard structure-predicting NN from the literature, particularly RosettaFold-AllAtom. The filter parameters used are mainly ligand heavy atom RMSD, pLDDT and Calpha RMSD of the protein ligand.

The unique characteristic and surplus merit of the presented work is mainly the in-house “LASErMPNN” (ligand-aware sequence engineering MPNN), which, as the authors describe in lines 106-115, has three major advantages over the well-known and most frequently used competitor, LigandMPNN: 1) distinct, pretrainable ligand encoder; 2) simultaneous decoding of sidechain dihedral angles; and 3) inclusion of ligand nodes in each round of encoding and decoding.

The authors then use three examples, 1) biotin-streptavidin sequence recovery; 2) sequence prediction and ranking of de novo drug-binding protein PiB; and 3) topoisomerase I inhibitor, to test the mode of action of their iterative composite flow.

Key results:

Significant improvement of 1st to 2nd round of designs. In the first round the binder design with the highest affinity with a Kd ~120 nM was improved to ~¨1.2 nM by a double mutation.

A reduction to self-consistency measured in RMSD and ligand pLDDT could address significant design tasks, which, therefore, challenges the current focus on the optimisation and filtering based on computed scores such e.g., ipTM, ipAE etc.

Originality and significance:

In the discussion section, in Fig. 1c and E20, the authors compare their consecutive use of the sequence-generating LASErMPNN and the structure-generating RFAA within the sequence expansion algorithm NISE, towards Rosetta-energy based iterative optimisation. While this is a comparison previously reported in Dauparas et al. (2025) when LigandMPNN was introduced, the relevant benchmark is the comparison of LigandMPNN and LASErMPNN directly and within the NISE algorithm. Further comparisons could include but are certainly not limited to Zhang et al. (2024).

In lines 453 to 455 the authors argue LigandMPNN could have been used with similar success within the NISE framework.

Since the novelty of the publication particularly arises from the introduction of the newly trained LASErMPNN, the authors currently fail to substantially differentiate themselves from prior art.

Data & methodology:

To enhance transparency and reproducibility, the authors should provide a detailed list of designed sequences, specifically including at least the four experimentally tested NISE-derived sequences and the 18 COMB-generated control designs. Additionally, for the NISE-derived sequences, it would be highly valuable to include the progression of sequence iterations, clearly displaying how individual amino acid positions evolved during the iterative design process. Alongside these sequences, the authors should report critical model decision parameters such as sequence prediction probabilities, ligand pLDDT scores, ligand heavy-atom RMSD, and protein Cα-RMSD at each iteration. Including this information would allow readers to gain deeper insights into the model's internal decision-making process, better understand how iterative refinement improved binding properties, and facilitate comparisons with future computational design studies.

Appropriate use of statistics and treatment of uncertainties (if applicable):

The manuscript does not contain any statistical analysis, nor was that warranted. The evaluation parameters of the NISE algorithm are displayed in absolute values providing scatterplots e.g. Fig 2b and d as well as mean values.

Suggested improvements:

The novelty of training a sequence-generating MPNN even with substantial points of differentiation from current models such as LigandMPNN is limited. Especially, since LigandMPNN performs equally well in the sequence recovery as shown in Figs. S3-5. The authors themselves argue in lines 451-453 LASErMPNN could be replaced with LigandMPNN within the NISE framework without a loss in precision or success rate. A shift of focus towards the application case would improve the manuscript, since the achievement of providing a high affinity protein ligand for non-protein small molecule hydrolysis protection with the displayed level of structural as well as functional detail is impressive. To shift the manuscript's emphasis more clearly towards the impressive experimental outcomes rather than primarily focusing on the introduction of LASErMPNN, several additional targeted experiments could be highly informative. For instance, it would be valuable to perform competitive binding assays using EPIC with structurally-related camptothecin derivatives, such as irinotecan, topotecan or camptothecin itself. Such experiments would directly demonstrate EPIC's selectivity for exatecan, highlighting the designed protein's ability to discriminate subtle yet therapeutically relevant structural differences. Furthermore, a functional enzymatic assay examining human topoisomerase I inhibition by exatecan alone and in complex with EPIC would clarify whether EPIC binding preserves or enhances the biological efficacy of exatecan, bridging the gap between biophysical affinity data and direct biological relevance. Cell-based cytotoxicity assays, for instance employing colorectal or ovarian carcinoma cell lines sensitive to camptothecin derivatives (e.g., HCT116 or OVCAR-3), could similarly unravel the practical therapeutic potential of EPIC–exatecan complexes. Finally, conducting detailed structural overlays between the EPIC–exatecan crystal structure and previously reported exatecan–topoisomerase I complexes (e.g., PDB: 1T8I) would explicitly illustrate the structural novelty or mimicry inherent in EPIC's mode of binding, adding significantly to the mechanistic and biological narrative of the manuscript. It should clearly be stated that not all of these are necessary, but some could help further substantiate the relevance to the general audience.

It would also be informative to test NISE on structurally similar but non-binding "decoy" ligands, to quantify and highlight the specificity of the self-consistency selection method clearly. Further, the authors should conduct ablation studies of self-consistency filters and systematically vary the thresholds or remove individual filters (such as ligand RMSD, Cα-RMSD, ligand pLDDT) to identify clearly how each impacts the final selection and success rates.

Detailed line-by-line feedback:

Lines 131-132: While the higher sequence recovery rate (53% to 38%) with biotin ligands present is encouraging, there is no classification of the already known sequence-generating MPNNs, such as the previously mentioned LigandMPNN. The authors should provide details for the sequence recovery rate for these alternative inverse folding models with and without ligand context. Furthermore, the authors should describe the physical chemical nature of the diverging residues. If the streptavidin-biotin sequence recovery test is a standard assessment in literature, the authors should include respective references.

Lines 175-176: The authors describe the process of scaffold generation prior to their main work. It would be interesting to know why particularly alpha helices were used as scaffold libraries. They are quite common in literature due to the inherent bias of RFdiffusion-based de novo shape generation. However, since the library was curated by the authors, it remains unclear why alpha helices were preferred.

Lines 183-185: For a skilled person unfamiliar with physics-based models it remains difficult to understand how the exatecan conformations were 'built' from the crystal structure of another molecule and refined using the mechanistic force fields. The authors should describe the process in more detail in the main text and include a structural comparison between the two molecules.

Lines 334-336: The relaxed structure obtained using Amber molecular mechanics could have been employed as the initial structure for the NISE algorithm. Why was the relaxation step only introduced during the binder maturation?

Lines 340-343: It remains unclear how the authors arrived at the mutations. The author should explain their choice in regard to the model sequence re-prediction.

Lines 358-360: The authors state that the binding position of exatecan and the surrounding pocket of their highest affinity binder EPIC deviates from the obtained crystal structure and attribute this to the structure prediction of RFAA. However, the statement is not supported by any argument. The authors should explain their hypothesis in more detail and provide experimental results to underpin it.

Lines 377-389: While being a very valuable analysis between the current state-of-the-art structure prediction models the entire paragraph does not refer or substantiate the novelty of the NISE algorithm or training of the LASErMPNN.

Lines 451-453: Irrelevant sentence with little to no additional value since the argument for the introduction of LASErMPNN is to design and optimise sequences.

References.

Dauparas, J., Lee, G.R., Pecoraro, R. et al. Atomic context-conditioned protein sequence design using LigandMPNN. Nat. Meth. 22:717–723 (2025). https://doi.org/10.1038/s41592-025-02626-1

Zhang, Z., Shen, W.X., Liu, Q. et al. Efficient generation of protein pockets with PocketGen. Nat. Mach. Intell. 6:1382–1395 (2024). https://doi.org/10.1038/s42256-024-00920-9

(Remarks on code availability)
Code review

The NISE repository provides the core implementation of the iterative selection–expansion pipeline, but its current setup requires users to manage two separate conda environments (one for LASErMPNN and another for Boltz-1x/2x) as well as manually install ProDy from source. It would be valuable to consolidate dependencies into a single environment.yml (or Dockerfile) that can be invoked with one command. Additionally, it appears that the current workflow assumes pre-docked protein–ligand PDB files, but the initial docking process is neither implemented nor clearly documented. Including guidance or references (such as COMBS or Boltzdesign1) would make the pipeline more accessible. Finally, expanding the README with a fully worked end-to-end example, complete with directory layout, sample commands, and expected output, would guide new users far more effectively than the current high-level instructions.

The LASErMPNN repository is well-organized and straightforward to install and run. To further enhance accessibility and reproducibility, it would be helpful to include example PDBs along with command-line examples demonstrating different use-cases (ideally reflecting the ones described in the paper). A Google Colab notebook that goes through setup and inference would make this tool even more accessible. As also noted in the repository as a to do item, publishing the exact train, validation, and test split files along with a script to download and organize the corresponding PDB structures would be a valuable improvement. Finally, proper versioning and distributing the model weights through GitHub releases would help reproduce results more reliably.

Version 1:

Reviewer comments:

Referee #2

(Remarks to the Author)
The authors (Benjamin Fry, Kaia Slaw, and Nicholas F. Polizzi) have thoroughly addressed the concerns we raised previously. In particular, they substantially expanded the comparison between LASErMPNN and LigandMPNN (Ext. Data Figs. 1–2 and Suppl. Figs. 38–39). Beyond reporting overall performance, they now analyze concrete physical determinants of design quality, including side-chain packing density around the ligand and the associated van der Waals attractive/repulsive terms. This additional breakdown is informative and suggests that LASErMPNN can yield lower-density, energetically favorable packing in certain contexts. At the same time, the authors emphasize that the two approaches may be advantageous in different settings rather than one method uniformly outperforming the other.

A second major strength that is now clearer is the flexibility of the NISE pipeline. The revised manuscript demonstrates that key components can be swapped without disrupting the overall workflow, particularly the substitution of RosettaFold-All Atom (RFAA) with Boltz-1 and Boltz-2, which improves the pass rate for designs meeting the self-consistency criteria (see lines 464–470). In addition, the authors broaden the set of selection metrics used after sequence expansion, including Boltz-2's binding-related score (reported as P(bind) / affinity_probability_binary, per the Boltz documentation). Taken together, this revision better motivates the authors' claim that NISE is modular and can readily integrate improved structure prediction models and new objective functions as they emerge which is an important practical advantage for the community.

The revision is further strengthened by the additional experimental validation on a completely new target, apixaban. The authors repeat the full process on this independent system and report very high success rates (5/6 designs) and exceptionally strong affinities, including favorable comparison to previously reported efforts against the same target. This added dataset supports the manuscript's broader message that NISE can generalize efficiently with relatively few designs.

However, several points would still benefit from further clarification. In particular, it would be valuable (primarily for community interpretability) to clarify the usability of P(bind) (Ext. Data Figs. 6–8). The authors already note that P(bind) should primarily be interpreted as a binder vs non-binder signal rather than an affinity-ranking metric, and they further show that it is only weakly correlated with ligand pLDDT suggesting it may provide partially orthogonal information (Ext. Data Fig. 8). At the same time, Ext. Data Fig. 7 indicates an unexpected relationship between P(bind) and affinity (including an apparent negative trend in parts of the EPIC series), which makes its practical interpretation less straightforward. Since P(bind) is used as a key selection/optimization criterion in the apixaban design campaign, it would therefore be very useful for readers to also see the corresponding P(bind)–affinity relationship (as well as ligand pLLDT which showed strong correlation for the EPIC series) for the apixaban dataset. This would help clarify whether P(bind) provides actionable signal in this setting and, more generally, how it should be interpreted in practice (e.g., strictly as a classifier and with what threshold, or whether it adds limited benefit beyond existing confidence and self-consistency filters). In addition, while Suppl. Table 2 now shows the progression of the EPIC "lineage", it would be helpful for the reader to highlight the exact residues that got changed in each round. The same analysis would be useful also for their new APEX binder.

From a practical perspective, the Github repository is well structured and has meaningful, comprehensible comments that guide the user for both (re-)training of LASErMPNN and the execution of the NISE pipeline using the example input. Furthermore, the authors substantiated and expanded the notes on and protocols of the initial target preparation by providing a detailed secondary Github repository for the rigid-rigid backbone docking using CARPdock, which includes the apixaban as exemplary case study. On that note, when we tested both Google Colab implementations of (1) CARPdock and (2) 1-round NISE, we found them to run smoothly and the opportunity to inspect the initial input as well as the output structures/sequence is helpful for the user.

The installation of the separate LASErMPNN installation using cuda 11 was clear and was simple to integrate with an existing Boltz-1x installation. For the combined installation within the recommended virtual environment, we found that prody and rdkit were not called in the setup bash script but had to be installed separately. These two packages should be listed in the initial setup.sh as well.

A more pressing point is the unclear guidance for running NISE. If the user is not following the example but sets their own input structure the recommendation in the readme file is to manually create folders and edit paths in the underlying python scripts. This is a rather unusual approach for code shared in a public repository. A more reasonable and user-friendly alternative would be to use arguments that parse this custom information. These arguments should be embedded in executional bash scripts with examples. We see this as a necessary improvement before the NISE framework can be presented to the broader community. The GitHub repository of deepmind/alphafold3/input.md and the RFdiffusion3 Documentation - foundry 0.1.7 documentation are great examples of user-friendly and easy-to-edit campaign customisation guidelines.

Overall, we believe the revised manuscript will be a valuable contribution for the community, particularly because it presents a flexible and modular pipeline that achieves strikingly high experimental success rates. The manuscript is also strengthened by the very rigorous and comprehensive analyses, with extensive supporting data that substantiate the main conclusions throughout. The authors appropriately frame the primary novelty as arising from the NISE framework rather than from a single component model. In this context, it is important to be explicit that while LASErMPNN shows clear advantages in specific analyses, it does not appear to represent a dramatic across-the-board improvement over LigandMPNN. The key remaining question is therefore whether the conceptual advance of the NISE framework as an iterative, modular integration of largely established design and structure-prediction tools constitutes sufficient novelty on its own. In our view, this case can be made due to the direct practical impact it can provide; the ability to swap components, incorporate new metrics, and achieve high experimental hit rates with relatively few designs is very useful and can easily integrate within existing design frameworks.

(Remarks on code availability)
See main comments above.

Referee #3

(Remarks to the Author)
To design new ligand binding proteins, Fry, Slaw, and Polizzi demonstrate a combination of two neural networks in an iterative design algorithm (NISE) for joint optimization of ligand-protein pose and protein sequence. They develop a new ligand-specific MPNN (LASErMPNN) for this purpose; they show zero shot design of nM and pM binders for two different small molecule drugs. The hit rates for the process are high, affinities and specificities excellent and consistent with the binding modes, and the biochemistry, structural biology, and biophysical characterization are exquisite. This paper has been a preprint for a while; this current version is fairly polished. As such, I have very minor experimental details, statistical methods, and a code availability statement to note. In terms of the overall impact, the strength of this paper is the use of joint optimization of sequence and structure in protein design. While the field moves too fast for me to state for certain, at the time of of the preprint this appeared to be a novel contribution. The main limitation of this method is that a realistic ligand-protein backbone pose must be given as input; this limits generalizability to non-specialists interested in their task-specific problems (though it appears quite useful to specialists!). Additionally, one could argue that this method is more of an 'affinity maturation' method rather than a true zero-shot design given that other algorithms p(sequence|structure) give binding hits with these realistic poses (albeit at weaker affinities). I think these are minor quibbles - overall, the paper presents a new type of protein design algorithm for which utility is demonstrated with strong experimental data.

Minor things:

1. Fluorescence anisotropy data. The data shown in Fig. 3f,g and panels do a disservice to the global fitting done for the anisotropy data as reported in the SI. Specifically, the traces shown are truncated before they are saturated (e.g. EPIC binder panel 3f), and error bars and statistical methods are not reported.

(Remarks on code availability)
Data availability statement (with trainee; "I" refers to the trainee):

I reviewed the LASErMPNN repository (https://github.com/polizzilab/LASErMPNN) and examined the NISE repository (https://github.com/polizzilab/NISE). I was able to install and run LASErMPNN for ligand-conditioned sequence design on a protein-ligand system outside the training set, confirming that the inference pipeline functions as described. The Google Colab notebooks for both tools are well-constructed and substantially lower the barrier to entry. The authors have been responsive to prior reviewer feedback -- the addition of setup.sh for NISE, Colab notebooks, CARPdock for initial pose generation, and training data splits on Zenodo are welcome improvements.

A few remaining suggestions:

Dependency management: In my experience installing LASErMPNN, the conda environment files do not pin package versions, which can cause installation difficulties given the tight coupling between PyTorch, CUDA, and torch-scatter/PyKeOps. The NISE setup.sh handles this better by pinning key versions. Aligning both repos on pinned dependencies (or providing a Docker/Singularity container) would improve long-term reproducibility.

Bundled example inputs: The NISE repository now includes example PDBs and sample commands in the README, which is helpful. However, LASErMPNN still does not bundle any example PDB files -- users must supply their own. Including even a single small example with expected output would allow users to verify their installation immediately.

Testing and validation: Adding a small test suite -- even a smoke test that loads the model, runs inference on a bundled example PDB, and checks output format -- would help users verify their installation and protect against regressions as the code evolves.

Overall, both repositories are functional and represent a meaningful contribution to the protein design toolkit. The suggestions above are intended to help maximize their impact and longevity as community resources.

Referee #5

(Remarks to the Author)
I co-reviewed this manuscript with one of the reviewers who provided the listed reports.

(Remarks on code availability)
I reviewed the LASErMPNN repository (https://github.com/polizzilab/LASErMPNN) and examined the NISE repository (https://github.com/polizzilab/NISE). I was able to install and run LASErMPNN for ligand-conditioned sequence design on a protein-ligand system outside the training set, confirming that the inference pipeline functions as described. The Google Colab notebooks for both tools are well-constructed and substantially lower the barrier to entry. The authors have been responsive to prior reviewer feedback -- the addition of setup.sh for NISE, Colab notebooks, CARPdock for initial pose generation, and training data splits on Zenodo are welcome improvements.

A few remaining suggestions:

Dependency management. In my experience installing LASErMPNN, the conda environment files do not pin package versions, which can cause installation difficulties given the tight coupling between PyTorch, CUDA, and torch-scatter/PyKeOps. The NISE setup.sh handles this better by pinning key versions. Aligning both repos on pinned dependencies (or providing a Docker/Singularity container) would improve long-term reproducibility.

Bundled example inputs. The NISE repository now includes example PDBs and sample commands in the README, which is helpful. However, LASErMPNN still does not bundle any example PDB files -- users must supply their own. Including even a single small example with expected output would allow users to verify their installation immediately.

Testing and validation. Adding a small test suite -- even a smoke test that loads the model, runs inference on a bundled example PDB, and checks output format -- would help users verify their installation and protect against regressions as the code evolves.

Overall, both repositories are functional and represent a meaningful contribution to the protein design toolkit. The suggestions above are intended to help maximize their impact and longevity as community resources.

**Open Access** This Peer Review File is licensed under a Creative Commons Attribution 4.0 International License, which permits use, sharing, adaptation, distribution and reproduction in any medium or format, as long as you give appropriate credit to the original author(s) and the source, provide a link to the Creative Commons license, and indicate if changes were made.
In cases where reviewers are anonymous, credit should be given to 'Anonymous Referee' and the source.
The images or other third party material in this Peer Review File are included in the article's Creative Commons license, unless indicated otherwise in a credit line to the material. If material is not included in the article's Creative Commons license and your intended use is not permitted by statutory regulation or exceeds the permitted use, you will need to obtain permission directly from the copyright holder.
To view a copy of this license, visit https://creativecommons.org/licenses/by/4.0/

We thank the reviewer for their enthusiasm for our work and the helpful comments and suggestions, which improved the manuscript (ms). In response to the referee, we added 1 new main figure, 22 new supplementary / extended data figures, and 5 new supplementary tables. We also added new main and supplementary text, which we mark in blue font face in the files appended with extension "_blue_markup.pdf". Most notably, we added new experiments and computational analyses that support the conclusion that the NISE algorithm is a novel and powerful way to rapidly produce high affinity small-molecule binding proteins, evidenced by the design of entirely new small-molecule binding proteins for a new target. Our responses to the reviewer in this document are written in blue font face.

Summary:

In their article "Zero-shot design of drug-binding proteins via neural selection-expansion", the authors Benjamin Fry, Kaia Slaw, and Nicholas F. Polizzi present a convincing extension to the design of proteins for small molecule binding, which is accompanied by a partial abandonment of confidence metrics such as ipTM, ipAE or similar (apart from ligand pLDDT) that are firmly anchored in the literature. Leveraging the self-consistency concept (named in line 52), their method repeatedly switches between a bespoke sequence-predicting MPNN and a standard structure-predicting NN from the literature, particularly RosettaFold-AllAtom. The filter parameters used are mainly ligand heavy atom RMSD, pLDDT and Calpha RMSD of the protein ligand.

The unique characteristic and surplus merit of the presented work is mainly the in-house "LASErMPNN" (ligand-aware sequence engineering MPNN), which, as the authors describe in lines 106-115, has three major advantages over the well-known and most frequently used competitor, LigandMPNN: 1) distinct, pretrainable ligand encoder; 2) simultaneous decoding of sidechain dihedral angles; and 3) inclusion of ligand nodes in each round of encoding and decoding.

During our revisions, we noticed a 4th advantage to using LASErMPNN and have updated the ms accordingly. Briefly, LigandMPNN tends to overpack designs, resulting in a marked increase in vdW repulsion energy relative to LASErMPNN designs.

Lines 136 - 140:

> "In a head-to-head comparison between LASErMPNN and LigandMPNN, we observed a general tendency for LigandMPNN to produce designs that were more overpacked, as measured by an increased density of protein heavy atoms within 5 Å of the ligand, as well as a higher vdW repulsion energy of both the ligand and overall structure (Extended Data Fig. 1, Kolmogorov-Smirnov test p-value < 1e-16)."

The authors then use three examples, 1) biotin-streptavidin sequence recovery; 2) sequence prediction and ranking of de novo drug-binding protein PiB; and 3) topoisomerase I inhibitor, to test the mode of action of their iterative composite flow.

Key results:

Significant improvement of 1st to 2nd round of designs. In the first round the binder design with the highest affinity with a Kd ~120 nM was improved to ~¨1.2 nM by a double mutation.

A reduction to self-consistency measured in RMSD and ligand pLDDT could address significant design tasks, which, therefore, challenges the current focus on the optimisation and filtering based on computed scores such e.g., ipTM, ipAE etc.

We thank the reviewer for recognizing the potential impact of our work. We focus in this paper on ligand heavy-atom RMSD and protein C-alpha (Ca) RMSD as metrics of self-consistency; and we use ligand pLDDT as a metric of model confidence. We note in a new Extended Data Figure (**Extended Data Fig. 8**, reproduced below) that ligand pLDDT is correlated with ipAE and ipTM. We also now show that a new parameter from Boltz-2, the probability of binding, or P(bind), is uncorrelated with ligand pLDDT, and can be jointly optimized alongside ligand pLDDT via the NISE design algorithm.

In new results, we used both ligand pLDDT and P(bind) during the NISE design trajectory of new apixaban-binding proteins (see below), which – after filtering by protein Ca and ligand RMSD – resulted in very high affinity binders to a new target, apixaban. Ultimately, we also filter designs on a biophysical parameter: buried "unsatisfied" polar atoms of the ligand and amino-acid sidechains. With this final filtering step, we minimize the number of polar atoms that are not hydrogen bonded (H-bonded). We clarify our ranking and filtering protocol in the paper (e.g., see section Correlation of Ligand pLDDT with other Confidence Metrics from Boltz-2 in supplement and lines 628-641 in Discussion).

.

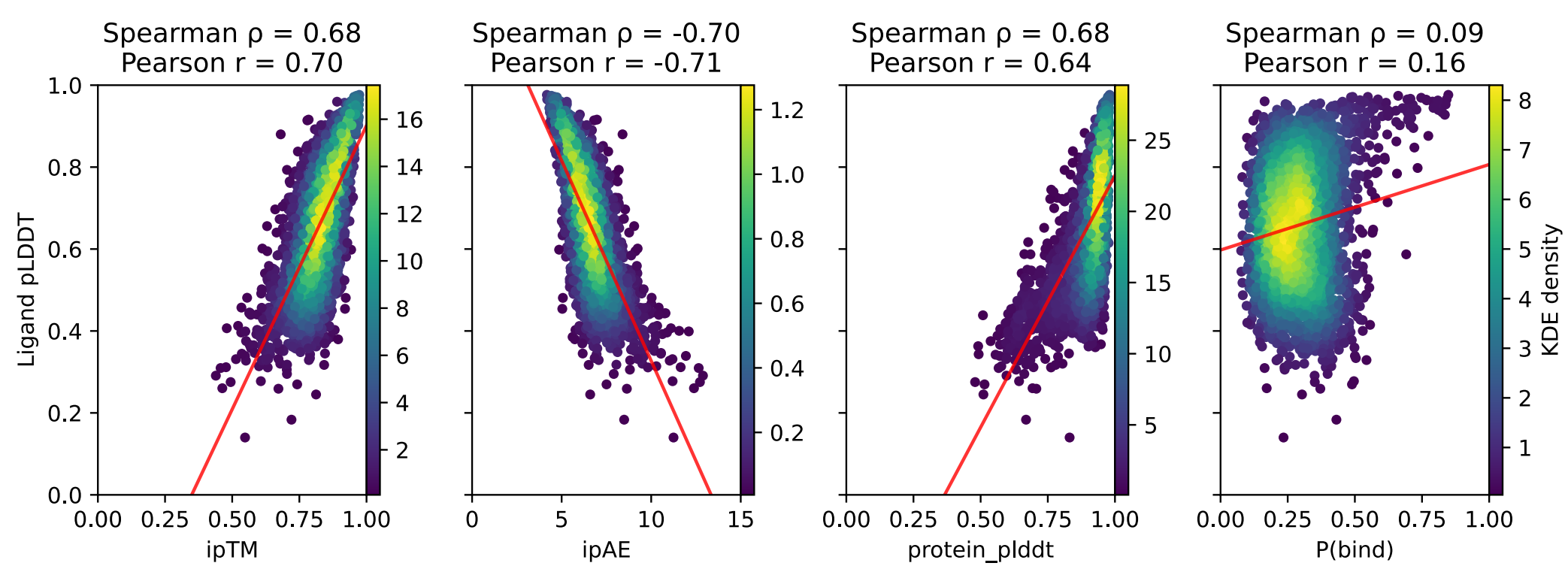


**Extended Data Fig. 8. Ligand pLDDT is correlated with other confidence metrics but not Boltz-2's P(bind).**

Originality and significance:

In the discussion section, in Fig. 1c and E20, the authors compare their consecutive use of the sequence-generating LASErMPNN and the structure-generating RFAA within the sequence expansion algorithm NISE, towards Rosetta-energy based iterative optimisation. While this is a comparison previously reported in Dauparas et al. (2025) when LigandMPNN was introduced, the relevant benchmark is the comparison of LigandMPNN and LASErMPNN directly and within the NISE algorithm. Further comparisons could include but are certainly not limited to Zhang et al. (2024).

We would like to emphasize that our analysis in Fig 1c is original, and no part of it was reported in the LigandMPNN paper. This is because the analysis in Fig. 1c tests the importance of the structure-refinement module within our iterative selection–expansion algorithm. We apologize for any confusion in our description in the initial submission. In the LigandMPNN paper, the authors compared LigandMPNN's native sequence recovery and Rosetta's native sequence recovery on a set of fixed protein backbones. Instead, we are testing if using Rosetta's energy function to optimize the structures themselves is equivalent to using a neural network to do so (via predicting co-structures). The results show that using a neural network for co-structure prediction is critical, as Rosetta's energy function failed to refine backbones enough for LASErMPNN to design more likely sequences for them. We added new text in the Discussion to make this clearer:

Lines 547 - 555:

> "We investigated the computational underpinnings of why NISE was so successful where other methods fell short. We hypothesized that, in addition to more extensive sampling focused on a select few starting points, using reciprocal neural networks trained on the PDB could push the designs into more realistic binding solutions. To test this idea, we performed a computational simulation and found that using Rosetta's mixed physics- and bioinformatics-based energy function failed to make protein structures more compatible with designed sequences (assessed via NLL and ligand pLDDT), while using reciprocal neural networks did (Fig. 1c). We interpret these results to mean that current empirical energy functions cannot fully capture the nuances of productive protein–ligand interactions."

Our analysis provides interpretation for why LigandMPNN and Rosetta failed to find good binders for an identical target (apixaban) in a previous paper by Baker's group[1], while using NISE with LASErMPNN and RFAA was quite successful (see below). It is important to note that Baker's group did not use an iterative selection–expansion protocol with LigandMPNN and Rosetta (selection–expansion is novel to our paper in this context), but our analysis suggests that, even if they did, Rosetta would not have been able to effectively optimize the coordinates of designs.

We have now added additional analysis to directly compare the performance of LASErMPNN and LigandMPNN within the NISE algorithm. We ran the NISE algorithm for the same input co-structure using both LASErMPNN and LigandMPNN as the sequence design model, and using the co-structure predictor Boltz-2. We added two new supplementary figures that illustrate the results (**Supp. Figs. 38** and **39**, reproduced below). The analysis of the two trajectories for the design of an apixaban-binding protein shows that both models for sequence design produce comparable aggregate confidence metrics (such as ligand pLDDT), as we hypothesized could be the case in the paper [because the models were trained on the same underlying data (the PDB) and perform similarly (with respect to sequence recovery) on a test set of proteins]. Our analysis also showed something quite interesting that we didn't fully anticipate. While metrics such as ligand pLDDT are similarly optimized between the two models, we found that LigandMPNN tends to produce overpacked designs compared with LASErMPNN (see response above). We added new figures and discussion around this phenomenon in the paper (e.g., Extended Data Fig. 1 and Supp. Figs. 40 and 41.) We reproduce **Supp. Fig. 40** below. Furthermore, LASErMPNN

and LigandMPNN do not perform equivalently on every task (e.g., see the streptavidin–biotin analysis below, where LASErMPNN outperforms LigandMPNN), so we expect the models to be complementary on a case-by-case basis. We have updated the NISE GitHub repository with an additional script that uses LigandMPNN for sequence generation in place of LASErMPNN within the NISE protocol, making it straightforward for users to compare the two and leverage whichever model is better suited to a particular design problem.

In summary, we show that LASErMPNN is superior or comparable to LigandMPNN in a number of ways. In addition to LASErMPNN, the major novelty of our work, we think, is the introduction of the iterative selection–expansion algorithm that only uses neural networks to design sequence and optimize the co-structure in an iterative fashion. Joining the two networks in series and selecting the most promising (confident) models allows NISE to jointly optimize sequence, structure, and ligand conformer in a way that no other previous algorithm has shown via high experimental success rates.

Lines 606-614:

> "However, a closer look at the designed binding-site residues revealed some differences between top designs from each method. Where the binding sites differed, LigandMPNN generally designed larger residues near the ligand (Supplementary Fig. 40). The general overpacking of structures by LigandMPNN (Extended Data Fig. 1 and Supplementary Fig. 41) might be attributed to its disjoint sampling of sequence and sidechain dihedrals: the entire sequence is designed first, followed by all sidechain dihedrals. By contrast, LASErMPNN jointly determines sequence and sidechain dihedrals within the same autoregressive decoding trajectory (Fig. 2a), perhaps affording a more nuanced awareness of sidechain packing that, in turn, influences sequence design."

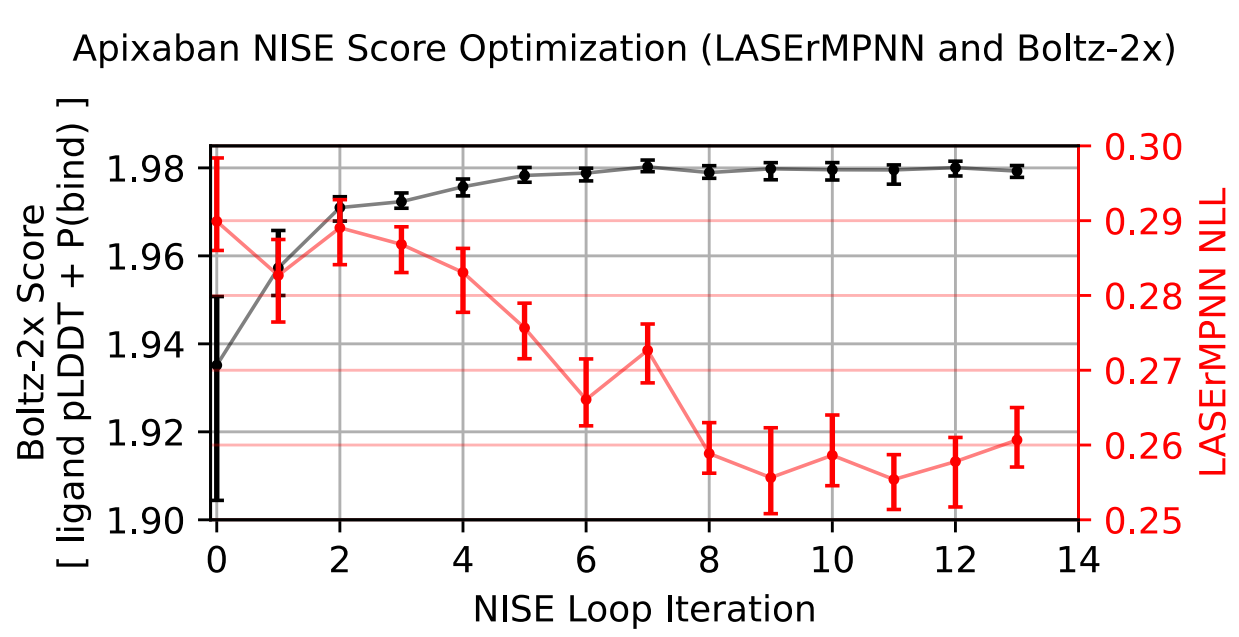


**Supplementary Fig. 38. NISE optimizes Boltz-2 Score and LASErMPNN NLL for apixaban–NTF2 designs.**

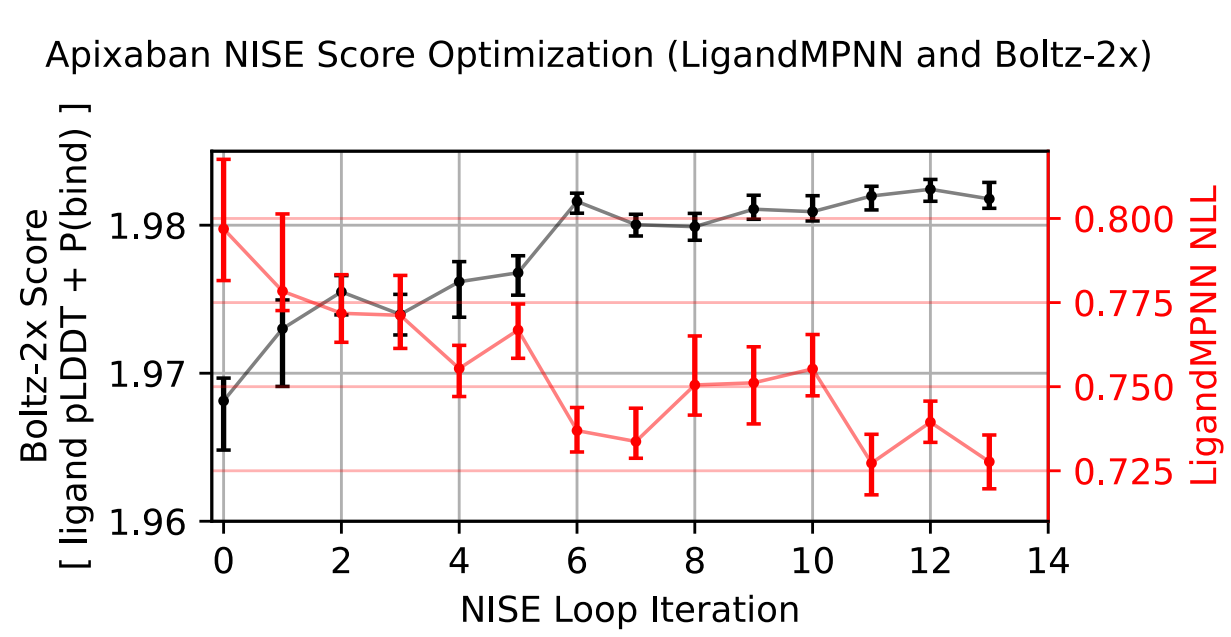


**Supplementary Fig. 39. NISE optimizes Boltz-2 Score and LigandMPNN NLL for apixaban–NTF2 designs.**

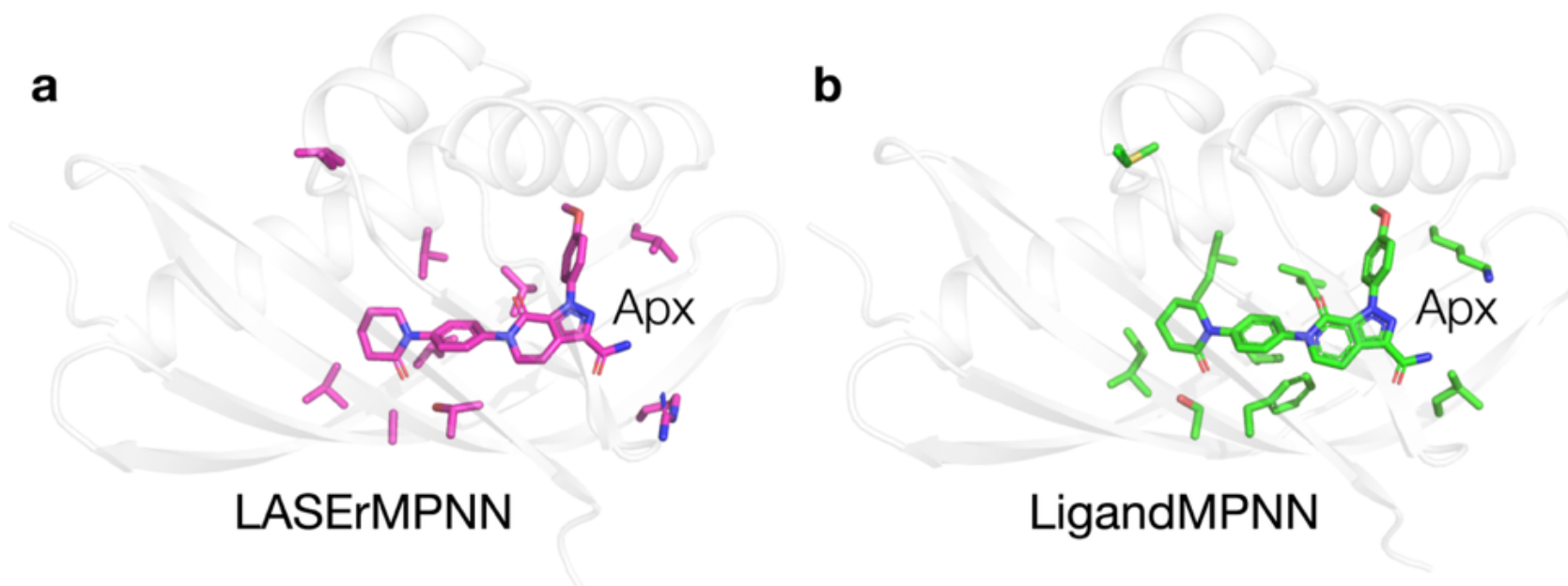


**Supplementary Fig. 40. LigandMPNN designs larger residues in the binding pockets of apixaban–NTF2 designs.**

In lines 453 to 455 the authors argue LigandMPNN could have been used with similar success within the NISE framework. Since the novelty of the publication particularly arises from the introduction of the newly trained LASErMPNN, the authors currently fail to substantially differentiate themselves from prior art.

We think LASErMPNN is preferable to LigandMPNN for a variety of reasons (see above), but the huge step change is the introduction of an algorithm that can iteratively computationally optimize designs for high affinity and high success rates. While Baker's group has used LigandMPNN (+ Rosetta) to design binders to some ligands with relatively weak affinity[1] (high nanomolar and micromolar dissociation constants), they needed to experimentally screen approximately 10,000 or more designs to find a binder, sometimes combining mutations from experimental deep-mutational scanning to improve affinity. These are costly and time consuming experiments that are not accessible to all groups, and the ultimate outcomes were only weak-affinity binders. Here, we show that we can encode tight binding completely in a computational algorithm, NISE. This is striking because, while design of mini-proteins that bind protein targets is becoming routine – with low nM binders achievable from a few designs for binding to various protein targets – the design of small-molecule binding proteins has remained very far from this. Indeed, the only experimental cases with high success rates was our previous work with COMBS (2 of 6 bound apixaban, $K_d$ = 600 nM[2]; 1 of 3 bound rucaparib, $K_d$ = 5 nM[3]). Here, we compare our new method, NISE, to COMBS and LigandMPNN+Rosetta, and NISE is unequivocally superior.

To demonstrate the power of NISE on a second small-molecule target, we have now added new experiments where we used the same mixed alpha/beta backbones as Baker's group for the design of an apixaban binder. Baker's group used LigandMPNN and Rosetta for design[1]; they docked the ligand into the NTF2 backbones using a method called RIFdock, then performed three rounds of LigandMPNN and Rosetta minimization (1 sequence each round). Their algorithm did not select the best designs each round as representatives for iteratively expanding the number of sequences. Only 1 design out of ~ 10,000 tested by Baker's group bound the drug, with a 700 nM dissociation constant ($K_d$). By contrast, we used LASErMPNN and Boltz-2 to design apixaban binders using our NISE algorithm. 5 of 6 tested designs bound the drug with low (< 50) nM $K_d$, the tightest being 80 pM $K_d$ – the tightest binder ever designed completely by computation. Thus, we differentiate ourselves from prior art, as NISE achieved a 83% success rate whereas LigandMPNN+Rosetta had only a 0.01% success rate. The full picture is not

entirely captured in the single parameter of success rate either: the designed binders from NISE had much higher affinity than the single binder from LigandMPNN+Rosetta (and also the two previously designed binders from COMBS). Each of the 5 binders from NISE bound at least an order of magnitude more tightly than the single binder from LigandMPNN+Rosetta, with the highest affinity NISE binder (which we call APEX) binding ~ 10,000x more tightly ($K_d$ = 80 pM or 0.08 nM, see **Fig. 6**, reproduced below). The key was coupling two neural networks to jointly solve this design task– the binding of small molecules involves creation of destabilizing cavities and subtle, precise interactions with a small molecule in a densely crowded pocket. Combining two neural networks helps to sample the appropriate degrees of freedom to iteratively optimize sequence and structure jointly for this task. It has not been accomplished previously. We have added a section in the results titled "Design of apixaban binders using NISE" that details our design process and results (lines 444-501).

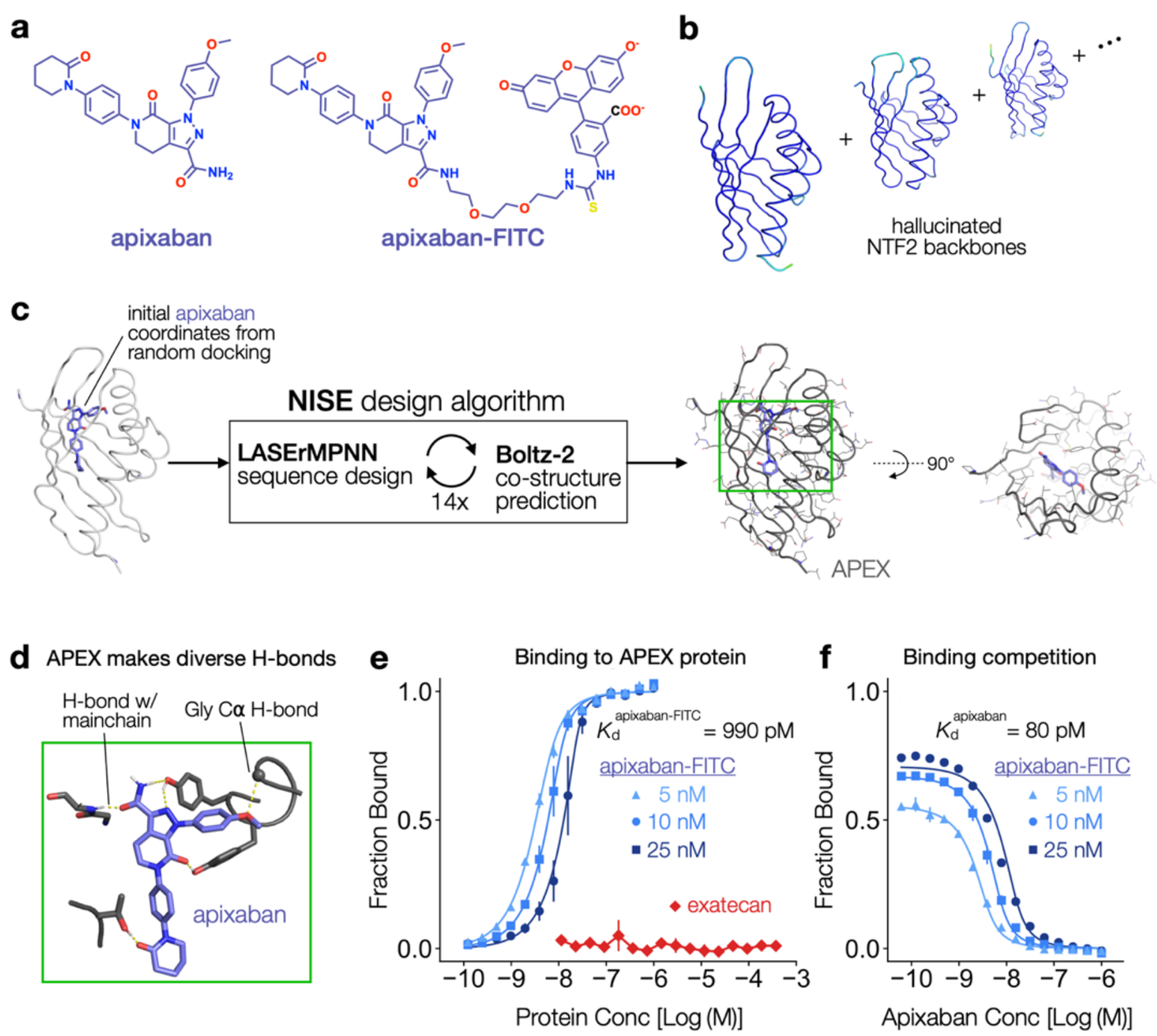


**Fig. 6. Design of high affinity apixaban-binding proteins using NISE and the NTF2 fold.**

Data & methodology:

To enhance transparency and reproducibility, the authors should provide a detailed list of designed sequences, specifically including at least the four experimentally tested NISE-derived sequences and the 18 COMB-generated control designs.

Designed protein sequences are listed in Supplementary Tables 1 and 2. We also provide the coordinate PDB files of the design models in Zenodo (https://doi.org/10.5281/zenodo.18308430). The following link should give the reviewer access

to the restricted upload (which we will release upon publication):
https://zenodo.org/records/18308430?token=eyJhbGciOiJIUzUxMiJ9.eyJpZCI6IjZhZTU0NTZkLTRmOGQtNGRkYy1iYjUyLTM3YTY5Y2MzZDE4MiIsImRhdGEiOnt9LCJyYW5kb20iOiI4YjM5OWM0NDRmOWUxMzc3YjYxNWI4NjQ2YjhiODA0MSJ9.JrgPfSpUGrJvufO0NfyGAhrUcgUuJgHZwo-jBkghkeDv7HF6DQn-AS-n_yPgYGcKOJ6bIYUXuCKnppWsQqcH4A

Additionally, for the NISE-derived sequences, it would be highly valuable to include the progression of sequence iterations, clearly displaying how individual amino acid positions evolved during the iterative design process. Alongside these sequences, the authors should report critical model decision parameters such as sequence prediction probabilities, ligand pLDDT scores, ligand heavy-atom RMSD, and protein Cα-RMSD at each iteration. Including this information would allow readers to gain deeper insights into the model's internal decision-making process, better understand how iterative refinement improved binding properties, and facilitate comparisons with future computational design studies.

We agree this could be a valuable analysis. We therefore found the "lineage" of designs that lead to EPIC within the NISE trajectory, starting from the original input co-structure. To track changes in model parameters across the lineage (including ligand pLDDT and  sequence identity from the previous "representative"), we made a new table showing each representative in the EPIC lineage (**Supp. Table 2**, reproduced below). EPIC itself came from round 22 of the NISE trajectory, and we note that not every iteration of NISE had a new representative (chosen only if the ligand pLDDT had increased relative to a current representative). The major sequence and structural changes tend to occur in the first few rounds of NISE, with more subtle sequence variation occurring in later rounds. It is important to stress that ligand pLDDT is not the only metric by which we filter designs before ordering DNA for experiments; so, even if ligand pLDDT plateaus during a NISE trajectory, new sequences can still improve other features, such as minimization of buried polar atoms that are not H-bonded.

**Supplementary Table 2. RFAA metrics for the EPIC lineage from the NISE trajectory**

| NISE Iter | Sequence | Protein pLDDT | Ligand (Exatecan) pLDDT | Protein RMSD (Å) | Ligand RMSD (Å) | Sequence Identity to Prev (Overall) | Sequence Identity to Prev (Buried) | Camptothecin pLDDT | Irinotecan pLDDT |
|---|---|---|---|---|---|---|---|---|---|
| **NISE Input** | DELLEALEKAFRELAKVGMTANKIFREMEEAARRGDEDRLTDGKKKHQDAAKKMEAILEKIWRILETIERWLRNIDEERAATMLELLTKLKRALEKYERIHETFKRIIEAARTAEEFLALLKILQELSKAADEAAKEIQYLFTQLLNT | 0.820 | 0.556 | - | - | - | - | 0.845 | 0.597 |
| **0** | TSLLQELKKLLEELKAQGEKAGKLVNRAKIAAAEGNEEELEECKEALKKAIAEEEAILEEIKKTLEEINNYLLKINSEEAENQIKLLDDLIAALEEFENIVKKAKALIEKSKNCEEFLKLEKEIDELTKKADEAAKKIIKLYNELLSE | 0.872 | 0.878 | 0.949 | 0.864 | 0.399 | 0.586 | 0.435 | 0.504 |
| **7** | NELLEELKNLLEELKEVGEKAKELVNAAKVAAENGDQEAFNACKEELKKAVKEMKSILKEIEEKLTSLREHLESIDSDKAAAQLKLLDELEKALAKFKAIVDKTVALIEESKDCETALKLLDDINELVKEADEAAAEIMELFTKLLDT | 0.878 | 0.870 | 0.843 | 0.751 | 0.520 | 0.741 | 0.422 | 0.697 |
| **10** | KKLLEQLDEEIEEVKEIGEKLEELLGELKQAAREGDEEKAQECLTKIREGLKEMIEKIKKIDSIIKQIQEKLKKDKSKEAKQKLVILGELKDALKRYKAIEARAKEIIDASKDVDQAKEAIGEIEELVAAAEREADEILQLWKELKET | 0.901 | 0.877 | 0.539 | 0.657 | 0.365 | 0.483 | 0.754 | 0.807 |
| **11** | AKKMAKVDRIIAELDKIGKELETALEKLEKAAQKKDEEAAKKALKELQGLIAKMNVLLDELDEEIQEILEEEKKKNTPKAQQKIKVLEELQAAIGQLKELVQKGQSILAETKDIKELLEAIEKIKGLVVQANAAAAEVQAKFAELMAI | 0.918 | 0.892 | 0.609 | 0.550 | 0.311 | 0.466 | 0.759 | 0.596 |
| **12** | VDKEKQIDEIIAELTEIGAELEKALEELKEAVENRDIAEAEKALKKLLELIEEQLVLLEELNETVESLKKEVEKEETEKAKEKVEILDKLLESIEKLQAIVEEGKKILEEVKDLDELAEAIGEVEELVVKADAEAKKVVDEYKKLKAI | 0.943 | 0.900 | 0.692 | 0.429 | 0.432 | 0.707 | 0.697 | 0.731 |
| **22 (EPIC)** | EDLIKKIKEIIKKLKELGAELEKALDRLKKAAEAKDIEAAEEALAELLKLQEEMLVLLEELKATVEKLLEEVRKIDTPAATKKVEVLEKLLDAINKMIDIVKEGLKILKEVKDLEELEEAIKEVEVLVKEADKYAAEIEEEYAKYLAI | 0.911 | 0.896 | 0.440 | 0.726 | 0.581 | 0.776 | 0.777 | 0.716 |

Appropriate use of statistics and treatment of uncertainties (if applicable):

The manuscript does not contain any statistical analysis, nor was that warranted. The evaluation parameters of the NISE algorithm are displayed in absolute values providing scatterplots e.g. Fig 2b and d as well as mean values.

Fig. 2b,d do not show data of the NISE algorithm per se. Instead, these panels show (i) the performance of LASErMPNN (sequence recovery) on a test set of proteins from the PDB and (ii) the filtering criteria of self-consistency and model confidence can rank a known de novo binder sequence (previously designed for the drug rucaparib) as a top sequence. These two panels are motivation to use both LASErMPNN and our filtering criteria within the NISE algorithm, but do not show performance of the NISE algorithm itself. For this, we point to, e.g., Fig. 1c, Fig. 3d,e, and Supp. Fig. 26.

Suggested improvements:

The novelty of training a sequence-generating MPNN even with substantial points of differentiation from current models such as LigandMPNN is limited. Especially, since LigandMPNN performs equally well in the sequence recovery as shown in Figs. S3-5. The authors themselves argue in lines 451-453 LASErMPNN could be replaced with LigandMPNN within the NISE framework without a loss in precision or success rate.

As we stated above, we think the introduction of the NISE algorithm for small-molecule binding proteins is a major step forward for the field, leading to dramatically improved success rates and affinities compared to current methods. LASErMPNN will be advantageous to LigandMPNN in certain cases, especially for design tasks that target small molecules with rare functional groups, since LASErMPNN benefits from a pretrained ligand encoder that is trained on copious synthetic data (e.g., quantum mechanical properties of millions of ligands, many not found in the PDB), whereas LigandMPNN is only trained on the (limited) data in the PDB. LigandMPNN also seems to suffer from a propensity to overpack designs leading to higher vdW repulsion energy than LASErMPNN designs (see above and **Extended Data Fig. 1**, reproduced below).

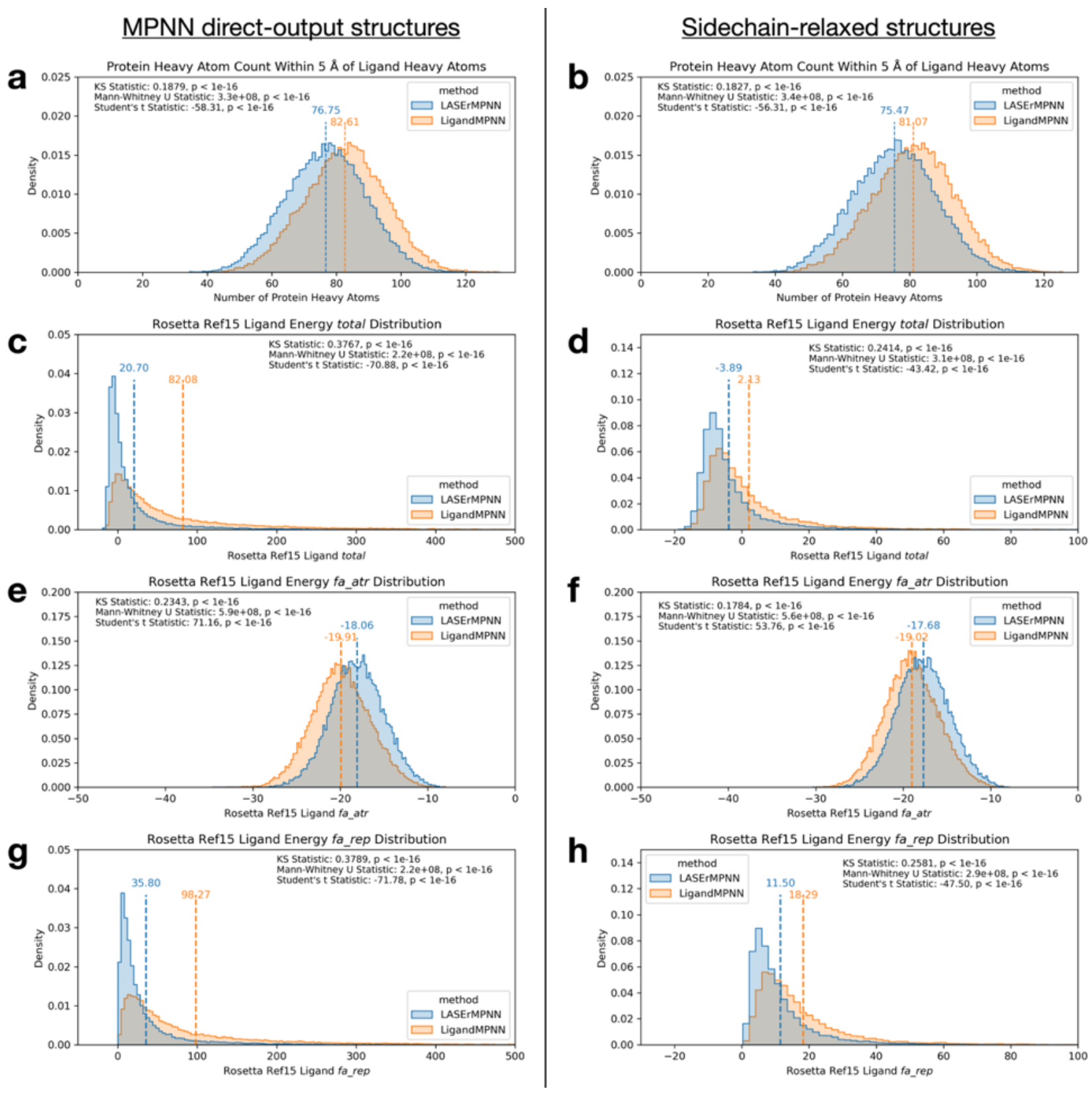


**Extended Data Fig. 1. LigandMPNN generates binding-site sequences that are more overpacked than those from LASErMPNN.**

A shift of focus towards the application case would improve the manuscript, since the achievement of providing a high affinity protein ligand for non-protein small molecule hydrolysis protection with the displayed level of structural as well as functional detail is impressive. To shift the manuscript's emphasis more clearly towards the impressive experimental outcomes rather than primarily focusing on the introduction of LASErMPNN, several additional targeted experiments could be highly informative. For instance, it would be valuable to perform competitive binding assays using EPIC with structurally-related camptothecin derivatives, such as irinotecan, topotecan or camptothecin itself. Such experiments would directly demonstrate EPIC's selectivity for exatecan, highlighting the designed protein's ability to discriminate subtle yet therapeutically relevant structural differences.

We measured binding affinities for a suite of structurally related camptothecin derivatives ("tecans") (Fig. 3g), which showed that EPIC binds most tightly to its target ligand, exatecan. As the tecans become more dissimilar to exatecan, the binding affinity with EPIC correspondingly weakens, showing that EPIC is thermodynamically selective for exatecan. We note that each of these binding titrations used the unmodified ligand because each of the tecans are intrinsically fluorescent, which is a practical convenience for performing the experiments. Therefore, we did not need to perform competition experiments to measure binding affinities. (Please note that experiments that monitor mixtures of various tecans with exatecan would show the same results, as affinity is a thermodynamic parameter; and relative affinity defines specificity.) Lastly, EPIC showed no binding to irinotecan and to fluorescently labeled versions of apixaban and dexamethasone (unrelated small molecules).

Furthermore, a functional enzymatic assay examining human topoisomerase I inhibition by exatecan alone and in complex with EPIC would clarify whether EPIC binding preserves or enhances the biological efficacy of exatecan, bridging the gap between biophysical affinity data and direct biological relevance. Cell-based cytotoxicity assays, for instance employing colorectal or ovarian carcinoma cell lines sensitive to camptothecin derivatives (e.g., HCT116 or OVCAR-3), could similarly unravel the practical therapeutic potential of EPIC–exatecan complexes.

Instead of shifting the focus of the paper into the potential therapeutic aspects of EPIC, we decided to pursue experiments that tested the NISE design algorithm on a different ligand (see **Fig. 6**, reproduced in a response above, and **Extended Data Fig. 9**, reproduced below). Because our paper is offering a new design method with unprecedentedly high success rates, we thought it might be interesting to the reader to see the design pipeline performed again on a second ligand, and with an entirely different protein fold other than helical bundles. We thus performed the NISE design pipeline from scratch for a different target, apixaban. Here, we used the same protein backbones – the mixed alpha/beta NTF2 folds – that Baker's group used in their attempt to design apixaban-binding proteins via LigandMPNN and Rosetta[1]. Using a subset of these same backbones, we ran a few NISE trajectories using LASErMPNN and Boltz-2 and ultimately chose 6 proteins to experimentally test (see section "Design of apixaban binders using NISE" and supplementary text for details). 5 of 6 designs bound apixaban very tightly (1 design with $K_d$ of 80 pM, three designs with $K_d$ between 1 and 10 nM, and one with $K_d$ of 50 nM), which is unprecedented. The best NISE binder, which we call APEX, bound the drug with low pM $K_d$, the tightest small-molecule binder ever designed by computation, to our knowledge. This extraordinary result is further emphasized when comparing with that of Baker's recent paper where only 1 of over 9,000 experimentally tested designs bound apixaban, with a $K_d$ of 700 nM (~10,000-fold weaker affinity than APEX). We therefore think these new experiments are exciting enough to include in the main text to further illustrate the importance and generalizability of the NISE design algorithm.

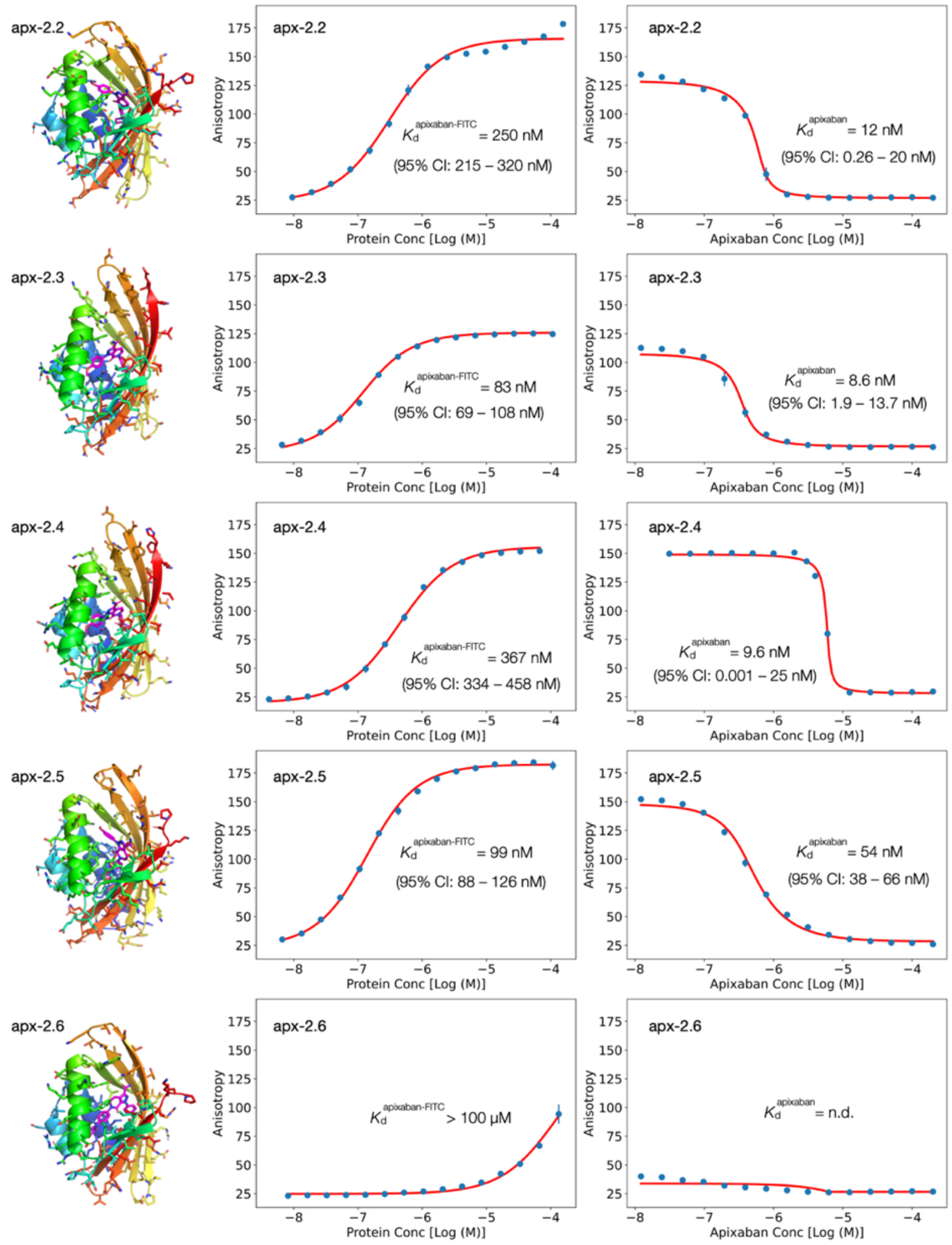


**Extended Data Fig. 9. NISE designs bind apixaban with high affinity.**

Finally, conducting detailed structural overlays between the EPIC–exatecan crystal structure and previously reported exatecan–topoisomerase I complexes (e.g., PDB: 1T8I) would explicitly illustrate the structural novelty or mimicry inherent in EPIC's mode of binding, adding significantly to the mechanistic and biological narrative of the manuscript. It should clearly be stated that not all of these are necessary, but some could help further substantiate the relevance to the general audience.

We now include a structural comparison in **Extended Data Fig. 3**, reproduced below, which shows that EPIC has a unique binding site and structure compared to Topoisomerase I. We also report structural comparisons for our new apixaban-binding protein, APEX, and apixaban's native target, factor Xa, as well as a de novo apixaban-binding protein previously designed via COMBS ($K_d$ = 600 nM). APEX has a unique binding site and structure (**Supp. Fig. 34** is reproduced below).

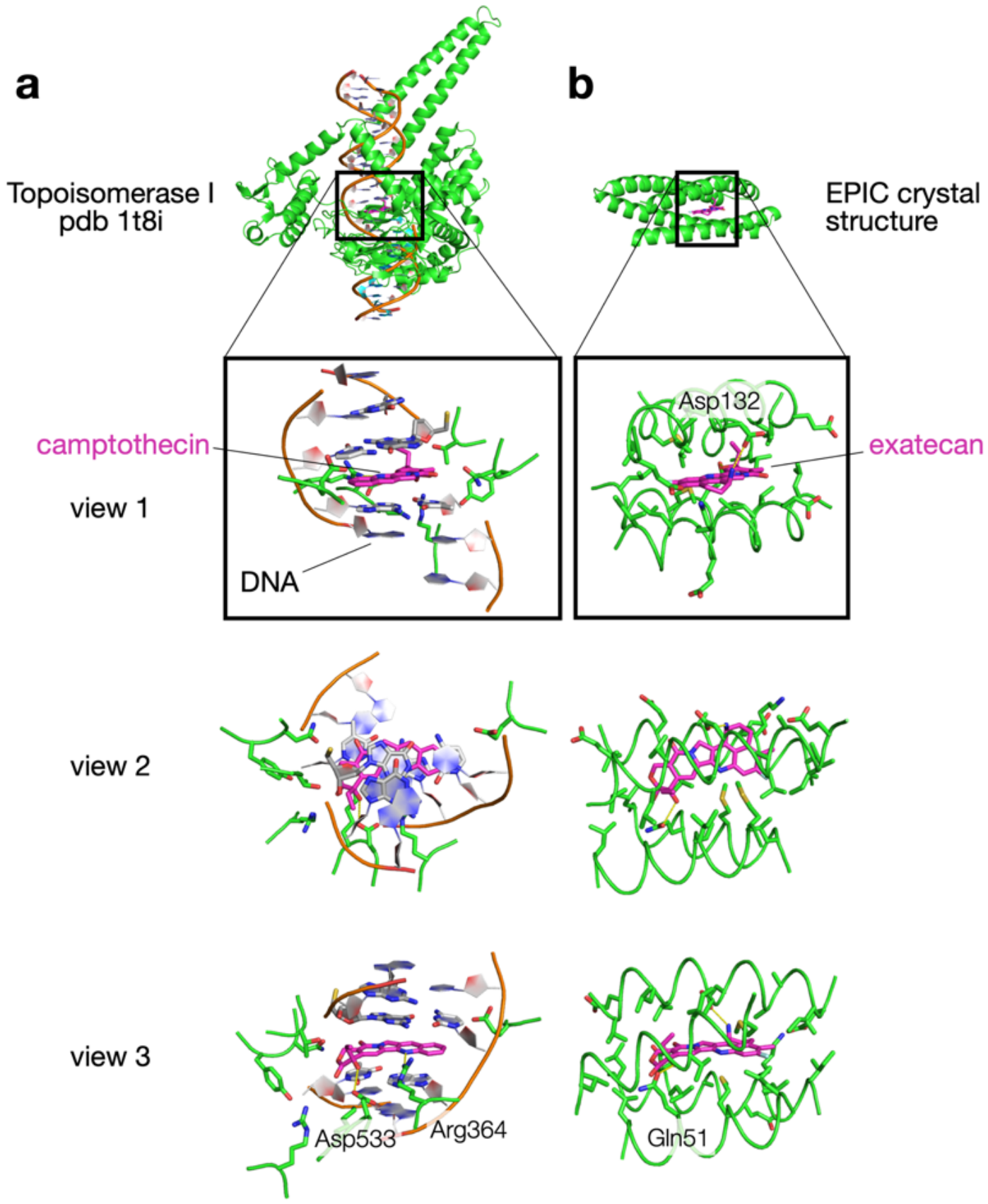


**Extended Data Fig. 3. Binding sites of camptothecin in Topoisomerase I and exatecan in EPIC are dissimilar.**

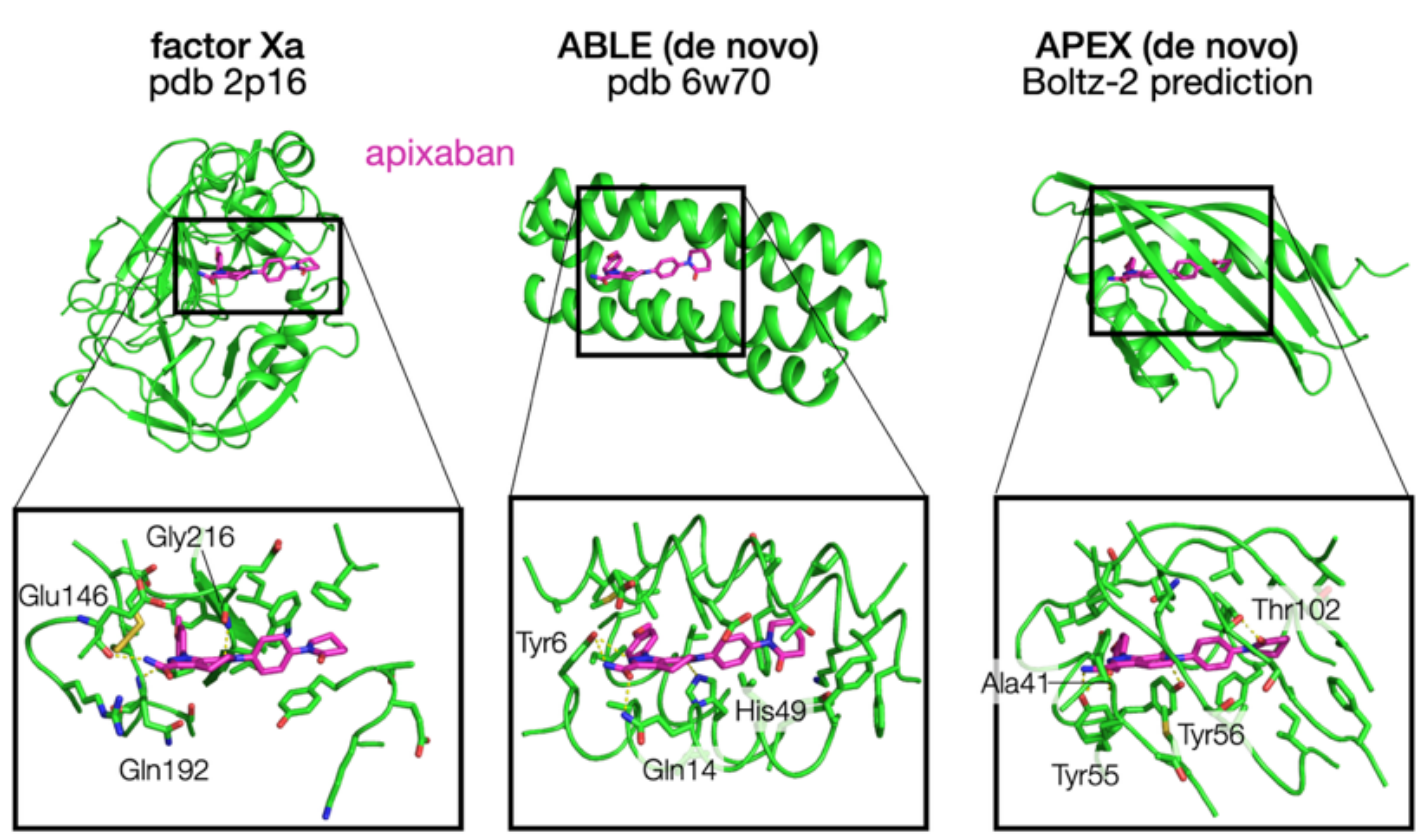


**Supplementary Fig. 34. Binding sites of apixaban in natural and de novo proteins are dissimilar.**

It would also be informative to test NISE on structurally similar but non-binding "decoy" ligands, to quantify and highlight the specificity of the self-consistency selection method clearly.

We co-folded the sequences of the EPIC lineage (see above) with camptothecin and irinotecan (see **Supp. Table 2**, reproduced above). The results showed predicted selectivity for exatecan at every iteration of NISE over camptothecin and irinotecan, as exatecan was consistently predicted with higher ligand pLDDT. We added discussion of this important topic to the paper.

Lines 346-350:

Although we did not explicitly bias against off-target ligands during design (i.e. negative design), assessment of the EPIC lineage from the NISE trajectory showed a consistently low ligand pLDDT when co-folded with the off-target ligand, camptothecin, while that of exatecan progressively increased, in agreement with experimental results that EPIC binds exatecan most tightly (Supplementary Table 2).

Further, the authors should conduct ablation studies of self-consistency filters and systematically vary the thresholds or remove individual filters (such as ligand RMSD, Cα-RMSD, ligand pLDDT) to identify clearly how each impacts the final selection and success rates.

We re-ranked the exatecan NISE designs by each of these metrics individually, then noted metrics for ligand heavy-atom RMSD, protein Ca RMSD, ligand and Ca pLDDT, and number of buried, non-H-bonded polar atoms (BUNs) metrics for the top 3 re-ranked designs (**Supp. Table 9**, reproduced below). We found that, due to the high degree of correlation between the parameters, the corresponding metrics tended to stay favorable even when only sorting on one parameter. Because protein designers usually seek to have "self-consistency" as a goal in protein design, we do not recommend dropping any of these filtering metrics. Our self-consistency cutoffs were chosen such that locations of the ligand during sequence design and after structure prediction could still plausibly form the intended molecular interactions. We describe the ablations in the supplement in section "Ablations of Filtering Metrics" (pg 28).

**Supplementary Table 9. Ranking ablations: top-ranked designs from NISE trajectory that generated EPIC, selected by re-ranking designs independently by RFAA confidence or self-consistency metrics.**

| criterion-rank | Ligand RMSD (Å) | Ligand pLDDT | Ca RMSD (Å) | Protein pLDDT | BUNs Score | sequences |
|---|---|---|---|---|---|---|
| ligand_rmsd-1 | 0.136 | 0.916 | 0.678 | 0.895 | 14 | SDLEKEIDELLTKLKELGKELEEALAALRKAVAEKDREAAEAARDRLLALQDETLILLKELKETVEKLKREVQKIDTPRAEEKVRILDDLLKSIEKLIDIVKEGRTILQDVKDLEELAKALEKVEKLVEEANKEAKNVVKNHDKYKEI |
| ligand_rmsd-2 | 0.141 | 0.932 | 0.387 | 0.9 | 14 | VDLEEKIDEIIEKLIEIGKELEQALKDLTKAVEEQDVEKAKKALKALLELLEEELKLLGELEETVKELLEKIKKIKTPEAKEKVEILEKMLEAIEKLTGIVKKGLEILKEVKNLEELQKAVKQVEKLVKEADIEAEKVIKLHDEFKAI |
| ligand_rmsd-3 | 0.148 | 0.923 | 0.583 | 0.885 | 11 | KDLEEEIDKILDELKKLGKKLEEALEKLKKAVEEENIEAAEKALKELIELQEKELELLEELKKTVESLLEEIKKKDTPEAKEKVLILENLLKAVDKLIKIVKKGLEIIEKTKDMEELKEAIKEIEELVEKANIEAEKIIEEHNKLKSI |
| ligand_plddt-1 | 0.91 | 0.946 | 0.561 | 0.877 | 11 | KELLEEIGRIINKLIVIGQKLETAYSKLKTAAEKNNIEAAEKALAELKKLIDELTVELEKLKKLLEELLEKQKELNSEEAEKYVKLIEEALDAVEQLEKIVDQGKKIIEKVTDLEELKTALNEIEVLQNQANVEAAKLVELYNELLKY |
| ligand_plddt-2 | 0.429 | 0.943 | 0.692 | 0.9 | 10 | VDKEKQIDEIIAELTEIGAELEKALEELKEAVENRDIAEAEKALKKLLELIEEQLVLLEELNETVESLKKEVEKEETEKAKEKVEILDKLLESIEKLQAIVEEGKKILEEVKDLDELAEAIGEVEELVVKADAEAKKVVDEYKKLKAI |
| ligand_plddt-3 | 0.282 | 0.941 | 0.598 | 0.907 | 13 | VDKIKKIGEILKKLIELGKELEAALKALKEACEEENTTAAEAALEKLLALIEEELVLLGELKKTVEELLEEIKAIDTPEAKQKVEILDELIKAIEKLIEIVEEGLKILKETSDLTELREAIKKVEELVVEADIEANKIIDYYKEFKAI |
| protein_rmsd-1 | 0.373 | 0.915 | 0.207 | 0.893 | 10 | VDLDAKVDSIVSELTEIGKKLEKALEKLKKAVDNKDVQEAEKALKELLELIKKLLELLKELKKTVEKLLEEVKKISSPEAKQKVEILEKLLKAIKKLIEIVEEGLKILEECKNLEELKAAIKEVEELVKEADKEAKKVVEELEKFKAL |
| protein_rmsd-2 | 0.369 | 0.896 | 0.213 | 0.898 | 10 | VDKEAVIDDILDKLQTLGKELTQALKDLEKAVEEKNVDEAEKALKKLLALIDETLVLLGELRKTVEDLLEEVLKIESPKAQAKVEVLTQMLKAIDKLIDIVKKGKQILEEVKNLDELKEAIKKVQKLKIEADQEADKVLREWAAYKAL |
| protein_rmsd-3 | 0.428 | 0.917 | 0.218 | 0.899 | 11 | IDLLAEIDEILKHLKKLGKEAEQALEELKQACEQDDVDKAKEALAKLLALIDEMLDLLDKLKETVEKLLEKIKKINSPEAKKKVEILEKLLEAIDKLIDIVKEGKDILEKVKDIDELRIAIKEVEKLVEEANVEAKKVIELYNELQAI |
| protein_plddt-1 | 0.508 | 0.936 | 0.303 | 0.918 | 11 | EALLEKIDTILDELKLIGEELEVALAELKAAAEAEDVEAAAAAKAKLLALIKEQKVLLDELKAAVEQLLAQEQQLDTEEARQKVEVLQELLAAIDDLEVIVDIGQAILEATKDLAELKEAIAIVEKLVVEADKQADKVLKLYDELKAI |
| protein_plddt-2 | 0.39 | 0.924 | 0.377 | 0.916 | 12 | VDLESEIGSILNQLEELGSQLSAALAALTAAVAAKDKAAADAALAELLALIEQELILLSQLLETVKQLKEKVLKINSPKANKKVEILDKMLEAIEKLIDIVQEGLTILREVKDLAELAAAIERVAALVAEADVIAKEVLDNYKKFKDI |
| protein_plddt-3 | 0.323 | 0.933 | 0.302 | 0.915 | 12 | VDLDAVIDRILEELQTLGEEAEAALAELKAAVAEKDVAAAEAALARLLAIQQRMLTLLGELHATVSKKLEEIKKINSPAAKKRVEILEALLEAIAALIGINQQGLEILQAVRDLEELAEAIKQVEELVAQADEHAAAVIALYDADKQI |
| EPIC | 0.726 | 0.911 | 0.44 | 0.896 | 9 | EDLIKKIKEIIKKLKELGAELEKALDRLKKAAEAKDIEAAEEALAELLKLQEEMLVLLEELKATVEKLLEEVRKIDTPAATKKVEVLEKLLDAINKMIDIVKEGLKILKEVKDLEELEEAIKEVEVLVKEADKYAAEIEEEYAKYLAI |

Detailed line-by-line feedback:

Lines 131-132: While the higher sequence recovery rate (53% to 38%) with biotin ligands present is encouraging, there is no classification of the already known sequence-generating MPNNs, such as the previously mentioned LigandMPNN. The authors should provide details for the sequence recovery rate for these alternative inverse folding models with and without ligand context.

We agree this would be a nice analysis. Because LigandMPNN was trained on streptavidin proteins, we retrained LigandMPNN on the same dataset as LASErMPNN, with the streptavidin fold rigorously held out from the training set. Under identical sampling conditions, LASErMPNN achieved an average binding-site sequence recovery of 53%, whereas LigandMPNN achieved an average recovery of 36% (**Extended Data Fig. 2,** reproduced below). Sequences designed by LASErMPNN were on average more self-consistent (compared to the input crystal structure) with respect to protein Ca RMSD and ligand heavy-atom RMSD than those from LigandMPNN (**Supp. Fig. 4**, reproduced below). While LASErMPNN outperformed LigandMPNN in this case, we caution against inferring general performance from this single-protein assessment. For a more general assessment of comparative performance, we refer to the performance on the heldout test set of proteins (Fig. 2b and Supp Figs. 35–37).

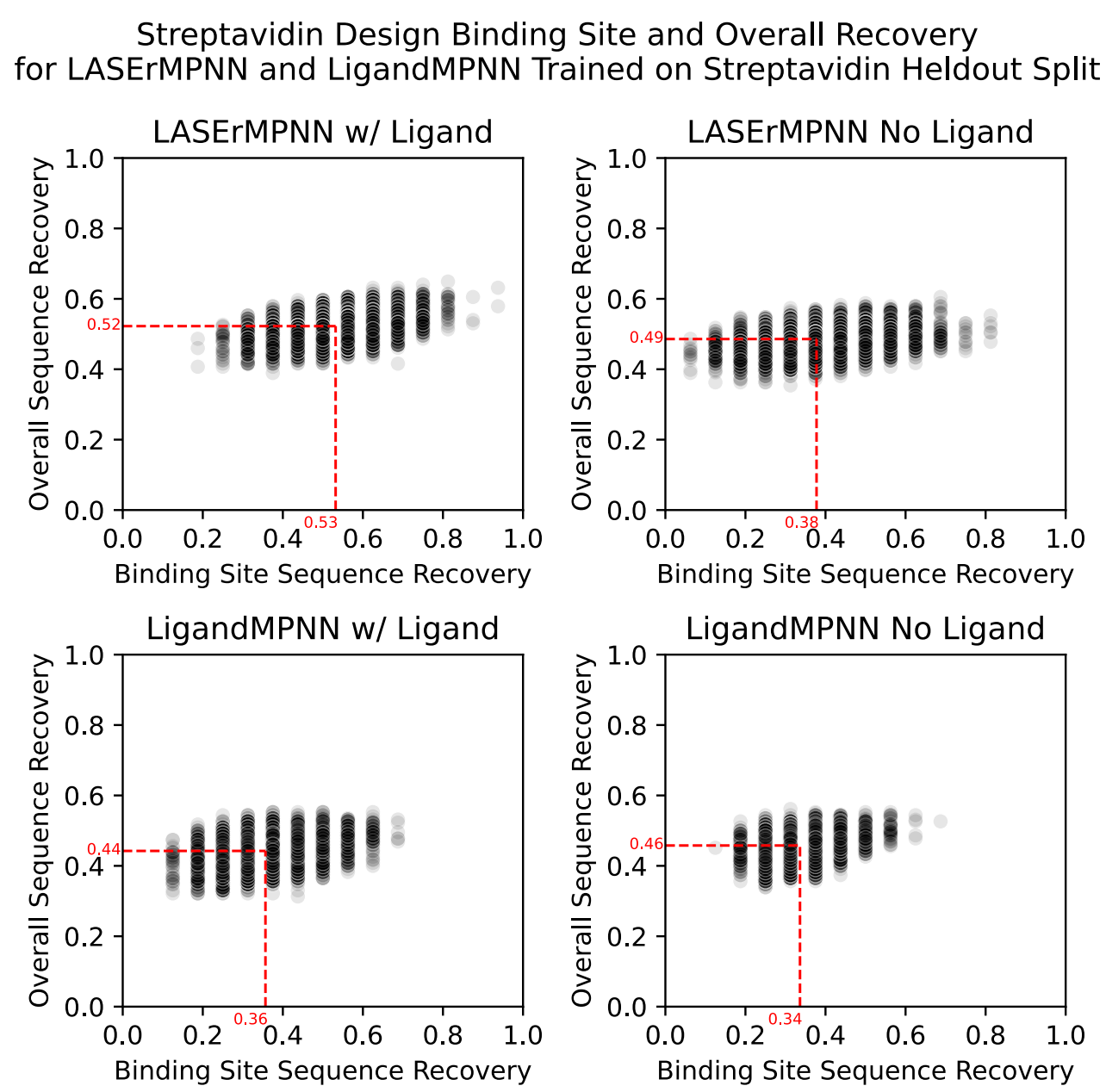


**Extended Data Fig. 2. A LASErMPNN model trained on the streptavidin-holdout dataset outperforms a reimplementation of the LigandMPNN model trained on the same dataset.**

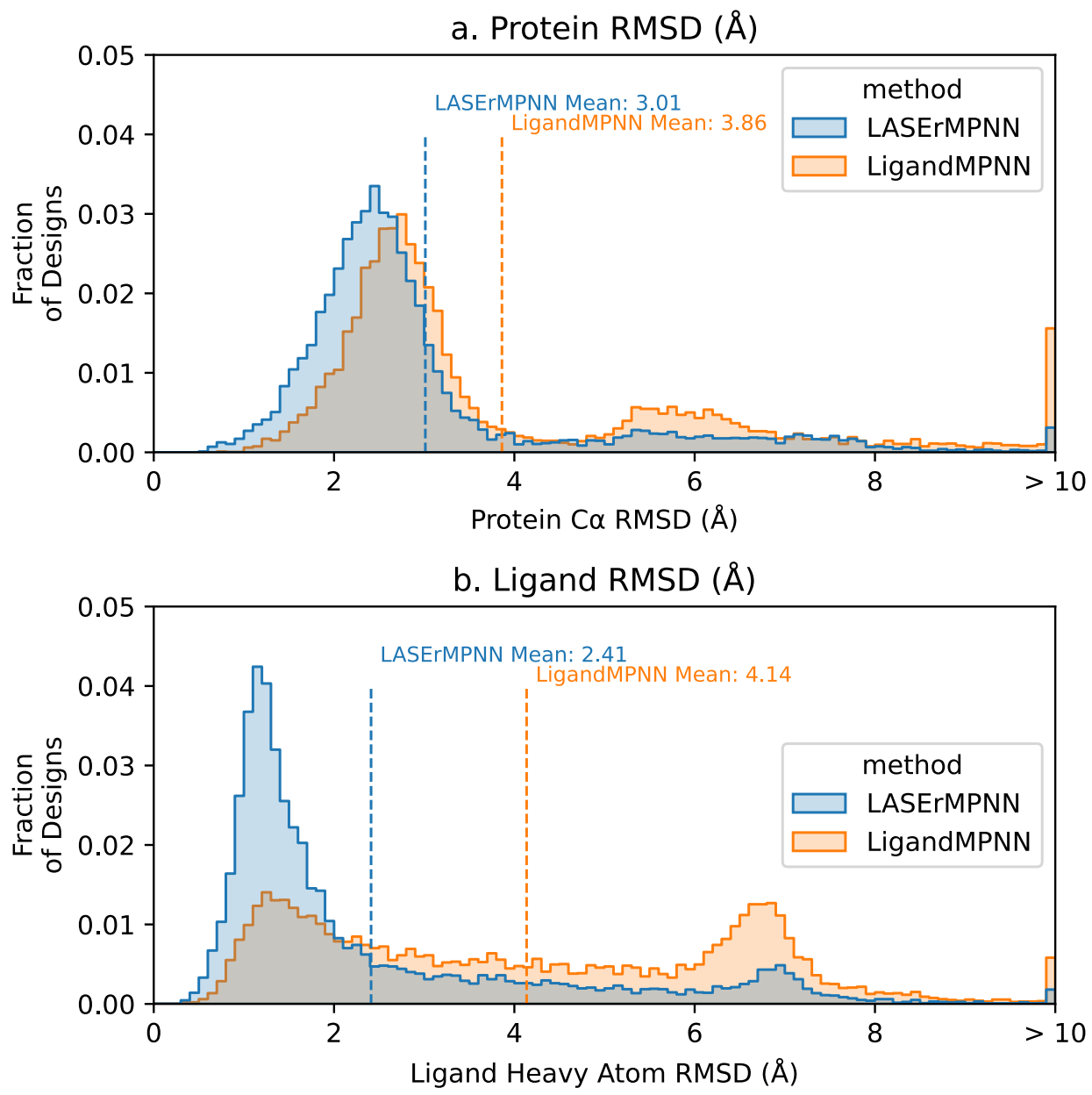


**Supplementary Fig. 4. Co-structure predictions of sequences designed by LASErMPNN more closely resemble the monomeric streptavidin structure than those from LigandMPNN.**

Furthermore, the authors should describe the physical chemical nature of the diverging residues. If the streptavidin-biotin sequence recovery test is a standard assessment in literature, the authors should include respective references.

Sequence recovery of streptavidin bound to biotin is not a standard benchmark, but it is a famous tight interaction well known to a general audience. We use streptavidin and biotin as an anecdotal example. It provides a good touchstone for readers to understand the model's performance. While we do not expect 100% sequence recovery in any individual protein, such as this, we note that the high recovery here for this evolutionarily optimized binder is promising, and the differences in residue identities are biochemically plausible.

In the LASErMPNN design with highest binding-site sequence recovery, the two residues missed by LASErMPNN were a surface exposed Ser25 (LASErMPNN designed a His here) and a buried Phe130 (LASErMPNN designed a Trp here). Residue 25, being a solvent exposed position, shows variability in a multiple sequence alignment (MSA) of streptavidin sequences, including a His at this position for some sequences, so deviation at this site is expected. (We include the MSA in zenodo.) This residue does not make any specific contacts with the ligand. Residue 130 is more conserved, as it is a buried residue. LASErMPNN puts a similar hydrophobic residue here (Trp) and also mutates a few nearby residues to accommodate it. Trp is also observed in the MSA at this position. Thus, the residues "missed" by LASErMPNN were quite plausible as determined from a MSA and from similar biochemical qualities of the sidechains, and are therefore consistent with ligand binding. We added the following text to the main document.

Lines 158-161:

> "The top 25 streptavidin designs showed up to 94% binding-site sequence recovery (out of 10,000 total designs, Fig. 2c), with the few residues in disagreement being biochemically plausible and represented in a multiple sequence alignment (see supplement)."

Lines 175-176: The authors describe the process of scaffold generation prior to their main work. It would be interesting to know why particularly alpha helices were used as scaffold libraries. They are quite common in literature due to the inherent bias of RFdiffusion-based de novo shape generation. However, since the library was curated by the authors, it remains unclear why alpha helices were preferred.

We describe the protocol for scaffold generation in the supplement (see pg 14, "Family-wide Hallucination of Four-helix Bundles"). We added text to the main article that summarizes the key motivations to using helical bundles for this design task.

Lines 199-202:

> "Helical bundles are highly designable scaffolds[42] that contain a central cavity that can fit a variety of small molecules. Their small size, high designability, and thermostability make them attractive scaffolds for de novo binder design[5,6,29,43]."

Furthermore, to show that our design method does not depend on using helical bundles, we now include results using mixed alpha/beta NTF2 scaffolds to design binders to apixaban (see **Fig. 6** above). NTF2 scaffolds, like helical bundles, are designable scaffolds that contain a central pocket. The NTF2 fold is more commonly observed in nature as a small-molecule binding scaffold than are helical bundles.

Lastly, we prefer using small, precomputed libraries of scaffolds (50 or less backbones) for small-molecule binder design because the proteins can be rigorously vetted for designability beforehand. That is, the proteins need to fold correctly in order to bind the ligand. And it is generally beneficial if a backbone can be encoded by many sequences. The presence of a destabilizing cavity in the protein often demands more of the fold and sequence than mini-binder design for protein targets, where the binding interface is on the surface of the protein and the entire core is well packed. Other methods, such as RFdiffusion and boltzdesign1 can, in principle, be used to generate custom backbones for ligands, but, in our hands, these methods produce backbones that are largely not designable and would fail many of our filtering metrics. We have deemed that using RFdiffusion or boltzdesign1 does not currently justify the added computational cost, which is significant, as most – even all – backbones generated by these methods would ultimately be discarded. We have added additional text in the Discussion around this subject (e.g., lines 671-682).

In summary, our current approach is to first attempt to find a good binding solution using our precomputed, highly designable scaffolds, as these would then have a good chance of folding (and thus subsequently binding the target). Many ligands can fit inside either helical bundles or NTF2 scaffolds, so these two folds alone can cover a large swath of chemical space. In the future, as generative methods of backbones for small-molecule binders improve, we might move to that approach. Either approach is compatible with the NISE iterative optimization protocol.

Lines 183-185: For a skilled person unfamiliar with physics-based models it remains difficult to understand how the exatecan conformations were 'built' from the crystal structure of another molecule and refined using the mechanistic force fields. The authors should describe the process in more detail in the main text and include a structural comparison between the two molecules.

We added a new supplemental figure (reproduced below) illustrating the creation of the exatecan conformers and expanded the supplemental text (pg 13, section "Creation of Exatecan Conformers"). We now refer to the supplemental figure and text in the main text.

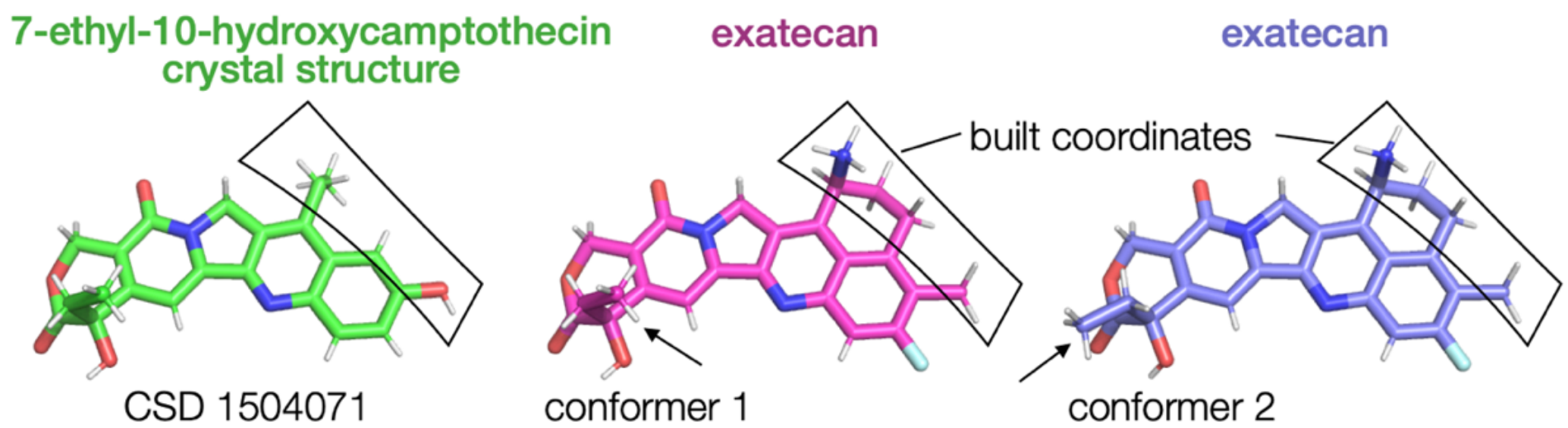


**Supplementary Fig. 7. Building conformers of exatecan.**

Lines 334-336: The relaxed structure obtained using Amber molecular mechanics could have been employed as the initial structure for the NISE algorithm. Why was the relaxation step only introduced during the binder maturation?

The initial structure did not yet have a sequence designed by LASErMPNN, so we did not want to relax it. While this structural relaxation could be carried out in principle at any step within the NISE cycle, we chose not to perform it within NISE because we wanted to test a purely neural optimization. The reviewer might also be wondering why we did not perform the relaxation/proofreading step before ordering the four original sequences. The answer is we did not know if they needed any further optimization at this point. Only after we saw that EPIC experimentally bound the ligand did we look into its further computational optimization. In the future, we can proofread designs before any experimental testing, but proofreading and any energetic relation would usually come at the end of the design protocol, as it is somewhat computationally costly. We note that energetic minimization and proofreading of the binding-site residues is not always necessary, as we achieved an extremely tight apixaban binder (APEX) directly from NISE, without energetic minimization and proofreading.

Lines 340-343: It remains unclear how the authors arrived at the mutations. The author should explain their choice in regard to the model sequence re-prediction.

We chose mutations based on increased probability of the residue at that position, as computed by LASErMPNN using the full sequence and predicted co-structure as context. Of these possible mutations, we added a line in the text that clarifies our reasoning:

Lines 379-381:

> "These mutations were prioritized because they were in direct contact with the ligand and represented both hydrophobic packing and polar H-bonded interactions."

Lines 358-360: The authors state that the binding position of exatecan and the surrounding pocket of their highest affinity binder EPIC deviates from the obtained crystal structure and attribute this to the structure prediction of RFAA. However, the statement is not supported by any argument. The authors should explain their hypothesis in more detail and provide experimental results to underpin it.

We solved an X-ray crystal structure of the exatecan-bound complex of EPIC and compared it to the RFAA-predicted structure. The conformer of the ligand is warped by RFAA relative to the experimentally determined conformer (but less so by Boltz-1 or AF3). The reason that we attribute the discrepancy to RFAA is that RFAA is responsible for predicting co-structure as new input to LASErMPNN during each NISE iteration. If a poorly modeled ligand is input to LASErMPNN, it follows that the discrepancy with the crystal structure is not the fault of LASErMPNN but rather that of the co-structure predictor. We now clarify and quantify our meaning with modified text as well as a new **Extended Data Fig. 4**, reproduced below.

Lines 400-405:

> "The binding mode of the ligand is as predicted, with some expected deviation near the δ-lactone due to RFAA struggling to accurately model the correct bond angles of the ligand in this region (Fig. 4d and Extended Data Fig 4; aromatic-ring superposition of the originally modeled conformer onto that from RFAA shows a 0.9 Å heavy-atom RMSD at the δ-lactone.). Boltz-1 and AF3 more accurately modeled the observed ligand conformer (Extended Data Fig. 5 and see supplemental text)."

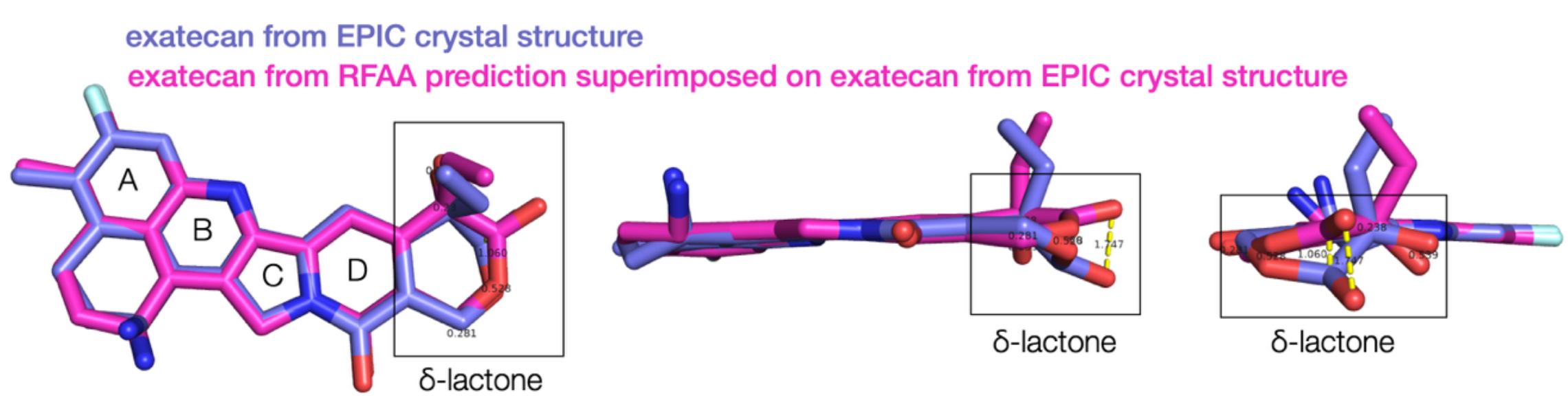


**Extended Data Fig. 4. Differences in the predicted and crystallographic conformers of exatecan.**

Lines 377-389: While being a very valuable analysis between the current state-of-the-art structure prediction models the entire paragraph does not refer or substantiate the novelty of the NISE algorithm or training of the LASErMPNN.

We moved this analysis to the supplement and moved Fig. 5d,e to Extended Data Figs. 5 and 6. This creates some space for our new Fig. 6 that describes the new apixaban binders.

Lines 451-453: Irrelevant sentence with little to no additional value since the argument for the introduction of LASErMPNN is to design and optimise sequences.

We think the line in question (reproduced below) is important:

"We attribute success to the ability to computationally nominate the most designable backbone–ligand structures and then subsequently optimize, filter, and rank LASErMPNN sequences using NISE."

We demonstrate in the paper that the entire NISE pipeline is critical for high success rates. This includes but is not limited to LASErMPNN. Structure prediction models like RFAA or Boltz-2, as well as a means to rank and filter designs, are also essential ingredients to maximizing success in the design of small-molecule binders. We now more clearly state our meaning in the Discussion.

Lines 539-545:

> "We attribute success of zero-shot design to *i*) the ability to computationally nominate the most designable backbone–ligand structures as input to NISE – poses generated via COMBS or even rigid-body docking with a rapid assessment of self-consistency, *ii*) the ability to subsequently optimize the three-dimensional co-structure and sequence, making each iteratively more compatible with the other through selection–expansion, and *iii*) filtering pooled designs by self-consistency, model confidence, and ultimately by a biophysical parameter, i.e. the number of buried, H-bonded polar atoms of ligand and protein."

References.

Dauparas, J., Lee, G.R., Pecoraro, R. et al. Atomic context-conditioned protein sequence design using LigandMPNN. Nat. Meth. 22:717–723 (2025). https://doi.org/10.1038/s41592-025-02626-1

Zhang, Z., Shen, W.X., Liu, Q. et al. Efficient generation of protein pockets with PocketGen. Nat. Mach. Intell. 6:1382–1395 (2024). https://doi.org/10.1038/s42256-024-00920-9

Referee #2 (Remarks on code availability):

Code review

The NISE repository provides the core implementation of the iterative selection–expansion pipeline, but its current setup requires users to manage two separate conda environments (one for LASErMPNN and another for Boltz-1x/2x) as well as manually install ProDy from source. It would be valuable to consolidate dependencies into a single environment.yml (or Dockerfile) that can be invoked with one command.

We have now created a setup.sh script to install a python virtual environment for performing inference calculations using LASErMPNN and Boltz-2. We provide separate environment.yml files for LASErMPNN training in the LASErMPNN github repository.

Additionally, it appears that the current workflow assumes pre-docked protein–ligand PDB files, but the initial docking process is neither implemented nor clearly documented. Including

guidance or references (such as COMBS or Boltzdesign1) would make the pipeline more accessible.

We provide the scripts for running COMBS in Zenodo. We also now provide a new docking script, CARPdock, which was used to dock apixaban into NTF2 scaffolds. We provide an example script for implementing CARPdock on Google Colab.

https://github.com/benf549/CARPdock

https://colab.research.google.com/github/benf549/CARPdock/blob/main/run_CARPdock.ipynb

Finally, expanding the README with a fully worked end-to-end example, complete with directory layout, sample commands, and expected output, would guide new users far more effectively than the current high-level instructions.

We have updated the README with an example.

The LASErMPNN repository is well-organized and straightforward to install and run. To further enhance accessibility and reproducibility, it would be helpful to include example PDBs along with command-line examples demonstrating different use-cases (ideally reflecting the ones described in the paper). A Google Colab notebook that goes through setup and inference would make this tool even more accessible.

We have now created a Google Colab notebook:
https://colab.research.google.com/github/polizzilab/LASErMPNN/blob/main/run_lasermpnn.ipynb

As also noted in the repository as a to do item, publishing the exact train, validation, and test split files along with a script to download and organize the corresponding PDB structures would be a valuable improvement. Finally, proper versioning and distributing the model weights through GitHub releases would help reproduce results more reliably.

We have uploaded the training data and splits in github:
https://github.com/polizzilab/LASErMPNN/tree/main/databases

**References**


1. Lee, G. R., Pellock, S. J., Norn, C., Tischer, D., Dauparas, J., Anishchenko, I., Mercer, J. A. M., Kang, A., Bera, A., Nguyen, H., Goreshnik, I., Vafeados, D., Roullier, N., Han, H. L., Coventry, B., Haddox, H. K., Liu, D. R., Yeh, A. H.-W. & Baker, D. Small-molecule binding and sensing with a designed protein family. *bioRxiv* 2023.11.01.565201 (2023).
2. Polizzi, N. F. & DeGrado, W. F. A defined structural unit enables de novo design of small-molecule-binding proteins. *Science* **369**, 1227–1233 (2020).
3. Lu, L., Gou, X., Tan, S. K., Mann, S. I., Yang, H., Zhong, X., Gazgalis, D., Valdiviezo, J., Jo, H., Wu, Y., Diolaiti, M. E., Ashworth, A., Polizzi, N. F. & DeGrado, W. F. De novo design of drug-binding proteins with predictable binding energy and specificity. *Science* **384**, 106–112 (2024).

We thank the reviewers for their enthusiasm for our work and the helpful comments and suggestions. In response to the referees, we updated the plotting of the binding curves in Figs. 3, 4, and 6, added panels **c** and **d** to Extended Data Fig. 7, and added a new Supplementary Fig. 29. Most notably, we confirmed previous results with repeated experiments and added computational analyses describing P(bind). In order to editorially trim the main text, we moved some discussion points to the supplement, ensuring no major changes in content were made. We provide a marked-up pdf ("_markup.pdf.") of the previous submission, highlighting deleted text in red (due to redundancy) and text moved to the supplement in green font face. Our responses to the reviewers in this document are written in blue font face.

Referee #2:

The authors (Benjamin Fry, Kaia Slaw, and Nicholas F. Polizzi) have thoroughly addressed the concerns we raised previously. In particular, they substantially expanded the comparison between LASErMPNN and LigandMPNN (Ext. Data Figs. 1–2 and Suppl. Figs. 38–39). Beyond reporting overall performance, they now analyze concrete physical determinants of design quality, including side-chain packing density around the ligand and the associated van der Waals attractive/repulsive terms. This additional breakdown is informative and suggests that LASErMPNN can yield lower-density, energetically favorable packing in certain contexts. At the same time, the authors emphasize that the two approaches may be advantageous in different settings rather than one method uniformly outperforming the other.

We're glad our expanded analysis was helpful.

A second major strength that is now clearer is the flexibility of the NISE pipeline. The revised manuscript demonstrates that key components can be swapped without disrupting the overall workflow, particularly the substitution of RosettaFold-All Atom (RFAA) with Boltz-1 and Boltz-2, which improves the pass rate for designs meeting the self-consistency criteria (see lines 464–470). In addition, the authors broaden the set of selection metrics used after sequence expansion, including Boltz-2's binding-related score (reported as P(bind) / affinity_probability_binary, per the Boltz documentation). Taken together, this revision better motivates the authors' claim that NISE is modular and can readily integrate improved structure prediction models and new objective functions as they emerge which is an important practical advantage for the community.

We're glad the modularity of NISE has come across more strongly now and think the paper is much improved for it.

The revision is further strengthened by the additional experimental validation on a completely new target, apixaban. The authors repeat the full process on this independent system and report very high success rates (5/6 designs) and exceptionally strong affinities, including favorable comparison to previously reported efforts against the same target. This added dataset supports the manuscript's broader message that NISE can generalize efficiently with relatively few designs.

However, several points would still benefit from further clarification. In particular, it would be valuable (primarily for community interpretability) to clarify the usability of P(bind) (Ext. Data

Figs. 6–8). The authors already note that P(bind) should primarily be interpreted as a binder vs non-binder signal rather than an affinity-ranking metric, and they further show that it is only weakly correlated with ligand pLDDT suggesting it may provide partially orthogonal information (Ext. Data Fig. 8). At the same time, Ext. Data Fig. 7 indicates an unexpected relationship between P(bind) and affinity (including an apparent negative trend in parts of the EPIC series), which makes its practical interpretation less straightforward. Since P(bind) is used as a key selection/optimization criterion in the apixaban design campaign, it would therefore be very useful for readers to also see the corresponding P(bind)–affinity relationship (as well as ligand pLLDT which showed strong correlation for the EPIC series) for the apixaban dataset. This would help clarify whether P(bind) provides actionable signal in this setting and, more generally, how it should be interpreted in practice (e.g., strictly as a classifier and with what threshold, or whether it adds limited benefit beyond existing confidence and self-consistency filters).

We now analyze P(bind)'s contribution to NISE trajectories by assessing designs with high ligand pLDDT. For our analysis, we added a new Supplementary Fig. 29, reproduced below. We found that designs with high ligand pLDDT had a wide distribution of P(bind). Of these high-pLDDT designs, we found that those with high P(bind) has higher shape complementarity than those with low P(bind). So, while P(bind) itself does not seem to correlate strongly with affinity, we find that its co-optimization with ligand pLDDT tends to push designs towards more well-packed binding pockets. We added some discussion around this in the main text and supplement. We also performed a more focused P(bind) analysis using the experimentally characterized apixaban binders, including this new data in panels **c** and **d** of Extended Data Fig. 7. Like our analysis with the exatecan/EPIC series in panels **a** and **b**, we found that P(bind) can discern binders from non-binders, but cannot differentiate stronger from weaker binders.

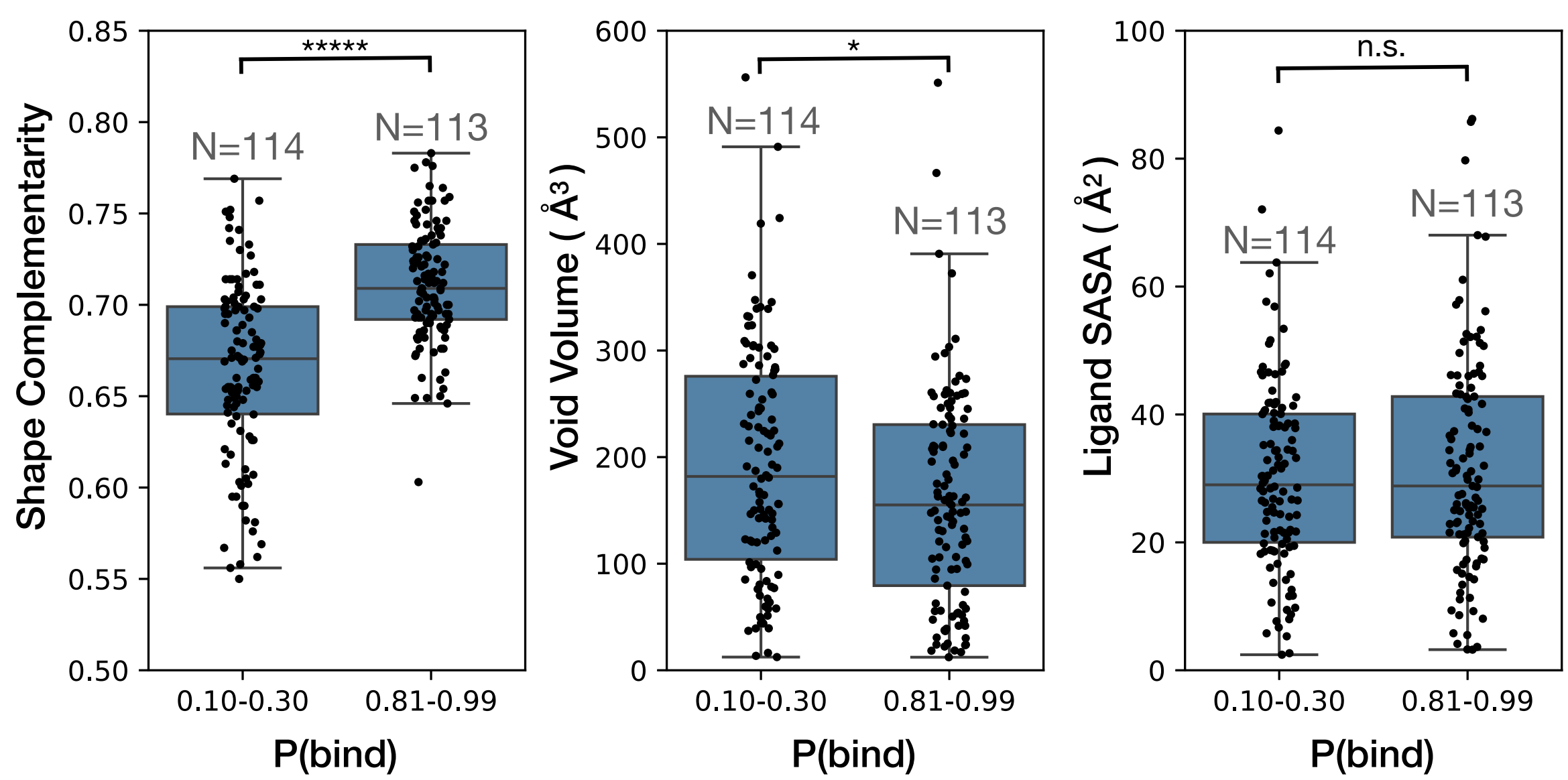


**Supplementary Fig 29. Boltz-2 P(bind) captures information about designed binding site quality.**

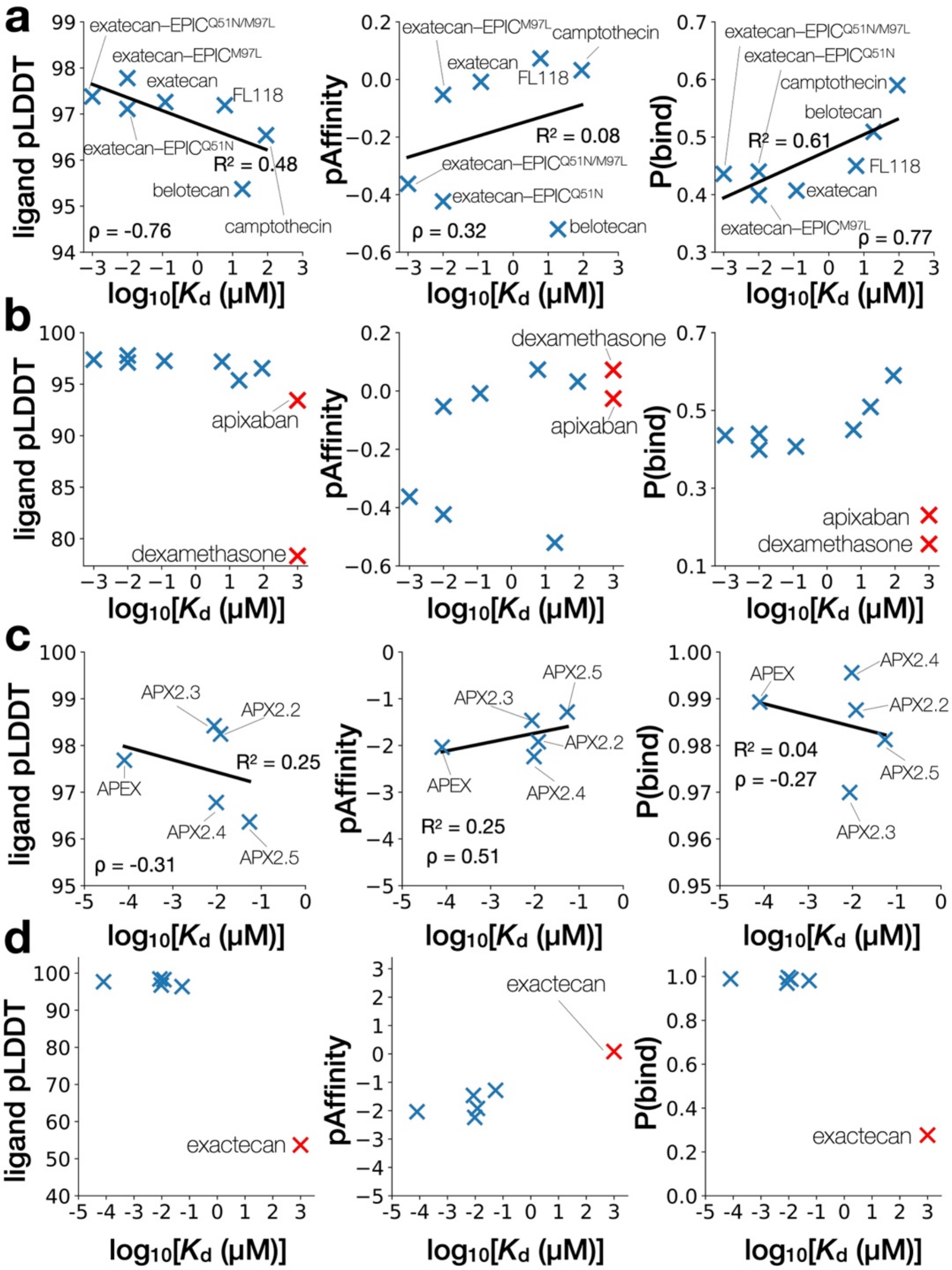


**Extended Data Fig. 7. Correlation between predicted metrics from Boltz-2 and binding affinity of NISE-designed proteins.**

In addition, while Suppl. Table 2 now shows the progression of the EPIC "lineage", it would be helpful for the reader to highlight the exact residues that got changed in each round. The same analysis would be useful also for their new APEX binder.

We have now provided highlights to the residues that changed between NISE rounds in Supplementary Tables 2 and 3. Snippet of Supp. Table 2 below.

| NISE Iter | Sequence |
|---|---|
| **NISE Input** | DELLEALEKAFRELAK<br>VGMTANKIFREMEEAA<br>RRGDEDRLTDGKKKHQ<br>DAAKKMEAILEKIWRI<br>LETIERWLRNIDEERA<br>ATMLELLTKLKRALEK<br>YERIHETFKRIIEAAR<br>TAEEFLALLKILQELS<br>KAADEAAKEIQYLFTQ<br>LLNT |
| **0** | TSLLQELKKLLEELKA<br>QGEKAGKLVNRAKIAA<br>AEGNEEELEECKEALK<br>KAIAEEEAILEEIKKT<br>LEEINNYLLKINSEEA<br>ENQIKLLDDLIAALEE<br>FENIVKKAKALIEKSK<br>NCEEFLKLEKEIDELT<br>KKADEAAKKIIKLYNE<br>LLSE |
| **7** | NELLEELKNLLEELKE<br>VGEKAKELVNAAKVAA<br>ENGDQEAFNACKEELK<br>KAVKEMKSILKEIEEK<br>LTSLREHLESIDSDKA<br>AAQLKLLDELEKALAK<br>FKAIVDKTVALIEESK<br>DCETALKLLDDINELV<br>KEADEAAAEIMELFTK<br>LLDT |

From a practical perspective, the Github repository is well structured and has meaningful, comprehensible comments that guide the user for both (re-)training of LASErMPNN and the execution of the NISE pipeline using the example input. Furthermore, the authors substantiated and expanded the notes on and protocols of the initial target preparation by providing a detailed secondary Github repository for the rigid-rigid backbone docking using CARPdock, which includes the apixaban as exemplary case study. On that note, when we tested both Google Colab implementations of (1) CARPdock and (2) 1-round NISE, we found them to run smoothly and the opportunity to inspect the initial input as well as the output structures/sequence is helpful for the user.

We're glad to know the code ran smoothly and appreciate the testing.

The installation of the separate LASErMPNN installation using cuda 11 was clear and was simple to integrate with an existing Boltz-1x installation. For the combined installation within the recommended virtual environment, we found that prody and rdkit were not called in the setup bash script but had to be installed separately. These two packages should be listed in the initial setup.sh as well.

We have now listed prody and rdkit in the setup.sh. Thank you for bringing this to our attention. https://github.com/polizzilab/NISE/blob/main/setup.sh

A more pressing point is the unclear guidance for running NISE. If the user is not following the example but sets their own input structure the recommendation in the readme file is to manually create folders and edit paths in the underlying python scripts. This is a rather unusual approach for code shared in a public repository. A more reasonable and user-friendly alternative would be to use arguments that parse this custom information. These arguments should be embedded in executional bash scripts with examples. We see this as a necessary improvement before the NISE

framework can be presented to the broader community. The GitHub repository of deepmind/alphafold3/input.md and the RFdiffusion3 Documentation - foundry 0.1.7 documentation are great examples of user-friendly and easy-to-edit campaign customisation guidelines.

We have now amended the NISE script so that it takes user-defined input/output paths and provide examples of usage.

https://github.com/polizzilab/NISE/blob/main/run_nise_boltz2x_cli.py

Overall, we believe the revised manuscript will be a valuable contribution for the community, particularly because it presents a flexible and modular pipeline that achieves strikingly high experimental success rates. The manuscript is also strengthened by the very rigorous and comprehensive analyses, with extensive supporting data that substantiate the main conclusions throughout. The authors appropriately frame the primary novelty as arising from the NISE framework rather than from a single component model. In this context, it is important to be explicit that while LASErMPNN shows clear advantages in specific analyses, it does not appear to represent a dramatic across-the-board improvement over LigandMPNN. The key remaining question is therefore whether the conceptual advance of the NISE framework as an iterative, modular integration of largely established design and structure-prediction tools constitutes sufficient novelty on its own. In our view, this case can be made due to the direct practical impact it can provide; the ability to swap components, incorporate new metrics, and achieve high experimental hit rates with relatively few designs is very useful and can easily integrate within existing design frameworks.

We thank the reviewer for their enthusiastic support of our work.

Referee #2 (Remarks on code availability):

See main comments above.

Referee #3:

To design new ligand binding proteins, Fry, Slaw, and Polizzi demonstrate a combination of two neural networks in an iterative design algorithm (NISE) for joint optimization of ligand-protein pose and protein sequence. They develop a new ligand-specific MPNN (LASErMPNN) for this purpose; they show zero shot design of nM and pM binders for two different small molecule drugs. The hit rates for the process are high, affinities and specificities excellent and consistent with the binding modes, and the biochemistry, structural biology, and biophysical characterization are exquisite.

We thank the reviewer for their enthusiasm for our work.

This paper has been a preprint for a while; this current version is fairly polished. As such, I have very minor experimental details, statistical methods, and a code availability statement to note. In

terms of the overall impact, the strength of this paper is the use of joint optimization of sequence and structure in protein design. While the field moves too fast for me to state for certain, at the time of of the preprint this appeared to be a novel contribution. The main limitation of this method is that a realistic ligand-protein backbone pose must be given as input; this limits generalizability to non-specialists interested in their task-specific problems (though it appears quite useful to specialists!).

Thanks for this feedback. Actually, we think NISE should be accessible even to non-specialists. (That is our hope/goal, at least.) We try to make clear in the revised paper that our workflow will create the docked pose for the user. Our NISE protocol outlined in the GitHub tutorial does this, using our brute-force docking script, CARPdock:

https://github.com/benf549/CARPdock#suggested-workflow

Lines 480–483 of main text:

> Moreover, tight binding was achieved by NISE using vastly different sequences and binding poses (Extended Data Fig. 9 and Supplementary Table 4). Notably, specialized docking methods were not needed; brute-force rigid-body docking sufficed to seed NISE.

Additionally, one could argue that this method is more of an 'affinity maturation' method rather than a true zero-shot design given that other algorithms p(sequence|structure) give binding hits with these realistic poses (albeit at weaker affinities). I think these are minor quibbles - overall, the paper presents a new type of protein design algorithm for which utility is demonstrated with strong experimental data.

We thank the reviewer for the great feedback and support. We'd like to emphasize that NISE created the high-affinity apixaban binders in a zero-shot fashion. 5 of 6 tested designs directly from NISE had dissociation constants less than 50 nM (and one was 80 pM). These designs did not need any affinity maturation, even by computational "proofreading". We therefore think our method should be general for both zero-shot design and for affinity maturation of de novo or natural small-molecule binders.

Minor things:

1. Fluorescence anisotropy data. The data shown in Fig. 3f,g and panels do a disservice to the global fitting done for the anisotropy data as reported in the SI. Specifically, the traces shown are truncated before they are saturated (e.g. EPIC binder panel 3f), and error bars and statistical methods are not reported.

We apologize that the (small) error bars were not originally visible beneath the large data markers. We now thinned the lines and reduced the marker size to show the error bars (standard deviation of 3 technical replicates), which are now reported in the figure legend. We made this plotting format consistent throughout the binding curves presented in Figs. 3, 4, and 6.

Re: binding saturation, we saw some light scattering at high protein concentrations, likely due to the short excitation wavelength (360 nm) used to excite the tecans (scattering obeys a $1/\lambda^4$ power

law). We repeated the experiments but again saw scattering at high concentrations. The dissociation constants for weak binders could be viewed as lower limits. We think this doesn't affect the main conclusions of the paper.

Referee #3 (Remarks on code availability):

Data availability statement (with trainee; "I" refers to the trainee):

I reviewed the LASErMPNN repository (https://github.com/polizzilab/LASErMPNN) and examined the NISE repository (https://github.com/polizzilab/NISE). I was able to install and run LASErMPNN for ligand-conditioned sequence design on a protein-ligand system outside the training set, confirming that the inference pipeline functions as described. The Google Colab notebooks for both tools are well-constructed and substantially lower the barrier to entry. The authors have been responsive to prior reviewer feedback -- the addition of setup.sh for NISE, Colab notebooks, CARPdock for initial pose generation, and training data splits on Zenodo are welcome improvements.

We thank the reviewer for the enthusiastic support.

A few remaining suggestions:

Dependency management: In my experience installing LASErMPNN, the conda environment files do not pin package versions, which can cause installation difficulties given the tight coupling between PyTorch, CUDA, and torch-scatter/PyKeOps. The NISE setup.sh handles this better by pinning key versions. Aligning both repos on pinned dependencies (or providing a Docker/Singularity container) would improve long-term reproducibility.

We have now made a Singularity/Apptainer container for easy use of NISE, with all components pre-installed. We uploaded the container to huggingface and tested it on the cloud. https://huggingface.co/benf549/NISE/blob/main/nise.sif

Details for using the container are now on github: https://github.com/polizzilab/NISE/blob/main/APPTAINER.md

Bundled example inputs: The NISE repository now includes example PDBs and sample commands in the README, which is helpful. However, LASErMPNN still does not bundle any example PDB files -- users must supply their own. Including even a single small example with expected output would allow users to verify their installation immediately.

Thanks for the suggestion. We now include the example of monomeric streptavidin (pdb 4jnj) in the LASErMPNN repo. We use this as a test example for checking the package installed properly: https://github.com/polizzilab/LASErMPNN/blob/main/example_pdbs/4jnj-1_prot.pdb

Testing and validation: Adding a small test suite -- even a smoke test that loads the model, runs inference on a bundled example PDB, and checks output format -- would help users verify their

installation and protect against regressions as the code evolves.

We now use monomeric streptavidin (see above) as a test example for checking the package installed properly.

Overall, both repositories are functional and represent a meaningful contribution to the protein design toolkit. The suggestions above are intended to help maximize their impact and longevity as community resources.

Thanks for the suggestions, which indeed improved the usability of the package.

Referee #5:

I co-reviewed this manuscript with one of the reviewers who provided the listed reports.

Referee #5 (Remarks on code availability):

I reviewed the LASErMPNN repository (https://github.com/polizzilab/LASErMPNN) and examined the NISE repository (https://github.com/polizzilab/NISE). I was able to install and run LASErMPNN for ligand-conditioned sequence design on a protein-ligand system outside the training set, confirming that the inference pipeline functions as described. The Google Colab notebooks for both tools are well-constructed and substantially lower the barrier to entry. The authors have been responsive to prior reviewer feedback -- the addition of setup.sh for NISE, Colab notebooks, CARPdock for initial pose generation, and training data splits on Zenodo are welcome improvements.

We thank the reviewer for the enthusiastic support.

A few remaining suggestions:

Dependency management. In my experience installing LASErMPNN, the conda environment files do not pin package versions, which can cause installation difficulties given the tight coupling between PyTorch, CUDA, and torch-scatter/PyKeOps. The NISE setup.sh handles this better by pinning key versions. Aligning both repos on pinned dependencies (or providing a Docker/Singularity container) would improve long-term reproducibility.

We have now made a Singularity/Apptainer container for easy use of NISE, with all components pre-installed. We uploaded the container to huggingface and tested it on the cloud. https://huggingface.co/benf549/NISE/blob/main/nise.sif

Details for using the container are now on github:
https://github.com/polizzilab/NISE/blob/main/APPTAINER.md

Bundled example inputs. The NISE repository now includes example PDBs and sample commands in the README, which is helpful. However, LASErMPNN still does not bundle any

example PDB files -- users must supply their own. Including even a single small example with expected output would allow users to verify their installation immediately.

Thanks for the suggestion. We now include the example of monomeric streptavidin (pdb 4jnj) in the LASErMPNN repo. We use this as a test example for checking the package installed properly. Instructions for running the pytest test suite have been added to the LASErMPNN GitHub README and an example input PDB file has been added at https://github.com/polizzilab/LASErMPNN/blob/main/example_pdbs/4jnj-1_prot.pdb

Testing and validation. Adding a small test suite -- even a smoke test that loads the model, runs inference on a bundled example PDB, and checks output format -- would help users verify their installation and protect against regressions as the code evolves.

We now use monomeric streptavidin (see above) as a test example for checking the package installed properly.

Overall, both repositories are functional and represent a meaningful contribution to the protein design toolkit. The suggestions above are intended to help maximize their impact and longevity as community resources.

Thanks for the suggestions, which indeed improved the usability of the package.
